# The NPR1 agonist antibody XXB750 in heart failure: a phase 2 randomized trial

**DOI:** 10.1038/s41591-026-04313-w

**Published:** 2026-03-30

**Authors:** Scott D. Solomon, John J. V. McMurray, G. Michael Felker, James L. Januzzi, Carolyn S. P. Lam, Adriaan A. Voors, Brian Claggett, Thomas G. Nuehrenberg, Adel R. Rizkala, Cornelia Koch, Wenyue Zhu, Martin P. Lefkowitz

**Affiliations:** 1 The Cardiovascular Division, Mass General Brigham, Harvard Medical School, Boston, MA USA; 2 University of Glasgow, Glasgow, UK; 3 Duke University, Durham, NC USA; 4 Duke National University of Singapore, Singapore, Singapore; 5 University of Groningen, Groningen, the Netherlands; 6 Novartis Pharma AG, Basel, Switzerland; 7 Novartis Pharmaceuticals, East Hanover, NJ USA; 8 Novartis Pharmaceuticals UK Ltd, London, UK

**Keywords:** Heart failure, Randomized controlled trials

## Abstract

Therapies targeting the natriuretic peptide system have the potential to reduce death or heart failure events in heart failure with reduced ejection fraction. Here we assess XXB750, a human monoclonal antibody activating natriuretic peptide receptor 1, in patients with heart failure. Patients with heart failure and a left ventricular ejection fraction <50% were enrolled. Those patients who were on background angiotensin-converting enzyme inhibitor or angiotensin receptor blocker treatment were randomized to receive 60 mg XXB750, 120 mg XXB750 or placebo in a blinded fashion or sacubitril/valsartan treatment in an open-label fashion. Those patients on background sacubitril/valsartan treatment were randomized to either 60 mg XXB750, 120 mg XXB750 or placebo treatment in a blinded fashion. The primary endpoint was the change in NT-proBNP levels at 16 weeks after treatment initiation, and safety was also assessed. We randomized 136 participants (70% male, 30% female) to 60 mg XXB750 (*n* = 26), 120 mg XXB750 (*n* = 55), matching placebo (*n* = 29) or sacubitril/valsartan (*n* = 25). At 16 weeks, NT-proBNP levels rose (ratio of change from baseline 1.34, 95% confidence interval (CI) 1.07–1.66) and cyclic guanosine monophosphate (cGMP) levels declined (ratio of change from baseline 0.77, 95% CI 0.65–0.91) in the pooled XXB750 arms, whereas NT-proBNP levels declined from baseline (ratio of change from baseline 0.70, 95% CI 0.45–1.10), and cGMP levels rose (ratio of change from baseline 1.38, 95% CI 1.13–1.69) in the sacubitril/valsartan arm. Death or worsening heart failure events occurred more frequently in those receiving XXB750 (25%) compared with those receiving sacubitril/valsartan (8%), or placebo (0%). Because of the excess heart failure events in participants receiving XXB750, the data monitoring committee recommended stopping the trial prematurely. In contrast to the expected mechanism of drug action, XXB750 treatment led to increased NT-proBNP levels, lowered cGMP levels and more worsening heart failure events, suggesting that XXB750 may paradoxically behave as a functional antagonist of endogenous natriuretic peptides in patients with heart failure. Clinicaltrials.gov registration: NCT06142383.

## Abstract (web summary)

In a randomized trial, treatment of patients with heart failure with an antibody designed to activate the guanylate cyclase receptor NPR1 led to an unexpected increase in NT-proBNP levels and worsening heart failure events compared with the control, prompting termination of the trial.

## Main

Despite major advances in the management of heart failure (HF) with reduced ejection fraction, patients remain at substantial risk for death and HF events. Therapeutic strategies for HF with reduced ejection fraction have primarily targeted neurohormonal pathways implicated in disease progression. Natriuretic peptides, including A- and B-type natriuretic peptide (ANP and BNP), are upregulated in HF and, through their action on the natriuretic peptide receptor 1 (NPR1, also known as NPRA) and membrane-bound particulate guanylate cyclase, stimulate natriuresis, diuresis and vasodilation. Elevations in ANP and BNP thus serve as a compensatory mechanism to counteract the adverse effects of activation of the renin–angiotensin–aldosterone and sympathetic nervous systems, as well as other harmful neurohumoral pathways^1^. Although the direct infusion of short-acting natriuretic peptides has not improved outcomes in acute decompensated HF^2^, indirect augmentation of endogenous natriuretic peptide activity by neprilysin inhibition has proven effective in chronic HF, as demonstrated by the use of sacubitril/valsartan^3^. Dosed twice daily, sacubitril/valsartan leads to increases in ANP^4^ and smaller elevations in BNP^5^ by inhibiting their degradation^6^, but sacubitril/valsartan is relatively short-acting, augments bradykinin (resulting in a slight risk for angioedema) and may be less effective in patients with altered natriuretic peptide secretion and processing^7–10^.

Sustained stimulation of the NPR1 receptor offers a potential alternative approach to targeting the NP system. The NPR1 agonist M-atrial natriuretic peptide (MANP), an analog of human ANP, increased cyclic guanosine monophosphate (cGMP) levels and natriuresis and lowered aldosterone levels and blood pressure when administered subcutaneously to hypertensive participants^11^. Similarly, REGN5381, an allosteric agonist of NPR1, reduced systolic blood pressure in both animal models and healthy human volunteers^12^. XXB750, a long-acting fully human monoclonal IgG1 antibody targeting the NPR1 receptor, has been shown to increase plasma cGMP in a dose-dependent fashion and correspondingly reduce blood pressure in healthy normotensive and hypertensive participants not taking antihypertensive medications^13^. XXB750 plasma concentrations peak at approximately 4–10 days and its half-life ranges from ~15 to ~25 days with single subcutaneous doses. Because of the potential for XXB750 as a therapeutic for HF, we sought to assess the safety and efficacy of XXB750 in three doses given in different dosing regimens compared with placebo in patients with HF and left ventricular ejection fraction (LVEF) <50% receiving standard therapy.

## Results

**Fig. 1:**
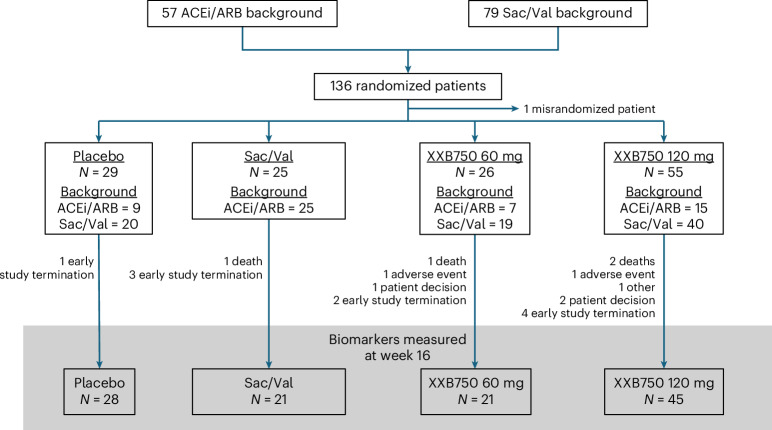
Patient flow diagram. Figure shows the flow of patients and participant disposition throughout the study. Sac/Val, sacubitril/valsartan.

Between 27 December 2023 and 8 August 2024, a total of 136 participants were randomized: 26–60 mg XXB750, 56–120 mg XXB750, 29 to matching placebo and 25 to open-label sacubitril/valsartan. One patient in the 120 mg XXB750 group was misrandomized and was excluded from further analyses. The participant disposition is illustrated in Fig. 1. Baseline characteristics of study participants according to randomly allocated treatment group are presented in Table 1 and were balanced between arms (baseline characteristics by background therapy are presented in Extended Data Table 1). The individuals in the trial were predominantly white (84%), with most participants from Europe or the USA. The mean age was 69.8 years, with a range from 38 to 91 years, and 70% were men. The majority of patients had a history of hypertension (88%), 53% had a history of atrial fibrillation, 44% had a history of diabetes and 36% had a history of myocardial infarction. The mean LVEF at baseline was 36.8% ± 8.1%. The mean estimated glomerular filtration rate (eGFR) was 66.3 ± 22 ml min 1.73 m^−^^2^, and the median NT-proBNP was 1391 ng l^−1^ (interquartile range 956–2,436). The trial was stopped following a recommendation of the data safety monitoring board on 6 August 2024 because of evidence of harm in patients receiving XXB750.

**Table 1:** Baseline characteristics by randomized group

	Placebo	Sacubitril/valsartan	XXB750 60 mg	XXB750 120 mg
	*n* = 29	*n* = 25	*n* = 26	*n* = 55
Age (years)	71.7 ± 11.0	67.3 ± 11.6	69.6 ± 9.7	70.1 ± 10.0
Male sex	21 (72.4%)	15 (60.0%)	17 (65.4%)	41 (74.5%)
Race				
Asian	2 (6.9%)	2 (8.0%)	1 (3.8%)	6 (10.9%)
Black or African American	2 (6.9%)	1 (4.0%)	1 (3.8%)	6 (10.9%)
White	25 (86.2%)	22 (88.0%)	24 (92.3%)	43 (78.2%)
Region				
America	9 (31.0%)	4 (16.0%)	8 (30.8%)	16 (29.1%)
Asia	2 (6.9%)	2 (8.0%)	1 (3.8%)	6 (10.9%)
Central Europe	8 (27.6%)	15 (60.0%)	7 (26.9%)	15 (27.3%)
Western Europe and other	10 (34.5%)	4 (16.0%)	10 (38.5%)	18 (32.7%)
(Baseline) New York Heart Association Class III	8 (27.6%)	7 (28.0%)	7 (26.9%)	17 (31.5%)
Kansas City Cardiomyopathy Questionnaire overall summary score	67.9 ± 21.2	61.0 ± 21.0	61.7 ± 20.9	69.1 ± 23.3
Baseline eGFR (ml min^−1^ 1.73 m^−2^)	66.1 ± 18.0	66.6 ± 20.1	71.2 ± 21.5	63.9 ± 20.9
History of atrial fibrillation or flutter	16 (55.2%)	12 (48.0%)	16 (61.5%)	28 (50.9%)
BMI (kg m^−2^)	30.4 ± 6.2	29.4 ± 5.3	29.6 ± 5.5	29.4 ± 5.9
NT-proBNP (ng l^−1^), serum or plasma	1,269 (981–3,476)	1,421 (1,116–2,562)	1,387 (702–2,512)	1,345 (947–2,309)
Baseline LVEF (%)	36.6 ± 8.0	40.4 ± 6.9	37.5 ± 6.8	34.8 ± 8.8
Medications				
Sacubitril/valsartan (stratification)	20 (69.0%)	0 (0.0%)	19 (73.1%)	40 (72.7%)
ACEi or ARB (stratification)	9 (31.0%)	25 (100.0%)	7 (26.9%)	15 (27.3%)
Aldosterone antagonists/mineralocorticoid receptor antagonists	24 (82.8%)	13 (52.0%)	19 (73.1%)	34 (61.8%)
Beta blockers	27 (93.1%)	23 (92.0%)	25 (96.2%)	52 (94.5%)
Calcium channel blockers	3 (10.3%)	5 (20.0%)	6 (23.1%)	3 (5.5%)
Cardiac glycosides (digoxin/digitalis glycoside)	3 (10.3%)	3 (12.0%)	1 (3.8%)	3 (5.5%)
Loop and thiazide or thiazide-like diuretics	22 (75.9%)	22 (88.0%)	20 (76.9%)	42 (76.4%)
Sodium–glucose cotransporter 2 inhibitors	20 (69.0%)	12 (48.0%)	19 (73.1%)	42 (76.4%)
Baseline diuretic daily dose—furosemide equivalent (mg)	33.3 ± 35.8	33.6 ± 50.5	30.0 ± 40.1	33.6 ± 44.4

### Efficacy results

**Fig. 2:**
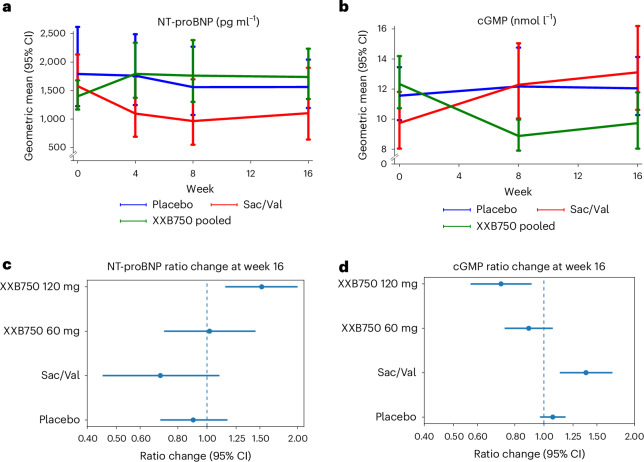
NT-proBNP and plasma cGMP levels. **a**,**b**, Geometric mean values of NT-proBNP (**a**) and cGMP (**b**); 95% CI are indicated. **c**,**d**, The ratio of change from baseline at week 16 for NT-proBNP (**c**) and cGMP (**d**); 95% CI are indicated. The vertical dashed blue lines are the unity lines indicating no difference between baseline and 16 weeks.

**Extended Data Fig. 1:**
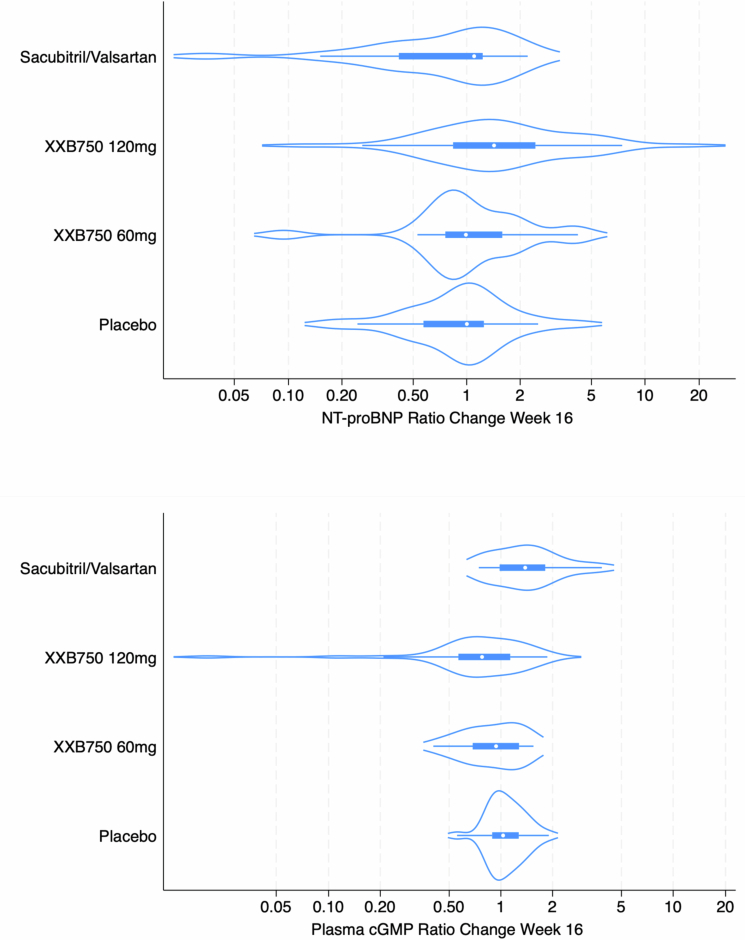
Distribution of NT-proBNP (top panel) or cyclic GMP (cGMP) (bottom panel) ratio change at Week 16 by treatment group. Violin plots display the distribution of the ratio change in NT-proBNP or cGMP from baseline to Week 16 for each treatment group. The width of each violin represents the relative data density at a given value (kernel density estimate), with wider regions indicating a higher concentration of observations. The central box indicates the interquartile range (25th–75th percentiles), and the white dot within the box denotes the median value. The outer limits of the whiskers are determined by the most distant points from the edges of the IQR box that are within 1.5 times the width of the box. The x-axis is shown on a logarithmic scale, where a value of 1 represents no change from baseline; values < 1 indicate decreases and values > 1 indicate increases in NT-proBNP or cGMP.

Between baseline and 16 weeks, NT-proBNP remained relatively constant in the placebo arm (ratio change of 0.90, 95% confidence intervals (CI) 0.70–1.17), decreased in the sacubitril/valsartan arms (ratio change of 0.70, 95% CI 0.45–1.10) and increased in the pooled XXB750 arms (ratio change of 1.34, 95% CI 1.07–1.66) (Table 2, Fig. 2 and Extended Data Fig. 1). Between baseline and 16 weeks, plasma cGMP, a marker of target engagement, remained relatively constant in the placebo arm (ratio change of 1.07, 95% CI 0.97–1.18), increased in the sacubitril/valsartan arm (ratio change of 1.38, 95% CI 1.13–1.69) and decreased in the pooled XXB750 group (ratio change of 0.77, 95% CI 0.65–0.91) (Table 2, Fig. 2 and Extended Data Fig. 1). Similar results were observed for urinary cGMP–creatinine ratio. Moreover, we observed a dose response for both biomarkers, with XXB750 120 mg showing a greater increase in NT-proBNP and a greater reduction in cGMP compared with the 60 mg dose (Fig. 2b). Least-square mean differences between treatment groups and placebo in NT-proBNP or plasma or urine cGMP were qualitatively similar (Extended Data Table 2). The increase in NT-proBNP and the reduction in cGMP with XXB750 were most apparent in those receiving background sacubitril/valsartan, with less meaningful changes observed in those receiving background ACEi or ARB (Table 3). We observed no heterogeneity in the response to XXB750 for either NT-proBNP (*P* interaction of 0.70) or cGMP (*P* interaction of 0.49) according to baseline NT-proBNP concentration (analyzed as quartiles). We observed no changes in systolic blood pressure, heart rate, aldosterone or troponin I in patients receiving XXB750 (Extended Data Table 2).

**Table 2:** Biomarkers and adverse events by treatment group

Biomarker (median, 95% CI)	Placebo	Sacubitril/valsartan	XXB750 60 mg	XXB750 120 mg	Pooled XXB750
	*n* = 29	*n* = 25	*n* = 26	*n* = 55	*n* = 81
NT-proBNP (ng l^−1^) week 0	1,792 (1,205–2,664)	1,579 (1,151–2,168)	1,383 (982–1,947)	1,407 (1,121–1,765)	1,399 (1,162–1,683)
NT-proBNP (ng l^−1^) week 4	1,762 (1,225–2,532)	1,096 (670–1,791)	1,329 (749–2,359)	2,052 (1,515–2,780)	1,793 (1,367–2,351)
NT-proBNP ratio change week 4	0.97 (0.81–1.17)	0.72 (0.50–1.04)	0.96 (0.63–1.47)	1.45 (1.19–1.78)	1.28 (1.05–1.55)
NT-proBNP (ng l^−1^) week 8	1,561 (1,053–2,312)	964 (527–1,765)	1,556 (785–3,085)	1,876 (1,342–2,621)	1,763 (1,294–2,400)
NT-proBNP ratio change week 8	0.86 (0.76–0.98)	0.64 (0.39–1.05)	1.15 (0.63–2.10)	1.41 (1.09–1.81)	1.32 (1.02–1.70)
NT-proBNP (ng l^−1^) week 16	1,563 (1,179–2,071)	1,102 (617–1,969)	1,247 (818–1,899)	2,031 (1,475–2,797)	1,739 (1,346–2,246)
NT-proBNP ratio change week 16	0.90 (0.70–1.17)	0.70 (0.45–1.10)	1.02 (0.72–1.45)	1.52 (1.15–2.00)	1.34 (1.07–1.66)
cGMP (nMol l^−1^) week 0	11.6 (9.9–13.6)	9.7 (8.0–11.9)	10.9 (8.3–14.3)	13.1 (11.1–15.5)	12.3 (10.7–14.2)
cGMP (nMol l^−1^) week 8	12.2 (10.0–14.9)	12.3 (9.9–15.2)	7.6 (6.1–9.4)	9.6 (8.3–11.1)	8.9 (7.9–10.0)
cGMP ratio change week 8	1.07 (0.96–1.19)	1.32 (1.08–1.62)	0.71 (0.56–0.89)	0.76 (0.65–0.87)	0.74 (0.66–0.84)
cGMP (nMol l^−1^) week 16	12.1 (10.2–14.2)	13.1 (10.5–16.4)	9.6 (7.3–12.5)	9.8 (7.5–12.8)	9.7 (8.0–11.8)
cGMP ratio change week 16	1.07 (0.97–1.18)	1.38 (1.13–1.69)	0.89 (0.74–1.07)	0.72 (0.57–0.91)	0.77 (0.65–0.91)
Adverse events (*n* (%))					
Treatment-emergent adverse event	13 (44.8%)	13 (52.0%)	18 (69.2%)	33 (60.0%)	51 (63.0%)
Serious adverse event	1 (3.4%)	3 (12.0%)	8 (30.8%)	18 (32.7%)	26 (32.1%)
Worsening HF event	0 (0.0%)	2 (8.0%)	7 (26.9%)	12 (21.8%)	19 (23.5%)
Worsening HF event requiring hospitalization	0 (0.0%)	2 (8.0%)	2 (7.7%)	7 (12.7%)	9 (11.1%)
All-cause death	0 (0.0%)	1 (4.0%)	1 (3.8%)	3 (5.5%)	4 (4.9%)
Death or worsening HF event	0 (0.0%)	2 (8.0%)	7 (26.9%)	13 (23.6%)	20 (24.7%)
Hypotension	2 (6.9%)	7 (28.0%)	4 (15.4%)	7 (12.7%)	11 (13.6%)
Dyspnea	0 (0.0%)	0 (0.0%)	4 (15.4%)	4 (7.3%)	8 (9.9%)
Acute kidney injury	0 (0.0%)	0 (0.0%)	3 (11.5%)	1 (1.8%)	4 (4.9%)
Blood creatinine increased	1 (3.4%)	3 (12.0%)	1 (3.8%)	0 (0.0%)	1 (1.2%)
Discontinued owing to adverse event	0 (0.0%)	0 (0.0%)	1 (3.8%)	4 (7.3%)	5 (6.2%)
Discontinued owing to a serious adverse event	0 (0.0%)	0 (0.0%)	1 (3.8%)	1 (1.8%)	2 (2.5%)

**Table 3:** Biomarkers and adverse events over time within groups by background therapy

	Background ACE/ARB			Background sacubitril/valsartan		
Biomarker	Placebo	XXB750 60 mg	XXB750 120 mg	Placebo	XXB750 60 mg	XXB750 120 mg
	*n* = 9	*n* = 7	*n* = 15	*n* = 20	*n* = 19	*n* = 40
NT-proBNP (ng l^−1^) week 0	1,746 (1,076–2,833)	1,323 (800–2,189)	1,060 (579–1,940)	1,813 (1,035–3,176)	1,405 (890–2,219)	1,569 (1,252–1,965)
NT-proBNP (ng l^−1^) Week 4	1,611 (768–3,380)	821 (259–2,600)	1,195 (608–2,347)	1,838 (1,165–2,899)	1,576 (772–3,215)	2,571 (1,869–3,538)
NT-proBNP ratio change week 4	0.92 (0.66–1.30)	0.65 (0.19–2.25)	1.13 (0.74–1.72)	1.00 (0.79–1.27)	1.11 (0.69–1.76)	1.62 (1.28–2.04)
NT-proBNP (ng l^−1^) week 8	1,352 (772–2,367)	677 (46–9,965)	1,134 (607–2,121)	1,670 (966–2,888)	1,988 (1,023–3,866)	2,265 (1,526–3,362)
NT-proBNP ratio change week 8	0.77 (0.59–1.02)	0.46 (0.03–6.86)	1.23 (0.74–2.07)	0.91 (0.78–1.06)	1.51 (0.94–2.44)	1.48 (1.09–2.01)
NT-proBNP (ng l^−1^) week 16	1,503 (990–2,282)	783 (221–2,778)	1,289 (610–2,723)	1,592 (1,077–2,354)	1,442 (904–2,301)	2,396 (1,687–3,403)
NT-proBNP ratio change week 16	0.86 (0.67–1.11)	0.53 (0.16–1.80)	1.21 (0.75–1.95)	0.92 (0.63–1.35)	1.25 (0.91–1.72)	1.65 (1.16–2.33)
cGMP (nMol l^−1^) week 0	8.32 (6.33–10.95)	7.40 (4.18–13.09)	8.65 (5.98–12.49)	13.40 (11.33–15.86)	12.64 (9.24–17.28)	15.28 (12.85–18.18)
cGMP (nMol l^−1^) week 8	7.83 (5.47–11.21)	5.90 (4.24–8.21)	7.18 (5.72–9.02)	15.01 (12.34–18.25)	8.17 (6.30–10.60)	10.71 (9.08–12.63)
cGMP ratio change week 8	0.94 (0.75–1.18)	0.96 (0.49–1.89)	0.94 (0.70–1.27)	1.13 (1.00–1.28)	0.65 (0.50–0.83)	0.69 (0.59–0.82)
cGMP (nMol l^−1^) week 16	9.07 (6.40–12.85)	5.49 (3.84–7.84)	8.70 (6.19–12.22)	13.58 (11.34–16.27)	11.39 (8.46–15.34)	10.25 (7.26–14.47)
cGMP ratio change week 16	1.13 (0.88–1.44)	0.89 (0.55–1.43)	0.98 (0.72–1.32)	1.05 (0.93–1.17)	0.89 (0.71–1.12)	0.64 (0.47–0.86)
Adverse events						
Treatment-emergent adverse event	4 (44.4%)	5 (71.4%)	5 (33.3%)	9 (45.0%)	13 (68.4%)	28 (70.0%)
Serious adverse event	1 (11.1%)	1 (14.3%)	3 (20.0%)	0 (0.0%)	7 (36.8%)	15 (37.5%)
Worsening HF event	0 (0.0%)	1 (14.3%)	3 (20.0%)	0 (0.0%)	6 (31.6%)	9 (22.5%)
Worsening HF event requiring hospitalization	0 (0.0%)	0 (0.0%)	2 (13.3%)	0 (0.0%)	2 (10.5%)	5 (12.5%)
All-cause death	0 (0.0%)	0 (0.0%)	1 (6.7%)	0 (0.0%)	1 (5.3%)	2 (5.0%)
Death or worsening HF event	0 (0.0%)	1 (14.3%)	3 (20.0%)	0 (0.0%)	6 (31.6%)	10 (25.0%)
Hypotension	0 (0.0%)	0 (0.0%)	2 (13.3%)	2 (10.0%)	4 (21.1%)	5 (12.5%)
Dyspnea	0 (0.0%)	1 (14.3%)	0 (0.0%)	0 (0.0%)	3 (15.8%)	4 (10.0%)
Acute kidney injury	0 (0.0%)	0 (0.0%)	0 (0.0%)	0 (0.0%)	3 (15.8%)	1 (2.5%)
Blood creatinine increased	1 (11.1%)	0 (0.0%)	0 (0.0%)	0 (0.0%)	1 (5.3%)	0 (0.0%)
Discontinued owing to adverse event	0 (0.0%)	0 (0.0%)	0 (0.0%)	0 (0.0%)	1 (5.3%)	4 (10.0%)
Discontinued owing to a serious adverse event	0 (0.0%)	0 (0.0%)	0 (0.0%)	0 (0.0%)	1 (5.3%)	1 (2.5%)

### Safety

**Fig. 3:**
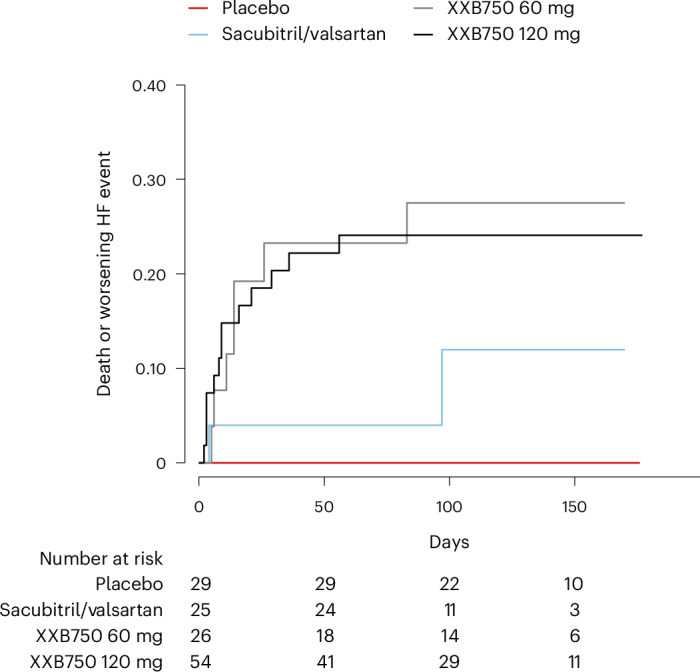
Death or worsening HF events among treatment groups. A Kaplan–Meier curve is shown comparing death or worsening HF events among the treatment groups. log-rank *P* = 0.0038, comparing pooled XXB750 arms to the placebo arm.

Overall, the total number of adverse events, serious adverse events or treatment-emergent adverse events was higher in those allocated to XXB750 compared with those allocated to sacubitril/valsartan or placebo (Table 2). There were four deaths (5%) in patients allocated to XXB750, one death (4%) in patients allocated to sacubitril/valsartan and zero deaths (0%) in patients allocated to placebo. Events believed by investigators to represent worsening HF occurred in 0 (0%) patients in the placebo group, 2 (8%) in the sacubitril/valsartan group, 7 (27%) in the 60 mg XXB750 group and 12 (22%) in the 120 mg XXB750 group (Fig. 3). HF leading to hospitalization occurred in zero (0%) patients in the placebo group, two (8%) patients in the sacubitril/valsartan group, two (8%) patients in the 60 mg XXB750 group and seven (13%) patients in the 120 mg XXB750 group. A composite outcome of all-cause death or a worsening HF event occurred in 0 (0%) patients in the placebo group, 2 (8%) patients in the sacubitril/valsartan group, 7 (27%) patients in the 60 mg XXB750 group and 13 (24%) patients in the 120 mg XXB750 group (and 25% in the pooled XXB groups). The number of worsening HF events was greater in patients assigned to XXB750 receiving background sacubitril/valsartan than in those on background angiotensin-converting enzyme inhibitor (ACEi) or angiotensin receptor blocker (ARB) (Table 3).

Dyspnea and hypervolemia adverse events were also more common in the XXB750 groups. Hypotension adverse events were more common in the XXB arm than in the placebo group but less frequent than among those randomized to sacubitril/valsartan. There were no classical, immune-mediated hypersensitivity reactions reported in the study. No adverse events related to severe tachycardia, severe acute bradycardia, serious injection site reactions, renal toxicity, hepatotoxicity or serious presyncope or syncope were reported during the study.

## Discussion

In this trial, we tested the hypothesis that the NPR1 receptor agonist XXB750 would, by activating particulate guanylyl cyclase and increasing intracellular cGMP, lead to arterial and venous dilatation in patients with HF. This, along with the potential lusotropic actions of natriuretic peptides, was expected to reduce NT-proBNP in patients with symptomatic HF. In contrast to the expected results, we observed a decrease in plasma cGMP and an increase in NT-proBNP, the magnitude of which was affected by XXB750 dose. These biomarker findings were associated with an excess of worsening HF events in patients receiving XXB750, leading to the early termination of the trial. The concordant biochemical and clinical findings appeared to be differentially influenced by whether or not patients were on background sacubitril/valsartan or ACEi or ARB, with those on background sacubitril/valsartan showing the greatest reduction in cGMP, the greatest rise in NT-proBNP, and the worst clinical outcomes. These findings have implications for further development of this and similar compounds for the treatment of HF.

Several natriuretic peptides, augmenting the particulate (membrane-bound) guanylate cyclase pathway, including ANP (carperitide) and BNP (nesiritide) and urodilatin (ularitide), administered as short-term intravenous infusions, have not been shown to reduce morbidity or mortality in phase III trials in acute HF^1,2,14^. Conversely, the oral neprilysin inhibitor sacubitril, which reduces natriuretic peptide breakdown, coupled with valsartan, improves outcomes in chronic HF and has a class I indication in all international guidelines^3^. The soluble (cytosolic) guanylate cyclase stimulator vericiguat has shown modest success in HF^15,16^, and additional approaches targeting this pathway, including the prevention of cGMP breakdown with PDE9 inhibition, are in development^17^.

However, the unexpected results of the present study suggest that among individuals with HF who have baseline activation of their natriuretic peptide systems with elevated levels of endogenous natriuretic peptides, XXB750 may behave not as an agonist but might paradoxically exert antagonist effects, possibly blunting the effects of endogenous natriuretic peptides. This interpretation is further supported by the observation that study participants allocated to XXB750—who were receiving background sacubitril/valsartan—appeared to have the greatest decrease in cGMP, the greatest increase in NT-proBNP and the worst outcomes, in contrast to minimal changes in the biomarkers, and have similar adverse events in study participants allocated to XXB750 who were on background ACEi or ARB. In other words, those with the greatest tonic elevation of natriuretic peptides appeared to exhibit the greatest paradoxical effects of XXB750. The elevation in endogenous natriuretic peptides, especially ANP, and subsequent stimulation of cGMP through the NPR1 receptor pathway is thought to play a key role in the observed benefit of sacubitril/valsartan, which inhibits the breakdown of the biologically active natriuretic peptides and other vasoactive proteins^4,18,19^. Although those patients randomized in this trial to switch from an ACEi or ARB to sacubitril/valsartan showed the expected increase in both plasma and urine cGMP (reflecting increased NPR1 signaling through augmented endogenous ANP and BNP levels) and reduction in NT-proBNP (reflecting ANP- and BNP-mediated vasodilatation, reduction in intracardiac pressures and reduced stimulus to myocardial natriuretic peptide secretion), this was in marked contrast to the findings in those allocated to XXB750. Contrary to our findings, a parallel study in patients with hypertension but without HF showed that XXB750 was associated with a dose-dependent increase in cGMP but with a neutral overall effect on ambulatory blood pressure, suggesting that in patients without HF, and thus without activation of their natriuretic peptide system, XXB750 exerts the expected target effect on guanylate cyclase^20^. Thus, this drug may behave as an agonist in normal volunteers and in patients with hypertension but as an antagonist in patients with HF.

The mechanisms underpinning these unexpected pharmacodynamic observations with XXB750 remain unclear. One potential hypothesis is that a monoclonal antibody, by producing a constant, sustained NPR1 activation owing to its long plasma half-life, might lead to downregulation or decreased responsiveness of the NPR1–particulate guanylyl cyclase–cGMP pathway. The desensitization of the NPR1 receptor, which is primarily mediated by receptor dephosphorylation^21^, can occur after prolonged or repeated exposure to high concentrations of natriuretic peptides^22^. Such downregulation has been observed after experimental exposure to high doses of ANP and BNP^23^. Whether such a response will occur with an engineered rather than endogenous ligand remains unclear. The observation that this does not occur during the augmentation of this pathway with an oral molecule such as sacubitril/valsartan, which likely results in less stimulation of the NPR1–particulate guanylyl cyclase–cGMP pathway than a direct agonist such as XXB750, might point to a potential benefit from activating this pathway with fluctuating pharmacokinetics, with peaks and trough plasma concentrations within the 24 h that may avoid the constant receptor activation exerted by a monoclonal antibody. Given the clearly unfavorable effects from the withdrawal of even the modest effects of neprilysin inhibition, future studies of NPR1 agonists should assess the optimal level of receptor activation necessary for therapeutic benefit and whether alternative kinetics of receptor activation may result in clinical benefits on top of neprilysin inhibition.

These findings need to be considered in light of their limitations. Because the trial was terminated early for safety concerns, it lacked sufficient power to adequately test the primary biomarker hypothesis, and the data presented need to be considered hypothesis-generating. In particular, this limits our ability to test for interactions on the basis of baseline therapy, and the finding that the adverse effects of XXB750 appear to be more pronounced in those patients on background sacubitril/valsartan needs to be viewed with caution. Nevertheless, the concordance of the biomarker and clinical findings has led to the discontinuation of the development of this specific antibody and suggests that vigilance is required in other development programs using similar agents. Nevertheless, there remains a strong rationale for developing effective and safe natriuretic peptide agonists and other approaches to augmenting the NPR1–particulate guanylyl cyclase–cGMP pathway in HF, including shorter-acting small molecules that bind to the NPR1 receptor with less affinity, and specific inhibitors of phosphodiesterase 9 responsible for the degradation of cGMP produced by particulate guanylyl cyclase^17^.

In conclusion, we found that the NPR1 receptor agonist monoclonal antibody XXB750 did not improve HF biomarkers in patients either on background ACEi or ARB therapy or sacubitril/valsartan and appeared to particularly worsen these biomarkers in participants on background sacubitril/valsartan therapy. XXB750-treated participants, especially those on background sacubitril/valsartan, experienced more serious and nonserious worsening HF adverse events, which led the data monitoring committee (DMC) to recommend early termination of the current study. These findings have implications for the development of natriuretic peptide therapeutics broadly beyond the current trial.

## Methods

### Trial design and oversight

We conducted an international, multicenter, parallel-group, randomized trial. The design enabled the evaluation of three doses of XXB750 compared with both placebo as well as open-label sacubitril/valsartan and accounted for background HF therapy (ACEi or ARB or sacubitril/valsartan). The steering committee designed and oversaw the conduct of the trial and data analysis, in collaboration with the sponsor (Novartis). The trial protocol (available with the full text of this article) was approved by institutional review boards or ethics committees at each trial center. Written informed consent was obtained from each patient. The authors who had access to the data (authors from Harvard Medical School and Novartis) vouch for the accuracy and completeness of the data, and all the authors vouch for the fidelity of the trial to the protocol. A list of study personnel, the trial protocol and statistical analysis plan are provided in Supplementary Information.

### Trial participants

Eligible participants were men and women aged ≥18 years with symptomatic HF (New York Heart Association class II–III) and LVEF <50% documented within 6 months before or during screening. Participants were required to have an NT-proBNP ≥600 ng l^−1^ in sinus rhythm or ≥900 ng l^−1^ in atrial fibrillation or flutter and to be receiving stable therapy for ≥4 weeks with either an ACEi (greater than or equal to enalapril 10 mg daily or equivalent), an ARB (at specific doses equivalent to enalapril 10 mg daily) or sacubitril/valsartan (≥49/51 mg twice daily), as well as other guideline-directed therapies as appropriate.

Key exclusion criteria included acute decompensated HF or hospitalization for HF within 3 months, systolic blood pressure ≥180 mm Hg or <105 mm Hg, prior intolerance to sacubitril/valsartan or any ARB, serum potassium >5.4 mmol l^−1^, eGFR <30 ml min^−1 ^1.73 m^−2^ or a history of angioedema.

### Trial procedures

Individuals who met eligibility criteria who were on background ACEi or ARB were randomly allocated to receive blinded subcutaneous XXB750 60-mg doses, XXB750 titrated to a maximum dose of 120 mg, matching subcutaneous placebo or open-label oral sacubitril/valsartan. Those who were on background sacubitril/valsartan were randomly allocated to either blinded XXB750 60-mg doses, XXB750 titrated to a maximum dose of 120 mg or matching placebo (Fig. 1). Randomization was stratified by region and ACEi or ARB versus sacubitril/valsartan background therapy to ensure that the XXB750 or placebo arms had approximately two thirds of patients on background sacubitril/valsartan therapy. Participants randomized to XXB750, or its matching placebo, received one subcutaneous injection every 4 weeks, with a total of four injections during the 16-week treatment period, whereas participants randomized to open-label sacubitril/valsartan were titrated, where possible, to a target dose of 97/103 mg twice daily for 16 weeks. Once the participants entered the trial, every effort was made to avoid modifications of any background HF therapy components, unless essential. All study treatments were discontinued at week 16, after which participants entered the safety follow-up period and were managed at the discretion of the investigator.

The original plan was to randomize 720 participants and include an XXB750 group with a target dose of 240 mg in an adaptive design. A staggered enrollment was planned, initially excluding the planned highest 240-mg target dose of XXB750 until the safety of the 60 mg and 120 mg target dose results were confirmed by the DMC. However, enrollment was stopped early after 135 participants had received randomly allocated treatment owing to an excess of adverse events in the XXB750 groups, and no patients were randomized to the planned 240-mg group.

### Outcomes

The primary objective of the study was to evaluate the efficacy and dose–response relationship of the three XXB750 target dose levels compared with placebo in reducing NT-proBNP, reflecting a reduction in left ventricular wall stress from baseline to week 16. Secondary objectives included evaluating the effect on NT-proBNP from adding the two highest doses of XXB750 to background ACEi or ARB treatment, compared with switching to sacubitril/valsartan. Safety assessments included adverse events, serious adverse events and adverse events of special interest, including serious hypotension, presyncope or syncope, hypersensitivity reactions, injection site reactions and severe tachycardia or bradycardia.

### Data safety monitoring board and study termination

An external DMC independent of the sponsor was appointed to monitor the study conduct, review safety regularly and determine if it was safe to continue the study according to the protocol. During a regular meeting on 6 August 2024, the DMC reviewed cumulative safety data from the study, identified a marked increase in the frequency of worsening HF events in participants receiving XXB750 versus those on placebo or on sacubitril/valsartan and recommended the termination of the trial. All participants who had been randomized to receive XXB750 or placebo were followed up for 12 weeks after they received the last dose, which was in accordance with the originally planned follow-up duration as per protocol.

### Statistical analysis

It was originally planned to randomly allocate a total of 720 participants in the ratio of 2:2:3:3:2 to placebo, 60 mg of XXB750, 120 mg of XXB750, 240 mg of XXB750 or sacubitril/valsartan, respectively. Assuming a common standard deviation of 0.67 for log NT-proBNP change from baseline and a two-sided 5% significance level, a sample size of 600 participants (120 allocated to placebo, 120 to 60 mg of XXB750, 180 to 120 mg of XXB750 and 180 to 240 mg of XXB750) would have provided a power of 90% if the underlying true maximum NT-proBNP reduction with XXB750 versus placebo was 23%.

Owing to the early study termination, the primary estimand and statistical modeling for the dose–response relationship were no longer applicable. The primary endpoint of change in NT-proBNP was summarized descriptively as geometric means and 95% CI for each treatment group administered in the trial and by pooling the two XXB750 groups. We provided exploratory least-square mean difference in change versus placebo at 16 weeks in sensitivity analyses using analysis of covariance with log-transformed baseline biomarker values and treatment terms as covariates. Separate assessments were made on the basis of the background treatment group (ACEi or ARB versus sacubitril/valsartan).

### Reporting summary

Further information on research design is available in the Nature Portfolio Reporting Summary linked to this article.

## Online content

Any methods, additional references, Nature Portfolio reporting summaries, source data, extended data, supplementary information, acknowledgements, peer review information; details of author contributions and competing interests; and statements of data and code availability are available at 10.1038/s41591-026-04313-w.

## Supplementary information

Supplementary Information Study personnel and committees, final protocol and SAP.

Reporting summary

## Figures and Tables

**Extended Data Table 1:**
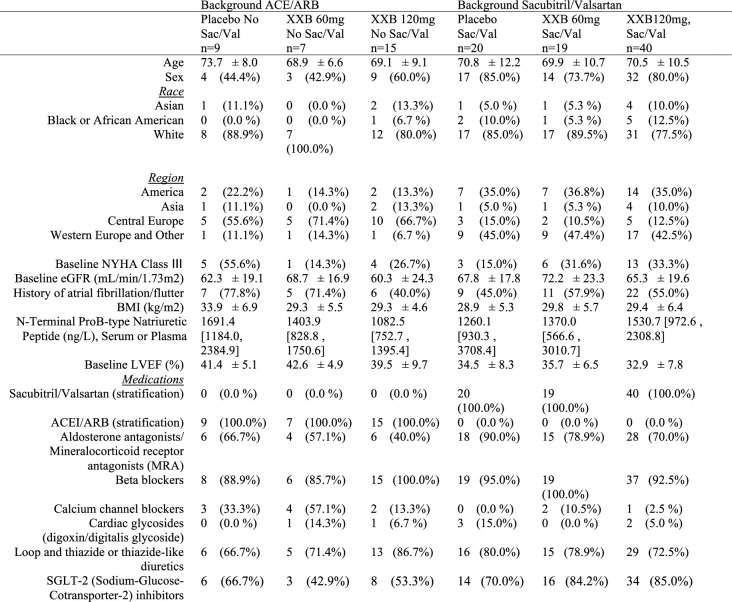
Baseline Characteristics by background therapy

**Extended Data Table 2:**
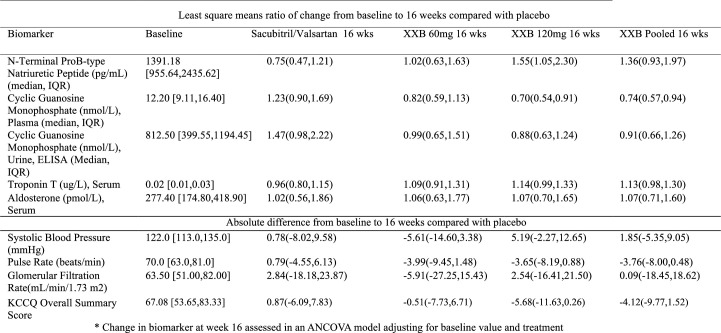
Least-Square Mean Differences* in Biomarkers Compared with Placebo at 16 weeks

## Data Availability

The data from this clinical trial are not publicly available. Novartis is committed to sharing access to patient-level data and supporting clinical documents with qualified external researchers. These requests are reviewed on an ongoing basis and approved expeditiously on the basis of scientific merit based on the policies described at the website below. All data provided are anonymized to respect the privacy of patients who have participated in the trial, in line with applicable laws and regulations. The data can be requested from https://www.novctrd.com/.
