# Supplementary material for: The NPR1 agonist antibody XXB750 in heart failure: a phase 2 randomized trial
Source: Nat Med. 2026 Mar 30;32(5):1694–700. doi: 10.1038/s41591-026-04313-w (PMC13190253; doi:10.1038/s41591-026-04313-w)
Supplement: Supplementary file 1 — Study personnel and committees, final protocol and SAP. [file 41591_2026_4313_MOESM1_ESM.pdf]

nature medicine

Supplementary information https://doi.org/10.1038/s41591-026-04313-w

The NPR1 agonist antibody XXB750 in heart failure: a phase 2 randomized trial

In the format provided by the authors and unedited

The NPR1 agonist XXB750 in Heart Failure

Supplementary Appendix

Table of Contents

Study Personnel **2**
List of Investigators by Country 2
Steering Committee 4
Independent Statistician 4
Data Monitoring Committee 4

Study Protocol and Statistical Analysis Plan **5**

Study Personnel

List of Investigators by Country

Bulgaria

Atanas Atanasov, UMHAT Sveta Marina
Adriana Dincheva, Medical Center Dea Medicals EOOD
Yordan Dzhumean, Cardiology department, MHAT Puls
Iskren Garvanski, Medical Center Hera EOOD
Valentina Mincheva Kabakchieva, Clinic of Cardiology, NMTranspHospital Tsar Boris Treti
Fedya Nikolov, Clinic of Cardiology, UMHAT Sveti Georgi EAD
Miroslav Stoyanov, Cardiology cabinet, MC New Polyclinic Gabrovo Ltd
Maria Tzekova, MC Medconsult Pleven OOD

China

Zuyi Yuan, The 1st Affiliated Hospital of Xi'an Jiaotong University

Germany

Anselm Baeumer, Praxis für Innere Medizin Kardiologie Pneumologie
Derk Frank, Klinik für Innere Medizin III, Universitätsklinikum Kiel
Markus Knapp, Kardiologische Praxis Dr. Knapp
Daniel Schick, Hausarztzentrum Butendorf
Nicole Toursarkissian, Praxis Dr. med. Nicole Toursarkissian
Peter Uebel, Haus Der Gesundheit
Andreas Wilke, Kardiologische Praxis Dr. Wilke

Hungary

Akos Kalina, Magyar Honvedseg Egeszsegugyi kozpont
Laszlo Koranyi, DRC Gyogyszervizsgalo Kozpont Kft

India

Pinto Nahata, Department of Cardiology, S P Medical college and A G Hosp
Prasad Renuka, Kles Dr Prabhakar Kore Hospital and Medical Research Centre

Italy

Pier Giuseppe Agostoni, Centro Cardiologico Monzino IRCCS
Nadia Aspromonte, UOSD Scompenso Cardiaco, IRCCS Policlinico Universitario Fondazione Agostino Gemelli
Egidio Imbalzano, UOC Medicina Interna, AOU Policlinico Gaetano Martino
Marco Merlo, Polo Cardiologica SC Cardiologia, Ospedale Di Cattinara
Marco Metra, Unita Operativa di Cardiologia, ASST Spedali Civili Di Brescia

Marco Mojoli, SC Cardiologia, Azienda Ospedaliera Santa Maria Degli Angeli
Riccardo Morgagni, UOC Cardiologia, Policlinico Tor Vergata

Japan

Junya Ako, Cardio-angiology, Kitasato University Hospital
Keisuke Kida, Cardiovascular Medicine, St Marianna University Hospital
Koichiro Kuwahara, Cardiology, Shinshu University Hospital
Noriaki Takama, Cardiovascular Medicine, Gunma University Hospital

Portugal

Ricardo Carvalho, Cardiologia, Unidade Local De Saude Gaia Espinho
Candida Fonseca, Servico de Medicina Interna, Hospital Sao Francisco Xavier

Slovakia (Slovak Republic)

Andrej Dzupina, Kardiologicka ambulancia, Alian s r o Poliklinika CK Plus
Peter Fulop, Interna SK sro Kardiologicka a interna ambulancia
Livia Jamriskova, Kardiologicka ambulancia
Ivan Majercak, Medical group Kosice s r o
Ladislav Sabo, INTERSTOM s r o Ambulancia vnutorneho lekarstva
Daniela Vinanska, Medispol s r o

Spain

Javier Salvador de Juan Baguda, Servicio De Cardiologia, Hospital Universitario 12 de Octubre
Luis Almenar Bonet, Servicio Cardiologia, Hospital Universitari i Politècnic La Fe
Jose Ramon Gonzalez Juanatey, Servicio de Cardiologia, Hospital Clinico Universitario De Santiago
Marta Cobo Marcos, Servicio de Cardiologia, Hospital Universitario Puerta de Hierro
Antonio Enrique Gomez Menchero, Servicio de Cardiologia, Hospital Juan Ramon Jimenez
Jose Manuel Garcia Pinilla, Servicio de Cardiologia, Hospital Universitario Virgen De La Victoria
Antonio Garcia Quintana, Servicio de Cardiologia, Hospital Universitario de Gran Canaria Doctor Negrín
Julio Nunez Villota, Servicio de Cardiologia, Hospital Clinico Universitario de Valencia

Taiwan

Kuan Cheng Chang, China Medical University Hospital

United States

Rafik Abadier, Nature Coast Clinical Research LLC
Felton Anderson, Anderson Medical Research
Justin Arambasick, The Research Group
Nonso Ezema, NexGen Research
Jean Foucauld, Cardiology Partners Clinical Research Institute

Robert I Garver, SEC Clinical Research
Dinesh Gupta, Tennessee Center for Clinical Trials
Alexis Gutierrez, Inpatient Research Clinical LLC
Richard Jackson, Dominion Medical Associates
Kaushik Jain, Tennessee Center for Clinical Trials
Ghiath Mikdadi, Heart Clinic of Hammond
Chinedu Nweke, American Clinical Trials
Qaiser Shafiq, Revival Research Institute
Ataul Qureshi, Aultman Hospital

Steering Committee

Scott D. Solomon, MD (Co-Chair), Brigham and Women's Hospital; John J.V. McMurray, MD (Co-Chair), University of Glasgow; G. Michael Felker, MD, Duke University; James L. Januzzi, MD, Massachusetts General Hospital; Carolyn S.P. Lam, MBBS PhD, Duke National University of Singapore; Adriaan A. Voors, MD, PhD, University of Groningen

Independent Statistician

Brian Claggett, PhD, Brigham and Women's Hospital

Data Monitoring Committee

Jean Lucien Rouleau, MD (Chair), Montreal Heart Institute; Marc Pfeffer, MD, PhD, Brigham and Women's Hospital; Raymond Townsend, MD, FAHA, FASH, University of Pennsylvania Medical Center; Thomas MacDonald, MD, MB CHB, FRCP, University of Dundee School of Medicine; Stuart Pocock, PhD, London School of Hygiene & Tropical Medicine

Study Protocol and Statistical Analysis Plan

This supplement contains the following items:

1. Original protocol, final protocol, summary of changes
2. Original statistical analysis plan, final statistical analysis plan, summary of changes

NOVARTIS

Novartis Research and Development

Clinical Trial Protocol Title:

A multi-center, randomized, placebo- and active-controlled, parallel-group, 24-week proof of concept and dose-finding study to evaluate efficacy, safety, and tolerability of XXB750 in patients with heart failure

Clinical Trial Protocol Number: CXXB750A12201 / NCT06142383

Version Number: 02 (Amended Protocol) (Clean)

Compound: XXB750

Brief Title: A proof of concept and dose-finding study of XXB750 in patients with heart failure

Study Phase: II

Sponsor Name: Novartis

Regulatory Agency Identifier Number(s): EU-CT Number 2023-504678-39-00

Approval Date: 23-Feb-2024

Property of Novartis
Confidential
May not be used, divulged, published, or otherwise disclosed
without the consent of Novartis

Clinical Trial Protocol Template Version 5.0 dated 14-Jan-2022

Table of contents

Table of contents 2
List of tables 6
List of figures 7
Amendment 02 (23-Feb-2024) 8
Amendment 01 (06-Feb-2024) 9
1 Protocol summary 11
1.1 Summary 11
1.2 Schema 15
1.3 Schedule of activities (SoA) 15
2 Introduction 19
2.1 Study rationale 19
2.2 Background 19
2.3 Benefit/Risk assessment 21
3 Objectives, endpoints, and estimands 23
3.1 Primary estimands 24
3.2 Secondary estimands 25
4 Study design 26
4.1 Overall design 26
4.1.1 Screening Period 26
4.1.2 Randomized Treatment Period 27
4.1.3 Safety Follow-up Period 28
4.2 Scientific rationale for study design 29
4.2.1 Rationale for target study population 30
4.2.2 Rationale for the primary endpoint 31
4.3 Justification for dose 31
4.3.1 Rationale for choice of background therapy 32
4.4 Rationale for choice of control drugs (comparator/placebo) or combination drugs 32
4.5 Rationale for public health emergency mitigation procedures 33
4.6 Purpose and timing of interim analyses/design adaptations 33
4.7 End of study definition 35
5 Study population 35
5.1 Inclusion criteria 36
5.2 Exclusion criteria 36
5.3 Screen failures 38
5.3.1 Replacement policy 39

5.3.2 Participant numbering 39
6 Study treatment(s) and concomitant therapy 39
6.1 Study treatment(s) 39
6.1.1 Additional study treatments 40
6.1.2 Treatment arms/group 40
6.1.3 Treatment duration 41
6.2 Preparation, handling, storage, and accountability 41
6.2.1 Handling of study treatment 42
6.2.2 Handling of other treatment 43
6.2.3 Instruction for prescribing and taking study treatment 43
6.3 Measures to minimize bias: randomization and blinding 44
6.3.1 Treatment assignment, randomization 44
6.3.2 Treatment blinding 44
6.3.3 Emergency breaking of assigned treatment code 46
6.4 Study treatment compliance 47
6.4.1 Recommended treatment of adverse events 47
6.5 Dose modification 47
6.5.1 Definitions of dose limiting toxicities (DLTs) 48
6.5.2 Follow-up for toxicities 49
6.6 Continued access to study treatment after the end of the study 50
6.6.1 Post trial access 50
6.7 Treatment of overdose 50
6.7.1 Reporting of study treatment errors including misuse/abuse 51
6.8 Concomitant and other therapy 51
6.8.1 Concomitant therapy 51
6.8.2 Prohibited medication 52
6.8.3 Rescue medicine 53
7 Discontinuation of study treatment and participant discontinuation/withdrawal 53
7.1 Discontinuation of study treatment 53
7.2 Participant discontinuation from the study 54
7.3 Withdrawal of informed consent and exercise of participants’ data privacy rights 54
7.4 Lost to follow-up 55
7.5 Early study termination by the Sponsor 55
8 Study Assessments and Procedures 55
8.1 Screening 55

8.2 Participant demographics/other baseline characteristics 56
8.3 Efficacy assessments 56
8.3.1 NT-proBNP 56
8.3.2 Secondary efficacy assessments 57
8.3.3 Appropriateness of efficacy assessments 57
8.4 Safety assessments 57
8.4.1 Physical examinations 57
8.4.2 Vital signs 57
8.4.3 Height and weight 58
8.4.4 Electrocardiograms 58
8.4.5 Clinical safety laboratory tests 58
8.4.6 Pregnancy testing 59
8.4.7 Other safety evaluations 60
8.4.8 Appropriateness of safety measurements 61
8.5 Additional assessments 61
8.5.1 Clinical Outcome Assessments (COAs) 61
8.5.2 Other assessments 62
8.6 Adverse events (AEs), serious adverse events (SAEs), and other safety reporting 63
8.6.1 Adverse events 63
8.6.2 Serious adverse events 66
8.6.3 SAE reporting 67
8.6.4 Pregnancy 69
8.6.5 Disease-related events and/or disease-related outcomes not qualifying as AEs or SAEs 69
8.6.6 Adverse events of special interest 70
8.7 Pharmacokinetics 70
8.8 Biomarkers 70
8.9 Immunogenicity assessments 72
8.10 Medical resource utilization and health economics 72
9 Statistical considerations 72
9.1 Analysis sets 72
9.2 Statistical analyses 73
9.2.1 General considerations 73
9.2.2 Participant demographics and other baseline characteristics 73
9.2.3 Treatments 74
9.3 Primary endpoint(s)/estimand(s) analysis 74

9.3.1 Definition of primary endpoint(s) 74
9.3.2 Statistical model, hypothesis, and method of analysis 74
9.3.3 Handling of intercurrent events of primary estimand (if applicable)....76
9.3.4 Handling of missing values not related to intercurrent event 77
9.3.5 Multiplicity adjustment (if applicable)....77
9.3.6 Sensitivity analyses 77
9.3.7 Supplementary analysis....77
9.4 Secondary endpoint(s)/estimand(s) analysis....78
9.4.1 Efficacy and/or pharmacodynamic endpoint(s) 78
9.4.2 Safety endpoints 79
9.5 Exploratory endpoint(s)/estimand(s) analysis 80
9.5.1 Exploratory variables 81
9.5.2 Analysis methods 81
9.5.3 Pharmacokinetics 83
9.5.4 Biomarkers 84
9.5.5 Immunogenicity 84
9.5.6 Patient reported outcomes 84
9.6 Other safety analyses 84
9.7 Other analyses....84
9.7.1 Subgroup analyses....84
9.7.2 DNA 85
9.8 Interim analysis....85
9.9 Sample size determination....85
9.9.1 Primary endpoint(s)....86
9.9.2 Secondary endpoint(s)....86
10 Supporting documentation and operational considerations....86
10.1 Appendix 1: Regulatory, ethical, and study oversight considerations....86
10.1.1 Regulatory and ethical considerations 86
10.1.2 Informed consent process....88
10.1.3 Data protection 89
10.1.4 Committees structure 90
10.1.5 Data quality assurance....90
10.1.6 Source documents 92
10.1.7 Publication policy....92
10.1.8 Protocol adherence and protocol amendments....93
10.2 Appendix 2: Abbreviations and definitions....94

10.2.1 List of abbreviations 94
10.2.2 Definitions 97
10.3 Appendix 3: Clinical laboratory tests 100
10.3.1 Clinically notable laboratory values and vital signs 100
10.4 Appendix 4: Participant Engagement 102
10.5 Appendix 5: Liver safety monitoring 103
10.5.1 Liver event and laboratory trigger definitions & follow-up requirements 103
10.6 Appendix 6: Renal safety monitoring 106
10.6.1 Specific Renal Alert Criteria and Actions and Event Follow-up 106
10.7 Appendix 7: Managing hypotension 107
10.8 Appendix 8: Managing hyperkalemia 109
11 References 111

List of tables

Table 1-1 Assessment Schedule 17
Table 3-1 Objectives and related endpoints 23
Table 4-1 Minimum required pre-study daily doses of ACEIs and ARBs 27
Table 4-2 Overview of the planned staggered enrollment strategy 34
Table 6-1 Investigational and control drug 39
Table 6-2 Auxiliary treatments 40
Table 6-3 Treatment arms, dose levels, and planned doses administered at dispensing visits during the Randomized treatment period 41
Table 6-4 Blinding and unblinding plan 45
Table 6-5 Diagnostic assessments to determine alternative causes of observed LFT abnormalities 49
Table 8-1 Clinical Safety Laboratory Tests 59
Table 8-2 MedDRA PT related to the underlying heart failure 68
Table 9-1 Selecting a winner based on combination of change of KCCQ CSS and change of NYHA class 82
Table 10-1 Clinically notable hematology abnormalities 100
Table 10-2 Clinically notable blood chemistry abnormalities 100
Table 10-3 Liver event and laboratory trigger definitions 103
Table 10-4 Follow up requirements for liver laboratory triggers - ALT, AST, TBL 104
Table 10-5 Follow-up requirements for liver laboratory triggers based on isolated elevations in TBL 105

Table 10-6 Renal event criteria and corresponding actions to be taken upon their occurrence 106

List of figures

Figure 1-1 Study design 15
Figure 6-1 Modification of injectable study medication 48
Figure 9-1 Dose-response curve of candidate models 75
Figure 10-1 Flow chart for managing hypotension 108

Amendment 02 (23-Feb-2024)

Amendment rationale

The purpose of this amendment is to make minor editorial modifications to the protocol.

Changes to the protocol

Changes to specific sections of the protocol are shown in the track changes version of the protocol using strikethrough red font for deletions and red underline for insertions.

List of changes to the protocol are listed below:

Section 3.2 **Secondary estimands**

- The formatting of the paragraph describing the treatment of interest of the second secondary estimand was corrected to match the formatting of the original protocol.

Section 5.2 **Exclusion criteria**

- The formatting for Exclusion criterion #26 was corrected to clarify that each subpoint is a component of the main criterion rather than a stand-alone one.

IRB/IEC

A copy of this amended protocol will be sent to the Institutional Review Boards (IRBs)/Independent Ethics Committees (IECs) and Health Authorities.

The changes described in this amended protocol require IRB/IEC approval prior to implementation.

Amendment 01 (06-Feb-2024)

Amendment rationale

The purpose of this amendment is to make modifications to the protocol that were requested by the EU Health Authorities.

In addition, minor editorial changes are made to correct typographical errors, to make some clarifications, and to resolve some minor inconsistencies in various sections of the protocol.

Changes to the protocol

Changes to specific sections of the protocol are shown in the track changes version of the protocol using strikethrough red font for deletions and red underline for insertions.

List of changes to the protocol are listed below:

Section 1.1 Summary

- A footnote was added to table 'Objectives, Endpoints, and Estimands' to clarify that if the treatment arms 4a and 4b are eliminated (see Section 4.6), the second secondary objective will evaluate only the 120 mg XXB750 arm to sacubitril/valsartan.
- The inclusion criterion was modified to require that other evidence-based therapy for heart failure should also be considered e.g., cardiac resynchronization therapy and implantable cardioverter-defibrillator in selected patients, as recommended by guidelines in selecting patients for the study.
- The exclusion criterion was updated to exclude patients with previous intolerability to any ARB (as per the investigator's judgement).

Section 3 Objectives, endpoints and estimands

- Table 3-1 was updated to include a footnote clarifying that in the event treatment arms 4a and 4b are eliminated (see Section 4.6), the second secondary objective will evaluate only the 120 mg XXB750 arm to sacubitril/valsartan.

Section 3.2 Secondary estimands

- This section was updated to reflect the estimand of the second secondary endpoint in the event treatment arms 4a and 4b are eliminated.

Section 4.1.2 Randomized Treatment Period

- This section was modified to clarify the distribution of participants across the different treatment arms according to their background anti-RAS treatment (i.e., ACEI/ARB vs. ARNI).

Section 4.6 Purpose and timing of interim analyses/design adaptations

- This section was modified to clarify that in case of elimination of treatment arms 4a and 4b, 180 participants will be allocated to treatment arm 3 from Group 2 for a total/overall sample size of 300 participants in this arm.
- A statement was added to confirm that the sponsor will inform the local health authorities as per the local regulations in case of any adaptations to study design based on feedback from the DMC.

Section 5.1 Inclusion criteria

- Inclusion criterion #7 was modified to require that other evidence-based therapy for heart failure should also be considered, e.g., cardiac resynchronization therapy and implantable cardioverter-defibrillator in selected patients, as recommended by guidelines in selecting patients for the study. Also, it was clarified that guideline recommended HF medications should be stable in dose for at least 4 weeks before screening.

Section 5.2 Exclusion criteria

- Exclusion criterion #6 was updated to exclude patients with previous intolerability to any ARB (as per the investigator's judgment). Exclusion criterion #13 was updated to exclude patients with known right ventricular dysfunction that is deemed to increase susceptibility to the hypotensive effects of therapy in the opinion of the investigator. Exclusion criterion #15 was updated to exclude patients with CRT implantation within 3 months of screening. Exclusion criterion #18 was updated to exclude patients with planned/anticipated major cardiac surgery during the involvement of the participant in the study.

Section 6.1.1 Additional study treatments

- Table 6-2 Auxillary treatments was updated to delete the statement "Each packaging will be labeled as per country requirements" since none of the participating countries have such requirements.

Section 9.4.1 Efficacy and/or pharmacodynamic endpoint(s)

- This section was updated to note that in the event treatment arms 4a and 4b are eliminated, the comparison made in the second secondary endpoint will be between only the 120 mg XXB750 and the sacubitril/valsartan arms.

Section 9.9.1 Primary endpoint(s)

- This section was updated to note the sample size and statistical power for evaluating the primary endpoint in the event treatment arms 4a and 4b are eliminated.

Section 9.9.2 Secondary endpoint(s)

- This section was updated to note the sample size and statistical power for evaluating the first and second secondary endpoints in the event treatment arms 4a and 4b are eliminated.

Miscellaneous corrections of typographical errors were made throughout the protocol.

IRB/IEC

A copy of this amended protocol will be sent to the Institutional Review Boards (IRBs)/Independent Ethics Committees (IECs) and Health Authorities.

The changes described in this amended protocol require IRB/IEC approval prior to implementation.

1 Protocol summary

1.1 Summary

Protocol Title:

A multicenter, randomized, placebo- and active-controlled, parallel-group, 24-week proof-of-concept and dose-finding study to evaluate efficacy, safety, and tolerability of XXB750 in patients with heart failure

Brief Title:

A proof-of-concept and dose-finding study of XXB750 in patients with heart failure

Purpose:

The purpose of this phase 2 study is to investigate the efficacy, safety, and tolerability of different doses of XXB750 given in different dosing regimens compared to placebo in heart failure (HF) patients with New York Heart Association classification II-III (NYHA class II-III) with left ventricular ejection fraction (LVEF) < 50% receiving standard of care (SoC) therapy. This study will also compare different dose levels of XXB750 versus a conversion to sacubitril/valsartan for the same outcomes. The results of this study will provide information needed for designing the XXB750 phase 3 HF program.

Study Indication /Medical Condition:

Heart failure

Treatment type:

Biological

Study type:

Interventional

Objectives, Endpoints, and Estimands:

Objectives	Endpoints
**Primary**	
• To evaluate the efficacy and dose-response relationship of three XXB750 target dose levels compared to placebo in reducing N-terminal prohormone B-type natriuretic peptide (NT-proBNP) from baseline to Week 16 in symptomatic HF patients with LVEF < 50% treated with standard of care, including ACEI/ARB or sacubitril/valsartan.<br>The primary clinical question of interest is: Is there a dose-response signal, and if so, to characterize the dose-response relationship of three XXB750 target dose levels versus placebo in reducing NT-proBNP from baseline to Week 16 in participants with heart	• Change in log NT-proBNP from baseline to Week 16

failure (NYHA classes II-III) with LVEF <50% regardless of type of baseline background HF therapy, discontinuation from study treatment, change in the dose of concomitant medications and use of prohibited concomitant medication.	
**Secondary**	
• To evaluate the treatment effect of the highest XXB750 target dose level compared to placebo in reducing NT-proBNP from baseline to Week 16 in symptomatic HF patients with LVEF < 50% treated with standard of care, including ACEI/ARB or sacubitril/valsartan.<br>The estimand of this first secondary objective will further demonstrate the efficacy of highest XXB750 target dose level compared with placebo, regardless of type of baseline background HF therapy, discontinuation from study treatment, change in the dose of background HF medications and use of prohibited concomitant medication; it will provide an estimate with a p-value in a hierarchical hypothesis testing procedure with overall type I error control after a dose-response signal is established in the primary objective.<br>• To evaluate the treatment effect of combined two highest XXB750 target dose levels administered in addition to a background of ACEI/ARB versus conversion from ACEI/ARB to sacubitril/valsartan, in reducing NT-proBNP from baseline to Week 16.*<br>• To evaluate the safety and tolerability of XXB750 up-titration regimens and dose levels.	• Change in log NT-proBNP from baseline to Week 16<br>• Adverse events, safety laboratory parameters, and vital signs from baseline to end of study (EOS)

*In the event treatment arms 4a and 4b are eliminated (see Section 4.6), the second secondary objective will evaluate only the 120 mg XXB750 arm to sacubitril/valsartan arm.

Trial Design:

Study CXXB750A12201 is a multicenter, randomized, placebo- and active-controlled, parallel-group study which is comprised of three periods:

- A 7-days screening period
- A 16-week parallel-group randomized treatment period, on either
  i) double-blind placebo-controlled treatment (XXB750 vs. placebo) or
  ii) open-label treatment (sacubitril/valsartan)
- An 8-week safety follow-up period

The study will enroll participants with HF (NYHA class II-III), elevated NT-proBNP levels and LVEF < 50% receiving SoC HF therapy.

Brief Summary:

The purpose of this study is to assess the efficacy and safety of different target doses of XXB750 given in different up-titration regimens on NT-proBNP change from baseline at week 16 compared to placebo and to sacubitril/valsartan in participants with symptomatic HF with LVEF < 50%.

XXB750 is a fully human monoclonal antibody with potent agonistic activity at the natriuretic peptide receptor 1 (NPR1). NPR1 catalyzes cyclic guanosine monophosphate (cGMP) synthesis, thereby enhancing natriuresis, diuresis and vasodilation as well as protecting from cardiac fibrosis. Consistent with its mechanism of action, XXB750 was shown to decrease cardiac hypertrophy and NT-proBNP levels in mice lacking atrial natriuretic peptide.

Patients with symptomatic HF (NYHA class II-III), elevated NT-proBNP levels and a LVEF < 50% despite receiving an ACEI, an ARB, or an angiotensin receptor neprilysin inhibitor (ARNI), and other guideline-recommended therapies for HFrEF and HFmrEF, will be recruited into the study. Participants must be on ACEI/ARB doses equivalent to enalapril 10 mg/day or higher (see Table 4-1) or sacubitril/valsartan doses of 49/51 mg twice daily (bid) or higher. Participants who successfully complete the Screening period are planned to be randomized to one of three target dose levels of XXB750 (60 mg, 120 mg or 240 mg) or matching placebo. In addition, a cohort of patients receiving an ACEI/ARB will be randomly selected to be converted to receive open-label sacubitril/valsartan in the randomized treatment period, replacing their pre-study ACEI/ARB. Participants who receive an ACEI and are randomized to receive open-label sacubitril/valsartan must undergo a 36-hr ACEI-free wash-out period before starting to take the randomized treatment (open-label sacubitril/valsartan) to minimize the risk of occurrence of angioedema. All patients will continue to receive their other background HF medication. Overall, a total of approximately 720 participants will be randomized into this study.

The randomized treatment period will last 16 weeks. Participants randomized to XXB750 or its matching placebo will receive 1 subcutaneous injection every 4 weeks, with a total of 4 injections during the 16-week treatment period. Participants randomized to conversion to sacubitril/valsartan will receive sacubitril/valsartan for 16 weeks. At the end of the 16-week randomized treatment period, all assigned study treatments will be discontinued and the participants will enter an 8-week safety follow-up period during which they will be treated according to the investigator's clinical judgment. Assessment of NT-proBNP will occur at baseline, week 4, week 8, week 12, week 16 (timepoint of primary endpoint), week 20 and week 24. Safety, efficacy and/or pharmacokinetic assessments will be performed serially throughout all three study periods.

Study Duration:

24 weeks

Treatment Duration:

16 weeks

Visit Frequency:

Every 1-4 weeks

Treatment of Interest

XXB750 target dose levels of 60 mg, 120 mg, and 240 mg or matching placebo or sacubitril/valsartan. Adaptive design features of the protocol may impact the planned target dose levels (Section 4.6).

Treatment Groups

Participants will be randomized to be treated during a 16-week randomized treatment period with:

- Placebo matching XXB750
- XXB750 60 mg target dose
- XXB750 120 mg target dose
- XXB750 240 mg target dose in one of two up-titration regimens
- Sacubitril/valsartan at target doses of 97/103 mg bid (only participants previously treated with ACEI/ARB)

Treatment groups may be modified per adaptive design feature of the study design (Section 4.6).

Number of Participants:

720 participants female or male participants aged 18 years or older with symptomatic HF and LVEF < 50% will be randomized to study intervention. Participants with LVEF > 40% will be limited to approximately 25% of the total randomized participants.

Key Inclusion Criteria

- Written informed consent must be obtained before any assessment is performed.
- Male and female outpatients who are ≥ 18 years old.
- Symptom(s) of HF NYHA class II-III at Screening.
- LVEF < 50% (most recent local measurement, made within 6 months prior to or during Screening using echocardiography, MUGA, CT scanning, MRI or ventricular angiography is acceptable, provided no subsequent measurement ≥ 50%).
- NT-proBNP ≥ 600 pg/ml if in sinus rhythm or
  NT-proBNP ≥ 900 pg/ml if in atrial fibrillation/flutter at Screening.
- Receiving an ACEI or an ARB at a stable dose of at least enalapril 10 mg/d or equivalent for at least 4 weeks before Screening or receiving sacubitril/valsartan at a stable dose of at least 49/51 mg bid for at least 4 weeks before Screening.
- Receiving other guideline recommended HF therapies as deemed appropriate by the investigator and that are stable in dose for at least 4 weeks before Screening, unless contraindicated, not tolerated, or not available to patient. Other evidence-based therapy for heart failure should also be considered, e.g., cardiac resynchronization therapy and an implantable cardioverter-defibrillator in selected patients, as recommended by guidelines.

Key Exclusion Criteria

- Current acute decompensated HF (exacerbation of chronic HF manifested by signs and symptoms that may require intravenous therapy) or hospitalization for HF within 3 months prior to screening.
- Office systolic blood pressure (SBP) ≥ 180 mmHg or < 105 mmHg at screening or at randomization.

- In subjects with ACEI/ARB medication at screening, previous inability to tolerate any dose of sacubitril/valsartan or any ARB (as per the investigator's judgment).
- Serum potassium > 5.4 mmol/L at screening.
- Estimated GFR (eGFR) < 30 mL/min/1.73m^2 at screening as measured by the CKD-EPI formula.
- Known history of angioedema.

Data Monitoring/Other Committee:

Yes (see Section 10.1.4 Committees Structure)

Key words

Heart failure, randomized, double-blind, dose finding, XXB750

1.2 Schema

Figure 1-1 Study design

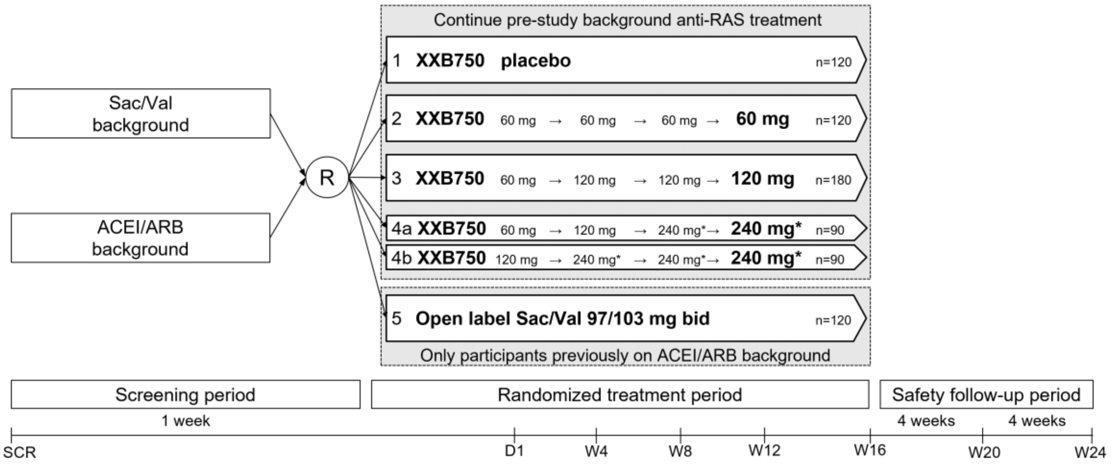

Sac/Val: Open-label sacubitril/valsartan. *May be modified per adaptive design feature of the study (Section 4.6). Doses of Sac/Val, ACEI or ARB in arms 1-4 equal to dose at randomization.

1.3 Schedule of activities (SoA)

Table 1-1 lists all scheduled assessments throughout the study. In addition to the protocol-required visits, participants may be seen at any time throughout the study at the discretion of the investigator to follow-up on any new laboratory abnormalities or adverse events (AEs).

Participants should be seen for all visits/assessments as outlined in the SoA or as close to the designated day/time as possible. Day 1 will be considered the reference visit for all study visits during the randomized treatment period, regardless of occurrence of any unscheduled visits. If one visit is missed, postponed, or brought forward, it should not result in the next visit being postponed or brought forward. For details on administration of injectable study medication in

this situation, please see Section 6.1.2. The next visit, if at all possible, should adhere to the original time schedule.

Participants who discontinue from study treatment are to complete the end of treatment visit as soon as possible, and continue to attend the follow-up visits as indicated in the SoA.

Participants who discontinue from the study should be scheduled for a final evaluation visit if they agree, as soon as possible, at which time all the assessments listed for the final visit of the randomized treatment period will be performed. At this final visit, all dispensed study treatment should be reconciled, and the adverse events and concomitant medications not previously reported must be recorded on the appropriate CRF.

Patient Reported Outcome (PRO) measure(s) must be completed by the participant before any assessments are performed at any given visit.

Throughout the randomized treatment and safety follow-up periods, participants will undergo safety and biomarker laboratory assessments as well as pharmacokinetic (PK) sampling per the schedule shown in Table 1-1. After randomization, all laboratory evaluations for planned visits will be performed through the central laboratory or other authorized laboratory. Unscheduled laboratory assessments can be performed locally as necessary or as permitted by the protocol.

As per Section 4.5, during a public health emergency as declared by local or regional authorities i.e., pandemic, epidemic or natural disaster that limits or prevents on-site study visits, alternative methods of providing continuing care may be implemented by the Investigator as the situation dictates. If allowable by a local health authority, national and local regulations and depending on operational capabilities, phone calls, virtual contacts (e.g., tele consultation) or visits by site staff/off-site healthcare professional(s) staff to the participant´s home, can replace certain protocol assessments, for the duration of the disruption until it is safe for the participant to visit the site again. If the Investigator delegates tasks to an off-site healthcare professional, the Investigator must ensure the individual(s) is/are qualified and appropriately trained to perform assigned duties. The Investigator must oversee their conduct and remains responsible for the evaluation of the data collected.

Table 1-1 Assessment Schedule

Epoch	Screening	Randomized Treatment										Safety Follow-up	
**Visit Name**	**Screening**	**Randomization**	**Day 2 Call**	**Week 2**	**Week 4**	**Day 30 Call**	**Week 8**	**Day 58 Call**	**Week 12**	**Day 86 Call**	**Week 16 EOT**	**Week 20**	**Week 24 EOS**
**Visit Numbers[1]**	**10**	**101**	**102**	**103**	**104**	**105**	**106**	**107**	**108**	**109**	**9998**	**201**	**202**
**Days**	**-7 to 1**	**1**	**2**	**14**	**28**	**30**	**56**	**58**	**84**	**86**	**112**	**140**	**168**
Informed consent	X												
Genetic consent (optional)	X												
Inclusion / Exclusion criteria	X	X											
Demography	X												
Medical history/current medical conditions	X												
Alcohol and smoking history	X												
Prior/Concomitant Medications	X	X	X	X	X	X	X	X	X	X	X	X	X
Complete Physical Examination	S										S		S
Abbreviated physical examination		S		S	S		S		S			S	
Body Height (H) and Weight (W)	H/W	W		W	W		W		W		W	W	W
Electrocardiogram (ECG)	X										X		X
Echocardiogram (optional)	S												
Vital Signs	X	X		X	X		X		X		X	X	X
Central lab blood chemistry	X			X[2]	X[2]		X[2]		X[2]		X	X[2]	X
Hematology	X										X		X
Coagulation Panel	X			X	X		X		X		X	X	X
NT-proBNP	X	X			X[3]		X[3]		X[3]		X	X	X
NYHA classification (HF signs and symptoms)	X	X			X		X		X		X	X	X
IWRS/IRT Registration	X	X		X	X		X		X		X	X	X
Study drug dispensation/administration[4]		X		X	X		X		X				
Study medication compliance[5]				S	S		S		S		S		

Epoch	Screening	Randomized Treatment										Safety Follow-up	
**Visit Name**	**Screening**	**Randomization**	**Day 2 Call**	**Week 2**	**Week 4**	**Day 30 Call**	**Week 8**	**Day 58 Call**	**Week 12**	**Day 86 Call**	**Week 16 EOT**	**Week 20**	**Week 24 EOS**
**Visit Numbers[1]**	**10**	**101**	**102**	**103**	**104**	**105**	**106**	**107**	**108**	**109**	**9998**	**201**	**202**
**Days**	**-7 to 1**	**1**	**2**	**14**	**28**	**30**	**56**	**58**	**84**	**86**	**112**	**140**	**168**
Adverse Events		X	X	X	X	X	X	X	X	X	X	X	X
Biomarker urine collection		X[3]					X[3]		X[3]		X		
Biomarker blood collection		X[3,6]					X[3]		X[3]		X		
PK blood collection[7]		X[8]			X[3,8]				X[3,8]		X		X
Anti-drug antibody[7]		X[3]			X[3]				X[3]		X		X
Exploratory DNA Sampling (optional)[9]		X											
Urinalysis	X	X		X	X		X		X		X	X	X
Assessment of fertility	S												
Pregnancy Test (serum)	X										X		X
Pregnancy Test (urine)		S			S		S		S			S	
KCCQ questionnaire		X									X		
Study period completion information	X										X		X

[X] Assessment to be recorded in the clinical database or received electronically from a vendor
[S] Assessment to be recorded in the source documentation only
[1] Visit structure given for internal programming purpose only
[2] Abbreviated safety panel (Please refer to Table 8-1)
[3] Samples must be taken prior to study drug administration.
[4] For participants on ACEI and randomized to treatment arm 5, a 36-hour washout period must be observed: Open-label sacubitril/valsartan to be started in evening of Day 2. Dispensing visit at Week 2 is for only patients randomized to receive open-label sacubitril/valsartan, which can be used as a dose titration visit.
[5] Only applicable to participants randomized to conversion to open-label sacubitril/valsartan (treatment arm 5)
[6] Additional collection with P100 tube. Please refer to Section 8.8.
[7] Not applicable to participants randomized to conversion to open-label sacubitril/valsartan (treatment arm 5)
[8] Sample to be collected 2-4h post dose.
[9] To be collected only for participants signing optional DNA consent.

2 Introduction

2.1 Study rationale

The purpose of this study is to investigate the efficacy, safety, and tolerability of three dose levels of XXB750 in HF patients (NYHA class II-III) with LVEF < 50% receiving standard of care (SoC) HF therapies. This study will provide evidence to understand the dose-response relationship of XXB750 in reducing NT-proBNP, a prognostic biomarker of HF clinical outcomes, and to choose the optimal dosing regimen to investigate in future phase 3 studies. In addition, this study will help to assess the benefit of XXB750 relative to placebo when given on top of SoC HF therapy, as well as its benefit in ACEI/ARB treated patients compared to converting them to sacubitril/valsartan (i.e., direct comparison of XXB750 vs sacubitril/valsartan).

2.2 Background

Heart failure (HF) is a major public health problem associated with a high mortality rate, frequent hospitalizations, and poor quality of life (QoL). Worldwide, an estimated 64.3 million people were living with heart failure in 2017, with a prevalence between 1-3% of the general adult population in developed countries (Groenewegen et al 2020, Savarese et al 2022). In the United States, the prevalence of HF is 6 million individuals, which is approximately 1.8% of the total US population (Roger 2021). Heart failure is associated with frequent hospitalizations, with 30-40% of patients having a history of hospitalization for HF (Crespo-Leiro et al 2016) and 22% of patients being re-admitted for HF within 1 year of their initial diagnosis (Lawson et al 2019).

Heart failure is a complex clinical syndrome that most commonly causes structural or functional impairment of ventricular filling or emptying, ultimately resulting in the heart's inability to meet the body's need for adequate supplies of oxygenated blood. The natural course of HF includes deterioration in symptoms, often leading to repeated hospitalizations for acute decompensations, and eventually death most frequently due to arrythmia (i.e., sudden death) or progressive pump failure (Desai et al 2015).

Heart failure with reduced left ventricular ejection fraction (HFrEF) is regarded as a common type of HF and is characterized by an LVEF ≤ 40%; it is the best studied type of HF. The current standard of care of pharmacologic treatment for HFrEF is comprised of four main drug classes: blockers of the renin angiotensin system (RAS) with either angiotensin converting enzyme inhibitors (ACEI), anangiotensin II receptor blockers (ARB), or angiotensin receptor neprilysin inhibitor (ARNI), β-blockers, mineralocorticoid receptor antagonists (MRAs) and sodium-glucose cotransporter 2 (SGLT2) inhibitors. These four classes have been shown to reduce morbidity and mortality in patients with HFrEF and are recommended as standard-of-care treatment as per recently published guidelines for the treatment of HF (McDonagh et al 2021, Heidenreich et al 2022). Adjacently situated to HFrEF on the LVEF spectrum is heart failure with mildly reduced ejection fraction (HFmrEF), which is characterized by an LVEF > 40% but < 50%.

Evidence based on analyses of large outcome trials suggests that drug classes effective in HFrEF may also be effective in HFmrEF patients, thereby supporting recommendations for using them

in this HF population in recent international HF treatment guideline updates (McDonagh et al 2021, Heidenreich et al 2022).

Despite the treatment of HFrEF patients with quadruple therapy as recommended by guidelines, it is projected that lifetime morbidity and mortality remain high (Vaduganathan et al 2020, Tromp et al 2022). In a cross-trial analysis, while overall mean survival was estimated to be prolonged with quadruple therapy as compared to conventional therapy, survival remains reduced by at least 10 years compared to the population's average life expectancy (Vaduganathan et al 2020). Similarly, a recent network meta-analysis (Tromp et al 2022) found prolonged survival with quadruple therapy but with even higher mortality rates than in the cross-trial analysis cited above. Thus, with considerable residual morbidity and mortality rates, the need for better therapies for HF remains unmet and high.

Disease development and progression of HF is intimately linked to an excessive neurohormonal response, resulting in an imbalance between detrimental pathogenic factors and compensatory mechanisms. Overactivation of the RAS and sympathetic nervous system (SNS) is believed to be harmful in the long-term because they result in sodium retention, fluid overload, increased blood pressure, and cardiac fibrosis and hypertrophy, especially in the absence of adequate levels of natriuretic peptides (NPs), which are regarded as a beneficial compensatory response via their direct vasodilator and natriuretic actions, as well as their antiproliferative and antihypertrophic effects (Levin et al 1998, Gardner et al 2007, Pandey 2008). This has been demonstrated by sacubitril/valsartan, an ARNI that simultaneously blocks angiotensin II, while enhancing NP action by blocking NP metabolism via the enzyme neprilysin, resulting in improved survival and reduced morbidity (McMurray et al 2014).

However, elevating NP levels by blocking neprilysin-mediated degradation (NEPi) may not fully leverage the beneficial potential of the NP system as the extent of NEPi-mediated NP agonism is limited by the amount of endogenous ANP and/or BNP levels. Also, neprilysin inhibition blocks only one pathway of NP breakdown, leaving other NP metabolic pathways, such as enzymatic degradation by dipeptidyl peptidase 4 and deactivation via NPR3 receptor binding, unaffected.

Direct natriuretic peptide type 1 receptor (NPR1) agonism may offer a more efficient way of targeting the NP system. In a recently published first-in-human (FIH) study, the NPR1 agonist M-atrial natriuretic peptide (MANP), an analog of human ANP, was shown to increase cGMP levels and natriuresis and to lower aldosterone levels and blood pressure when administered subcutaneously (SC) to hypertensive patients (Chen et al 2021).

XXB750 is a fully human monoclonal IgG1 antibody agonist of the NPR1. The action of XXB750 is specific for NPR1, with XXB750 selectively binding to and activating NPR1, while it does not bind to NPR3 or activate NPR2 in vitro. XXB750 replicates the action of ANP (increasing plasma cGMP, lowering BP and increasing diuresis) in non-clinical pharmacology studies. It effectively substitutes for ANP in ANP knockout mice, reversing both cardiac hypertrophy and elevations in NT-proBNP. XXB750 also increases plasma cGMP and lowers BP in cynomolgus monkeys.

XXB750 has a half-life of CCI [redacted] in healthy volunteers and has been shown to be safe and well tolerated in single SC doses up to 450 mg in healthy volunteers with mildly elevated blood pressure. Pharmacodynamic effects (cGMP increase, blood pressure lowering) of XXB750 (for

doses ≥ 120 mg) were of greater magnitude than what was seen with sacubitril in healthy volunteers. Also, single 120 mg SC doses of XXB750 have been tested in HF patients with ACEI/ARB and other background HF medications. XXB750 was found to have an acceptable safety and tolerability profile. This data, together with evidence of the beneficial effects of ANP and other ANP mimetics in heart failure patients, provides a scientific rationale to develop XXB750 for the treatment of heart failure. Refer to the XXB750 investigator's brochure for more detailed information on the safety, pharmacokinetics, and pharmacodynamic biomarker data available on this biologic.

The current study is a combined phase 2a/2b study which aims to establish proof-of-concept and dose range relationship for XXB750 in reducing NT-proBNP levels in HF patients (NYHA class II-III) with LVEF < 50%, to inform the dose regimen of XXB750 to be tested in Phase 3, and to further characterize the benefit-risk profile of XXB750 in patients with HF.

2.3 Benefit/Risk assessment

Participants are expected to benefit from extensive monitoring of their HF condition and symptoms during the course of the study. Participants will continue to receive their background standard of care HF medications, including ACEIs/ARBs or ARNI, during the duration of the study. Some patients treated with ACEIs will be randomly assigned to receive open-label the ARNI sacubitril/valsartan, which is considered by international HF treatment guidelines to be preferred over ACEIs in HFrEF due to improved efficacy in reducing long-term morbidity and mortality (Fiuzat et al 2022, Heidenreich et al 2022, McDonagh et al 2021). Participants randomized to the investigational treatment XXB750 may potentially benefit from improved augmentation of the natriuretic peptide system.

During the total study duration of 24 weeks, a total of 8 in-person visits will be conducted at the site, with 3 of the visits lasting up to several hours. Study visit procedures include blood draws to obtain blood samples totally approximately 183 mL to 207 mL over the duration of the study, depending on which sub-studies the patient participates in. Patients included in this study are expected to have no background illnesses that will prevent them from producing sufficient new blood to compensate for the blood obtained for the study over its duration. Some patients may experience bruising from the phlebotomy needed in obtaining the blood samples. Study participants may also experience some discomfort associated with some study procedures, such as blood pressure measurements and urine sample collection.

Due to the mode of action of XXB750 and other background medications the patient may be taking for HF and other comorbidities, there is a risk of occurrence of hypotension and hypotension-related symptoms, such as dizziness, lightheadedness, orthostasis, postural symptoms, pre-syncope or syncope. Reflex rise in heart rate has also been reported with XXB750. In patients randomized to XXB750 the risk of symptomatic hypotension is greatest in the first few days after dosing but can potentially happen at any time during the study. Participants will receive a blood-pressure monitoring device to enable BP assessments at home as needed and per the investigator's request in the event they experience potential hypotension-related AEs. Additionally, participants will be contacted 2-3 days after each dose administration via a scheduled telephone call to monitor their tolerability and inquire about their general well-being. The current protocol includes a guidance on how to address symptomatic hypotension in the context of this study (Section 10.7).

All injections of XXB750 and its matching placebo will be administered during site visits as single SC injections of 1.6 mL, which may cause injection site reactions or bruising. Like many monoclonal antibodies, XXB750 may result in formation of anti-drug antibodies (ADA). Although available data on ADA formation do not indicate that they result in any safety problems or interfere with the PK or PD of XXB750, they will be studied carefully in this study and other ongoing XXB750 studies to better understand their significance. Hypersensitivity reactions are a potential risk for XXB750, although it has not been observed so far in XXB750 clinical studies. Hypersensitivity occurrence and its clinical significance will be monitored during the current the study. Participants will be monitored at the site for a minimum of 20 minutes after each injection. For additional information on XXB750 and its available safety information, please refer to the latest edition of the XXB750 investigator's brochure.

Women of child-bearing potential must be informed that taking the study treatment may involve unknown risks to the fetus if pregnancy were to occur during the study and agree that in order to participate in the study, they must adhere to the contraception requirements outlined in the exclusion criteria. If there is any question that the participant will not reliably comply with the recommendations of contraception, they should not be entered or continue in the study. In general, the levels of a monoclonal antibody (mAb) in semen are expected to be low, and therefore, the subsequent levels in a female partner would be negligible. Based on this principle, the probability that XXB750 would cause teratogenicity is very low. Therefore, no restrictions will be placed on male participants with regard to sexual activity or condom use.

Patients randomized to receive open-label sacubitril/valsartan in place of their background ACEI/ARB may experience hypotension/hypotension-related side effects. They may also experience renal dysfunction and hyperkalemia, although the latest published evidence indicates that sacubitril/valsartan results in a lower incidence of these side effects that ACEIs/ARBs. Angioedema has also been reported with sacubitril/valsartan. The risk of angioedema will be minimized by observing a 36-hour ACEI- and sacubitril/valsartan-free washout period whenever patients are switched from an ACEI to open-label sacubitril/valsartan (such as at the start of the randomized treatment period and the start of the safety follow-up period, as applicable). Also, patients with history of angioedema will be excluded from the trial. For more potential information and side effects of sacubitril/valsartan, please refer to your locally approved sacubitril/valsartan label.

The risk to participants in the study will be minimized by compliance with the eligibility criteria, close clinical monitoring, including home BP measurement and a follow-up call after each injection of study drug. Eligible participants with eGFR $\geq$ 30 ml/min/1.73m^2 will enter the study with close monitoring of renal function and serum potassium at every scheduled study site visit to ensure their safety. All participants will be carefully monitored by the investigators for satisfactory risk-to-benefit ratio.

A periodic review of the safety data by an independent Data Monitoring Committee (DMC) will further enhance safety for the study participants. Additionally, enrollment of participants to treatment arms with doses of XXB750 higher than 120 mg will only start after the benefit-risk balance of XXB750 administration is deemed to be acceptable by the DMC. This will be evaluated in an interim safety analysis that includes data of the lower dose arms from this study along with additional data from the ongoing phase 1b study administrating XXB750 up to 240 mg in patients with HF.

Should a participant develop clinically significant AEs during the course of the study, the blinded study treatment may, as per the investigator's discretion, be temporarily or permanently discontinued to minimize the risk to participants, and they will be managed by the treating physicians according to local standard of care.

Given the close monitoring, criteria for patient selection, and the known safety and tolerability profile of the drug in humans, it is considered that overall risks for participants in this study are acceptable.

3 Objectives, endpoints, and estimands

Table 3-1 Objectives and related endpoints

Objective(s)	Endpoint(s)
**Primary objective(s)**	**Endpoint(s) for primary objective(s)**
• To evaluate the efficacy and dose-response relationship of three XXB750 target dose levels compared to placebo in reducing NT-proBNP from baseline to Week 16 in symptomatic HF patients with LVEF < 50% treated with standard of care, including ACEI/ARB or sacubitril/valsartan.	• Change in log NT-proBNP from baseline to Week 16
**Secondary objective(s)**	**Endpoint(s) for secondary objective(s)**
• To evaluate the treatment effect of the highest XXB750 target dose level compared to placebo in reducing NT-proBNP from baseline to Week 16 in symptomatic HF patients with LVEF < 50% treated with standard of care, including ACEI/ARB or sacubitril/valsartan.	• Change in log NT-proBNP from baseline to Week 16
• To evaluate the treatment effect of combined two highest XXB750 target dose levels administered in addition to a background of ACEI/ARB versus conversion from ACEI/ARB to sacubitril/valsartan, in reducing NT-proBNP from baseline to Week 16.*	• Change in log NT-proBNP from baseline to Week 16
• To evaluate the safety and tolerability of XXB750 up-titration regimens and dose levels.	• Adverse events (incidence and severity, including but not limited to, hypotension, tachycardia, bradycardia, hypersensitivity and injection site reactions), vital signs (blood pressure, pulse), safety laboratory tests, and ECG parameters from baseline to end of study (EOS).
**Exploratory objective(s)**	**Endpoint(s) for exploratory objective(s)**
• To evaluate the effects of highest XXB750 target dose level compared to placebo in improving a composite hierarchical outcome of: 1) time to all-cause death, 2) number of HF hospitalizations, 3) Kansas City Cardiomyopathy Questionnaire (KCCQ) clinical summary score (CSS) categorical change, 4) NYHA class change , 5) relative change in NT-proBNP levels between baseline and Week 16.	• Hierarchically ordered composite consisting of: 1) time to all-cause death up to Week 16; 2) number of HF hospitalizations up to Week 16; 3) categorical KCCQ CSS change from baseline to Week 16; 4) NYHA class change from baseline to Week 16; 5) relative change in NT-proBNP levels from baseline to Week 16.
• To evaluate changes in each domain of the KCCQ, including clinical summary score (CSS) and overall summary score (OSS), from baseline to Week 16.	• Change in all domains of the KCCQ from baseline to Week 16.
• To evaluate the effects of XXB750 on slowing the rate of decline in estimated glomerular filtration rate (eGFR) from baseline to Week 16.	• Estimated glomerular filtration rate (eGFR) at scheduled assessment visits.

Objective(s)	Endpoint(s)
• To evaluate the change in biomarkers related to heart failure, cardiovascular disease and renal function from baseline to Week 8 and to Week 16.	• Change from baseline in biomarkers including, but not limited to, plasma and urinary cGMP, urinary cGMP-to-creatinine ratio, urinary albumin-to-creatinine ratio, BNP and hs-Troponin, at Week 8 and Week 16.
• To evaluate the change in NYHA class and in signs and symptoms of heart failure from baseline to Week 16.	• Change in NYHA class and signs and symptoms of HF from baseline to Week 16.
• To explore the population pharmacokinetic (PK) properties of XXB750 in HF patients.	• Population PK analysis results including mathematic models and parameters.
• To evaluate immunogenicity (IG) of XXB750.	• Detection of immune response markers, including anti-drug antibodies.

*In the event treatment arms 4a and 4b are eliminated (see Section 4.6), the second secondary objective will evaluate only the 120 mg XXB750 arm to sacubitril/valsartan arm.

3.1 Primary estimands

The primary clinical question of interest is:

Is there a dose-response signal, and if so, what is the dose-response relationship among three XXB750 target dose levels and placebo in reducing NT-proBNP from baseline to Week 16 in participants with HF (NYHA classes II-III) and LVEF < 50%, treated with standard of care, including ACEI/ARB or sacubitril/valsartan, regardless of type of baseline background HF therapy, discontinuation from the study treatment, change in the dose of concomitant medications and receiving prohibited concomitant medication?

The justification for the primary estimand is that it will capture both the effects of XXB750 and the effects of changes in other HF medications. This will provide a clear assessment of the XXB750 treatment effect in clinical practice versus placebo.

The primary estimand is described by the following attributes:

1. Population: participants with HFrEF and HFmrEF (LVEF < 50%), NYHA classes II-III and elevated NT-proBNP levels at screening while taking optimal guideline-directed HF therapy.
2. Variable: change in log NT-proBNP from baseline to 16 weeks
3. Treatment of interest: Treatment groups with three XXB750 target dose levels, and placebo all administered on top of ACEI/ARB or sacubitril/valsartan and other guideline-recommended standard of care therapy for HF.

Handling of intercurrent events:

- Discontinuation of study treatment: ignore (treatment policy strategy)
- Change in concomitant medications: ignore (treatment policy strategy)
- Receiving prohibited concomitant medication: ignore (treatment policy strategy)

4. The summary measure: difference in variable means between treatments. Since the variable in attribute 2 is equivalent to the log of ratio of Week 16 NT-proBNP over baseline NT-proBNP, and the difference in variable means between 2 treatment groups is the log of ratio of ratio to baseline of the 2 groups, therefore, the summary measure can be

back-transformed by exponentiation and expressed as ratio of geometric mean of ratio to baseline of the 2 treatment groups.

3.2 Secondary estimands

Treatment effect of the highest XXB750 target dose level compared to placebo in reducing NT-proBNP from baseline at Week 16 in HF patients treated with standard of care, including ACEI/ARB or sacubitril/valsartan

The first secondary clinical question of interest is:

What is the effect of the highest XXB750 dose level versus placebo in reducing NT-proBNP level from baseline to Week 16 in participants with heart failure (NYHA classes II-III) and LVEF < 50%, treated with standard of care, including ACEI/ARB or sacubitril/valsartan, regardless of type of baseline background HF therapy, discontinuation from study treatment, change in the dose of concomitant medications, and receiving prohibited concomitant medication?

The justification for the first secondary estimand is that it will capture both the effects of study medication and the effects of changes in guideline-directed concomitant medications. This will provide a clear assessment of the XXB750 treatment effect in clinical practice versus placebo.

The attributes and handling of intercurrent events are the same as those described in primary estimand except that the treatment of interest being the highest XXB750 dose level and placebo, instead of all XXB750 dose levels as described below:

Highest XXB750 dose level versus placebo in combination with guideline-recommended standard of care therapy for HF.

Treatment effect of combined two highest XXB750 target dose levels in addition to a background of ACEI/ARB versus conversion from ACEI/ARB to sacubitril/valsartan, in reducing NT-proBNP from baseline to Week 16

The clinical question of interest for this objective is:

What is the effect of combined two highest XXB750 dose groups in addition to a background of ACEI/ARB versus conversion from ACEI/ARB to sacubitril/valsartan in reducing NT-proBNP level from baseline to Week 16 in participants with heart failure (NYHA classes II-III) and LVEF < 50% regardless of type of baseline background HF therapy, discontinuation from the study treatment, change in the dose of concomitant medications and receiving prohibited concomitant medication?

The attributes and handling of intercurrent events are the same as those described in primary estimand except that:

- Population: participants with HFrEF and HFmrEF (LVEF < 50%), NYHA classes II-III and elevated NT-proBNP levels and on ACEI/ARB at screening while taking other guideline-directed HF therapy.
- Treatment of interest: two highest XXB750 dose levels in addition to ACEI/ARB background medication versus conversion from ACEI/ARB to sacubitril/valsartan in

combination with guideline-recommended standard of care therapy for HF. In the event treatment arms 4a and 4b are eliminated (see Section 4.6), treatment of interest will be only the 120 mg dose XXB750 dose level in addition to ACEI/ARB background medication versus conversion from ACEI/ARB to sacubitril/valsartan in combination with guideline-recommended standard of care therapy of HF.

Evaluation of the safety and tolerability of XXB750 up-titration regimens and target dose levels

Adverse events (AEs) and serious AEs, laboratory parameters and vital signs will be evaluated and summarized in each treatment group for different treatment periods (randomized treatment period and safety follow-up period). Please refer to Section 9.4.2 for analysis details.

4 Study design

4.1 Overall design

Study CXXB750A12201 is a multicenter, randomized, placebo- and active-controlled, parallel-group phase 2 study which is comprised of three periods (Figure 1-1):

- A screening period of approximately 7 days
- A 16-week parallel-group randomized treatment period on either
  i) double-blind placebo-controlled treatment (XXB750 vs. placebo) or
  ii) open-label treatment (sacubitril/valsartan)
- An 8-week safety follow-up period

The study will randomize adult participants with LVEF < 50% receiving ACEI/ARB/ARNI and guideline-recommended HF therapies for HFrEF or HFmrEF to three XXB750 target dose levels; a cohort of patients treated with ACEI/ARB before the study will be randomized to be converted to open-label sacubitril/valsartan in place of their pre-study ACEI/ARB. The study will randomize a total of approximately 720 patients. The percentage of patients with LVEF > 40% will be limited to approximately 25% of the total sample.

Refer to Figure 1-1 for an overview of study design, and to Section 6.1.2 for details on the administered doses in the different treatment arms.

A staggered approach to enrollment will be followed in this protocol. The first group of 300 participants will be randomized to placebo (treatment arm 1), XXB750 60 mg target dose (treatment arm 2), XXB750 120 mg target dose (treatment arm 3), and conversion to sacubitril/valsartan (treatment arm 5). The independent Data monitoring Committee will complete a safety analysis of the XXB750 doses tested and if it is safe to allow the dosing of patients with XXB750 planned top dose of 240 mg (i.e., allowing enrollment in arms 4a and 4b as planned). See Section 4.6 for details on planned interim analyses and adaptive design features.

4.1.1 Screening Period

The purpose of the screening period is to assess participants' eligibility to enter into the randomized treatment period of the study. At the Screening visit, participants will be asked to

review and sign the inform consent form (ICF) before performing any study-related assessments. The inclusion and exclusion criteria will be assessed to verify the candidate's eligibility for enrollment into the study.

HF, cardiovascular (CV) and other medical history will be collected. An ECG will be performed. Protocol-specified biochemical criteria, including the required eGFR, potassium, and NT-proBNP levels will be assessed using a central laboratory. The sample obtained to assess NT-proBNP for study eligibility must be collected on the same day as performing the ECG. Concomitant medications will be recorded. In order to be eligible, participants are required to receive sacubitril/valsartan or ACEI/ARB for their HF at study entry, with minimum doses of sacubitril/valsartan 49/51 mg bid or an ACEI/ARB equivalent to enalapril 10 mg/day that have been stable/unchanged for at least 4 weeks. Table 4-1 lists total daily doses of commonly used ACEIs and ARBs with doses considered equivalent to enalapril 10 mg/day for the purpose of eligibility.

Table 4-1 Minimum required pre-study daily doses of ACEIs and ARBs

ACEIs	Minimum daily dose	ARBs	Minimum daily dose
Enalapril	10 mg	Valsartan	160 mg
Lisinopril	10 mg	Candesartan	16 mg
Benazepril	20 mg	Azilsartan	40 mg
Captopril	100 mg	Eprosartan	400 mg
Cilazapril	2.5 mg	Fimasartan	60 mg
Delapril	15 mg	Irbesartan	150 mg
Fosinopril	20 mg	Losartan	50 mg
Imidapril	10 mg	Olmesartan	10 mg
Moexipril	7.5 mg	Telmisartan	40 mg
Perindopril	4 mg		
Quinapril	20 mg		
Ramipril	5 mg		
Temocapril	2 mg		
Trandolapril	2 mg		
Zofenopril	30 mg		

Other HF medication and their doses will be recorded. If the participant is not taking other classes of HF medications that are recommended per locally applicable guidelines and available locally such as beta-blockers, MRAs and/or SGLT2 inhibitors, the investigator must provide a qualifying reason for that on the appropriate electronic case report form (eCRF). The participant will continue his/her therapy, including his/her HF medications with no change during the screening period.

Refer to Table 1-1 and Section 8.1 for a full list and details of screening procedures, respectively.

4.1.2 Randomized Treatment Period

Only participants who successfully complete the screening period by meeting all inclusion criteria and none of the exclusion criteria (refer to Section 5 for a full list of inclusion and

exclusion criteria) will be eligible for randomization and invited to return to the site to complete the procedures of the Randomization visit (Day 1) as indicated in Table 1-1.

Baseline values for vital signs, biomarkers (including NT-proBNP), PK, HF signs and symptoms (as well as NYHA class), and KCCQ questionnaire will be established at this visit.

At randomization participants will be stratified by pre-study anti-RAS treatment of ACEI/ARB vs. ARNI. Participants in the pre-study ACEI/ARB stratum (N = 320) will be randomized at a final 4:4:6:3:3:12 ratio to receive either placebo (n = 40; 12.5% of participants) or XXB750 at a target dose of 60 mg, 120 mg, 240 mg with low starting dose, or 240 mg with a high starting dose (n = 160; 50% of participants across XXB750 arms) in a double-blind fashion (all given every four weeks) or be converted to receive sacubitril/valsartan at a target dose of 97/103 mg bid (n= 120; 37.5% of participants). Participants in the pre-study ARNI stratum (N = 400) will be randomized at a final 4:4:6:3:3 ratio to receive either placebo (n= 80; 20% of participants) or XXB750 at a target dose of 60 mg, 120 mg, 240 mg with low starting dose, or 240 mg with a high starting dose (n = 320; 80% of participants across XXB750 arms) in a double-blind fashion. Thus, in participants randomized to injectable study medication (treatment arms 1,2,3,4a, 4b), it is anticipated to result in a 1:2 ratio of ACEI/ARB vs. ARNI background anti-RAS treatment. All study medications will be taken in addition to other background medications.

For participants on prior ACEI treatment and randomized to treatment arm 5 (open-label sacubitril/valsartan), an ACEI-free wash-out period of 36 hours is mandatory to minimize the potential risk of angioedema. For example, if a participant's last dose of pre-study ACEI was on Wednesday morning, he/she should be instructed to take the first dose of the open-label study sacubitril/valsartan on Thursday evening. Please refer to Section 6.1.2 for details on treatment arms, the up-titration regimens, and the doses the participant will receive at the respective visits.

Participants will undergo blood sampling and other assessments at each site visit and be contacted two days after each injection of study medication as outlined in Table 1-1 and described in Section 8.

The modification of participants background heart failure therapy should be avoided if at all possible during the randomized treatment period, which is planned to last for 16 weeks, unless it is truly required to ensure patient safety. The dosing schedule of the study medications should be adhered to as instructed by the protocol.

Please refer to Section 6.4.1 for recommendations for management of select adverse events and to Section 6.5 for guidance on dose modification of the study medication should the need arise.

4.1.3 Safety Follow-up Period

Because XXB750 has a half-life of CCI [redacted] and might be detectable in the blood for approximately 90 days after administration of the last dose, the safety follow-up period is included in the study design to monitor the safety of participants while the study medication is tapering off. After completing the procedures of the Week 16 visit, the investigator may modify the participant's medication regimen as necessary for the patient's HF status and as per guidelines. This may include the replacement of the oral study treatment sacubitril/valsartan with commercially available sacubitril/valsartan, ACEI, or ARB per the investigator's clinical

judgment and local and international HF treatment guidelines and according to locally approved medication label. If at the start of the safety follow-up period the investigator wishes to place patients who were previously randomized to open-label sacubitril/valsartan on an ACEI, a 36 hour sacubitril/valsartan-free washout period must be observed before the patient begins to take the ACEI in order to minimize the likelihood of occurrence of angioedema. For example, if a participant's last dose of open-label study sacubitril/valsartan is on Wednesday evening, the first dose of the ACEI should be taken on Friday morning.

Two study visits will be conducted to collect safety data, NT-proBNP, and PK laboratory assessments and to monitor the participant’s overall health status (i.e., Week 20 and Week 24 visits).

Participants who prematurely discontinue the study medication should complete similar safety follow-up procedures, which should be scheduled 8 and 12 weeks after the last dose administration of XXB750 or its matching placebo, respectively.

4.2 Scientific rationale for study design

The current study aims to investigate several aspects of the efficacy and safety of XXB750 in HF patients with LVEF < 50%. The primary objective of the study aims to characterize the dose-response pattern of XXB750 when added to treatment with ACEI/ARB or sacubitril/valsartan in patients with LVEF < 50%. A secondary objective of the study aims to determine the relative efficacy of modulating the NP system via NPR1 agonism compared to modulating it via neprilysin inhibition in reducing NT-proBNP in this patient population. Moreover, since XXB750 is the first injectable long-acting NPR1 agonist, the study aims to identify an optimal dosing regimen that best balances risks with benefits of XXB750.

With all the above in mind, the current study design is considered to be optimal for accomplishing its objectives and for arriving to sound and reliable answers to the scientific questions of the study, while minimizing the burden to the participants and to the investigators and ensuring patient safety. Regarding the primary objective, i.e., the dose-response finding (DRF) aspect of the study, this is a randomized, double-blind, and placebo-controlled study. This design is typical for DRF studies as it compares the effects of three doses of XXB750 to placebo (all given SC every 4 weeks) to help form a dose-response curve with change in NT-proBNP level as the endpoint. Patients will be randomized to receive XXB750 or its matching placebo in a blinded manner on top of other background guideline-direct HF therapy. As a result, this design maximizes the chances of producing reliable and internally and externally valid results to support sound conclusions about the efficacy and safety of the experimental treatment (i.e., XXB750 in this study). A multicenter setting has been chosen to ensure adequate enrollment into the study and to include an internationally representative sample that accounts for regional and local differences in practice norms.

Based on extrapolation from data established for sacubitril/valsartan, it is expected that the full effect of NT-proBNP reduction will be achieved in all treatment groups after 8 weeks of study treatment at target dose levels. Thus, considering that target dose levels are reached at Week 8 in all treatment arms, the 16-week duration of the randomized treatment period is expected to be sufficient to examine the effect of each target dose level of XXB750 to lower NT-proBNP.

The current design is also adequate for assessing the relative effect of the investigational modality of NP modulation, i.e., NPR1 agonism with XXB750, compared to the NP modulation mechanism currently available in clinical practice, i.e., neprilysin inhibition using sacubitril, in reducing NT-proBNP. Because the only available neprilysin inhibitor is commercially delivered via a compound with valsartan, the study is designed to include sufficient ACEI/ARB-treated participants who will be randomized to receive either XXB750 at different target dose levels (i.e., NPR1 agonist + ACEI/ARB cohort) or be converted to receive open-label sacubitril/valsartan at the approved target dose in place of their ACEI/ARB (i.e., neprilysin inhibitor + ARB cohort). The open-label design of the sacubitril/valsartan cohort optimally balances the degree of burden to patients and complexity of the study, while ensuring the conclusions based on this cohort are scientifically robust. Indeed, the primary endpoint of the study is change in NT-proBNP, which is an objective measure.

To better explore the safety profile of XXB750 and the most optimal regimen of its administration, patients assigned to the highest XXB750 target dose will be further assigned to one of two up-titration regimens to be administered in a blinded manner. The regimens differ in the initial starting dose and will allow for understanding whether a slower, more gradual dosing regimen will result in improved tolerability and higher adherence rate to the target dose than a more condensed regimen that requires fewer up-titration steps.

4.2.1 Rationale for target study population

The current study will include subjects with symptomatic HF (NYHA class II-III) with LVEF < 50%, i.e., HFrEF and HFmrEF subjects, who are being treated with guideline-directed therapy. The percentage of patients with LVEF > 40% will be limited to approximately 25% of the total sample, which is in line with the reported relative prevalence of HFrEF and HFmrEF (McDonagh et al 2021). This population was chosen because it is a population that continues to have a projected unacceptably high rate of morbidity and mortality despite the latest approved treatment modalities.

Despite recently introduced recommendations for a quadruple pharmacological therapy for the treatment of HFrEF, including ARNI, beta-blockers, MRA and SGLT2 inhibitors (McDonagh et al 2021, Heidenreich et al 2022), outcomes on this comprehensive therapy are projected to leave significant room for improvement (Vaduganathan et al 2020, Tromp et al 2022). Thus, there remains an unmet need for further therapies improving mortality and morbidity in patients with HFrEF.

Treatment options for HFmrEF patients are less well-studied and, until relatively recently, recommendations for treatment of patients with HFmrEF were mostly based on post-hoc and subset analyses from HFrEF and HFpEF trials including patients with HFmrEF (Heidenreich et al 2022). More recently, the approved labels of the ARNI sacubitril/valsartan as well as of the SGLT2 inhibitors dapagliflozin and empagliflozin were expanded to include treatment of HFmrEF patients in some regions of the world. Nonetheless, a need for identifying novel treatments that will lower morbidity and mortality in HFmrEF patients remains unmet. Given that medications thought to be beneficial in HFrEF tend to also be beneficial in HFmrEF, it is deemed appropriate to study XXB750 in a combined sample of HFrEF and HFmrEF patients. Moreover, given that sacubitril/valsartan and XXB750 are both NP modulators, albeit via differing mechanisms with differing potencies, it is reasonable to

expect that XXB750 may also provide benefit in HFmrEF patients. This provides a strong rationale for the choice of studying the effects of XXB750 in patients with LVEF up to 50%.

The study will include patients who have been already treated with guideline-direct HF therapy so that the incremental benefit of the investigational treatment XXB750 will be clearly measured to better project the extent of improvements it can provide on top of the current standard of care. All eligible participants must be receiving an anti-RAS agent, i.e., an ACEI, ARB, or the ARNI sacubitril/valsartan. Participants should also be receiving beta blockers MRAs and SGLT2 inhibitors, unless contraindicated, not tolerated, not available to the patient or not recommended per local practice standards.

4.2.2 Rationale for the primary endpoint

The primary endpoint of this study is the change in log NT-proBNP from baseline to Week 16.

Aggregate clinical data has demonstrated that NT-proBNP not only provides value for the diagnosis, and helps the management of patients with HF, but also predicts clinical outcome (mortality and morbidity). Studies suggest that NT-proBNP levels strongly correlate with adverse clinical outcomes in HF patients including death and hospital admission for chronic HF (Zile et al 2016, Januzzi et al 2021, Schmitt et al 2021). More importantly, changes in natriuretic peptides were significantly associated with treatment effects on HF hospitalization across contemporary HF trials evaluating RAS inhibition (Vaduganathan et al 2018). With regard to ARNI, in a landmark analysis based on the PARADIGM-HF trial data, adjusted for baseline values, a 50% reduction in NT-proBNP levels was associated with approximately 32% reduction in the composite of CV death or HF hospitalization (Zile et al 2016).

In view of XXB750's mode of action, change of NT-proBNP is thus considered an appropriate surrogate endpoint for clinical outcomes of HF in this short-term study in patients with HF. The results will be used to determine the projected benefit of XXB750 on long-term HF outcomes when added to SoC guideline-directed HF therapy.

4.3 Justification for dose

Doses and administration regimens of XXB750 were selected based on the safety, tolerability, and pharmacodynamic profile observed in first interpretable results (FIR) from the FIH trial of XXB750 (study CXXB750A02101) and data observed in a phase 1b study in patients with HFrEF/HFmrEF (study CXXB750A12101).

Study CXXB750A02101 investigated the safety, tolerability, PK, and pharmacodynamics of single SC doses of XXB750 ranging from 1 mg to 600 mg in healthy volunteers without (1 mg, 3 mg, 10 mg, 30 mg, 60 mg, 120 mg, and 240 mg) and with elevated blood pressure (450 mg and 600 mg). The highest single tolerated dose was found to be 450 mg. There was no observed distinguishable effect on plasma cGMP or BP at doses of 1 mg, 3 mg, and 10 mg and placebo injection. XXB750 showed cGMP elevation and BP lowering effect at doses of ≥ 30 mg, which appeared to be greater than the effect of a placebo injection. For these doses, plasma cGMP elevation reached its maximum at day 2 post-dose, while it was lower than peak effect but still elevated compared to placebo at day 28 post-dose. Blood pressure lowering achieved maximum effect by day 3 and showed a gradual decline over the following weeks. At day 28 post-dose, the BP lowering effect was still present with doses ≥ 120 mg (SBP change from baseline of

~7 mmHg and ~5 mmHg for 120 mg and 240 mg dose cohorts, respectively). In the phase 1b study in HF patients with LVEF <50% receiving ACEI/ARBs and other background HF medications (cohort 1), a single XXB750 SC dose of 120 mg resulted in a maximum decrease in SBP of 7 mmHg, which occurred on day 2 post-dosing and was found to be well tolerated. Please refer to the XXB750 investigators brochure for detailed data on studies CXXB750A02101 and CXXB750A12101).

Given the sustained cGMP elevation and acceptable safety and tolerability profile, the 120 mg dose has been selected as the highest starting dose to be tested in this study. Based on pharmacometric modeling of the data from study CXXB750A02101, XXB750 doses of 60 mg, 120 mg and 240 mg are selected to be evaluated as target dose levels, to further investigate efficacy and safety of XXB750 in HF patients, and to support its use in subsequent studies as a once every 4 weeks SC administration regimen.

In the open-label sacubitril/valsartan cohort the target dose will be 97/103 mg bid, which is the target dose shown to be effective in reducing morbidity and mortality in HFrEF patients (McMurray et al 2014). The effectiveness of this dose extends to HF patients with higher LVEF with the greatest benefit occurring in patients with LVEF below normal, including patients with HFmrEF (Solomon et al 2020).

For more information about studies CXXB750A02101 and CXXB750A12101, please refer to the XXB750 Investigator's Brochure.

4.3.1 Rationale for choice of background therapy

The current study aims that all participants are treated with guideline-directed heart failure therapy as recommended per recently published guidelines (McDonagh et al 2021, Heidenreich et al 2022). To reduce variability across the studied sample and to ensure patients are treated with reasonably effective doses of background anti-RAS therapy, eligible patients must be receiving approximately half the target dose of their pre-study ACEI/ARB/ARNI or higher per international treatment guidelines and locally approved labels.

Other components of guideline-directed therapies for HFrEF and HFmrEF are recommended as background therapy at individually optimized doses, unless not tolerated or not available to patients.

Overall, these requirements will ensure that participants are treated according to current guideline recommendations including sacubitril/valsartan or an ACEI/ARB.

To avoid an influence of dose adjustments on baseline NT-proBNP levels, all background heart failure therapy must be given at a stable dose for at least 4 weeks prior to screening.

4.4 Rationale for choice of control drugs (comparator/placebo) or combination drugs

The primary objective of this study is to evaluate the dose-response effect of three dose levels of XXB750 on lowering NT-proBNP when administered on top of standard of care therapy recommended for patients with HFrEF (McDonagh et al 2021, Heidenreich et al 2022). As such, a placebo is considered the appropriate control for achieving this objective. A placebo

comparator is also appropriate for evaluating the safety and tolerability of XXB750 when given at the three dose levels.

Another objective of this study is to assess the benefit of NPR1 agonism with XXB750 on reduction in NT-proBNP relative to neprilysin inhibition. Thus, sacubitril/valsartan, a compound that delivers neprilysin inhibition and angiotensin receptor blockade, is the only clinically available and appropriate control. To ensure this objective is properly achieved, a sufficient number of ACEI/ARB treated participants will be randomized to either receive XXB750 while continuing to take their ACEI/ARB (i.e., receive NPR1 agonism + anti-RAS treatment in treatment arms 2, 3, 4a and 4b) or stop their pre-study ACEI/ARB and be converted to sacubitril/valsartan (i.e., receive neprilysin inhibition + anti-RAS treatment in treatment arm 5).

4.5 Rationale for public health emergency mitigation procedures

During a public health emergency as declared by local or regional authorities e.g., pandemic, epidemic, or natural disaster, mitigation procedures to ensure participant safety and trial integrity may be implemented. Notification of the public health emergency as declared by local or regional authorities should be discussed among investigators and Novartis. All procedures adapted to the situation must be submitted, if required as per local regulations, through a protocol amendment for approval by local or regional Health Authorities and Ethics Committees prior to implementation of mitigation procedures.

4.6 Purpose and timing of interim analyses/design adaptations

Limited safety data is currently available for XXB750, and it is thus prudent to dose the medication cautiously in participants until more is learned about its side effects.

Staggered enrollment and the safety interim analysis

To help guard the safety of study participants, randomization will initially exclude the planned highest target dose of XXB750 (i.e., arms 4a and 4b) until an interim analysis is conducted to evaluate the safety of the lower two target doses and that a sufficient safety margin exists to allow patients to be dosed with the highest target dose arms.

The randomized participant sample will be divided into two groups. Group 1 will consist of the first approximately 300 participants who will be randomized to receive placebo, XXB750 target dose of 60 mg every 4 weeks, XXB750 target dose of 120 mg every 4 weeks, or sacubitril/valsartan at a target dose of 97/103 mg bid in a 1:1:2:1 ratio (see Table 4-2).

Group 2 will consist of the remaining approximately 420 participants who are planned to be randomized to receive placebo, XXB750 target dose of 60 mg q 4 weeks, XXB750 target dose of 120 mg q 4 weeks, XXB750 target dose of 240 mg q 4 weeks at a starting dose of 60 mg, XXB750 target dose of 240 mg at a starting dose of 120 mg, or sacubitril/valsartan 97/103 mg bid in a 2:2:2:3:3:2 ratio (see Table 4-2).

The DMC will conduct an early safety interim analysis of the data of Group 1 when approximately 85 participants have completed at least 9 weeks of randomized treatment to assess the safety profile of repeat doses of XXB750 up to 120 mg in the study participants.

Additionally, safety data from cohort 2 of the ongoing XXB750 phase 1b study CXXB750A12101 (i.e., safety profile of two XXB750 120 mg doses followed by one 240 mg dose all given 4 weeks apart and followed for 12 weeks after the last dose) will be reviewed to provide further insight into the tolerability of the planned 240 mg dose of XXB750 in HF patients. If the totality of available safety evidence is deemed acceptable by the DMC to allow for dosing of XXB750 at doses of 240 mg, enrollment of Group 2 will start once Group 1 enrollment is completed.

In addition to the planned interim safety analysis summarized above, there will be safety data reviews conducted regularly by the DMC approximately twice annually. Refer to Section 10.1.4 for more information about the role of the DMC in this study.

Table 4-2 Overview of the planned staggered enrollment strategy

Treatment arm	Target dose, up-titration scheme	Group 1 n	Group 2 n	Total n
1	Placebo	60	60	120
2	XXB750 60 mg	60	60	120
3	XXB750 120 mg	120	60	180
4a*	XXB750 240 mg, initial dose 60 mg	0	90	90
4b*	XXB750 240 mg, initial dose 120 mg	0	90	90
5	Sacubitril/valsartan 97/103 mg bid	60	60	120
Total		300	420	720

*Highest target XXB750 dose may potentially be changed to be 180 mg. Refer to the adaptive design features section below for details.

Adaptive design features

If at the DMC early safety review described above, it is deemed inappropriate or unadvisable to continue the study as planned, i.e., to allow patients to be randomized to as planned into groups 4a and 4b at a target dose of 240 mg every 4 weeks, one of the following adaptations to the design of the study may be implemented per the recommendation of the DMC:

1. Change the highest target dose in the study from the planned XXB750 240 mg every 4 weeks to 180 mg every 4 weeks.
   In Group 2, participants randomized to treatment arm 4a will receive XXB750 60 mg SC at the Randomization followed by XXB750 120 mg SC at Week 4 and XXB750 180 mg SC at Week 8 and Week 12. Participants randomized to treatment arm 4b will receive XXB750 120 mg SC at the Randomization followed by XXB750 180 mg SC at Week 4, Week 8, and Week 12. The planned dosing schedule of all other treatment arms will remain unmodified. The sample size allotted to all treatment arms will remain unchanged.
2. Eliminate treatment arm 4b (top dose either XXB750 240 mg or 180 mg every 4 weeks). In this case the highest target dose will be reached after a starting dose of 60 mg (treatment arm 4a). In Group 2, all participants previously allocated to treatment arm 4b will be reallocated to treatment 4a. Thus, the 420 participants in Group 2 will be randomized to receive placebo, XXB750 target dose of 60 mg every 4 weeks, XXB750 target dose of 120 mg every 4 weeks, XXB750 target dose of 240 mg (or 180 mg) every 4

weeks at a starting dose of 60 mg, or sacubitril/valsartan 97/103 mg bid in a 1:1:1:3:1 ratio.

3. Eliminate treatment arms 4a and 4b.
   In this case the highest target dose in the study will be XXB750 120 mg every 4 weeks (treatment arm 3) and 180 participants will be allocated to treatment arm 3 from Group 2 for a total/overall sample size of 300 participants in this arm. Thus, Group 2 will include 360 participants who will be randomized to receive placebo, XXB750 target dose of 60 mg every 4 weeks, XXB750 target dose of 120 mg every 4 weeks, or sacubitril/valsartan 97/103 mg bid in a 1:1:3:1 ratio. The planned dosing schedule of these treatment arms will remain unmodified. In this case the total sample size of the study will be approximately 660 participants.

Participating investigators will be informed promptly of any of the adaptations to the design of the study shown above that are to be implemented in consultation with the DMC, so that they can inform the participants and ethics committees/IRBs as required by local regulations. The sponsor will inform the local health authorities as per local regulations.

Administrative interim analysis informing other programs

An additional administrative interim analysis to inform other development programs and studies may be conducted when approximately 430 participants are randomized into the study (at least 40 participants in each of arms 1-4) and are followed for at least 16 weeks. The results of this administrative interim analysis will not lead to any changes in this study and its results will be kept blinded for anyone who is directly involved in the conduct of this study. More details about the DMC review process and how unblinding will be handled in the context of DMC reviews will be provided in the DMC charter.

4.7 End of study definition

The end of the study is defined as the date of the last visit of the last participant in the study globally.

Study completion is defined as when the last participant finishes his/her Week 16 visit and any repeat assessments associated with this study visit have been documented and followed-up appropriately by the Investigator.

All randomized participants will have a safety follow-up after last administration of study treatment as outlined in Section 1.3 SoA and Section 4.1.3. Serious adverse event (SAE) reporting continues during this time period as described in Section 8.6.3. Documentation of attempts to contact the participant are required to be recorded in the source documentation.

5 Study population

The study population will consist of male and female participants 18 years old or older with symptomatic HFrEF or HFmrEF as defined in the inclusion criteria. A total of approximately 720 participants will be randomized into this study. The percentage of patients with LVEF > 40% will be limited to approximately 25% of the total sample.

5.1 Inclusion criteria

Participants eligible for inclusion in this study must meet **all** of the following criteria:

1. Signed informed consent must be obtained prior to participation in the study.
2. Male and female outpatients who are ≥ 18 years old at screening.
3. Current symptom(s) of HF NYHA class II-III.
4. LVEF < 50% (any local measurement, made within the past 6 months prior to or during screening using echocardiography, MUGA, CT scanning, MRI or ventricular angiography is acceptable, provided there is no subsequent measurement ≥ 50%).
5. NT-proBNP ≥ 600 pg/mL if in sinus rhythm or
   NT-proBNP ≥ 900 pg/ml if in atrial fibrillation/flutter at screening.
6. Receiving an ACEI or an ARB at a stable dose of at least enalapril 10 mg/d or equivalent for at least 4 weeks before screening or receiving sacubitril/valsartan at a stable dose of at least 49/51 mg bid for at least 4 weeks before screening.
7. Receiving other guideline recommended HF therapies as deemed appropriate by the investigator and that are stable in dose for at least 4 weeks before screening, unless contraindicated, not tolerated, or not available to patient. Other evidence-based therapy for heart failure should also be considered, e.g., cardiac resynchronization therapy (CRT) and an implantable cardioverter-defibrillator in selected patients, as recommended by guidelines.

5.2 Exclusion criteria

Participants meeting **any** of the following criteria are **not** eligible for inclusion in this study.

1. Current acute decompensated HF (exacerbation of chronic HF manifested by signs and symptoms that may require intravenous therapy) or hospitalization for HF within 3 months prior to screening.
2. Current symptomatic hypotension (for example dizziness/presyncope).
3. Office systolic blood pressure (SBP) ≥ 180 mmHg or < 105 mmHg at screening or at randomization.
4. History of hypersensitivity to XXB750, sacubitril/valsartan, or any of the study drug's excipients or to any other biological drugs.
5. Received a monoclonal antibody or immunoglobulin-based agent within 1 year of screening.
6. In subjects with ACEI/ARB medication at screening, previous inability to tolerate any dose of sacubitril/valsartan or any ARB (as per the investigator's judgment).
7. Known history of angioedema (as per the investigator's judgment).
8. Serum potassium > 5.4 mmol/L (or equivalent plasma potassium value) at screening.
9. Estimated GFR < 30 mL/min/1.73m^2 as measured by the CKD-EPI formula at screening.
10. Presence of known functionally significant bilateral renal artery stenosis at screening.
11. Evidence of urinary obstruction, or difficulty in voiding at screening, or of congenital renal abnormalities with known effect on renal function.
12. Clinically significant congenital heart disease that could be the cause of the patient's symptoms and signs of HF.

13. Presence of hemodynamically significant valvular heart disease other than functional mitral insufficiency or known right ventricular dysfunction that is deemed to increase susceptibility to the hypotensive effects of therapy in the opinion of the investigator.
14. Clinically significant cardiac arrhythmias (e.g., ventricular tachycardia), high-grade atrio-ventricular (AV) block (e.g., Mobitz type II and third-degree AV block in the absence of a pacemaker) or symptomatic bradycardia within 6 months of screening according to the investigator's judgement.
15. Acute myocardial infarction (AMI) or unstable angina, any history of ischemic or hemorrhagic stroke, or any percutaneous coronary intervention (PCI) or coronary artery bypass graft (CABG) within 6 months of screening, or CRT implantation within 3 months of screening.
16. History or current diagnosis of severe pulmonary disease (e.g. COPD) requiring chronic supplemental oxygen, corticosteroid, or nebulizer therapy or pulmonary arterial hypertension (WHO group 1 or 4) requiring pharmacology treatment at screening.
17. At screening, symptomatic anemia or hemoglobin < 10 g/dL in male subjects or < 9 g/dL in female subjects.
18. Prior major organ transplant, intention to transplant (i.e. on transplant list) or planned/anticipated major cardiac surgery during the involvement of the participant in the study.
19. History or presence of any other disease where the life expectancy is less than 3 years.
20. History of malignancy of any organ system (other than localized basal or squamous cell carcinoma of the skin or localized prostate cancer), treated or untreated, within the past 3 years, regardless of whether there is evidence of local recurrence or metastases.
21. Evidence of hepatic disease as determined by any one of the following: aspartate aminotransferase (AST; serum glutamic-oxaloacetic transaminase, SGOT) or alanine aminotransferase (ALT; serum glutamic-pyruvic transaminase, SGPT) values exceeding 3x the upper limit of normal (ULN), or total bilirubin (TBL) > 1.5 mg/dL at screening.
22. History or current diagnosis of drug abuse or alcohol dependency.
23. Lacking the ability to comprehend or follow instructions, or for any reason in the opinion of the investigator, a patient that would be unlikely or unable to comply with study protocol.
24. Concurrent enrollment in any other investigational drug or device trial (participation in non-interventional registries is acceptable).
25. Use of other investigational drugs at the time of enrollment, or within 30 days or 5 half-lives of enrollment, whichever is longer.
26. Pregnant, nursing or planning to become pregnant (documented negative pregnancy test required within a maximum of 7 days prior to enrollment of all women of childbearing potential). Documentation of highly effective contraception is also required for women of childbearing potential (see below).

    Women of child-bearing potential are defined as all women physiologically capable of becoming pregnant, **unless** they are using highly effective methods of contraception while taking study treatment and for 3 months after last double-blind injectable study treatment

or for 7 days after the last dose of oral study treatment (if randomized to receive open-label sacubitril/valsartan). Highly effective contraception methods include:

- Total abstinence (when this is in line with the preferred and usual lifestyle of the participant). Periodic abstinence (e.g. calendar, ovulation, symptom-thermal, post-ovulation methods) and withdrawal are not acceptable methods of contraception.
- Female sterilization (have had surgical bilateral oophorectomy with or without hysterectomy, total hysterectomy, bilateral salpingectomy, or bilateral tubal ligation) at least six weeks before taking study treatment. In case of oophorectomy alone, only when the reproductive status of the woman has been confirmed by follow-up hormone level assessment.
- Male sterilization (vasectomy) of the male partner(s) of the female participant (at least 6 months prior to screening). For female participants on the study, the vasectomized male partner(s) should be the sole partner(s) for that participant.
- Use of oral, (estrogen and progesterone), injected, or implanted hormonal methods of contraception or placement of an intrauterine device (IUD) or intrauterine system (IUS), or other forms of hormonal contraception that have comparable efficacy (failure rate < 1%), for example hormone vaginal ring or transdermal hormone contraception.

In case of use of oral contraception, women should have been stable on the same pill for a minimum of 3 months before taking study treatment.

Women are considered post-menopausal if they have had 12 months of natural (spontaneous) amenorrhea with an appropriate clinical profile (e.g., age-appropriate history of vasomotor symptoms). Women are considered not of childbearing potential if they are post-menopausal or have had surgical bilateral oophorectomy (with or without hysterectomy), total hysterectomy, bilateral salpingectomy or bilateral tubal ligation at least six weeks before enrollment on study. In the case of oophorectomy alone, only when the reproductive status of the woman has been confirmed by follow up hormone level assessment is not she considered to be of childbearing potential.

If local regulations deviate from the contraception methods listed above to prevent pregnancy, local regulations apply and will be described in the ICF.

5.3 Screen failures

A screen failure occurs when a participant who consents to participate in the clinical study is subsequently found to be ineligible and therefore does not enter the Randomized treatment period of the study. A minimal set of screen failure information is required to ensure transparent reporting of screen failure participants to meet the Consolidated Standards of Reporting Trials (CONSORT) publishing requirements and to respond to queries from regulatory authorities. Minimal information includes demography, reason(s) for screen failure, eligibility criteria, adverse events related to AxMPs and any SAE.

The reason for screen failure should be recorded on the appropriate CRF. The demographic information, informed consent, and inclusion/exclusion pages must also be completed for screen failure participants. Screening laboratory assessments will also be collected and entered into the clinical database. No other data will be entered into the clinical database for participants who are screen failures, unless the participant experienced a serious adverse event during the

screening period (see Section 8.6.3 for SAE reporting details). If the participant fails to be randomized, the IWRT (Interactive Web Response Technology)/IRT (Interactive Response Technology) system must be updated with the screen fail that the participant will not be randomized. Data and samples collected from participants prior to screen failure may still be analyzed.

Individuals who do not meet the criteria for participation in this study (screen failure), will have one opportunity to be re-screened again for this study, ≥ 2 weeks after the original screening date.

5.3.1 Replacement policy

Not applicable.

5.3.2 Participant numbering

Each participant is identified in the study by a Participant Number (Participant No.), that is assigned when the participant is enrolled for screening and is retained for the participant throughout his/her participation in the trial. The Participant No. consists of the Site Number (Site No.) (as assigned by Novartis to the investigative site) with a sequential participant number suffixed to it, so that each participant's participation is numbered uniquely across the entire database. Upon signing the informed consent form, the participant is assigned to the next sequential Participant No. available.

A new ICF must be signed if the investigator chooses to re-screen the participant after a participant has screen failed, and the participant will be assigned a new Participant Number. Additional details regarding Screening are provided in Section 8.1.

6 Study treatment(s) and concomitant therapy

6.1 Study treatment(s)

Study treatment includes investigational drugs XXB750, its matching placebo, and sacubitril/valsartan.

Oral study medication refers to sacubitril/valsartan as investigational/control drug.

XXB750 or matching placebo will be administered as a SC injection every 4 weeks during the randomized treatment period (Randomization, Week 4, Week 8 and Week 12). Table 6-1 lists investigational and control drug.

Table 6-1 Investigational and control drug

Treatment Title	XXB750 150 mg/mL	XXB750 0 mg/mL (placebo)	Sacubitril/valsartan
Treatment Description	Liquid in Vial	Liquid in Vial	Solid
Type	Biologic	Placebo	Drug
Dose Formulation	Concentrate for solution for injection	Solution for injection	Tablet
Unit Dose Strength(s)	150 mg/ml	0 mg/ml	97/103 mg, 49/51 mg and 24/26 mg

**Dosage Level(s)**	as per Table 6-3	as per Table 6-3	as per Table 6-3
**Route of Administration**	Subcutaneous use	Subcutaneous use	Oral
**Use**	Experimental	Placebo	Active comparator
**IMP**	Yes	Yes	Yes
**Sourcing**	Novartis (global)	Novartis (global)	Provided locally
**Packaging and Labeling**	Study treatment will be provided in vials. Each vial will be labeled as per country requirement. Open-label packs	Study treatment will be provided in vials. Each vial will be labeled as per country requirement. Open-label packs	As per local packaging. Each package will be labeled as per country requirement. Open-label packs

6.1.1 Additional study treatments

Auxiliary medicinal products (under EU CTR) in this trial are ACEI, ARB and sacubitril/valsartan. When participants enter the randomized treatment period, they will remain on the same background ACEI or ARB or on sacubitril/valsartan (as applicable) they had been receiving for their usual care before randomization without change in dose, source, or type of ACEI/ARB/ARNI (per local requirements). Exception is made to those participants that are randomized to arm 5, who will be switched from their pre-study ACEI or ARB to open-label sacubitril/valsartan, which will be provided by Novartis during the 16 weeks randomized treatment period.

Table 6-2 Auxiliary treatments

Treatment Title	ACEI	ARB	Sacubitril/valsartan
**Treatment Description**	Once or twice daily	Once or twice daily	Twice daily
**Type**	Drug	Drug	Drug
**Dose Formulation**	Tablet	Tablet	Tablet
**Route of Administration**	Oral	Oral	Oral
**Use**	Background medication	Background medication	Background medication
**Authorization status of the AMP in EEA**	Yes	Yes	Yes
**Sourcing**	Provided locally as per country requirement	Provided locally as per country requirement	Provided locally as per country requirement
**Packaging and Labeling**	Auxiliary treatment will be provided in commercially available packaging.	Auxiliary treatment will be provided in commercially available packaging.	Auxiliary treatment will be provided in commercially available packaging.

6.1.2 Treatment arms/group

Table 6-3 summarizes planned study treatment arms and their dosing schedule. Please refer to Section 4.6 for potential adaptive design features that may potentially affect the treatment arms at the DMC early safety review.

Table 6-3 Treatment arms, dose levels, and planned doses administered at dispensing visits during the Randomized treatment period

Treatment arm	Target dose level	Titration scheme	Rando-mization	Week 2	Week 4	Week 8	Week 12
**Arm 1**	**XXB750 placebo**		Placebo		Placebo	Placebo	Placebo
**Arm 2**	**XXB750 60 mg**		60 mg		60 mg	60 mg	60 mg
**Arm 3**	**XXB750 120 mg**		60 mg		120 mg	120 mg	120 mg
**Arm 4**	**XXB750 240 mg***	**4a**	60 mg		120 mg	240 mg*	240 mg*
		**4b**	120 mg		240 mg*		
**Arm 5**	**Sacubitril/valsartan open-label, 97/103 mg bid**	Starting dose as per label and investigator's judgment	49/51 mg bid	49/51 mg bid	97/103 mg bid	97/103 mg bid	97/103 mg bid
			24/26 mg bid				

* May be lowered to a target dose of 180 mg, determined after interim safety analysis

In treatment arms 1 through 4, all patients will receive SC injections of XXB750 or matching placebo. Refer to Section 6.5 for instructions on adjusting the dose of XXB750 in the context of management of adverse events.

In treatment arm 5, restricted to patients on ACEI/ARB, the pre-study ACEI/ARB medication will be discontinued and replaced by open-label sacubitril/valsartan. Participants on pre-study ACEIs must be instructed to observe an ACEI-free washout wash-out period of 36 hours is mandatory prior to the first dose of open-label sacubitril/valsartan to minimize the potential risk of angioedema. Refer to Section 4.1.2 for a description of how to implement the ACEI-free washout.

Patients randomized to open-label sacubitril/valsartan may be initiated on 24/26 mg to 49/51 mg bid per the investigator's clinical judgment (taking into account the pre-study ACEI/ARB dose) and must be gradually force-titrated to the target dose of 97/103 mg bid by Week 4. Every attempt should be made to maximize the dose of the study open-label sacubitril/valsartan by modifying other background medications, such as diuretics, nitrates, and calcium channel blockers, as per the investigator's clinical judgment. The dose of open-label sacubitril/valsartan may be down-titrated if the participant experiences dose-limiting side effects that cannot be managed by modifications of the patient's concomitant medications.

6.1.3 Treatment duration

The planned duration of randomized treatment is 16 weeks. After the 16-week randomized treatment period, participants will enter an 8-week safety follow-up period in which participants may be treated at the discretion of the investigator taking into account that their heart failure clinical status may still be affected by the study treatment for some time after its discontinuation.

6.2 Preparation, handling, storage, and accountability

Each study site will be supplied with study treatment as described under Section 6.1 in Table 6-1 (investigational and control drugs).

A unique medication number is printed on the study medication label (applicable only for globally sourced investigational and control drug, i.e. XXB750 or matching placebo).

Unblinded qualified and properly trained members of the study team at the site will identify the IMP kits to be administered or be dispensed to the participants by contacting the IRT system and obtaining the medication number(s). Drug accountability and reconciliation data are recorded in the IRT system.

For locally sourced open-label oral study medication, according to the treatment assigned to the participant, Investigator staff will select the study medication to be dispensed to the participant. Site personnel will record the locally sourced supply dispensation into the participant's source document.

As per Section 4.5, during a public health emergency as declared by local or regional authorities i.e., pandemic, epidemic or natural disaster, that limits or prevents on-site study visits, delivery of injectable IMP directly to a participant's home is not possible because XXB750 is to be administered subcutaneously by a trained and certified personnel or nurse, and because other essential assessments and PK sampling etc. requires the patient to be on site.

Supply, preparation, dispensation and return of IMP will be described in the Pharmacy Manual.

6.2.1 Handling of study treatment

Study treatment must be received by the unblinded person at the study site, handled and stored safely and properly and kept in a secured location which only designated site personnel have access to. Upon receipt, all study treatment must be stored according to the instructions specified in the product information(s) and in the Investigator's Brochure and in accordance with Good Manufacturing Practice (GMP) and Good Clinical Practice (GCP) procedures.

Clinical supplies are to be dispensed only in accordance with the protocol. Technical complaints are to be reported to the respective Novartis Country Organization Quality Assurance.

The subcutaneous study treatment of XXB750 or respective placebo needs to be prepared by unblinded qualified site personnel (in accordance with local regulations, e.g. pharmacist) in order to ensure treatment blinding. Details about handling, storage and administration will be provided in a separate Pharmacy Manual.

Medication labels will be in the local language and comply with the legal requirements of each country. They will include storage conditions for the study treatment but no information about the participant except for the medication number.

The designated site staff must maintain an accurate record of the shipment and dispensing of IMP in the IRT drug accountability log. Monitoring of drug accountability will be performed by field monitors during site or remote monitoring visits, and at the completion of the trial. The investigator must provide accountability also for locally sourced study materials/treatment used for administration (e.g. i.v. syringes).

The site may destroy and document destruction of unused study treatment, drug labels and packaging, as appropriate in compliance with site processes, monitoring processes, and per local regulation/guidelines. Otherwise, the investigator will return all unused study treatment, packaging, drug labels, and a copy of the completed drug accountability log to the Novartis monitor or to the Novartis address provided in the investigator folder at each site.

Oral study medication (i.e. open-label sacubitril/valsartan as active comparator) will be dispensed in an open-label fashion at scheduled site visits. Participants will be asked to return all unused oral study medication and packaging at each site visit or at the time of discontinuation of oral study medication. Site personnel will record the locally sourced supply dispensation into the participant's source document.

6.2.2 Handling of other treatment

The participant's use of other medications will be monitored (dose, dose changes and reasons for changes) throughout the whole duration of the study by documentation of all medications at each visit.

6.2.3 Instruction for prescribing and taking study treatment

All study medications will be administered in addition to other background medications the participant is taking, including other background HF medications.

Table 6-3 shows how the study treatments should be prescribed at each dispensing visit during the randomized treatment period. Please refer to Section 6.5 for dose modification algorithm to manage adverse events that may be contributed to the injectable study medication.

All kits of study treatment assigned by IRT will be recorded in the IRT system.

XXB750 Injectable Study Medication

Participants in treatment arms 1-4 will receive an SC injection of XXB750/matching placebo according to the participant's randomized treatment assignment. The unblinded, qualified site personnel will prepare the study drug for injection in a blinded way according to the Pharmacy Manual; qualified blinded study staff will inject the study drug subcutaneously in the upper arm or abdomen. Injections should not be given into areas of active skin disease or injury such as sunburns, skin rashes, inflammation, or skin infections. After injection, the participant will be observed for potential hypersensitivity reactions for ≥20 minutes, before he/she can leave the site.

Only the unblinded qualified site personnel in charge of preparing the study medication will be aware of the contents of the administered injectable study medication. Refer to Section 6.2.1 for more details regarding the preparation of the study medications for dispensing.

Oral Study Medication

At every dispensing visit during the randomized treatment period, the open-label sacubitril/valsartan oral study medication will be dispensed to participants in treatment arm 5 in sufficient quantities to last until the next scheduled visit (Section 6.1.2, Table 6-1, and Table 6-3).

- Participants should be instructed to take the oral open-label sacubitril/valsartan study medication twice daily at approximately the same times of each day except for study site visit days. On these days, participants should bring their oral study medication to the site and take it after blood sampling for this visit has been performed.

- Participants should take the oral study medication without regard to food. Each dose may be taken with a glass of water.
- If vomiting occurs during the course of treatment, participants should not take the oral study treatment again before the next scheduled dose.
- If the patient misses taking any study drug dose, he/she should take it as soon as possible, unless if it is almost time for the following scheduled dose. In this case, the patient should skip the missed dose and return back to his/her regular study drug administration schedule.

6.3 Measures to minimize bias: randomization and blinding

6.3.1 Treatment assignment, randomization

At the randomization visit, all eligible participants will be randomized via IRT to one of the treatment arms (see Section 1.2 and Table 6-3). The investigator or his/her delegate will contact the IRT after confirming that the participant fulfills all the inclusion/exclusion criteria. The IRT will assign a randomization number to the participant, which will be used to link the participant to a treatment arm and will specify a unique medication number for the injectable product to be dispensed to the participant.

The randomization numbers will be generated using the following procedure to ensure that treatment assignment is unbiased and concealed from participants and investigator staff. A participant randomization list will be produced by the IRT provider using a validated system that automates the random assignment of participant numbers to randomization numbers. These randomization numbers are linked to the different treatment arms, which in turn are linked to medication numbers. A separate medication list will be produced by or under the responsibility of Novartis Global Clinical Supply (GCS) using a validated system that automates the random assignment of medication numbers to packs containing the investigational product.

Randomization will be stratified by region, and ACEI/ARB or sacubitril/valsartan background medication, respectively. Stratification will serve to prevent a potential imbalance of regions and ACEI/ARB or sacubitril/valsartan background medication, across the arms. In addition, stratified randomization will make sure that arms 1-4 will have 1:2 ratio of participants on ACEI/ARB or sacubitril/valsartan background medication, respectively, and arm 5 will include only participants on prior ACEI/ARB background medication. The randomization scheme for participants will be reviewed and approved by a member of the Randomization Office.

6.3.2 Treatment blinding

Participants, investigator staff, persons performing the assessments, and the Novartis Clinical Trial Team (CTT) will remain blinded to the identity of the treatment from the time of randomization until database lock.

The following methods will be used to maintain the blind:

1. Randomization data will be kept strictly confidential until the time of final database lock and will not be accessible by anyone else involved in the study with the following exceptions:

- Unblinded staff (i.e. pharmacist/nurse) or other designated qualified site personnel who prepare the double-blind study medication doses at the study site and unblinded CRA monitoring the sites who will monitor study medication preparation at the site.
- The bioanalyst for PK analyses (to avoid the unnecessary analysis of samples belonging to participants randomized to placebo treatment)
- The DMC and an external independent analysis team who need to prepare efficacy interim and safety monitoring analysis reports for the DMC. For details on the DMC, please refer to Section 10.1.4.1.

These personnel will not be involved in any other trial activities.

2. The identity of the treatments will be concealed by the use of study treatments that are all identical in packaging, labeling, schedule of administration, and appearance. All XXB750/matching placebo injections will be prepared such that they will all have the same injection volumes regardless of dose to maintain the blinding. Blinded label supply (also mention respective drug name) will be provided at sites to the unblinded site pharmacist to prepare study drug and to conceal treatment code from participants and the investigator staff administering the study injections/dispensing the study medications and performing the assessments.
3. Data with unblinding potential, such as PK concentrations and immunogenicity data collected after the randomization visit, will be kept blinded to all personnel included in study conduct activities until the time of final database lock.
4. Sponsor staff responsible for decision making at the clinical program development level (the Novartis decision making team) will receive the aggregated unblinded results at the treatment group level at the time of the administrative interim analysis informing other programs, as outlined in Table 6-4. The team will not have access to the individual participant treatment codes.

The DMC will review unblinded data reports created by an external independent analysis team. More details about the DMC review process and how unblinding will be handled in the context of DMC reviews will be provided in the DMC charter.

Table 6-4 Blinding and unblinding plan

Role	Time or Event					
	**Randomization list generated**	**Treatment allocation & dosing**	**Safety event (single subject unblinded)**	**Safety monitoring review**	**IA for safety**	**Administrative IA to inform other programs**
Participants	B	B	UI	B	B	B
Site staff	B	B	UI	B	B	B
Unblinded site personnel involved in dose preparation	B	UI	B	B	B	B
Global Clinical Supply	UG/UI	UG/UI	UG/UI	UG/UI	B	B
Randomization Office	UI	UI	UI	UI	UI	UI

Role	Time or Event					
	**Randomization list generated**	**Treatment allocation & dosing**	**Safety event (single subject unblinded)**	**Safety monitoring review**	**IA for safety**	**Administrative IA to inform other programs**
Unblinded Sponsor staff, e.g., for study treatment re-supply, unblinded monitor(s), sample analyst(s)	UG	UI	B	UG	B	B
Unblinded Pharmacovigilance Sponsor staff	B	B	UI	B	B	B
DMC	UI	UI	UI	UI	UI	UI
External independent Statistician/statistical programmer/ data analysts	UI	UI	UI	UI	UI	UI
Biomarker analyst	B	B	B	B	B	B
PK analyst	UI	UI	B	UI	B	B
Sponsor CTT	B	B	B	B	B	B
Novartis decision making team for IAs	B	B	B	B	B	UG
All other Sponsor staff not identified above (i.e. management, support functions)	B	B	B	B	B	B

B: Complete blinded

UG: Unblinded at the group level (i.e. has access to unblinded group level summary results, but not to the individual participant treatment codes)

UI: Unblinded to individual participant treatment codes

6.3.3 Emergency breaking of assigned treatment code

Emergency code breaks must only be undertaken when it is required to in order to treat the participant safely.

Most often, discontinuation from study treatment and knowledge of the possible treatment assignments are sufficient to treat a study participant who presents with an emergency condition. Emergency treatment code breaks are performed using the IRT. When the Investigator contacts the system to break a treatment code for a participant, he/she must provide the requested participant identifying information and confirm the necessity to break the treatment code for the participant. The Investigator will then receive details of the investigational drug treatment for the specified participant and a fax or email confirming this information. The system will automatically inform the Novartis monitor for the site and the study team that the code has been broken.

It is the Investigator's responsibility to ensure that there is a dependable procedure in place to allow access to the IRT/code break cards at any time in case of emergency. The Investigator will provide:

- protocol number
- participant number

In addition, oral and written information to the participant must be provided on how to contact his/her backup in cases of emergency, or when he/she is unavailable, to ensure that un-blinding can be performed at any time.

In case of emergency unblinding, the participants should be discontinued from study treatment and continue with the study visit as scheduled without study medication administration.

6.4 Study treatment compliance

Injections of XXB750 or matching placebo will be administered at the site. All study treatment administered, dispensed and returned must be recorded in the Drug Accountability and Returns Management functionality in the IRT system. The dispensed and returned data recorded electronically in the IRT database will be considered the source record.

For participants randomized to arm 5, the investigator must promote adherence to treatment by instructing the participant to take the study medication exactly as prescribed and by stating that compliance is necessary for the participant's safety and the validity of the study. The participant must also be instructed to contact the investigator if he/she is unable for any reason to take the study treatment as prescribed. Compliance will be assessed by the investigator and/or study personnel at each visit using information provided by the participant. This information should be captured in the source document at each visit.

Pharmacokinetic parameters (measures of treatment exposure) will be determined in all participants treated with XXB750, as detailed in Section 8.7.

6.4.1 Recommended treatment of adverse events

Recommendations for handling liver events are described in Appendix 5.

Recommendations for handling potential drug induced liver injury (DILI) cases are described in Section 6.5.2.1.

Recommendations for handling hypotension are described in Appendix 7.

Recommendations for handling hyperkalemia are described in Appendix 8.

Recommendations for handling renal events are described in Section 10.6.1.

Medication used to treat adverse events (AEs) must be recorded on the appropriate CRF.

6.5 Dose modification

For participants who do not tolerate the protocol-specified dosing schedule, dose adaptations are recommended in order to allow participants to continue the study treatment.

In general, every effort should be made to resolve AEs that may be attributed to study medication and/or foundational background HF treatments before the next dose of study

medication can be administered. Detailed recommendations for dose modifications of concomitant non-HF treatments, diuretics, and foundational background HF treatments (i.e., ACEI/ARB, sacubitril/valsartan, MRA, SGLT2 inhibitor and/or beta-blocker) in case of hypotension or hyperkalemia are provided in Appendix 7 (Section 10.7, Figure 10-1) and Appendix 8 (Section 10.8), respectively.

For dosing of the injectable study medication, the patient should be evaluated at the scheduled study visit when the study medication is due to be administered. The flow chart in Figure 6-1 shows how to evaluate the participant based on the following principles:

- If by the next dosing visit the participant's foundational background HF treatments are fully resumed at pre-study doses, the injectable study medication will be administered as planned.
- If the dose of at least one foundational background HF treatment is still required to be reduced relative to the pre-study dose, the injectable study medication will be administered at the same dose as at the previous visit (i.e., no dose escalation).
- If one of the foundational background HF treatments had been stopped and cannot be resumed due to the AE not being resolved, the injectable study medication dose should be skipped and administered at the next dosing visit at the same dose level as previously administered to the participant if the patient has been able to resume taking the foundational background HF treatment previously stopped.

Figure 6-1 Modification of injectable study medication

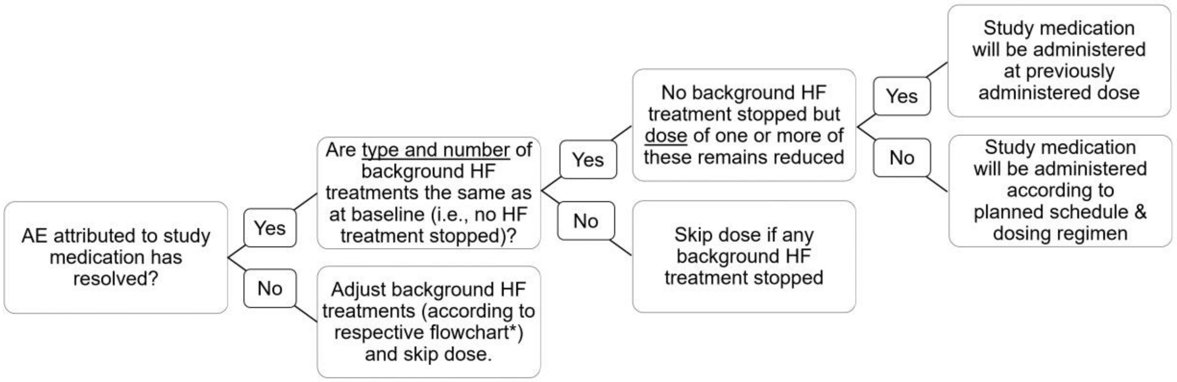

*Figure 10-1

Evaluate foundational background HF treatments (i.e., ACEI/ARB/ARNI, MRA, SGLT2i, beta blocker) relative to baseline with respect to type of drug and dose. Adverse events, especially events indicative of symptomatic hypotension, should be evaluated and potentially require action as outlined in the respective appendix. For the evaluation of planned study drug administration, only AEs related to study drug or foundational background HF treatments should be considered.

Safety criteria determining dose interruption or discontinuation based on liver or renal events are listed in Appendix 5 (Section 10.5) and Appendix 6 (Section 10.6), respectively.

All dose changes must be recorded on the appropriate CRF.

6.5.1 Definitions of dose limiting toxicities (DLTs)

Not applicable.

6.5.2 Follow-up for toxicities

Refer to Appendix 5 (Section 10.5) for the follow-up of liver events and Appendix 6 (Section 10.6) for the follow-up of renal events.

6.5.2.1 Follow up on potential drug-induced liver injury (DILI) cases

Transaminase increases combined with total bilirubin increases may be indicative of potentially severe DILI and should be considered as clinically important events and assessed appropriately to establish the diagnosis. The required clinical information, as detailed below, should be sought to obtain the medical diagnosis of the most likely cause of the observed laboratory abnormalities.

The threshold for potential DILI may depend on the participant's baseline aspartate aminotransferase (AST), alanine aminotransferase (ALT) and total bilirubin (TBL) values; participants meeting any of the following criteria will require further follow-up as outlined below:

- For participants with normal AST and ALT and TBL values at baseline: AST or ALT > 3.0 x ULN combined with TBL > 2.0 x ULN
- For participants with elevated AST or ALT or TBL values at baseline: [AST or ALT > 2 x baseline level] OR [AST or ALT > 300 U/L] (whichever occurs first) combined with [TBL > 2 x baseline level AND > 2.0 x ULN]

As DILI is essentially a diagnosis of exclusion, other causes of abnormal liver tests should be considered and their role clarified before DILI is assumed to be the cause of liver injury.

A detailed history, including relevant information such as review of ethanol consumption, concomitant medications, herbal remedies, supplement consumption, history of any pre-existing liver conditions or risk factors, should be collected.

Laboratory tests should include AST, ALT, TBL, direct and indirect bilirubin, gamma-glutamyl transferase (GGT), prothrombin time (PT)/international normalized ratio (INR), alkaline phosphatase, albumin, and creatine kinase. If available, testing of glutamate dehydrogenase (GLDH) is additionally recommended. Refer to Section 10.5.1 for further guidance.

Perform relevant examinations (ultrasound or MRI, endoscopic retrograde cholangiopancreatography [ERCP]) as appropriate, to rule out an extrahepatic cause of cholestasis.

Table 6-5 provides guidance on specific clinical and diagnostic assessments which can be performed to rule out possible alternative causes of observed liver function test (LFT) abnormalities.

Table 6-5 Diagnostic assessments to determine alternative causes of observed LFT abnormalities

Disease	Assessment
Hepatitis A, B, C, E	• IgM anti-HAV; HBsAg, IgM & IgG anti-HBc, HBV DNA; anti-HCV, HCV RNA, IgM & IgG anti-HEV, HEV RNA
CMV, HSV, EBV infection	• IgM & IgG anti-CMV, IgM & IgG anti-HSV; IgM & IgG anti-EBV

Disease	Assessment
Autoimmune hepatitis	● Antinuclear Antibodies (ANA) & Anti-Smooth Muscle Antibody (ASMA) titers, total IgM, IgG, IgE, IgA
Alcoholic hepatitis	● Ethanol history, GGT, MCV, CD-transferrin
Nonalcoholic steatohepatitis	● Ultrasound or MRI
Hypoxic/ischemic hepatopathy	● Medical history: acute or chronic congestive heart failure, hypotension, hypoxia, hepatic venous occlusion. Ultrasound or MRI
Biliary tract disease	● Ultrasound or MRI, ERCP as appropriate
Wilson disease (if < 40 years old)	● Cæruloplasmin
Hemochromatosis	● Ferritin, transferrin
Alpha-1-antitrypsin deficiency	● Alpha-1-antitrypsin

MCV: mean corpuscular volume; ERCP: Endoscopic retrograde cholangiopancreatography; HAV: hepatitis A virus; HBV: hepatitis B virus; HCV: hepatitis C virus; HEV: hepatitis E virus; CMV: cytomegalovirus; EBV: Epstein Barr virus; CD-transferrin: carbohydrate-deficient transferrin; MRI: magnetic resonance imaging; HBsAg: Hepatitis B virus surface antigen; HSV: Herpes simplex virus

Other causes should also be considered based upon participants' medical history such as hyperthyroidism (causing thyrotoxic hepatitis where T3, T4 and thyroid-stimulating hormone [TSH] should be tested), CV disease (causing ischemic hepatitis where an ECG could be performed, and the history of prior hypotensive episodes should be inquired), and Type 1 diabetes mellitus (causing glycogenic hepatitis).

Obtain PK sample to determine exposure to study treatment and metabolites.

Following the appropriate causality assessments, as outlined above, the causality of the treatment is estimated as "probable" (i.e. > 50% likely), if it appears greater than all other possible causes of liver injury combined. The term "treatment-induced" indicates probably caused by the treatment, not by something else, and only such a case can be considered a DILI case and should be reported as an SAE.

All cases confirmed by repeat testing meeting the laboratory criteria defined above, with no other alternative cause for LFT abnormalities identified, should be considered as "medically significant" and thus, meet the definition of SAE and should be reported as a SAE using the term "potential treatment-induced liver injury." All events should be followed-up with the outcome clearly documented.

6.6 Continued access to study treatment after the end of the study

After Week 16, regular standard of care for heart failure should be provided to the participant by the investigator and/or referring physician as per the local standard of care and applicable guidelines.

6.6.1 Post trial access

No post-trial access program is planned for this trial. Participants who complete or prematurely discontinue participation in this trial will switch to standard of care treatment.

6.7 Treatment of overdose

In the event of an overdose, the investigator should:

- Contact the medical monitor immediately.
- Evaluate the participant to determine, in consultation with the medical monitor, whether study treatment should be interrupted or whether the dose should be reduced.
- Closely monitor the participant for any AE/SAE and laboratory abnormalities as required according to the pharmacodynamic and pharmacokinetic characteristics of the drug concerned.
- Obtain a plasma sample for PK analysis within 6 days from the date of the last dose of injectable study treatment if requested by the medical monitor (determined on a case-by-case basis).
- Document the quantity of the excess dose as well as the duration of the overdose.
- Follow the recommendations of the approved product label about overdose in case this involves sacubitril/valsartan study medication.

6.7.1 Reporting of study treatment errors including misuse/abuse

Study treatment errors are unintentional errors in the prescribing, dispensing, administration or monitoring of study treatment.

Study treatment misuse refers to situations where the study treatment is intentionally and inappropriately used not in accordance with the protocol.

Study treatment abuse corresponds to the persistent or sporadic, intentional excessive use of study treatment, which is accompanied by harmful physical or psychological effects.

Study treatment errors and uses outside of what is foreseen in the protocol, including misuse or abuse, must be reported on the AE (or SAE, if the event meets the definition of an SAE) CRF.

6.8 Concomitant and other therapy

6.8.1 Concomitant therapy

All medications, procedures, and significant non-drug therapies (including physical therapy and blood transfusions) administered after the participant entered the study must be recorded on the appropriate CRFs.

Each concomitant drug must be regularly assessed, based on the locally applicable label, against all entry criteria, prohibited medication and comparator reference labels (i.e. labels selected as Reference Safety Information). If in doubt, the investigator should contact the Novartis medical monitor before randomizing a participant or allowing a new medication to be started. If the participant is already enrolled, contact Novartis to determine if the participant should continue participation in the study. The participant should be instructed and encouraged to report any new medications/therapies he/she takes at any point in the study.

Heart failure therapy

As described in earlier sections of the protocol, all participants will receive the study medication on top of the guideline-directed background heart failure therapy in arms 1-4 or as a replacement of ACEI/ARB therapy in arm 5. Once the participants enters the study, every effort should be

made to avoid dose modifications of all heart failure therapy components, unless absolutely necessary to manage AEs.

If the participant experiences AEs and non-pharmacological interventions were not effective in relieving these AEs, the investigator can down-titrate or stop concomitant medications or oral study medications to rectify the situation, per his/her clinical judgment and reflecting the nature of the AE.

Before adjustment(s) are made to doses of foundational background HF treatments (i.e., ACEI/ARB/ARNI, MRA, beta blocker, and SGLT2 inhibitor), consideration should be given to modifications of other medications (e.g., in the case of hypotension, to adjust diuretics, calcium channel blockers (CCBs), alpha-blockers or nitrates).

If adjustment of background heart failure treatments are considered the only possibility to manage AEs or tolerability, the investigator should down-titrate or temporarily stop these medications (i.e. ACEI/ARB, sacubitril/valsartan, beta-blocker, MRA or SGLT2 inhibitor) or sacubitril/valsartan if this is the randomized study medication (i.e., participant randomized to arm 5) as per his/her clinical judgment. Background HF treatments should be reinstated at their pre-study dose as soon as possible to ensure proper care per the investigator's judgment.

Please refer to Section 10.7 pertaining to hypotension and to Section 10.8 pertaining to hyperkalemia for instructions on the steps to be taken to address these if they occur.

6.8.1.1 Permitted concomitant therapy requiring caution and/or action

Anti-hypertensive medication

Anti-hypertensive medication not directly targeting the RAS (including but not limited to CCBs, alpha-blockers, centrally acting agents, etc.) should be used with caution during screening and randomized treatment periods due to the theoretically increased possibility of occurrence of hypotension.

Phosphodiesterase-5 (PDE-5) inhibitors, oral nitrates and vericiguat

PDE-5 inhibitors, oral nitrates and vericiguat should be used with caution during screening and randomized treatment periods due to the increased possibility of the occurrence of hypotension.

Intravenous (IV) nitrates, nesiritide and carperitide

The concomitant administration of XXB750 with IV nitrates, nesiritide, and carperitide has not been studied. In the event a study participant requires the concomitant administration of one of these IV medications with the study medications, the investigator should consider starting them at a low dose or a slow infusion rate while monitoring the participant's BP carefully.

6.8.2 Prohibited medication

Other than the guideline-directed heart failure treatment mandated in the study, use of the treatments described below are not allowed during the randomized treatment period.

For participants in treatment arm 5, the concomitant use of an ACEI/ARB is strictly prohibited while the patient is receiving study medication during the randomized treatment period. If the

investigator believes that treatment with an ACEI/ARB is necessary, the open-label sacubitril/valsartan study drug must be discontinued. Study medication should be stopped 36 hours prior to the first dose of an ACEI. Similarly, if study medication (i.e. sacubitril/valsartan) is to be restarted, the background treatment with an ACEI should be discontinued 36 hours prior to resuming the open-label sacubitril/valsartan study medication.

6.8.3 Rescue medicine

Not applicable.

7 Discontinuation of study treatment and participant discontinuation/withdrawal

7.1 Discontinuation of study treatment

Discontinuation of study treatment for a participant occurs when study treatment is permanently stopped for any reason (prior to the planned completion of study treatment administration, if any) and can be initiated by either the participant or the Investigator.

The Investigator must discontinue study treatment for a given participant if, he/she believes that continuation would negatively impact the participant's well-being.

Discontinuation from study treatment is required under the following circumstances :

- Participant/guardian decision
- Pregnancy
- Use of prohibited treatment as per recommendations in the prohibited treatment section
- Any situation in which continued study participation might result in a safety risk to the participant
- Following emergency unblinding

If discontinuation from study treatment occurs, the Investigator should make a reasonable effort to understand the primary reason for the participant's discontinuation from study treatment and record this information.

Participants who discontinue from study treatment agree to return for the end of treatment and follow-up visits and undergo all study procedures indicated in Section 1.3 Schedule of Activities. Thus, discontinuation of study medication does not imply discontinuation from the study as a whole, unless the participant withdraws his/her consent for participation. If the participant cannot or is unwilling to attend any visit(s), the site staff should maintain regular telephone contact with the participant, or with a person pre-designated by the participant. This telephone contact should preferably be done according to the study visit schedule.

After discontinuation from study treatment, at a minimum, in abbreviated visits, the following data should be collected at clinic visits or via telephone/email contact:

- New / concomitant treatments
- Adverse Events / Serious Adverse Events

The Investigator must also contact the IRT to register the participant's discontinuation from study treatment.

7.2 Participant discontinuation from the study

Discontinuation from study is when the participant permanently stops receiving the study treatment, and further protocol-required assessments or follow-up, for any reason.

If the participant agrees, a final evaluation at the time of the participant's study discontinuation should be made as detailed in Section 1.3 Schedule of Activities.

7.3 Withdrawal of informed consent and exercise of participants' data privacy rights

Withdrawal of consent/opposition to use of data and/or biological samples occurs in countries where the legal justification to collect and process the data is consent and when a participant:

- Explicitly requests to stop use of their data
  and
- No longer wishes to receive study treatment
  and
- Does not want any further visits or assessments (including further study-related contacts)

This request should be as per local regulations (e.g. in writing) and recorded in the source documentation.

Withdrawal of consent impacts ability to further contact the participant, collect follow-up data (e.g. to respond to data queries) and potentially other country-specific restrictions. It is therefore very important to ensure accurate recording of withdrawal vs. discontinuation based on the protocol definitions of these terms.

In this situation, the Investigator should make a reasonable effort (e.g. telephone, e-mail, letter) to understand the primary reason for the participant's decision to withdraw their consent/exercise data privacy rights and record this information. The Investigator shall clearly document if the participant has withdrawn his/her consent for the use of data in addition to a study discontinuation.

Study treatment must be discontinued and no further assessments conducted, and the data that would have been collected at subsequent visits will be considered missing.

Further attempts to contact the participant are not allowed unless safety findings require communicating or follow-up.

If the participant agrees, a final evaluation at the time of the participant's withdrawal of consent/exercise data privacy rights should be made as detailed in Section 1.3 Schedule of Activities.

Further details on withdrawal of consent or the exercise of participants' data privacy rights are included in the corresponding informed consent form.

7.4 Lost to follow-up

For participants whose status is unclear because they fail to appear for study visits or fail to respond to any site attempts to contact them without stating an intention to discontinue from study treatment or discontinue from study or withdraw consent (or exercise other participants' data privacy rights), the Investigator must show "due diligence" by documenting in the source documents steps taken to contact the participant, e.g. dates of telephone calls, registered letters, etc. A participant should not be considered as lost to follow-up until due diligence has been completed.

7.5 Early study termination by the Sponsor

The study can be terminated by Novartis at any time.

Reasons for early termination (but not limited to) :

- Unexpected, significant, or unacceptable safety risk to participants enrolled in the study.
- Decision based on recommendations from applicable board(s) after review of safety and efficacy data
- Discontinuation of study treatment development

In taking the decision to terminate, Novartis will always consider participant welfare and safety. Should early termination be necessary, participants must be seen as soon as possible and treated as a participant who discontinued from study treatment. Participants should be invited for EOT visit and Investigator should ensure the participant will be appropriately switched to standard of care therapy. The Investigator may be informed of additional procedures to be followed in order to ensure that adequate consideration is given to the protection of the participant's interests. The Investigator or Novartis depending on local regulation will be responsible for informing IRBs/IECs of the early termination of the trial.

8 Study Assessments and Procedures

The Assessment Schedule (Table 1-1) lists all the assessments and when they are performed. All data obtained from these assessments must be supported in the participant's source documentation. Adherence to the study design requirements, including those specified in the Assessment Schedule, are essential and required for study conduct.

8.1 Screening

Once the participant has signed the informed consent form, the participant may enter the screening period.

The initial screening period can last approximately 7 days. Upon verification of eligibility inclusion/exclusion criteria, the participant will be eligible to enter the randomized treatment period. Concomitant medication information will be collected to ensure patient's eligibility for the trial. No changes in the patient's background anti-RAS treatment should be made after screening.

LVEF measurements required for eligibility will be based on the most recent locally obtained echocardiograms, multiple gated acquisition scanning (MUGA), cardiac computed

tomographic angiography (CTA), magnetic resonance imaging (MRI), or ventricular angiographies performed within the last 6 months provided that no subsequent measurements were ≥ 50%. If an LVEF measurement from the last 6 months is not available, the patient may enter the trial based on a LVEF < 50% obtained during the screening period, i.e., before assigning any study medication. If a patient has an implanted cardiac resynchronization therapy device or a myocardial infarction, the LVEF value used to qualify for the study must be obtained at least three months after the implantation of that device or the reference myocardial infarction.

Blood samples obtained for assessing eligibility NT-proBNP must be obtained on the same day as the screening ECG. In the case where a safety laboratory assessment at screening is outside of the allowed range specified in the eligibility criteria, the assessment may be repeated once per the investigator's discretion to confirm the patient's eligibility for continuing in the study. If the repeat value remains outside of the specified range, the participant must be screen failed. Refer to Section 5.3 (Screen failures) for further details.

8.2 Participant demographics/other baseline characteristics

Country-specific regulations should be considered for the collection of demographic and baseline characteristics in alignment with CRF.

Participant demographics: full date (only if required and permitted) or year of birth or age, sex, race/predominant ethnicity (if permitted) and relevant medical history/current medical conditions (until date of signature of informed consent) will be recorded in the eCRF. Where possible, the diagnosis and not symptoms should be recorded. Participant race/ethnicity data are collected and analyzed to assess the degree of diversity of the study population as required by Health Authorities and to identify any differences in the safety and/or efficacy profile of the treatment due to these characteristics.

All prescription medications, over-the-counter drugs and significant non-drug therapies used within 30 days before the start of the study must be documented. See the protocol Section 6.8 for further details on what information must be recorded on the appropriate page of the eCRF.

8.3 Efficacy assessments

Planned time points for all efficacy assessments are provided in the Assessment Schedule (Section 1.3).

8.3.1 NT-proBNP

NT-proBNP will be assessed in all participants by using the central laboratory at the screening visit to determine eligibility for the trial and at time points outlined in the Schedule of Assessments (see Table 1-1). These samples will be obtained before participants take their morning study drug dose (if applicable per assignment to treatment arm 5).

Only the screening NT-proBNP value will be reported to the investigator for the purpose of determining eligibility for inclusion in the study. Samples collected for determining NT-proBNP levels at Randomization and thereafter will be analyzed in batches to ensure uniformity of analytical processes and reagents. Post-Screening NT-proBNP values will not be reported to investigators to avoid the potential introduction of bias. Moreover, investigators are encouraged

to avoid assessing NT-proBNP levels from local laboratories as much as feasible to avoid introduction of bias.

8.3.2 Secondary efficacy assessments

Not applicable.

8.3.3 Appropriateness of efficacy assessments

NT-proBNP is considered the best available surrogate marker when evaluating NP system modulation in heart failure and thus used to assess efficacy in this study. Reduction in NT-proBNP levels has been linked to improved risk of long-term HF outcomes. Refer to Section 4.2.2 for more details.

8.4 Safety assessments

Safety assessments are specified below with Section 1.3 Schedule of Activities detailing when each assessment is to be performed.

For details on AE collection and reporting, refer to Section 8.6.

As per Section 4.5, during a public health emergency as declared by local or regional authorities i.e. pandemic, epidemic or natural disaster, that limits or prevents on-site study visits, regular phone or virtual calls can occur for safety monitoring and discussion of the participant´s health status until it is safe for the participant to visit the site again.

8.4.1 Physical examinations

Physical examinations will be performed by the investigator or his/her designee who are qualified to do so per local regulations/standards. Information about all physical examinations performed must be included in the source documentation at the study site. Clinically relevant findings that are present before signing informed consent must be recorded on the appropriate CRF page that captures medical history. Significant findings made after signing informed consent which meet the definition of an AE must be recorded as an AE.

A **complete physical** examination will include examination of the general appearance, vital signs (blood pressure [SBP and DBP] and pulse), skin, neck (including thyroid), eyes, ears, nose, throat, lungs, heart, abdomen, back, lymph nodes, extremities, the vascular, and the neurological systems. If indicated based on medical history and/or symptoms, rectal, external genitalia, breast, and pelvic exams will be performed.

An **abbreviated physical** examination will include examination of the general appearance and vital signs (blood pressure [SBP and DBP] and pulse).

Refer to Assessment Schedule Table 1-1 for details of the type and frequency of the physical examination.

8.4.2 Vital signs

Vital signs include BP and pulse measurements. BP will be measured in the sitting position after 5 minutes of rest using an automated validated device (e.g., OMRON) or a standard

sphygmomanometer with an appropriately sized cuff on the non-dominant arm. Guidelines for the management of hypotension are provided in Section 10.7.

8.4.3 Height and weight

Height in centimeters (cm) will be measured at screening.

Body weight (to the nearest 0.1 kilogram (kg) in indoor clothing without shoes) will be measured at screening, randomization and every visit thereafter.

8.4.4 Electrocardiograms

Single local 12-lead ECGs will be collected at Screening, Week 16 and Week 24 visits.

ECGs will be locally collected and evaluated. Interpretation of the tracing must be made by a qualified physician and documented in source and on the appropriate CRF. Each ECG tracing should be labeled with the study number, participant initials (where regulations permit), participant number, date and kept in the source documents at the study site. Clinically significant abnormalities present at randomization should be documented as medical history/current medical conditions on the appropriate CRF. New or worsened clinically significant findings occurring after entering the randomized treatment period must be recorded as adverse events.

The original ECGs on non-heat-sensitive paper or a certified copy on non-heat sensitive paper, appropriately signed, must be archived at the study site.

Additionally, unscheduled safety ECGs may be repeated at the discretion of the investigator at any time during the study as clinically indicated. For any ECGs that raises a safety concern, two additional ECGs must be performed to confirm the safety finding. ECG safety monitoring, or a review process (as per the investigator's discretion), should be in place for clinically significant ECG findings at baseline before administration of study treatment and during the study.

8.4.5 Clinical safety laboratory tests

A central laboratory will be used for analysis of all specimens collected. Details on the collection, shipment of samples and reporting of results by the central laboratory are provided to investigators in the laboratory manual. Please refer to Table 1-1.

If participants cannot visit the site for safety lab assessments conducted through central labs during a public health emergency as declared by local or regional authorities, i.e., pandemic, epidemic or natural disaster, that limits or prevents on-site study visits or as deemed appropriate by the investigator in the course of study conduct, an alternative (local) lab collection site may be used.

For China, POCT (e.g. urinalysis, urine pregnancy) is to be analyzed at local laboratories. When study-required testing is not available at a local site, a central lab could be used.

Clinically notable laboratory findings are defined in Section 10.3.1. Clinically significant abnormalities must be recorded as either medical history/current medical conditions or adverse events as appropriate.

Table 8-1 lists the clinical safety laboratory tests performed during the study. The tests with an asterisk (*) beside their names are part of the abbreviated panel of clinical safety laboratory tests.

Table 8-1 Clinical Safety Laboratory Tests

Test Category	Test Name
Hematology	Hematocrit, Hemoglobin, Mean Corpuscular Hemoglobin, Mean Corpuscular HGB Concentration, Mean Corpuscular Volume, Platelets, Erythrocytes, Leukocytes, Differential (% Basophils, Eosinophils, Lymphocytes, Monocytes, Neutrophils), Hemoglobin A1c
Chemistry	Albumin*, Alkaline phosphatase*, ALT*, AST*, Gamma-glutamyl-transferase (GGT)*, Lactate Dehydrogenase (LDH), Calcium*, Magnesium, Phosphate, Chloride, Sodium*, Potassium*, Creatinine (enzymatic)*, Creatine Kinase*, Direct Bilirubin*, Total Bilirubin, Lipid Panel (Total Cholesterol, LDL Cholesterol, High-density lipoprotein [HDL] Cholesterol, Triglycerides [random]), Total Protein, Blood Urea Nitrogen (BUN)*, eGFR*, Uric Acid, Amylase, Lipase, Glucose (random)
Urinalysis	Macroscopic (Dipstick) Panel* (Color, Bilirubin, Occult Blood, Macroscopic Blood, Glucose, Ketones, Leukocyte Esterase, Nitrite, pH, Protein, Specific Gravity, Urobilinogen)<br>Microscopic Panel** (Erythrocytes, Leukocytes, Casts, Crystals, Bacteria, Epithelial cells)
Coagulation	Prothrombin Time/INR*
Pregnancy Test	Highly sensitive serum or urine human chorionic gonadotropin (hCG) pregnancy test (as needed for women of childbearing potential)
Assessments of Fertility	Follicle-stimulating hormone and estradiol (as needed in women of non-child bearing potential only)
*Test is part of the abbreviated panel of clinical safety laboratory tests<br>**Microscopic Panel only to be tested in the event that the Macroscopic (Dipstick) results were positive	

8.4.6 Pregnancy testing

All women of childbearing potential will undergo pregnancy testing. Additional pregnancy testing might be performed if requested by local requirements.

Serum pregnancy testing with beta-human chorionic gonadotropin (β-hCG) must be done at screening, at Week 16 (EOT) visit and at Week 24 (EOS) visit. Urine pregnancy test should be performed at randomization visit (before randomization), at Week 4, Week 8, Week 12, and Week 20. A negative Screening serum pregnancy test result (not older than 7 days) **and** a negative Randomization urine pregnancy test result, must be available before the subject is randomized. A positive urine pregnancy test must be confirmed with a serum pregnancy test. If the latter is also positive, the participant must be discontinued from study treatment.

As per Section 4.5, during a public health emergency as declared by local or regional authorities' i.e. pandemic, epidemic or natural disaster, that limits or prevents on-site study

visits, if participants cannot visit the site to have serum pregnancy tests, urine pregnancy test kits may be used. Relevant participants can perform the urine pregnancy test at home and report the result to the site. It is important that participants are instructed to perform the urine pregnancy test first and only if the test result is negative proceed with the administration of the study treatment. A communication process should be established with the participant so that the site is informed and can verify the pregnancy test results (e.g. following country specific measures).

In general, the levels of a mAb like XXB750 in semen are expected to be low; therefore, the subsequent levels in female partner would be negligible. Based on this principle, the probability that XXB750 would cause teratogenicity is very low. Therefore, no restrictions will be placed on male participants with regard to sexual activity or condom use.

Assessments of fertility

Assessment of fertility must be performed at screening. A woman is considered of childbearing potential from menarche and until becoming post-menopausal unless permanently sterile. Permanent sterilization methods include hysterectomy, bilateral tubal ligation, bilateral salpingectomy and bilateral oophorectomy. In the case of oophorectomy alone, only when the reproductive status of the woman has been confirmed by follow-up hormone level assessment is she considered to be not of child-bearing potential. Medical documentation of permanent method of sterilization must be retained as source documents.

A postmenopausal state is defined as no menses for 12 months without an alternative medical cause and an appropriate clinical profile.

In absence of the medical documentation confirming permanent sterilization, or if the post-menopausal status is not clear, the investigator should use his medical judgment to appropriately evaluate the fertility state of the woman and document it in the source document. Also, in these cases, follicle-stimulating hormone (FSH) testing is required, at screening, for any female participant regardless of reported reproductive/menopausal status.

8.4.7 Other safety evaluations

8.4.7.1 Liver safety monitoring

To ensure participant safety and enhance reliability in determining the hepatotoxic potential of an investigational drug, a standardized process for identification, monitoring and evaluation of liver events has to be followed.

Please refer to Table 10-1 in Section 10.5.1 for complete definitions of liver laboratory triggers.

Once a participant is exposed to study treatment, every liver event defined in Table 10-1 should be followed up by the investigator or designated personnel at the trial site, as summarized below. Additional details on actions required in case of liver events are outlined in Table 10-2 and Table 10-3. Repeat liver chemistry tests (i.e., SGOT, SGPT, total bilirubin (TBL), PT/INR, alkaline phosphatase (ALP) and GGT) should be done to confirm elevation.

These liver chemistry repeats will be performed using the central laboratory. If results will not be available from the central laboratory, then the repeats can also be performed at a local

laboratory to monitor the safety of the participant. If a liver event is subsequently reported, any local liver chemistry tests previously conducted that are associated with this event should have results recorded on the appropriate CRF.

If the initial elevation is confirmed, close observation of the participant will be initiated, including consideration of treatment interruption if deemed appropriate.

If a liver AE is reported that corresponds to these liver events, an assessment of the causal role of each study medication in the origin of the liver AE must be provided.

The following actions should be considered:

- Discontinuation of the investigational drug (refer to the discontinuation of study treatment, Section 7.1), if appropriate
- Hospitalization of the participant if appropriate
- Causality assessment of the liver event
- Thorough follow-up of the liver event should include, as per the Investigator's discretion: obtaining more details on the history of the liver event (including its symptoms), as well as on prior and concurrent diseases, concomitant drug use, serology tests, imaging and pathology assessments, hepatologist's consultancy, ruling out any underlying liver disease.

In addition, the follow-up actions described in Section 6.5.2.1 should be performed. All follow-up information and findings from consultations and procedures must be recorded as appropriate in the CRFs.

8.4.7.2 Renal safety monitoring

Once a participant is exposed to study treatment, renal laboratory alerts or renal safety events should be monitored and followed up by the investigator or designated trial staff as summarized in Section 10.6.1.

8.4.7.3 Immunogenicity

Immunogenicity testing will be performed on each participant. The results will be analyzed to determine, among others, any impact of anti-drug antibodies (ADA) on the efficacy of XXB750 and on the occurrence of adverse events. Refer to Section 8.9 for more details on immunogenicity testing.

8.4.8 Appropriateness of safety measurements

The safety assessments selected are standard for this indication/participant population. PK sampling and immunogenicity assessments performed periodically during the study will allow to determine relationships between drug blood concentrations, existence of ADA (neutralizing or not) and the occurrence of certain adverse events.

8.5 Additional assessments

8.5.1 Clinical Outcome Assessments (COAs)

As per Section 4.5, during a public health emergency as declared by local or regional authorities i.e. pandemic, epidemic or natural disaster, that limits or prevents on-site study visits, COA data

may be collected remotely (e.g. web portal, telephone interviews) depending on local regulations, technical capabilities, and following any applicable training in the required process.

Patient reported outcomes (PRO)

The participant should be made aware that completed measure(s) are not reviewed by the investigator/study personnel.

The KCCQ is a self-administered questionnaire and requires, on average, 4-6 minutes to complete. It contains 23 items, covering physical function, clinical symptoms, social function, self-efficacy and knowledge, and Quality of Life (QoL), each with different Likert scaling wording, including limitations, frequency, bother, change in condition, understanding, levels of enjoyment and satisfaction. A change of 5 points on the scale scores, either as a group mean difference or an intra-individual change appears to be clinically significant, based on comparisons of changes in the scale scores to clinical indicators and patient global reports of change. The KCCQ is a valid, reliable and responsive health status measure for patients with CHF and may serve as a clinically meaningful outcome in CV clinical research, patient management and quality assessment (Green et al 2000).

The HF symptoms and physical limitation domains scores show the best correlation for improvements following a CHF exacerbation (Green et al 2000). Thus, the clinical summary score based on the HF symptoms and physical limitation domains scores of the KCCQ will be one exploratory endpoint next to the overall summary score when the instruments will be evaluated as a whole.

The KCCQ questionnaire will be completed at randomization and Week 16 visits, before any other study procedures and regardless of discontinuation of study treatment.

The KCCQ is available in a number of validated translations. However, participants in whose language a validated translation of the KCCQ is not available will be exempt from completing this instrument.

8.5.2 Other assessments

8.5.2.1 Estimated glomerular filtration rate (eGFR)

The eGFR to determine eligibility of the patient for screening into the trial will be calculated at screening from the serum creatinine concentration which will be measured centrally. The eGFR will be further measured as part of the abbreviated safety laboratory samples at each visit to guide the investigator to take any appropriate action as necessary (e.g., stopping study treatment) in case of eGFR decrease.

Estimated GFR will only be calculated using the following version of the CKD-EPI formula (Inker et al 2021):

$eGFR_{cr} = 142 \times \min(S_{cr}/\kappa, 1)^{\alpha} \times \max(S_{cr}/\kappa, 1)^{-1.200} \times 0.9938^{Age} \times 1.012$ [if female]

where $\kappa = 0.7$ (female) or 0.9 (male) and $\alpha = -0.241$ (female) or -0.302 (male)

8.5.2.2 Urinary cGMP to creatinine ratio

Urinary cGMP as a measure of XXB750's engagement with its target NPR1 will be assessed in spot urine samples at visits as outlined in Table 1-1. To allow indexing of urinary biomarkers, creatinine in spot urine will also be measured. Samples will be obtained before taking the morning study medication (as applicable per patient's treatment assignment).

8.5.2.3 Urine albumin to creatinine ratio (UACR)

Spot urine samples will be collected before taking the morning study drug dose (as applicable per assigned study medication) at the visits indicated in Table 1-1 for analysis of urinary albumin and creatinine (measured as UACR).

8.6 Adverse events (AEs), serious adverse events (SAEs), and other safety reporting

The definitions of adverse events (AEs) and serious adverse events (SAEs) can be found in Section 8.6.

AEs will be reported by the participant (or, when appropriate, by a caregiver, surrogate, or the participant’s legally authorized representative).

The Investigator and any qualified designees are responsible for detecting, documenting, and recording events that meet the definition of an AE or SAE and remain responsible for following up all AEs (see Section 7).

For the investigational product, information about adverse drug reactions and how to manage them can be found in the Investigator's Brochure (IB) or equivalent documentation. Information about adverse drug reactions can also be found in the product information for marketed products.

The method of recording, evaluating, and assessing causality of AEs and SAEs and the procedures for completing and transmitting SAE reports are provided in Section 8.6.3.

8.6.1 Adverse events

An adverse event (AE) is any untoward medical occurrence (e.g., any occurrence of unfavorable and unintended sign(s), symptom(s) or medical condition, including abnormal laboratory findings, or worsening of any pre-existing sign(s), symptom(s) or medical condition) in a participant after providing written informed consent for participation in the study. Therefore, an AE may or may not be temporally or causally associated with the use of any treatment used in this study. This includes events reported by the participant (or, when appropriate, by a caregiver, surrogate, or the participant’s legally authorized representative).

Abnormal laboratory values or test results constitute AEs only if they fulfill at least one of the following criteria:

- they induce clinical signs or symptoms
- they are considered clinically significant
- they require therapy

Clinically significant abnormal laboratory values or test results must be identified through a review of values outside of normal ranges/clinically notable ranges, significant changes from baseline or the previous visit, or values considered to be non-typical in participant with the underlying disease. Alert ranges for laboratory and other test abnormalities are included in Section 10.3.1.

The Investigator has the responsibility for managing the safety of individual participant and identifying adverse events.

Novartis qualified medical personnel will be readily available to advise on trial-related medical questions or problems.

The occurrence of adverse events must be sought by non-directive questioning of the participant at each visit during the study. Adverse events also may be detected when they are volunteered by the participant during or between visits or through physical examination findings, laboratory test findings, or other assessments, excluding Patient-Reported Outcomes (which are related to the underlying heart failure).

Adverse events must be recorded under the signs, symptoms, or diagnosis associated with them, accompanied by the following information (as far as possible) (if the event is serious refer to Section 8.6.2):

1. The severity grade.
    - Mild: A type of AE that is usually transient and may require only minimal treatment or therapeutic intervention. The event does not generally interfere with usual activities of daily living.
    - Moderate: A type of AE that is usually alleviated with additional specific therapeutic intervention. The event interferes with usual activities of daily living, causing discomfort but poses no significant or permanent risk of harm to the research participant.
    - Severe: A type of adverse event that interrupts usual activities of daily living, or significantly affects clinical status, or may require intensive therapeutic intervention
2. Causality
    - The investigator is obligated to assess the relationship between any treatment used in the study (study treatment, AxMP(s)) and each occurrence of each AE. The investigator will use clinical judgment to determine the relationship. A reasonable possibility of a relationship conveys that there are facts, evidence, and/or arguments to suggest a causal relationship, rather than a relationship cannot be ruled out. Alternative causes, such as underlying disease(s), concomitant therapy, and other risk factors, as well as the temporal relationship of the event to study intervention administration, will be considered and investigated. For causality assessment, the investigator will also consult the IB and/or product information, for marketed products. The causality assessment is one of the criteria used when determining regulatory reporting requirements.
3. Its duration (start and end dates or ongoing) and the outcome must be reported.
4. Whether it constitutes a SAE (see Section 8.6.2 for definition of SAE) and which seriousness criteria have been met.

5. Action taken regarding with study treatment. All adverse events must be treated appropriately. Treatment may include one or more of the following:
   - Dose not changed
   - Dose Reduced/increased
   - Drug interrupted/permanently discontinued
6. Its outcome

Conditions that were already present at the time of informed consent should be recorded in medical history of the participant.

Adverse events (including lab abnormalities that constitute AEs) should be described using a diagnosis whenever possible, rather than individual underlying signs and symptoms.

Adverse event monitoring should be continued at least until Visit Week 24 (EOS) or until 3 months after the last dose of injectable study medication, whichever is longer.

Once an adverse event is detected, it must be followed until its resolution or until it is judged to be not recovered/not resolved (e.g. continuing at the end of the study), and assessment must be made at each visit (or more frequently, if necessary) of any changes in severity, the suspected relationship to the interventions required to treat it, and the outcome.

Information about adverse drug reactions for the investigational drug can be found in the Investigator's Brochure (IB).

Abnormal laboratory values or test results constitute adverse events only if they fulfill at least one of the following criteria:

- they induce clinical signs or symptoms
- they are considered clinically significant
- they require therapy

Clinically significant abnormal laboratory values or test results must be identified through a review of values outside of normal ranges/clinically notable ranges, significant changes from baseline or the previous visit, or values which are considered to be non-typical in participant with the underlying disease. Alert ranges for laboratory and other test abnormalities are included in Section 10.3.

Reporting of AEs related to AxMP(s)

All AEs related to any authorized AxMP used in this study must be reported to Novartis.

In assessing causality, the investigators will use the points above.

If a suspicion that a medical occurrence could be related to study treatment (and/or interaction with study treatment) cannot be ruled out, the reporting rules for study treatment apply.

Handling of AEs

All adverse events must be treated appropriately. More information about how to manage AEs can be found in the IB. Information about adverse drug reactions can also be found in product information for marketed products.

Once an AE is detected, the Investigator must pro-actively followed up the participant, until resolution of the AE, or until it is judged to be not recovered/not resolved (e.g., continuing at the end of the study), or until stabilization, or until the participant is lost to follow-up. Any change in severity or suspected relationship to study treatment must be assessed at each visit (or more frequently, if necessary).

8.6.2 Serious adverse events

An SAE is defined as any adverse event [appearance of (or worsening of any pre-existing)] undesirable sign(s), symptom(s), or medical conditions(s) which meets any one of the following criteria:

- fatal
- life-threatening

Life-threatening in the context of a SAE refers to a reaction in which the participant was at risk of death at the time of the reaction; it does not refer to a reaction that hypothetically might have caused death if it were more severe (please refer to the ICH-E2D Guidelines).

- results in persistent or significant disability/incapacity
- constitutes a congenital anomaly/birth defect, fetal death or a congenital abnormality or birth defect
- requires inpatient hospitalization or prolongation of existing hospitalization, unless hospitalization is for:
  - routine treatment or monitoring of the studied indication, not associated with any deterioration in condition
  - elective or pre-planned treatment for a pre-existing condition that is unrelated to the indication under study and has not worsened since signing the informed consent
  - social reasons and respite care in the absence of any deterioration in the participant's general condition
  - treatment on an emergency outpatient basis for an event not fulfilling any of the definitions of a SAE given above and not resulting in hospital admission
- is medically significant, e.g. defined as an event that jeopardizes the participant or may require medical or surgical intervention to prevent one of the outcomes listed above

Medical and scientific judgment should be exercised in deciding whether other situations should be considered serious reactions, such as important medical events that might not be immediately life-threatening or result in death or hospitalization but might jeopardize the participant or might require intervention to prevent one of the other outcomes listed above. Such events should be considered as "medically significant." Examples of such events are intensive treatment in an emergency room or at home for allergic bronchospasm, blood dyscrasias, or convulsions that do not result in hospitalization or development of dependency or abuse (please refer to the ICH-E2D Guidelines).

All new malignant neoplasms will be assessed as serious under "medically significant" if other seriousness criteria are not met.

All reports of intentional misuse and abuse of the product are also considered serious adverse events irrespective of whether a clinical event has occurred.

Any suspected transmission via a medicinal product of an infectious agent is also considered a serious adverse reaction.

8.6.3 SAE reporting

To ensure participant safety, every SAE, regardless of causality, occurring after the participant has provided informed consent and until End of Study visit must be reported to Novartis safety immediately, without undue delay, but under no circumstances later than within 24 hours of obtaining knowledge of the events (Note: If more stringent, local regulations regarding reporting timelines prevail). Detailed instructions regarding the submission process and requirements are to be found in the Investigator folder provided to each site. Information about all SAEs is collected and recorded on the electronic Serious Adverse Event Report Form with paper backup (as further detailed in the CRF Completion Guideline). All applicable sections of the form must be completed in order to provide a clinically thorough report.

SAE occurring between the time the participant has provided informed consent until either EOS visit or until 3 months after the last dose of injectable study medication (whichever is longer) must be reported to Novartis. Any SAE experienced by participants who discontinue study treatment prematurely but remain in the study for follow-up, should continue to be reported until either EOS visit or until 3 months after the last dose of injectable study medication (whichever is longer).

All follow-up information for the SAE including information on complications, progression of the initial SAE and recurrent episodes must be reported as follow-up to the original episode immediately, without undue delay, but under no circumstances later than within 24 hours of the Investigator receiving the follow-up information (Note: If more stringent, local regulations regarding reporting timelines prevail). An SAE occurring at a different time interval or otherwise considered completely unrelated to a previously reported one must be reported separately as a new event.

Any SAEs experienced after the EOS visit or 3 months after the last dose of injectable study medication (whichever is longer) should only be reported to Novartis Safety if the Investigator suspects a causal relationship to study treatment, unless otherwise specified by local law/regulations.

The investigator must review and provide an assessment of causality for each SAE. There may be situations in which an SAE has occurred, and the investigator has minimal information to include in the initial report to Novartis. However, it is very important that the investigator always makes an assessment of causality for every event before the initial transmission of the SAE data to Novartis. The investigator may change his/her opinion of causality in light of follow-up information and send an SAE follow-up report with the updated causality assessment.

Reporting of Suspected Unexpected Serious Adverse Reactions (SUSARs)

If a SAE is not previously documented in the IB or product information for marketed products and is thought to be related to any study treatment, Novartis may urgently require further information from the investigator for HA reporting. Novartis may need to issue an Investigator

Notification (IN) to inform all investigators involved in any study with the same study treatment. SUSARs will be reported to the competent authorities and relevant ethics committees in accordance with national regulatory requirements in participating countries, including EU Clinical Trial Regulation 536/2014.

Study-specific unblinding rules for suspected unexpected serious adverse reactions (SUSARs) that are disease-related

In clinical trials evaluating treatments for high morbidity and/or high mortality disease states, SAEs that are known consequences of the underlying disease or condition under investigation, or events common in the study population, are anticipated to occur with some frequency during the course of the trial, regardless of drug exposure. While the investigator must still report all SAEs according to the instructions provided in Section 8.6.2 and Section 8.6.3, SUSARs containing terms that are considered consistent with the MedDRA Preferred Terms listed in Table 8-2 will not be unblinded and will not be reported in an expedited timeframe to regulatory agencies, ethic committees or investigators during the course of the study (FDA 2021). Investigator notifications will not be issued for these reports. An external independent DMC will review data of the ongoing trial on a regular basis (Section 10.1.4.1). The opinion and the recommendations of the DMC will be notified by Novartis as soon as possible to the competent authorities and the EC's where they qualify for expedited reporting. If specifically requested by a local Health Authority (HA), SUSARs reporting the terms in Table 8-2 will be expedited to this Health Authority as blinded reports and Investigator notifications will not be issued for these events. Alternatively, if specifically requested by a local HA, SUSARs reporting the terms in Table 8-2 that occur in participants under the jurisdiction of the requesting HA will be expedited to that HA as unblinded reports and investigator notifications will be issued for these events to investigators under the jurisdiction of this HA. These events will be presented in the clinical study report at the end of the study. Any other SUSAR not reporting any term included in Table 8-2 will be unblinded and reported to the competent authorities and relevant ECs. In these cases, IN will be issued when applicable.

Table 8-2 MedDRA PT related to the underlying heart failure

Cardiac output decreased	Pulmonary arterial wedge pressure increased	Cardiogenic shock	Ventricular failure	Pulmonary oedema
Left ventricular end-diastolic pressure decreased	Pulmonary arterial wedge pressure abnormal	Cardiac failure high output	Low cardiac output syndrome	Left ventricular failure
Ejection fraction abnormal	Cardiac failure	Cardiac failure congestive	Cardio-respiratory distress	Acute left ventricular failure
Ejection fraction decreased	Cardiac failure acute	Cardiac failure chronic	Acute pulmonary oedema	

Reporting of SAEs related to AxMP(s)

All SAEs related to any AxMP (whether authorized or not) used in this study must be reported to Novartis within 24 hours of the site becoming aware of it. In assessing causality, the investigators will use the points above. If a suspicion that the medical occurrence could be

related to study treatment (or and interaction with study treatment) cannot be ruled out, the reporting rules for study treatment apply.

8.6.4 Pregnancy

- Details of all pregnancies in female participants will be collected after the start of study treatment and until 3 months after the last dose of injectable study medication .
- While pregnancy itself is not considered to be an AE or SAE, any pregnancy complication or elective termination of a pregnancy for medical reasons will be reported as an AE or SAE.
- Abnormal pregnancy outcomes (e.g. spontaneous abortion, fetal death, stillbirth, congenital anomalies, ectopic pregnancy) are considered SAEs and will be reported as such.
- Any post study pregnancy-related SAE considered reasonably related to the study treatment by the Investigator will be reported to Novartis as described in Section 8.6.3. While the Investigator is not obligated to actively seek this information in former study participants, he or she may learn of an SAE through spontaneous reporting.
- Any female participant who becomes pregnant while participating in the study will discontinue study treatment. This participant may stay in the study and follow the assessments, if she wishes to do so. All assessments that are considered as a risk during pregnancy must not be performed. The participant may continue all other protocol assessments.

Pregnancies

If a female trial participant becomes pregnant, the study treatment should be stopped, and the pregnancy consent form should be presented to the trial participant. The participant must be given adequate time to read, review and sign the pregnancy consent form. This consent form is necessary to allow the Investigator to collect and report information regarding the pregnancy. To ensure participant safety, each pregnancy occurring after signing the informed consent must be reported to Novartis within 24 hours of learning of its occurrence. The pregnancy should be followed up to determine outcome, including spontaneous or voluntary termination, details of the birth, and the presence or absence of any birth defects, congenital abnormalities, or maternal and/or newborn complications.

Pregnancy should be recorded and reported by the Investigator to the Novartis PS&PV Department. Pregnancy follow-up should be recorded on the same form and should include an assessment of the possible relationship to the study treatment of any pregnancy outcome. Any SAE experienced during pregnancy must be reported.

After consent is provided, the pregnancy reporting will occur up to one year after the estimated date of delivery. Post-natal follow-up should occur at 1, 3 and 12 months after delivery.

8.6.5 Disease-related events and/or disease-related outcomes not qualifying as AEs or SAEs

No disease-related events not qualifying as AEs or SAEs are specified in this study.

8.6.6 Adverse events of special interest

Adverse events of special interest (AESI) are defined as events which are of scientific and medical interest specific to Novartis's product or program, for which ongoing monitoring may be appropriate. Such events may require further investigation in order to characterize and understand them. AESI are defined on the basis of potential safety risks for the product, class effects, and data from preclinical studies. The following are currently defined as AESI for XXB750 in this study: serious hypotension (i.e., meeting any of the seriousness criteria listed in Section 8.6.2), serious hypersensitivity reactions (including, but not limited to, allergic reactions, anaphylaxis or anaphylactoid reactions, cytokine release syndrome, serum sickness or serum sickness-like reactions), serious injection site reactions, severe tachycardia, severe acute bradycardia and serious presyncope/syncope.

8.7 Pharmacokinetics

A population pharmacokinetics (PK) sub-study will be conducted in approximately 200 participants who are randomized to either XXB750 or matching placebo. Pharmacokinetics samples will be collected in these participants at the visits defined in the assessment schedule at randomization (2-4 hours post dose), Week 4 (prior to AND 2-4 hours post dose), Week 12 (prior to AND 2-4 hours post dose), Week 16 and Week 24. Follow instructions outlined in the laboratory manual regarding sample collection, numbering, processing and shipment. In order to better define the PK profile, the timing of the PK sample collection may be altered based on emergent data. The number of samples/blood draws and total blood volume collected will not exceed those stated in the protocol.

Pharmacokinetic samples will be obtained in all eligible participants enrolled at sites participating in this sub-study, but evaluated only in participants treated with XXB750. XXB750 concentration will be determined by a validated method; the anticipated Lower Limit of Quantification (LLOQ) is 50 ng/mL. Concentrations will be expressed in mass per volume units. Concentrations below the LLOQ will be reported as "zero" and missing data will be labeled as such in the Bioanalytical Data Report. The individual data will be listed. Population PK analysis of XXB750 will be conducted and the results of this analysis will be reported separately.

8.8 Biomarkers

As described in the Assessment Schedule (Table 1-1), blood and urine will be collected for biomarker assessments from all participants (where feasible). The sample collection information, including the exact time and date of collection, must be entered on the appropriate sample collection eCRF page(s).

Biomarkers related to heart failure and/or XXB750 mechanism of action or treatment effect will be analyzed. In addition to NT-proBNP, plasma and urine cGMP, BNP and hs-Troponin will be analyzed. Biomarkers related to the renin-angiotensin-aldosterone system may also be tested using biobanked samples. ANP will be tested at baseline to determine if ANP levels at the start of XXB750 treatment modify its NT-proBNP effect. Urine creatinine will be analyzed to allow indexing of urine cGMP and other urine biomarkers. Proteins (via SomaScan profiling) or

metabolites that may be associated with heart failure development or progression, study drug treatment response or predict response to treatment will also be analyzed.

Due to both posture and time of collection affecting levels of several of the planned biomarkers, the laboratory manual will include instructions for: 1) participants to be calm and seated for 15 minutes prior to blood biomarker sampling and 2) urine and blood biomarker samples be collected in the morning with no greater than a 1-hour difference between collection times at the post-randomization visits compared to the time at the randomization visit. For a detailed description regarding sample collection, numbering, processing, and shipment, please refer to the instructions provided in the laboratory manual.

Biobank samples (where permitted) will be collected to allow the list of biomarkers to change during the study due to new assay availability, technical issues or as more relevant biomarkers/proteins are determined during conduct of the XXB750 program or clinical research in heart failure. Thus, additional biomarkers related to XXB750 or heart failure, may be tested during the study or after study completion. Additional biomarkers may include those related to fibrosis such as soluble ST2 or collagen synthesis or degradation biomarkers. Except for NT-proBNP levels at screening, biomarker results will remain blinded and will not be available to investigators or the Novartis study team during the study. The Novartis biomarker bioanalyst, who is not involved in study management, will have access to unblinded biomarker data to assess for the need of re-analyzing samples due to suspected technical issue. The Novartis biomarker bioanalyst will remain blinded to the patient level and group level treatment assignments during the entire study.

While the goal of the biomarker assessments is to provide supportive data for the clinical study, there may be circumstances when a decision is made to stop a collection, or to not perform or discontinue an analysis due to either practical or strategic reasons (e.g., inadequate sample number, issues related to the quality of the sample or issues related to the assay that preclude analysis, impossibility to perform correlative analyses, etc.). Therefore, depending on the results obtained during the study, sample analysis for specific biomarkers or specific participants may be omitted at the discretion of Novartis.

Exploratory DNA sampling

The study includes an optional genetic research component which requires a separate informed consent signature if the participant agrees to participate. As permitted by local governing regulations and by IRB/EC, it is required as part of this protocol that the investigator presents these options to the participant.

The optional DNA sample is to be collected at randomization or later, as described in Assessment Schedule (Table 1-1). Instructions for sample collection and shipment will be provided in the laboratory manual. The sample will be sent to the Central Lab for DNA extraction and stored by the Central Lab or Novartis until genetic analyses are performed during the study or after the study end.

The purpose of genetic research may be to better understand the safety and efficacy of XXB750, to learn more about human diseases, or to help develop ways to detect, monitor and treat diseases. As technology changes over time, the most appropriate technology will be used at the

time the exploratory genetic research is performed. This may include the study of the entire genome.

The use of DNA to search for biomarkers of disease and drug action is exploratory. Any results from this DNA study will not be placed in the participant's medical records. To maximize confidentiality, all samples will be double-coded to prevent the exposure of the participant's information and identity. This double-coding process allows Novartis to go back and destroy the sample at the participant's request. In addition, sample information is stored in one secured database while genetic data is stored in an independent secured database.

8.9 Immunogenicity assessments

Anti-drug antibody (ADA) samples will be obtained in participants randomized to injectable study medication (i.e. treatment arms 1-4) according to Section 1.3 Schedule of Activities. A validated ligand binding assay (i.e. ELISA, MSD, etc.) will be used for the detection of potential anti-XXB750 antibodies. Confirmed immunogenicity positive samples may be further analyzed for presence of neutralizing antibodies using a validated method.

In case of a suspected allergic hypersensitivity reaction, samples to measure ADA and a panel of immunoglobulins (IGs), as well as samples to measure tryptase (if sampling occurs within 4 hours following a suspected hypersensitivity reaction), are also to be collected.

If the hypersensitivity reaction is observed at the site following study drug administration, samples for ADA, the IG panel, and tryptase, should be collected as soon as possible after the reaction (and no longer than 4 hours after the reaction commenced). In the event the reaction occurs off-site, the ADA and IG panel samples should be collected at the time of the next site visit. Additionally, the ADA and IG panel samples should also be collected at the final visit from participants who discontinued study treatment or were withdrawn from the study. IG panel testing is planned for drug-specific IgE and other immunoglobulins (e.g. IgA/IgG1/IgG2/IgG3/IgG4/IgM). Depending on the results of the IG panel, antibody epitope mapping, immunogenicity profiling, and serological profiling may also be performed using backup IG panel and backup pre-dose PK samples.

Follow instructions outlined in the Laboratory Manual regarding sample collection, numbering, processing, and shipment.

The detailed methods for immunogenicity assessment will be described in the Bioanalytical Data Report.

8.10 Medical resource utilization and health economics

Medical resource utilization and health economics parameters are not evaluated in this study.

9 Statistical considerations

9.1 Analysis sets

The following analysis sets will be defined for statistical analysis:

Screened Set (SCR) - All participants who signed the informed consent form. The SCR includes only unique screened participants, i.e., in the re-screened participants only the chronologically last screening data is counted.

Randomized Analysis Set (RAS) - All randomized participants who received a randomization number, regardless of receiving trial medication.

Full Analysis Set (FAS) - All participants to whom study treatment has been assigned by randomization and who are not mis-randomized. Mis-randomized participants are those who have not been qualified for randomization, have been inadvertently randomized into the study and did not receive any double-blind study medication. According to the intent to treat principle, participants will be analyzed according to the treatment they have been assigned to during the randomization procedure.

Safety Set (SAF) - All randomized participants who received at least one dose of study treatment. Participants will be analyzed according to the study treatment received, where treatment received is defined as the randomized/assigned treatment if the participant took at least one dose of that treatment or the first treatment received if the randomized/assigned treatment was never received.

PK Analysis Set (PKS) - All randomized participants with at least one available valid (i.e., not flagged for exclusion) PK concentration measurement, who received any study XXB750 treatment.

Immunogenicity Prevalence Set (IPS) - All participants in the safety set with a non-missing baseline ADA sample **or** at least one non-missing post-baseline ADA sample.

Immunogenicity Incidence Set (IIS) - All participants in the safety set with a non-missing baseline ADA sample **and** at least one non-missing post-baseline ADA sample.

9.2 Statistical analyses

9.2.1 General considerations

Data analyses are described in Section 9 of the study protocol with more details available in statistical analysis plan (SAP).

Unless specified otherwise, baseline is defined as the measurement obtained at the randomization visit, or the measurement obtained at an earlier visit (scheduled or unscheduled) which was closest to the randomization visit, if the randomization visit measurement is missing.

All efficacy analyses will be based on FAS and all safety analyses will be based on SAF unless specified otherwise. In descriptive summaries, continuous variables will be summarized using n, mean, standard deviation (SD), median, minimum, 25th percentile (Q1), 75th percentile (Q3), and maximum; categorical variables will be summarized using frequency and percentage.

9.2.2 Participant demographics and other baseline characteristics

Demographic and other baseline data including disease history and standard background medications will be summarized descriptively by treatment group.

Relevant medical histories and current medical conditions at baseline will be summarized by system organ class and preferred term, by treatment group.

9.2.3 Treatments

The duration of exposure in day(s) to XXB750/matching placebo and sacubitril/valsartan as well as the dose intensity (computed as the ratio of actual cumulative dose received and actual duration of exposure) and the relative dose intensity (computed as the ratio of dose intensity and planned dose intensity) will be summarized by means of descriptive statistics and treatment group using the safety set.

Concomitant medications and significant non-drug therapies prior to and after the start of the study treatment will be summarized, by treatment group.

9.3 Primary endpoint(s)/estimand(s) analysis

Primary estimands are described in Section 3.1. The primary endpoint and statistical analysis will be described in this Section.

9.3.1 Definition of primary endpoint(s)

The primary efficacy variable is change in log NT-proBNP from baseline to Week 16, which is equivalent to the log of ratio of Week 16 NT-proBNP over baseline NT-proBNP.

Missing values at Week 16 will be imputed as described in Section 9.3.3 and Section 9.3.4.

9.3.2 Statistical model, hypothesis, and method of analysis

The primary objective of determination of a dose-response signal and to characterize the dose-response relationship in XXB750 doses compared to placebo will be evaluated using an optimally weighted contrast test following the Multiple Comparison Procedure-Modeling (MCP-MOD) methodology described in Pinheiro et al 2006 and Pinheiro et al 2014.

A candidate model set is defined corresponding to the range of the expected mean response in each of the dose groups. The candidate model set is used to generate a set of weights for the calculation of optimal contrasts between the responses in the studied dose groups and the placebo group. A statistical test comparing all doses in the different dose groups simultaneously to the placebo is used, hence a multiplicity adjustment is applied that accounts for the multiple possible dose response behavior considered as well as the common placebo between dose groups. A critical value is derived from a multivariate t-distribution using the correlation matrix induced by the correlations between the weights corresponding to the candidate sets as well as the correlation between the tests of shapes in the dosing groups to the placebo group.

Test of the dose response signal

The null hypothesis of a flat dose-response relationship for the change from baseline in logarithm NT-proBNP compared to placebo will be tested at a 1-sided significance level of 2.5% against the alternative hypothesis of a dose-response relationship leading to a significant reduction in the NT-proBNP.

Hence, the following null and alternative hypotheses will be tested:

- **H_{10}**: there is no dose-response relationship for XXB750 (i.e. the dose response relationship is flat).
- **H_{11}**: there is a dose-response relationship for XXB750 (i.e. there is more reduction in NT-proBNP in XXB750 doses based on the assumed candidate models).

There are five candidate models to capture the shape of the dose-response relationship for XXB750 at the Week 16 endpoint, as depicted in Figure 9-1. The candidate models generating the contrast weights are described below:

- Model 1: Emax with ED50 at 15 mg dose level
- Model 2: Emax with ED50 at 120 mg dose level
- Model 3: Sigmoid Emax with ED50 at 60 mg dose level and hill parameter h=3
- Model 4: Sigmoid Emax with ED50 at 120 mg dose level and hill parameter h=3
- Model 5: Beta-model with delta1=0.2, delta2=0.3 and scale=288

Figure 9-1 Dose-response curve of candidate models

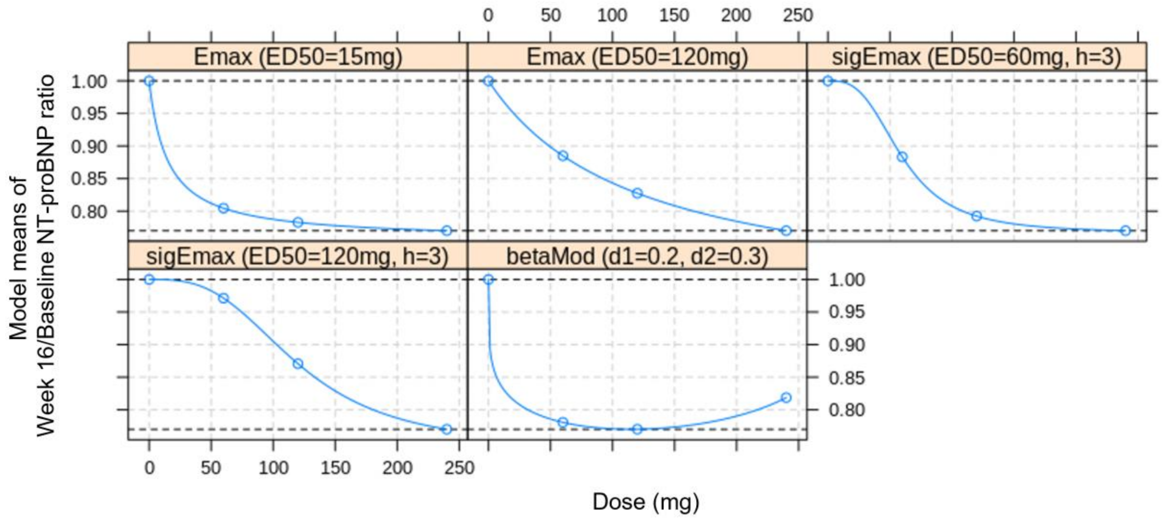

The analysis to derive the test statistics is based on an analysis of covariance (ANCOVA) model with the change from baseline logarithm of NT-proBNP to Week 16 as response variable, treatment (placebo and XXB750 dose groups), LVEF (≤ 40%, > 40%), background medication stratification factor (ACEI/ARB, sacubitril/valsartan), and region stratification factor as factors, and baseline logarithm of NT-proBNP as a covariate.

The response variable of the change in logarithm NT-proBNP from baseline at Week 16 used in the above ANCOVA model is from an imputed dataset, where a missing Week 16 NT-proBNP is imputed using the multiple imputation method as described in Section 9.3.3 and Section 9.3.4. In order to account for the imputation uncertainty, this ANCOVA model will be repeated for each imputed dataset, which results in a set of least squares (LS) mean estimate for all treatment groups and the related covariance matrices. Rubin's rule will be used to combine the multiple sets of LS mean estimates and the related covariance matrices to a single

set of LS mean estimates of change of NT-proBNP at Week 16 for all treatment groups and the related covariance matrix.

The optimal contrasts derived from the candidate model sets will be applied to the combined estimated dose means and covariance matrix to obtain t-statistics for each candidate model and the common critical value $C_{0.025}$. $C_{0.025}$ is the common critical value derived from the reference multivariate t-distribution with the 5x5 correlation matrix induced by test the candidate dose response models with respect to comparing all XXB750 dose groups to placebo.

The H_{10} will be rejected and the statistical significance of dose-response in NT-proBNP reduction is established if the max (t1, t2,..., t5) $\geq C_{0.025}$.

Model averaging to obtain the dose response

The response data in each imputed dataset, including relevant covariates, will be used to fit the models in the candidate set. The estimated dose-response will be derived by using model averaging methods on a subset of candidate models, for which the associated contrast tests are statistically significant. If there are more than three candidate models that are statistically significant, the top three models with largest t-statistics as calculated above will be selected as basis for the model averaging.

Model averaging will be carried out for each imputed dataset, and the resulting mean efficacy estimates and confidence intervals will be derived using the combination variance that accounts for the uncertainty of the imputed data using Rubin's rules. Comparisons between XXB750 doses and placebo will be simultaneously derived for the model averaged estimates together with confidence intervals reflecting the imputation procedure applied.

Selection of the optimal dose will be based on the model averaged dose response estimates of NT-proBNP reduction of XXB750 over the dose range studied.

Summaries of absolute values and change from baseline in NT-proBNP by treatment group and visit will be presented using statistics as described in Section 9.2.1 additionally with geometric mean and coefficient of variation. Figures will be produced to visually show the mean NT-proBNP level by visit over study period (randomized treatment period and safety follow-up period) for each treatment group.

The FAS will be used for the above analyses.

9.3.3 Handling of intercurrent events of primary estimand (if applicable)

Intercurrent events for the primary endpoint are defined in Section 3.1 and the strategy for handling of intercurrent events in the primary analysis is described below:

1. Discontinuation of study treatment: NT-proBNP data collected after discontinuation of study treatment will be used in the analysis (treatment policy strategy).
2. Unforeseen change in the dose of allowed concomitant medications: NT-proBNP data collected after unforeseen change in the dose of allowed concomitant medication will be used in the analysis (treatment policy strategy).
3. Receiving prohibited concomitant medication: NT-proBNP data collected after receiving prohibited concomitant medication will be used in the analysis (treatment policy strategy).

If a missing primary endpoint occurs after intercurrent event(s), it will be imputed based on participants with observed endpoint after same type of intercurrent event(s) in the respective treatment group. If there is no or very limited participants with observed endpoint after intercurrent event(s) in the respective treatment group, then the missing primary endpoint will be imputed based on the respective treatment group combined with lower dose group(s). if there are still very limited participants with observed endpoint after intercurrent event(s), then missing will be imputed based on those with observed endpoint in the placebo group.

9.3.4 Handling of missing values not related to intercurrent event

Missing data for the primary endpoint will be imputed using a multiple imputation approach assuming that missingness mechanism can be retrieved from observed data (missing at random (MAR)). The imputation model will include the longitudinal sequence of NT-proBNP data collected at randomization, Week 4, Week 8, Week 12, and Week 16 visits, LVEF factor (≤ 40%, > 40%), background medication stratification factor (ACEI/ARB, sacubitril/valsartan), region stratification factor, and other baseline variables as appropriate, imputing for each treatment group separately.

The full detailed information about the multiple imputation algorithms will be specified in a separate statistical analysis plan.

9.3.5 Multiplicity adjustment (if applicable)

Please refer to Section 9.3.2 for primary analysis of MCP-MOD for multiplicity adjustment with respect to testing for a dose response signal across multiple candidate shapes. The secondary hypotheses included in the order of the hierarchical testing procedure are an evaluation and demonstration of: (1) treatment effect of highest XXB750 target dose level versus placebo; (2) combined two highest XXB750 target dose levels versus conversion to sacubitril/valsartan in participants receiving ACEI/ARB background medication. The secondary hypotheses will be tested in the order described above and statistical inference will be made only if the primary hypothesis is rejected.

9.3.6 Sensitivity analyses

The primary analysis for which missing data imputation is based on missing at random or retrieve dropouts would favor the XXB750 treatment groups when early treatment/study discontinuation is due to XXB750 related adverse events or if the participant dies. Placebo multiple imputation method will be performed, when participants randomized to XXB750 treatment groups are assumed to be similar to placebo treated participants after above mentioned events.

9.3.7 Supplementary analysis

Supportive analyses for the primary analysis results are listed below:

1. Results based on the single best dose response model fit will also be reported.
2. The mixed model for repeated measure (MMRM) analysis of covariance (ANCOVA) with treatment, LVEF (≤40%, >40%), background medication stratification factor (ACEI/ARB, sacubitril/valsartan), and region stratification factor, visit (Week 4, Week 8, Week 12, or Week 16), and treatment-by-visit as factors, and baseline logarithm NT-proBNP as a

covariate, with a common unstructured covariance matrix among visits will be used. Treatment comparisons at Week 16 will be provided. The analysis will be based on all available data up to Week 16 in the FAS and based on likelihood method with an assumption of missing at random for missing data.

3. In addition to treatment policy strategy to handle the intercurrent events in the primary analysis, a supplemental analysis based on a hypothetical strategy will be also used to handle the intercurrent events. This strategy assumes that premature discontinuations of study treatment, change in the dose of allowed concomitant medication, receiving prohibited concomitant medication or lost to follow-up/discontinue study would not have occurred in the participants. This analysis uses partial data available up to the occurrence of the intercurrent events, and missing data after the intercurrent events will be imputed as based on the pattern of other participants who do not have intercurrent events (missing at random). The imputation steps are same as described in Section 9.3.4 and the newly imputed data will be used in the analysis as described in Section 9.3.2.
4. Analysis based on the while on-treatment strategy to handle the intercurrent of premature discontinuations of study treatment will be performed. This analysis uses the data taken at the end of treatment visit as the Week 16 assessment and repeat the analysis described in Section 9.3.2.
5. Treatment grouping is based on highest dose received and repeat the analysis described in Section 9.3.2.
6. Subgroup analyses will be performed for the primary analysis. The analysis will be done using the analysis specified in Section 9.3.2 at individual subgroup level separately. Please see Section 9.7 for subgroup details.

Summary statistics for NT-proBNP and change from baseline will be summarized by treatment group for observed and imputed values. Figures will be produced to visually show the observed and the imputed mean changes by visit over 16 weeks randomized treatment epoch for each treatment group. FAS will be used for the analyses.

9.4 Secondary endpoint(s)/estimand(s) analysis

The secondary efficacy endpoints are described in Section 9.4.1 and are part of the hierarchical testing strategy. Analyses related to safety endpoints are described in Section 9.4.2.

9.4.1 Efficacy and/or pharmacodynamic endpoint(s)

There are two efficacy secondary objectives:

1. To evaluate the treatment effect of the highest XXB750 target dose level compared to placebo in reducing NT-proBNP from baseline at Week 16 in HF patients treated with standard of care, including ACEI/ARB or sacubitril/valsartan.
2. To evaluate the treatment effect of combined two highest XXB750 target dose levels in addition to a background of ACEI/ARB versus conversion from ACEI/ARB to sacubitril/valsartan, in reducing NT-proBNP from baseline to Week 16.

The efficacy variable in secondary objective 1 is the change from baseline at Week 16 in logarithm of NT-proBNP and will be analyzed using a repeated measures ANCOVA model with treatment (highest XXB750 target dose level or placebo), LVEF (≤40%, >40%),

background medication stratification (ACEI/ARB, sacubitril/valsartan), and region stratification factor, visit (Week 4, Week 8, Week 12, or Week 16), and treatment-by-visit as factors, and baseline logarithm of NT-proBNP as a covariate, with a common unstructured covariance matrix among visits. Within treatment estimates and between treatment comparisons at Week 16 will be provided. Please refer to Section 3.2 and Section 9.3.3 for the intercurrent events and its handling strategy and Section 9.3.4 for handling missing data not related to intercurrent event. The analysis will be run on each of the imputed datasets as described in Section 9.3.2. Overall results are obtained by applying Rubin's rules on the estimates obtained from imputed datasets.

The efficacy variable in secondary objective 2 and analysis model is the same as those described above in secondary objective 1, except that treatments being compared are combined two highest XXB750 target dose levels with background medication of ACEI/ARB stratum versus conversion from ACEI/ARB to sacubitril/valsartan. In the event treatment arms 4a and 4b are eliminated, the comparison will be between only the 120 mg XXB750 and the sacubitril/valsartan treatment arms.

9.4.2 Safety endpoints

For all safety analyses, the safety set will be used unless specified otherwise. All listings and tables will be presented by treatment group.

Adverse events

All information obtained on adverse events will be displayed by treatment group and participant.

The number (and percentage) of participants with treatment emergent adverse events (events started after the first dose of study medication or events present prior to start of double-blind treatment but increased in severity based on preferred term) will be summarized in the following ways:

- by treatment, primary system organ class and preferred term.
- by treatment, primary system organ class, preferred term and maximum severity.
- by treatment, Standardized MedDRA Query (SMQ) and preferred term.

Separate summaries will be provided for study medication related adverse events, death, serious adverse events, other significant adverse events leading to discontinuation, and adverse events leading to dose adjustment.

The number (and proportion) of participants with adverse events of special interest and related to potential risks (as per the Investigator's Brochure) will be summarized by treatment.

A participant with multiple adverse events within a primary system organ class is only counted once towards the total of the primary system organ class.

Adverse events which will be counted for a specific treatment period are those which are treatment emergent. These events are those with an onset after the start of the treatment period, or which were present prior to the start of the treatment period but increased in severity, changed

from being not suspected to being suspected of study treatment relationship, or developed into SAEs after the start of the treatment period.

Vital signs

Summary statistics will be provided by treatment and visit for all vital signs, as well as for the proportion of participants presenting with clinically notable abnormal values. Graphical displays of the values of selected vital signs over time may be provided by treatment group.

More details on the analysis of vital signs will be described in the Statistical Analysis Plan (SAP).

12-lead ECG

A standard 12 lead ECG will be performed at Randomization, Week 16, and Week 24. Interpretation of the tracing must be made by a qualified physician and documented on the ECG section of the CRF. Each ECG tracing should be labeled with the study and subject number, date, and kept in the source documents at the study site. Clinically significant abnormalities should also be recorded on the Medical History/AE CRF page.

Summary Statistics for continuous data and number and percentage of participants for categorical data (e.g. interpretation) will be provided by treatment and visit.

More details on the analysis of ECG parameters will be described in the SAP.

Clinical laboratory evaluations

Laboratory data collected during the study will be summarized by presenting shift tables using extended normal ranges (baseline to most extreme post-baseline value), summary statistics of raw data and change from baseline by treatment and visit. The number of participants meeting pre-defined clinically notably laboratory values and percent changes will also be summarized by treatment.

Immunogenicity

All immunogenicity results including (the incidence of ADA and/or neutralizing antibodies) will be summarized by treatment and listed by treatment group, participant, and visit/time. IPS and IIS will be used for analyses.

More details on the analysis of immunogenicity parameters will be described in the SAP.

9.5 Exploratory endpoint(s)/estimand(s) analysis

Exploratory objectives are defined in Section 3.

Analyses of the exploratory efficacy variables will be based on the Full Analysis Set (FAS). Summary statistics by treatment group will be performed for the baseline values, the post-baseline values, and the change from baseline, with baseline defined as the randomization visit (or the measurement obtained at an earlier visit (scheduled or unscheduled) which is closest to the randomization visit, if the randomization visit measurement is missing). At each post baseline visit, only participants with a value at both baseline and that post-baseline visit will be

included in the change from baseline summary and missing post-baseline values will not be imputed.

9.5.1 Exploratory variables

Below are the variables for the exploratory analysis:

1. Number of winners based on the hierarchically ordered composite endpoint consisting of: 1) time to all-cause death; 2) number of HF hospitalizations; 3) categorical KCCQ CSS change; 4) NYHA class change; 5) relative change in NT-proBNP levels from baseline to Week 16;
2. Rate of change in eGFR (eGFR slope) from baseline to Week 16;
3. Change from baseline at assessment visits in biomarkers (details are provided in Section 9.5.4);
4. Change in NYHA class from baseline to Week 16;
5. Changes in HF signs and symptoms from baseline to Week 16;
6. Change in all domains of the Kansas City Cardiomyopathy Questionnaire from baseline to Week 16;
7. Population PK analysis results including mathematic models and parameters (details are provided in Section 9.5.3);
8. Incidence of anti-XXB750 antibodies (details are provided in Section 9.5.5).

9.5.2 Analysis methods

In general, exploratory variables will be analyzed in the FAS unless specified otherwise. Statistical tests will be performed at the two-sided significance level of 0.05. To better satisfy the normality assumption, the log-transformation will be taken on each biomarker prior to statistical analysis. There will be no multiplicity adjustment for any analysis of exploratory variables.

Win ratio analysis

The hierarchical ordered composite endpoint will be analyzed using the win ratio approach (Pocock et al 2012), with treatment as fixed-effect factor and stratified by region and background medication (ACEI/ARB, sacubitril/valsartan) (Dong et al 2018). Every participant in the highest XXB750 target dose level group will be compared to every participant in the placebo group within each stratum in the following order:

1. all pairs will be compared first by time to all-cause death; if there is no winner, then
2. they will be compared for the number of HF hospitalizations; if there is no winner, then
3. they will be compared according to the combination of change in KCCQ CSS and change in NYHA class from baseline to Week 16; if still there is no winner, then
4. they will be compared according to reduction in NT-proBNP levels from baseline to Week 16.
5. If the comparison between changes in NT-proBNP levels does not result in a winner, the respective pairwise comparison is tied.

When comparing the time to all-cause death and number of HF hospitalizations between two participants and at least one of the times is censored, the comparison is only performed up to the censoring time. Three categories are defined for the change of KCCQ CSS: worsened (decrease of 5 points or more), improved (increase of 5 points or more), or unchanged (neither worsened nor improved). The comparison of KCCQ CSS categorical change is in favor of the participant in the highest XXB750 arm if the category of the participant is better compared to that in placebo arm. Similarly, three categories are defined for the change of NYHA class, worsened, improved, or unchanged; and the comparison of NYHA class change is in favor of the participant in the highest XXB750 arm if the class change of the participant is better compared to that in the placebo arm. Please refer to Table 9-1 for the example of combination of change in KCCQ CSS and change in NYHA class.

Table 9-1 Selecting a winner based on combination of change of KCCQ CSS and change of NYHA class

Example	Participant A		Participant B		Result of comparison
	Change of KCCQ CSS from baseline to Week 16	Change of NYHA Class from baseline to Week 16	Change of KCCQ CSS from baseline to Week 16	Change of NYHA Class from baseline to Week 16	
1	Improved	Improved	Improved	Improved	Tie
2	Improved	Worsened	Worsened	Improved	Tie
3	Worsened	Unchanged	Worsened	Worsened	A wins
4	Worsened	Worsened	Worsened	Improved	B wins

The comparison of NT-proBNP is performed based on the relative change between baseline and Week 16 and takes into account that relative changes within ±25% are considered medically irrelevant; in detail, let μ_{XXB750} and $\mu_{placebo}$ be the relative changes of participants in the highest XXB750 and the placebo arm, respectively. Then, a comparison of NT-proBNP is in favor of the participant in the highest XXB750 arm if $\mu_{XXB750} < 0.75\ \mu_{placebo}$. On the other hand, the comparison is in favor of the participant in the placebo arm if $\mu_{placebo} < 0.75\ \mu_{XXB750}$.

The win ratio is estimated by the ratio of the number of winners in the highest XXB750 treatment group and the number of winners in the placebo group with weights defined as the reciprocal of the stratum sample size.

$$\Psi = \frac{\sum_{m=1}^{M} w^{(m)} n_{XXB750}^{(m)}}{\sum_{m=1}^{M} w^{(m)} n_{placebo}^{(m)}}$$

where $n_{XXB750}^{(m)}$ and $n_{placebo}^{(m)}$ are the total of wins in the mth stratum for the highest XXB750 and placebo treatment groups, respectively; $w^{(m)}=1/N^{(m)}$ and $N^{(m)}$ is the total sample size in the mth stratum.

The estimated win ratio and the corresponding 2-sided 95% CI will be provided. The contribution to the number of winners of each component of the win ratio will be summarized.

Analysis of continuous variables

Continuous variables, which are assumed to be normally distributed, are:

- Rate of change (slope) in eGFR from baseline at Week 16;
- Change in KCCQ from baseline at Week 16.

For the rate change in eGFR, the eGFR slope will be estimated from a repeated measures ANCOVA model including treatment, LVEF (≤40%, >40%), background medication stratification factor (ACEI/ARB, sacubitril/valsartan), region stratification factor, time (when the eGFR is assessed in weeks), and treatment-by-time as fixed effects with random intercept and slope (time) and a common unstructured covariance. The least-square means of slopes for within and between treatment groups, and the corresponding two-sided 95% confidence interval will be provided.

Changes from baseline in KCCQ scores will be analyzed based on an ANCOVA model in which treatment, LVEF (≤40%, >40%), background medication stratification factor (ACEI/ARB, sacubitril/valsartan) and region stratification factor will be included as factors and baseline value as a covariate. Treatment comparisons and effect size estimates at Week 16 will be provided. The analysis will be performed based on all available data up to Week 16 in the FAS.

Analysis of ordinal variables

The ordinal variables include

- Change in NYHA class from baseline;
- Changes in HF signs and symptoms from baseline.

The ordinal variables will be analyzed, at Week 4, Week 8, Week 12, and Week 16, using a repeated measures proportional cumulative odds model. The model will include patient as a random effect and the respective baseline category, treatment, LVEF (≤40%, >40%), background medication stratification factor (ACEI/ARB, sacubitril/valsartan), region stratification factor, visit (all available post-randomization visits) and treatment-by-visit interaction as fixed effect factors. This model assumes that the treatment effect sizes across measurement categories are the same. The visit-wise effect size estimates and their 95% confidence intervals will also be provided. The analysis will be based on all available data in the FAS and likelihood method with an assumption of missing at random for missing data.

For population PK analysis and incidence of anti-XXB750 antibodies, please refer to Section 9.5.3 and Section 9.5.5, respectively.

Analysis methods for biomarkers are described in Section 9.5.4 with more details available in the statistical analysis plan (SAP).

All exploratory endpoints will be summarized over 16 weeks randomized treatment epoch for each treatment group. Additional summary over safety follow-up will be provided as appropriate. FAS will be used for analyses.

9.5.3 Pharmacokinetics

Sparse PK samples are collected for population PK analysis. Results will be reported separately. Individual drug concentration will be listed in Clinical Study Report (CSR).

9.5.4 Biomarkers

Both blood and urine samles will be collected at all sites where geographically feasible at Randomization, Week 8, Week 12, and Week 16 as described in Table 1-1. Biomarkers to be analyzed may include, but are not necessarily limited to: cyclic GMP (plasma and urine), urinary cGMP-to-creatinine ratio, urinary albumin-to-creatinine ratio, BNP and hs-Troponin (in addition to primary endpoint NT-proBNP).

The change from baseline to a pre-defined time-point in logarithmic scale will be analyzed using a repeated measures ANCOVA model with treatment, LVEF (≤40%, >40%), background medication stratification factor (ACEI/ARB, sacubitril/valsartan), and region stratification factor, visit (Week 8, Week 12, or Week 16), and treatment-by-visit as factors, and respective baseline in logarithm scale as a covariate, with a common unstructured covariance matrix among visits will be used. Estimates of treatment difference and their two-sided 95% confidence intervals will be provided. These estimates and confidence intervals will be back-transformed for presentation.

In addition, biomarker data will be summarized by treatment and time point. Further displays of biomarker data may be considered and will be described separately in the Statistical Analysis Plan (SAP).

9.5.5 Immunogenicity

All immunogenicity results including the incidence of ADA and/or neutralizing antibodies will be summarized by treatment and listed by treatment group, participant, and visit/time. IPS and IIS will be used for analyses.

More details on the analysis of immunogenicity parameters will be described in the SAP.

9.5.6 Patient reported outcomes

The change in HF symptoms and physical limitation scores assessed by the clinical summary score of the KCCQ and the change in other domains of KCCQ is one of the exploratory efficacy endpoints. Please refer to Section 9.5.2 for the analysis method.

9.6 Other safety analyses

Please refer to statistical analysis plan for details.

9.7 Other analyses

9.7.1 Subgroup analyses

The subgroups of interest for primary analysis and selected secondary/exploratory analyses include but are not limited to:

- Age (< 65, ≥ 65)
- Sex (female, male)
- Region
- LVEF (≤ 40%, > 40%)

- Type of RAS inhibition (ACEI/ARB, sacubitril/valsartan)
- Dose level of ACEI/ARB or sacubitril/valsartan at randomization.

Please refer to statistical analysis plan for more details.

9.7.2 DNA

Exploratory DNA studies are designed to investigate the association between genetic factors (genotypes) and clinical assessments (phenotypes) which are collected during the clinical trial. Without prior evidence of a strong association, a number of possible associations are evaluated with exploratory analyses. A range of statistical tests are used for the analyses. Additional data, from other clinical trials, are often needed to confirm associations. Alternatively, if the number of participants enrolled in the study is too small to complete proper statistical analyses, the data may be combined, as appropriate, with those from other studies to enlarge the dataset for analysis.

Data generated on hypothesis-free platforms will be reported separately outside of CSR.

9.8 Interim analysis

One safety interim analysis will be conducted when data from the ongoing XXB750 phase 1b study CXXB750A12101 becomes available. This analysis will include all available data of the study CXXB750A12101 and treatment arms 1-3 and 5 of the current study to assess the need for adapting the design of the current study.

An additional administrative interim analysis informing other programs may be conducted when approximately 430 participants are randomized into the study (approximately 40 participants each in arms 1-4) and are followed for 16 weeks to help the planning of another trial in a different development program. The results of this interim analysis will not lead to any changes in this study and will be kept blinded to anyone directly involved in the conduct of the study. More details about the DMC review process and how unblinding will be handled in the context of DMC reviews will be provided in the DMC charter.

No alpha adjustment will be made for the interim safety analysis, safety data monitoring, and the administrative interim analysis. All interim analyses and safety data monitoring will be performed by an independent statistician who will not be involved in the trial conduct. The results will be reviewed by the independent DMC.

The primary analysis will be performed after all participants have completed Week 16 (or discontinued prior to Week 16). A final analysis will be performed after all participants completed Week 24 (or discontinued prior to Week 24). Formal testing of the primary endpoint with full level alpha of 0.025 (1-sided) will be performed at the primary analysis time point.

Regular safety data monitoring, including the safety interim analysis and the administrative interim analysis, is planned as specified in Section 4.6.

9.9 Sample size determination

The study is planned to randomize 720 participants in total.

9.9.1 Primary endpoint(s)

For the primary endpoint analysis, 600 participants allocated in the ratio of 2:2:3:3 to placebo, XXB750 60 mg dose level, XXB750 120 mg dose level, and highest XXB750 dose level, respectively.

Assuming a common standard deviation of 0.67 for log NT-proBNP change from baseline and a one-sided 2.5% significance level (with adjustments for multiple comparisons using MCP-MOD), a sample size of 600 participants (120 in placebo, 120 in XXB750 60 mg dose level, 180 in XXB750 120 mg dose level, and total of 180 in highest XXB750 dose level) or, in the event treatment arms 4a and 4b are eliminated (see Section 4.6), a sample size of 540 participants (120 in placebo, 120 in XXB750 60 mg dose level, and 300 in XXB750 120 mg dose level) will provide a power of at least 90% if the underlying true maximum NT-proBNP reduction on XXB750 vs. placebo is 23%.

9.9.2 Secondary endpoint(s)

A common standard deviation of 0.67 for log NT-proBNP change from baseline and a one-sided 2.5% significance level is assumed for all sample size determinations relating to secondary endpoints.

For the first secondary endpoint, a sample size of 120 participants in placebo and 180 participants in XXB750 optimal dose level, respectively, will provide a minimum power of 90% if the true NT-proBNP reduction on XXB750 vs. placebo is 23%. In the case of when treatment arms 4a and 4b are eliminated after safety interim analysis, a sample size of 120 participants in placebo and 300 participants in XXB750 120 mg dose level will provide a power of approximately 95% if the true NT-proBNP reduction on XXB750 vs. placebo is 23%.

For the second secondary endpoint, in participants with ACEI/ARB background medication converted to sacubitril/valsartan, a sample size of 120 participants in a group combining the two highest XXB750 target dose levels and 120 participants in sacubitril/valsartan conversion arm will provide approximately 80% power if the underlying true maximum NT-proBNP reduction with adding XXB750 vs. conversion to sacubitril/valsartan is 22%. In the case when treatment arms 4a and 4b are eliminated, a sample size of 100 participants in XXB750 120 mg dose level and 120 participants in sacubitril/valsartan conversion arm will provide approximately 77% power if the underlying true maximum NT-proBNP reduction with adding XXB750 vs. conversion to sacubitril/valsartan is 22%.

10 Supporting documentation and operational considerations

10.1 Appendix 1: Regulatory, ethical, and study oversight considerations

10.1.1 Regulatory and ethical considerations

This study will be conducted in accordance with the protocol and with the following:

- Consensus ethical principles derived from international guidelines including the Declaration of Helsinki and Council for International Organizations of Medical Sciences (CIOMS) international ethical guidelines
- Applicable ICH Good Clinical Practice (GCP) guidelines
- Applicable laws and regulations

The protocol, protocol amendments, ICF, Investigator's Brochure, [IDFU], and other relevant documents (e.g. advertisements) must be submitted to an IRB/IEC by the Investigator and reviewed and approved by the IRB/IEC before the study is initiated.

Any amendments to the protocol will require IRB/IEC approval before implementation of changes made to the study design, except for changes necessary to eliminate an immediate hazard to study participants.

Protocols and any substantial amendments/modifications to the protocol will require health authority approval prior to initiation except for changes necessary to eliminate an immediate hazard to study participants.

The Investigator will be responsible for the following:

Signing a protocol signature page confirming his/her agreement to conduct the study in accordance with these documents and all of the instructions and procedures found in this protocol and to give access to all relevant data and records to Novartis monitors, auditors, Novartis Quality Assurance representatives, designated agents of Novartis, IRBs/IECs, and regulatory authorities as required

Providing written summaries of the status of the study to the IRB/IEC annually or more frequently in accordance with the requirements, policies, and procedures established by the IRB/IEC

Notifying the IRB/IEC of SAEs or other significant safety findings as required by IRB/IEC procedures

Providing oversight of the conduct of the study at the site and adherence to requirements of 21 CFR, ICH guidelines, the IRB/IEC, European regulation 536/2014 for clinical studies (if applicable), European Medical Device Regulation 2017/745 for clinical device research (if applicable), and all other applicable local regulations

Inform Novartis immediately if an inspection of the clinical site is requested by a regulatory authority.

This clinical study was designed and shall be implemented, executed and reported in accordance with the ICH Harmonized Tripartite Guidelines for Good Clinical Practice, with applicable local regulations (including European Directive 2001/20/EC or European Clinical Trial Regulation 536/2014, US CFR 21), and with the ethical principles laid down in the Declaration of Helsinki.

10.1.2 Informed consent process

The Investigator or his/her representative will explain the nature of the study, including the risks and benefits, to the patient or their legally authorized representative and answer all questions regarding the study.

Patients must be informed that their participation is voluntary. Patients or their legally authorized representatives will be required to sign a statement of informed consent that meets the requirements of 21 CFR 50, local regulations, ICH guidelines, privacy and data protection requirements, where applicable, and the IRB/IEC or study center.

Informed consent must be obtained before conducting any study-specific procedures (e.g. all of the procedures described in the protocol). The process of obtaining informed consent must be documented in the participant source documents.

The medical record must include a statement that written informed consent was obtained before the participant was enrolled in the study and the date the written consent was obtained. The authorized person obtaining the informed consent must also sign the ICF.

A copy of the ICF(s) must be provided to the participant or their legally authorized representative.

Patients who are re-screened are required to sign a new ICF.

The ICF will contain a separate section that addresses the use of remaining mandatory samples for optional additional research, e.g. genetic analysis. The Investigator or authorized designee will explain to each participant the objectives of the additional research. Participants will be told that they are free to refuse to participate and may withdraw their consent at any time and for any reason during the storage period. A separate signature will be required to document a participant's agreement to allow any remaining specimens to be used for additional research. Participants who decline to participate in this optional additional research will document this.

Eligible patients may only be included in the study after providing (witnessed, where required by law or regulation), IRB/IEC-approved informed consent.

If applicable, in cases where the participant's representative(s) gives consent (if allowed according to local requirements), the participant must be informed about the study to the extent possible given his/her level of understanding. If the participant is capable of doing so, he/she must indicate agreement by personally signing and dating the written informed consent document.

Information about common side effects already known about the investigational treatment can be found in the Investigator's Brochure (IB). This information will be included in the participant informed consent and should be discussed with the participant upon obtaining consent and also during the study as needed. Any new information regarding the safety profile of the investigational drug that is identified between IB updates will be communicated as appropriate, for example, via an Investigator notification or an aggregate safety finding. New information might require an update to the informed consent and then must be discussed with the participant.

The following informed consents are included in this study:

- Main study consent, which also included:

    - A subsection that requires a separate signature for the 'Optional Consent for Additional Research' to allow future research on data/samples collected during this study
    - Optional consent for activities that may be done outside of the study site
- As applicable, Pregnancy Outcomes Reporting Consent for female participants or the female partners of any male participants who took study treatment
- Patient information sheet for female partners of any male participants who took study treatment
- Optional Genetics Consent to provide a sample for exploratory DNA studies

The study includes an optional sub-study/ DNA component which requires a separate signature if the participant agrees to participate. It is required as part of this protocol that the Investigator presents this option to the participants, as permitted by local governing regulations. The process for obtaining consent should be exactly the same as described above for the main informed consent.

Declining to participate in these optional assessments (DNA) will in no way affect the participant's ability to join the main research study.

A copy of the approved version of all consent forms must be provided to Novartis after IRB/IEC approval.

As per Section 4.5, during a public health emergency as declared by local or regional authorities i.e. pandemic, epidemic or natural disaster, that may challenge the ability to obtain a standard written informed consent due to limits that prevent an on-site visit, Investigator may conduct the informed consent discussion remotely (e.g. telephone, videoconference) if allowable by a local health authority.

Guidance issued by local regulatory bodies on this aspect prevail and must be implemented and appropriately documented (e.g. the presence of an impartial witness, sign/dating separate ICFs by trial participant and person obtaining informed consent, etc.).

10.1.3 Data protection

Participants will be assigned a unique identifier by Novartis. Any participant records or datasets that are transferred to Novartis will contain the identifier only; participant names or any information which would make the participant identifiable will not be transferred.

The participant must be informed that his/her personal study-related data will be used by Novartis in accordance with local data protection law. The level of disclosure must also be explained to the participant who will be required to give consent for their data to be used as described in the informed consent.

The participant must be informed that his/her medical records may be examined by Clinical Quality Assurance auditors or other authorized personnel appointed by Novartis, by appropriate IRB/IEC members, and by inspectors from regulatory authorities.

Novartishas appropriate processes and policies in place to handle personal data breaches according to applicable privacy laws.

10.1.4 Committees structure

In this study, an Executive Committee will support development and conduct of the study, while an independent, program-wide Data Monitoring Committee will oversee patient safety and frequently review patient safety data as outlined below.

10.1.4.1 Data Monitoring Committee

This study will include a data monitoring committee (DMC) which will function independently of all other individuals associated with the conduct of this clinical trial, including the site investigators participating in the study. The DMC will assess at defined intervals the progress of a clinical trial, safety data, and critical efficacy variables and recommend to Novartis whether to continue, modify, or terminate a trial.

At the time of the interim analysis for safety in order to open enrollment into treatment arms 4a and 4b (see Section 4.6), the DMC will have 3 options to recommend based on the safety profile:

- To continue the study as per protocol (i.e. to open enrollment into both arms 4a and 4b as planned).
- To not open both treatment arms 4a and 4b, or to not open treatment arm 4b.
- To replace the originally planned 240 mg target dose with a 180 mg target dose as the highest target dose (at either low and high starting dose or only a low starting dose).

The DMC may also conduct a planned administrative interim analysis to inform other programs. Details on the DMC early safety interim analysis and the administrative interim analysis to inform other programs are described in Section 4.6 and Section 9.8.

More details regarding composition, responsibilities, data monitoring, and meeting frequency, and documentation of DMC reports, minutes, and recommendations will be described in a separate charter that is established between Novartis and the DMC.

10.1.4.2 Executive Committee

The Executive Committee (EC) will be established comprising of outside medical experts i.e. not being members of the DMC and Novartis representatives from the Clinical Trial Team.

The EC will ensure transparent management of the study according to the protocol through recommending and approving modifications as circumstances require. The EC will review protocol amendments as appropriate. Together with the clinical trial team, the EC will also develop recommendations for publications of study results including authorship rules.

10.1.5 Data quality assurance

Monitoring details describing strategy, including definition of study critical data items and processes (e.g. risk-based initiatives in operations and quality such as risk management and mitigation strategies and analytical risk-based monitoring), methods, responsibilities, and requirements, including handling of noncompliance issues and monitoring techniques (central, remote, or on-site monitoring) are provided in the monitoring plan.

Records and documents, including signed ICFs, pertaining to the conduct of this study must be retained by the Investigator for 15 years after study completion unless local regulations or institutional policies require a longer retention period. No records may be destroyed during the retention period without the written approval of Novartis. No records may be transferred to another location or party without written notification to Novartis.

10.1.5.1 Data collection

Designated Investigator staff will enter the data required by the protocol into the Electronic Case Report Forms (eCRF). The eCRFs have been built using fully validated secure web-enabled software that conforms to 21 CFR Part 11 requirements. Investigator site staff will not be given access to the EDC system until they have been trained. Automatic validation programs check for data discrepancies in the eCRFs, allow modification and/or verification of the entered data by the Investigator staff.

The Investigator/designee is responsible for assuring that the data (recorded on CRFs) (entered into eCRF) is complete, accurate, and that entry and updates are performed in a timely manner. The Investigator must certify that the data entered are complete and accurate.

After final database lock, the Investigator will receive copies of the participant data for archiving at the investigational site.

All data should be recorded, handled, and stored in a way that allows its accurate reporting, interpretation, and verification.

10.1.5.2 Database management and quality control

Novartis personnel (or designated CRO) will review the data entered by investigational staff for completeness and accuracy. Electronic data queries stating the nature of the problem and requesting clarification will be created for discrepancies and missing values and sent to the investigational site via the EDC system. Designated Investigator site staff are required to respond promptly to queries and to make any necessary changes to the data.

Concomitant treatments and prior medications entered into the database will be coded using the World Health Organization (WHO) Drug Reference List, which employs the Anatomical Therapeutic Chemical classification system. Medical history/current medical conditions and adverse events will be coded using the Medical Dictionary for Regulatory Activities (MedDRA) terminology.

Dates of screenings, randomizations, screen failures and study completion, as well as randomization codes and data about all study treatment (s) dispensed to the participant and all dosage changes will be tracked using an Interactive Response Technology (IRT). The system will be supplied by a vendor, who will also manage the database. The data will be sent electronically to Novartis (or a designated CRO) at specific timelines.

Each occurrence of a code break via IRT will be reported to the clinical team and monitor. The code break functionality will remain available until study shut down or upon request of Novartis.

Once all the necessary actions have been completed and the database has been declared to be complete and accurate, it will be locked **and the treatment codes will be unblinded** and made

available for data analysis for the CSR at the conclusion of the study. Any changes to the database after that time can only be made after written agreement by Novartis development management.

10.1.6 Source documents

Source documents provide evidence for the existence of the participant and substantiate the integrity of the data collected. Source documents are filed at the Investigator's site.

Data reported on the CRF or entered in the eCRF that are transcribed from source documents must be consistent with the source documents or the discrepancies must be explained. The Investigator may need to request previous medical records or transfer records, depending on the study. Also, current medical records must be available.

The Investigator must give the monitor access to all relevant source documents to confirm their consistency with the data capture and/or data entry. The Investigator must also keep the original informed consent form signed by the participan*t* (a signed copy is given to the participant). Definition of what constitutes source data and its origin can be found in monitoring guidelines.

The Investigator must maintain accurate documentation (source data) that supports the information entered in the CRF. Study monitors will perform ongoing source data verification to confirm that data entered into the CRF by authorized site personnel are accurate, complete, and verifiable from source documents; that the safety and rights of participants are being protected; and that the study is being conducted in accordance with the currently approved protocol and any other study agreements, ICH GCP, and all applicable regulatory requirements.

Key study personnel must be available to assist the field monitor during all visits. Continuous remote monitoring of each site's data may be performed by a centralized Novartis / CRA organization. Additionally, a central analytics organization may analyze data & identify risks & trends for site operational parameters, and provide reports to Novartis clinical teams to assist with trial oversight.

10.1.7 Publication policy

The protocol will be registered in a publicly accessible database such as clinicaltrials.gov and as required in EudraCT . In addition, after study completion and finalization of the study report the results of this trial will be submitted for publication and posted in a publicly accessible database of clinical trial results, such as the Novartis clinical trial results website and all required health authority websites (e.g. Clinicaltrials.gov, EudraCT etc.).

For details on the Novartis publication policy including authorship criteria, please refer to the Novartis publication policy training materials that were provided to you at the trial Investigator meetings.

Any data analysis carried out independently by the Investigator should be submitted to Novartis before publication or presentation.

Summary results of primary and secondary endpoints will be disclosed based upon the global Last Participant Last Visit (LPLV) date, since multinational studies are locked and reported based upon the global LPLV.

10.1.8 Protocol adherence and protocol amendments

This protocol defines the study objectives, the study procedures and the data to be collected on study participants. Additional assessments required to ensure safety of participants should be administered as deemed necessary on a case by case basis. Under no circumstances including incidental collection is an Investigator allowed to collect additional data or conduct any additional procedures for any purpose involving any investigational drugs under the protocol, other than the purpose of the study. If despite this interdiction prohibition, data, information, observation would be incidentally collected, the Investigator shall immediately disclose it to Novartis and not use it for any purpose other than the study, except for the appropriate monitoring on study participants.

Investigators ascertain they will apply due diligence to avoid protocol deviations. If an Investigator feels a protocol deviation would improve the conduct of the study this must be considered a protocol amendment, and unless such an amendment is agreed upon by Novartis and approved by the IRB/IEC and health authorities, where required, it cannot be implemented.

10.1.8.1 Protocol amendments

Any change or addition to the protocol can only be made in a written protocol amendment that must be approved by Novartis, health authorities where required, and the IRB/IEC prior to implementation.

Only amendments that are required for participant safety may be implemented immediately provided the health authorities are subsequently notified by protocol amendment and the reviewing IRB/IEC is notified.

Notwithstanding the need for approval of formal protocol amendments, the Investigator is expected to take any immediate action required for the safety of any participant included in this study, even if this action represents a deviation from the protocol. In such cases, Novartis should be notified of this action and the IRB/IEC at the study site should be informed according to local regulations.

10.2 Appendix 2: Abbreviations and definitions

10.2.1 List of abbreviations

ACEI(s)	Angiotensin converting enzyme inhibitor(s)
AE	Adverse Event
AESI	Adverse Events of Special Interest
ALP	Alkaline Phosphatase
ALT	Alanine Aminotransferase
ANA	Antinuclear Antibodies
ANCOVA	Analysis of covariance
ARB(s)	Angiotensin receptor blocker(s)
ARNI	Angiotensin receptor neprilysin inhibitor
ASCT	Autologous Stem Cell Transplant
ASMA	Anti-smooth muscle antibody
AST	Aspartate Aminotransferase
AV	Atrio-ventricular
AxMP	Auxiliary Medicinal Product
bid	bis in die/twice a day
BLRM	Bayesian Logistic Regression Model
BMA	Bone Modifying Agents
BMI	Body Mass Index
BP	Blood pressure
BUN	Blood Urea Nitrogen
CABG	Coronary artery bypass graft
CDS	Core Data Sheet
cGMP	Cyclic guanosine monophosphate
CK	Creatine Kinase
CO	Country Organization
COA	Clinical Outcome Assessment
CQA	Clinical Quality Assurance
CRF	Case Report/Record Form (paper or electronic)
CRO	Contract Research Organization
CRT	Cardiac resynchronization therapy
CSR	Clinical study report
CTT	Clinical Trial Team
CV	Cardiovascular
DBP	Diastolic Blood Pressure
DIN	Drug Inducted Nephrotoxicity
DLT	Dose Limiting Toxicity
DMC	Data Monitoring Committee
ECG	Electrocardiogram
EDC	Electronic Data Capture
eGFR	Estimated glomerular filtration rate
EOS	End of study
EOT	End of treatment

ERCP	Endoscopic retrograde cholangiopancreatography
eSAE	Electronic Serious Adverse Event
eSource	Electronic Source
FDA	Food and Drug Administration
FIH	First in Human
FSH	Follicle Stimulating Hormone
GCP	Good Clinical Practice
GCS	Global Clinical Supply
GGT	Gamma-glutamyl transferase
GLDH	Glutamate Dehydrogenase
GMP	Good Manufacturing Practice
h	Hour
HBsAg	Hepatitis B virus surface antigen
HBV	Hepatitis B Virus
HCV	Hepatitis C Virus
HED	Human Equivalent Dose
HF	Heart failure
HFmrEF	Heart failure with mildly reduced ejection fraction
HFrEF	Heart failure with reduced ejection fraction
HSCT	Hematopoietic Stem Cell Transplant
i.v.	intravenous
IB	Investigator's Brochure
ICF	Informed Consent Form
ICH	International Council for Harmonization of Technical Requirements for Pharmaceuticals for Human Use
IDFU	Investigational directions for use
IEC	Independent Ethics Committee
IN	Investigator Notification
INR	International Normalized Ratio
IRB	Institutional Review Board
IRT	Interactive Response Technology
KCCQ	Kansa City Cardiomyopathy Questionnaire
LCZ696	Sacubitril/valsartan
LDH	lactate dehydrogenase
LFT	Liver function test
LLN	lower limit of normal
LLOQ	lower limit of quantification
LVEF	Left ventricular ejection fraction
MedDRA	Medical dictionary for regulatory activities
mg	milligram(s)
mL	milliliter(s)
MRA(s)	Mineralcorticoid receptor antagonist(s)
Nab	Neutralizing antibody
NCDS	Novartis Clinical Data Standards
NP(s)	Natriuretic petide(s)
NPR1	Natriuretic Peptide Receptor 1

NT-proBNP	N-terminal prohormone B-type natriuretic peptide
NYHA	New York Heart Association
OHP	Off-site Healthcare Professional
p.o.	Oral(ly)
PA	Posteroanterior
PCI	Percutaneous coronary intervention
PD	Pharmacodynamic(s)
PK	Pharmacokinetic(s)
PKS	PK analysis set
POCT	Point of Care Testing
PRO	Patient Reported Outcomes
PS&PV	Patient Safety and Pharmacovigilance
PSD	Premature Subject Discontinuation
PT	prothrombin time
q4w	Every 4 weeks
QD	Once a day
QoL	Quality of life
R Value	ALT/ALP x ULN
RAAS	Renin-angiotensin-aldosterone system
RD	Recommended Dose
RU	Resource Utilization
s.c.	subcutaneous
SAE	Serious Adverse Event
SAP	Statistical Analysis Plan
SBP	Systolic Blood Pressure
SD	standard deviation
SGLT2I(s)	Sodium Glucose Co-transporter-2 Inhibitor(s)
SGOT	Serum Glutamic Oxaloacetic Transaminase
SGPT	Serum Glutamic Pyruvic Transaminase
SoA	Schedule of Activities
SUSAR	Suspected Unexpected Serious Adverse Reaction
TBL	Total bilirubin level
ULN	upper limit of normal
WHO	World Health Organization

10.2.2 Definitions

Additional treatment	Medicinal products that may be used during the clinical trial as described in the protocol, but not as an investigational medicinal product (e.g. any background therapy)
Assessment	A procedure used to generate data required by the study
Auxiliary medicinal product	Medicinal product used for the needs of a clinical trial as described in the protocol, but not as an investigational medicinal product (e.g. rescue medication, challenge agents, background treatment or medicinal products used to assess end-points in the clinical trial).<br>Concomitant therapy is not considered as AMP.
Biologic Samples	A biological specimen including, for example, blood (plasma, serum), saliva, tissue, urine, stool, etc. taken from a study participant
Clinical Outcome Assessment (COA)	A measure that describes or reflects how a participant feels, functions, or survives
Clinical Trial Team	A group of people responsible for the planning, execution and reporting of all clinical trial activities. Examples of team members include the Study Lead, Medical Monitor, Trial Statistician etc.
Coded Data	Personal Data which has been de-identified by the investigative center team by replacing personal identifiers with a code.
Discontinuation from study	Point/time when the participant permanently stops receiving the study treatment and further protocol required assessments or follow-up, for any reason. No specific request is made to stop the use of their samples or data.
Discontinuation from study treatment	Point/time when the participant permanently stops receiving the study treatment for any reason (prior to the planned completion of study intervention administration, if any). Participant agrees to the other protocol required assessments including follow-up. No specific request is made to stop the use of their samples or data.
Dosage	Dose of the study treatment given to the participant in a time unit (e.g. 100 mg once a day, 75 mg twice a day)
Electronic Data Capture (EDC)	Electronic data capture (EDC) is the electronic acquisition of clinical study data using data collection systems, such as Web-based applications, interactive voice response systems and clinical laboratory interfaces. EDC includes the use of Electronic Case Report Forms (eCRFs) which are used to capture data transcribed from source data/documents used at the point of care
End of the clinical trial	The end of the clinical trial is defined as the last visit of the last participant.
Enrollment	Point/time of participant entry into the study at which informed consent must be obtained. The action of enrolling one or more participants
eSource (DDE)	eSource Direct Data Entry (DDE) refers to the capture of clinical study data electronically, at the point of care. eSource Platform/Applications combines source documents and case report forms (eCRFs) into one application, allowing for the real time collection of clinical trial information to Novartis and other oversight authorities, as appropriate
Estimand	As defined in the ICH E9(R1) addendum, estimand is a precise description of the treatment effect reflecting the clinical question posed by the trial objective. It summarizes at a population-level what the outcomes would be in the same participants under different treatment conditions being compared. Attributes of an estimand include the population, variable (or endpoint) and treatment of interest, as well as the specification of how the remaining intercurrent events are addressed and a population-level summary for the variable.
Intercurrent events	Events occurring after treatment initiation that affect either the interpretation or the existence of the measurements associated with the clinical question of interest.
Investigational drug/ treatment	The drug whose properties are being tested in the study
Investigational Product/	A pharmaceutical form of an active ingredient or placebo being tested or used as a reference (such as an active comparator) in a clinical trial, including a product with a

Investigational Medicinal product	marketing authorization when used or assembled (formulated or packaged) in a way different from the approved form, or when used for an unapproved indication, or when used to gain further information about an approved use.
Medication number	A unique identifier on the label of medication kits
Mis-randomized participants	Mis-randomized participants are those who were not qualified for randomization and who did not take study treatment, but have been inadvertently randomized into the study or the participant allocated to an invalid stratification factor
Off-site	Describes trial activities that are performed at remote location by an off-site healthcare professional, such as procedures performed at the participant's home.
Off-site healthcare Professional (OHP)	A qualified healthcare professional, who performs certain protocol procedures for the participant in an off-site location such as a participant's home.
Orthostatic hypotension	A reduction in SBP of ≥20 mmHg or in DBP of ≥10 mmHg within 3 min of standing
Other treatment	Treatment that may be needed/allowed during the conduct of the study (i.e. concomitant or rescue therapy)
Part	A sub-division of a study used to evaluate specific objectives or contain different populations. For example, one study could contain a single dose part and a multiple dose part, or a part in participants with established disease and in those with newly-diagnosed disease
Participant	A trial participant (can be a healthy volunteer or a patient). "Participant" terminology is used in the protocol whereas term "Subject" is used in data collection
Participant number	A unique number assigned to each participant upon signing the informed consent. This number is the definitive, unique identifier for the participant and should be used to identify the participant throughout the study for all data collected, sample labels, etc.
Patient-Reported Outcome (PRO)	A measurement based on a report that comes directly from the participant about the status of a participant's health condition without amendment or interpretation of the participant's report by a clinician or anyone else
Period	The subdivisions of the trial design (e.g. Screening, Treatment, Follow-up) which are described in the Protocol. Periods define the study phases and will be used in clinical trial database setup and eventually in analysis
Perpetrator drug	A drug which affects the pharmacokinetics of the other drug
Personal data	Participant information collected by the Investigator that is coded and transferred to Novartis/Sponsors for the purpose of the clinical trial. This data includes participant identifier information, study information and biological samples.
Randomization	The process of assigning trial participants to investigational drug or control/comparator drug using an element of chance to determine the assignments in order to reduce bias.
Randomization number	A unique identifier assigned to each randomized participant
Remote	Describes any trial activities performed at a location that is not the investigative site.
Rescreening	If a participant fails the initial screening and is considered as a Screen Failure, he/she can be invited once for a new Screening visit after medical judgment and as specified by the protocol
Screen Failure	A participant who did not meet one or more criteria that were required for participation in the study
Source Data/Document	Source data refers to the initial record, document, or primary location from where data comes. The data source can be a database, a dataset, a spreadsheet or even hard-coded data, such as paper or eSource
Start of the clinical trial	The start of the clinical trial is defined as the signature of the informed consent by the first participant.
Study treatment	Any drug or combination of drugs or intervention administered to the study participants as part of the required study procedures; includes investigational drug(s), control(s) or background therapy

Tele-visit	Procedures or communications conducted using technology such as telephone or video-conference, whereby the participant is not at the investigative site where the Investigator will conduct the trial.
Treatment arm/group	A treatment arm/group defines the dose and regimen or the combination, and may consist of 1 or more cohorts.
Treatment of interest	The treatment of interest and, as appropriate, the alternative treatment to which comparison will be made. These might be individual interventions, combinations of interventions administered concurrently, e.g. as add-on to standard of care, or might consist of an overall regimen involving a complex sequence of interventions. This is the treatment of interest used in describing the related clinical question of interest, which might or might not be the same as the study treatment.
Variable (or endpoint)	The variable (or endpoint) to be obtained for each participant that is required to address the clinical question. The specification of the variable might include whether the participant experiences an intercurrent event.
Withdrawal of consent	Withdrawal of consent from the study occurs when the participant explicitly requests to stop use of their data and/or biological samples AND no longer wishes to receive study treatment, AND does not agree to further protocol required assessments. This request should be in writing (depending on local regulations) and recorded in the source documentation.<br>This request should be distinguished from a request to discontinue the study. Other study participant's privacy rights are described in the corresponding informed consent form.

10.3 Appendix 3: Clinical laboratory tests

10.3.1 Clinically notable laboratory values and vital signs

Clinically notable laboratory abnormalities for selected tests performed by Central Laboratory (based on: 1) proportional or absolute changes from baseline; 2) the limit of the normal range established by the Central Laboratory; 3) cut-off values used to define Grade 3 as established by the Common Terminology Criteria for Adverse Events [CTCAE] Version 5.0 (US Department of Health and Human Services 2017) or higher).

Hematology

Table 10-1 Clinically notable hematology abnormalities

Parameter	Shift	Criteria
Red blood cell count	Increase<br>Decrease	> 50% increase AND value > ULN<br>> 30% decrease AND value < LLN
Hemoglobin	Increase<br>Decrease	> 50% increase AND value > ULN<br>either (> 30% decrease AND value < LLN) OR value < 8.0 g/dL
Hematocrit	Increase<br>Decrease	> 50% increase AND value > ULN<br>> 30% decrease AND value < LLN
White blood cell count	Increase<br>Decrease	value > 100,000 cells/mm^3<br>value < 2000 cells/mm^3
Platelet count	Decrease	value < 50,000 platelets/mm^3
ULN: upper limit of the normal range; LLN: lower limit of the normal range		

Blood chemistry

Table 10-2 Clinically notable blood chemistry abnormalities

Parameter	Shift	Criteria
Alkaline phosphatase	Increase	value > 5 x ULN if baseline was normal OR value > 5 x baseline if baseline was abnormal
ALT (SGPT)	Increase	value > 5 x ULN if baseline was normal OR value > 5 x baseline if baseline was abnormal
AST (SGOT)	Increase	value > 5 x ULN if baseline was normal OR value > 5 x baseline if baseline was abnormal
Total bilirubin	Increase	value > 3 x ULN if baseline was normal OR value > 3 x baseline if baseline was abnormal
BUN	Increase	≥ 50% increase
Creatinine	Increase	≥ 50% increase
Potassium	Increase	value > 6.0 mmol/L
	Decrease	value < 3.0 mmol/L
Chloride	Increase	> 115 mEq/L
	Decrease	< 90 mEq/L

**Parameter**	**Shift**	**Criteria**
Calcium	Increase	corrected serum calcium > 3.1 mmol/L
	Decrease	corrected serum calcium < 1.75 mmol/L
Uric acid	Increase	> 50% increase
Plasma glucose	Increase	> 25% increase
	Decrease	value < 40 mg/dL
ULN: upper limit of the normal range		

Along with investigators, the clinical trial team from Novartis will receive alerts for clinically notable values via fax/e-portal (or appropriate means agreed with central labs and Novartis).

10.4 Appendix 4: Participant Engagement

The following participant engagement initiatives are included in this study and will be provided, as available, for distribution to study participants at the time points indicated. If compliance is impacted by cultural norms or local laws and regulations, sites may discuss modifications to these requirements with Novartis.

- Thank You letter
- Plain language trial summary - after CSR publication

10.5 Appendix 5: Liver safety monitoring

10.5.1 Liver event and laboratory trigger definitions & follow-up requirements

Table 10-3 Liver event and laboratory trigger definitions

	**Definition/threshold**
If ALT or AST normal at baseline	
Liver laboratory triggers	· 3 x ULN < ALT or AST ≤ 5 x ULN<br>· TBL > ULN*
Liver events	· ALT or AST > 5 × ULN<br>· ALP > 2 × ULN (in the absence of known bone pathology)<br>· Total bilirubin > 3 × ULN (in the absence of known Gilbert syndrome)<br>· ALT or AST > 3 × ULN AND (TBL > 2 x ULN OR INR > 1.5)<br>· Potential Hy's Law cases (defined as ALT or AST > 3 × ULN and Total bilirubin > 2 × ULN [mainly conjugated fraction] without notable increase in ALP to > 2 × ULN)<br>· Any clinical event of jaundice (or equivalent term)<br>· ALT or AST > 3 × ULN accompanied by (general) malaise, fatigue, abdominal pain, nausea, or vomiting, or rash with eosinophilia<br>· Any adverse event potentially indicative of a liver toxicity
If ALT or AST elevated at baseline:	
Liver laboratory triggers	· ALT or AST > 2 x baseline or > 200 U/L (whichever occurs first)<br>· TBL > ULN*
Liver events	· ALT or AST > 3 x baseline or > 300 U/L (whichever occurs first)<br>· (ALT or AST > 2 × baseline or > 200 U/L [whichever occurs first]) AND (TBL > 2 x ULN OR INR > 1.5)<br>· ALT or AST > 5 × ULN<br>· ALP > 2 × ULN (in the absence of known bone pathology)<br>· Total bilirubin > 3 × ULN (in the absence of known Gilbert syndrome)<br>· Potential Hy's Law cases (defined as ALT or AST > 3 × ULN and Total bilirubin > 2 × ULN [mainly conjugated fraction] without notable increase in ALP to > 2 × ULN)<br>· Any clinical event of jaundice (or equivalent term)<br>· (ALT or AST > 2 × baseline or > 200 U/L [whichever occurs first]) accompanied by (general) malaise, fatigue, abdominal pain, nausea, or vomiting, or rash with eosinophilia<br>· Any adverse event potentially indicative of a liver toxicity

* Fractionate bilirubin, evaluate for cholestatic liver injury (ALP) or alternative causes of bilirubin elevation. Treat alternative causes according to local institutional guidelines

Table 10-4 Follow up requirements for liver laboratory triggers - ALT, AST, TBL

	ALT	TBL	Liver Symptoms	Action
**ALT increase without bilirubin increase:**				
	**If normal at baseline:**<br>ALT > 3 x ULN<br>**If elevated at baseline:**<br>ALT > 2 x baseline<br>or > 200 U/L (whichever occurs first)	Normal<br>For participants with Gilbert's syndrome: No change in baseline TBL	None	● **No change to study treatment**<br>● Measure ALT, AST, ALP, GGT, TBL, INR, albumin, CK, and GLDH in 48-72 hours.<br>● Follow-up for symptoms.
	**If normal at baseline:**<br>ALT > 5 x ULN for more than two weeks<br>**If elevated at baseline:**<br>ALT > 3 x baseline<br>or > 300 U/L (whichever occurs first) for more than two weeks	Normal<br>For participants with Gilbert's syndrome: No change in baseline TBL	None	● **Interrupt study treatment**<br>● Measure ALT, AST, ALP, GGT, TBL, INR, albumin, CK, and GLDH in 48-72 hours.<br>● Follow-up for symptoms.<br>● Initiate close monitoring and workup for competing etiologies.<br>● Study treatment can be restarted only if another etiology is identified and liver enzymes return to baseline.
	**If normal at baseline:**<br>ALT > 8 x ULN	Normal	None	
**ALT increase with bilirubin increase:**				
	**If normal at baseline:**<br>ALT > 3 x ULN<br>**If elevated at baseline:**<br>ALT > 2 x baseline<br>or > 200 U/L (whichever occurs first)	TBL > 2 x ULN (or INR > 1.5)<br>For participants with Gilbert's syndrome: Doubling of direct bilirubin	None	
	**If normal at baseline:**<br>ALT > 3 x ULN<br>**If elevated at baseline:**<br>ALT > 2 x baseline<br>or > 200 U/L (whichever occurs first)	Normal or elevated	Severe fatigue, nausea, vomiting, right upper quadrant pain	

Table 10-5 Follow-up requirements for liver laboratory triggers based on isolated elevations in TBL

Criterion	Actions required	Follow-up monitoring
**Total Bilirubin (isolated)**		
Any elevation > ULN	Fractionate bilirubin, evaluate for cholestatic liver injury (ALP) or alternative causes of bilirubin elevation. Treat alternative causes according to local institutional guidelines	As shown below according to the degree of elevation
>1.5 – 3.0 ULN	● Maintain treatment<br>● Repeat LFTs within 48-72 hours	Monitor LFTs weekly until resolution to ≤ Grade 1 or to baseline
> 3 - 10 × ULN (in the absence of known Gilbert syndrome)	● Interrupt treatment<br>● Repeat LFT within 48-72 hours<br>● Hospitalize if clinically appropriate<br>● Establish causality<br>● Record the AE and contributing factors (e.g. concomitant medications, medical history, lab test) in the appropriate CRF	Monitor LFTs weekly until resolution to ≤ Grade 1 or to baseline (ALT, AST, total bilirubin, Alb, PT/INR, ALP and GGT)<br>Test for hemolysis (e.g. reticulocytes, haptoglobin, unconjugated [indirect] bilirubin)
> 10 x ULN	● Discontinue the study treatment immediately after all other alternative causes are ruled out<br>● Record the AE and contributing factors(e.g. concomitant medications, medical history, lab test)in the appropriate CRF	Monitor ALT, AST, total bilirubin, Alb, PT/INR, ALP and GGT until resolution (frequency at investigator discretion)
Any AE potentially indicative of a liver toxicity	● Consider study treatment interruption or discontinuation<br>● Hospitalization if clinically appropriate<br>● Establish causality<br>● Record the AE and contributing factors(e.g. concomitant medications, medical history, lab test)in the appropriate CRF	Investigator discretion

10.6 Appendix 6: Renal safety monitoring

10.6.1 Specific Renal Alert Criteria and Actions and Event Follow-up

Table 10-6 Renal event criteria and corresponding actions to be taken upon their occurrence

Renal event	Actions
Confirmed serum creatinine increase of 25 – 49% from baseline	● Consider causes and possible interventions<br>● Follow-up within 2-5 days
Confirmed serum creatinine increase ≥ 50% from baseline (corresponds to KDIGO criterion for acute kidney injury)	● Consider causes and possible interventions<br>● Repeat assessment within 24-48h if possible<br>● Consider drug interruption or discontinuation unless other causes are diagnosed and corrected<br>● Consider participant hospitalization and specialized treatment
New onset dipstick proteinuria ≥ 300 mg/dL<br>OR<br>Protein-creatinine ratio (PCR) ≥ 1g/g Cr (or mg/mmol equivalent as converted by the measuring laboratory)	● Consider causes and possible interventions<br>● Assess serum albumin and serum total protein<br>● Repeat assessment to confirm<br>● Consider drug interruption or discontinuation unless other causes are diagnosed and corrected
New onset of hematuria ≥ 1 mg/dL on urine dipstick	● Repeat assessment to confirm<br>● Distinguish hemoglobinuria from hematuria<br>● Urine sediment microscopy<br>● Assess serum creatinine<br>● Exclude infection, trauma, bleeding from the distal urinary tract or bladder, menstruation<br>● Consider bleeding disorder

FOLLOW-UP OF RENAL EVENTS

Assess, document and record in CRF:

- Urine dipstick and sediment microscopy evidence of drug-induced nephrotoxicity (DIN): crystals, red blood cells (dysmorphic/glomerular vs. non-dysmorphic/non-glomerular), white blood cells, tubular epithelial cells
- Blood pressure and body weight
- Serum creatinine, BUN, electrolytes (sodium, potassium, phosphate, calcium) and uric acid
- Urine output

Review and record possible contributing factors to the renal event (co-medications, other co-morbid conditions) and additional diagnostic procedures (MRI etc.) in the CRF

Monitor participant regularly (frequency at investigator's discretion) until:

- Event resolution: (sCr within 10% of baseline or PCR < 1 g/g Cr, or ACR <300 mg/g Cr) or
- Event stabilization: sCr level with ±10% variability over last 6 months or protein-creatinine ratio stabilization at a new level with ±50% variability over last 6 months.
- Analysis of urine markers in samples collected over the course of the DIN event

10.7 Appendix 7: Managing hypotension

Investigators should monitor BP closely to identify episodes of symptomatic hypotension at each clinic visit. The occurrence of symptomatic hypotension should be reported as adverse events. Participants should be instructed to report to the site if they experience any symptoms relating to hypotension (i.e. giddiness, lightheadedness, syncope, falls, etc.) at their earliest convenience. The investigator should evaluate the events and take appropriate action. Investigator should exercise best clinical judgment based on laboratory assessments, volume status and any other clinical considerations in individual participants.

When deciding on how to modify background medication, it should be taken into account that XXB750, a mAb with a [redacted], is dosed by subcutaneous injection every 4 weeks and, consequently, it is not possible to modify its dose with the intention of producing immediate relief/resolution of symptomatic hypotension or other AEs. Therefore, the recommended way of managing symptomatic hypotension is as follows:

As a general guidance, **persistent and troublesome symptoms attributed to hypotension**, regardless of blood pressure readings, **should be addressed**. In contrast, **asymptomatic low blood pressure is common in patients with HF** receiving conventional therapy and **does not usually require intervention.**

Ensure the participant is not dehydrated and if appropriate, consider **adjusting diuretic dose.**

Consider reducing or stopping non-HF medications that may be contributing to hypotension (e.g., calcium channel blockers, alpha-blockers, nitrates, etc.).

If symptoms persist despite the above steps, consider reducing the dose of HF treatments (e.g., ACEI/ARB, sacubitril/valsartan, MRA, SGLT2 inhibitor and beta-blocker) or sacubitril/valsartan if it is the randomized study medication (i.e., participant randomized to arm 5).

Heart failure medication should be up-titrated or reintroduced at the earliest time point possible per investigator's best clinical judgment.

Figure 10-1 Flow chart for managing hypotension

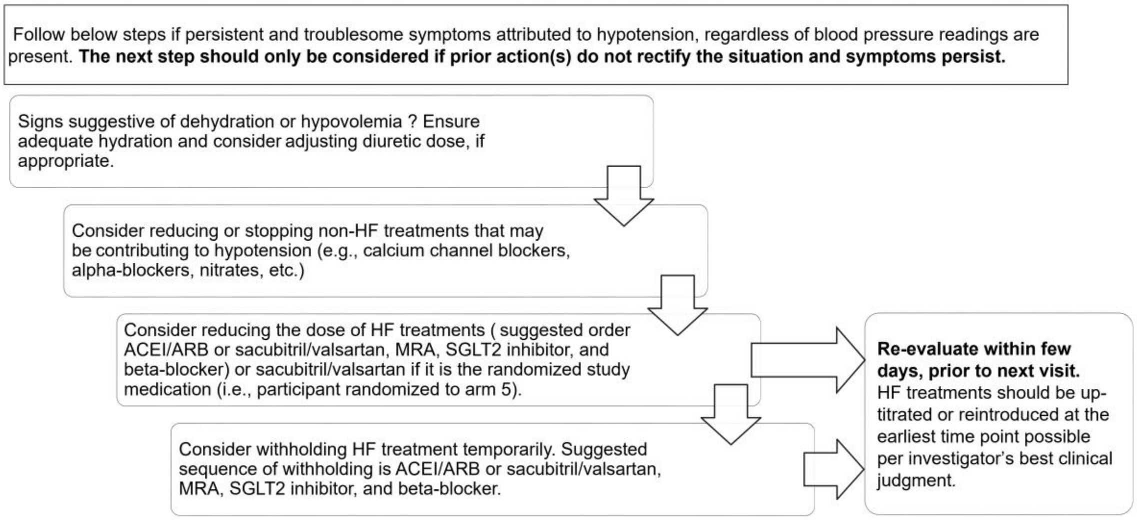

10.8 Appendix 8: Managing hyperkalemia

General principles

Elevation of potassium levels above the cut-off values defined below should be repeated and confirmed before any action is taken.

Any participant who presents with a potassium level > 5.4 mmol/L at screening, confirmed by repeat testing, should be considered a screen failure. Any participant with a serum potassium > 5.3 mmol/L after randomization requires regular, repeated checks of potassium concentration (beyond that required in the protocol) until it is clear that the potassium concentration is stable and not rising into the range of concern (≥ 5.5 and < 6.0 mmol/L) or of potential danger (≥ 6.0 mmol/L). Participants with an elevated potassium value should be managed according to the corrective actions outlined below. Hyperkalemia should be followed until resolution.

Corrective action for management of hyperkalemia

Serum potassium > 5.3 mmol/L and ≤ 5.5 mmol/L

- Confirm potassium concentration in a non-hemolyzed sample
- Reinforce low potassium diet and restriction of food/drinks with high potassium content (e.g. orange juice, melon, bananas, low-salt substitutes, etc.)
- Review medical regimen (including dietary supplements and over-the-counter medications) for agents known to cause hyperkalemia
- Consider reduction in dose or discontinuation of the following non-disease modifying agents:
  - Potassium-sparing diuretics (e.g., amiloride and triamterene) including when in combination products with thiazide or loop diuretics
  - Potassium supplements, e.g., potassium chloride
  - Salt substitutes
  - Trimethoprim and trimethoprim-containing combination products, such as Bactrim® and Septra® (trimethoprim/sulfamethoxazole fixed combination)
  - Non-steroidal anti-inflammatory drugs (NSAIDs)
  - Cyclo-oxygenase-2 (COX-2) inhibitors
  - Herbal Supplements: For example, Noni juice, alfalfa (*Medicagosativa*), dandelion (*Taraxacum officinale*), horsetail (*Equisetum arvense*), nettle (*Urtica dioica*), milkweed, lily of the valley, Siberian ginseng, hawthorn berries
- Consider down-titration of background HF agents:
  - Aldosterone antagonists (if they are believed to be the most likely cause of hyperkalemia)
  - Sacubitril/valsartan or ACEI/ARB, according to the investigator’s medical judgment
- Repeat serum potassium measurement within 3 to 5 days
- If serum potassium remains > 5.3 and ≤ 5.5 mmol/L, regularly monitor serum potassium levels to ensure stability (suggested once monthly)

Serum potassium > 5.5 and < 6.0 mmol/L

- Confirm potassium concentration in a non-hemolyzed sample
- Apply all measures outlined for serum potassium > 5.3 and ≤ 5.5 mmol/L
- Consider down-titration or discontinuation of background HF agents according to the investigator's medical judgment:
  - Aldosterone antagonists (if they are believed to be the most likely cause of hyperkalemia)
  - Sacubitril/valsartan or ACEI/ARB
- Repeat serum potassium measurement after 2-3 days
- If serum potassium < 5.5 mmol/L, consider resumption of background HF agent at a lower dose and repeat potassium measurement within 5 days

Serum potassium ≥ 6.0 mmol/L

- Immediately hold further administration of all non-disease modifying and disease-modifying medications known to cause hyperkalemia as listed above.
- Confirm potassium concentration in a non-hemolyzed sample
- Urgently evaluate patient and treat hyperkalemia as clinically indicated
- Apply all measures outlined for serum potassium > 5.3 and < 6.0 mmol/L

Discontinued background HF agents or study drug must not be resumed without individualized case discussion with, and permission from, Novartis medical monitor or his/her designee. In general, discontinued non-disease modifying agents known to cause hyperkalemia should not be resumed during the duration of the study.

11 References

References are available upon request.

Chen HH, Wan SH, Iyer SR, et al (2021) First-in-Human Study of MANP: A Novel ANP (Atrial Natriuretic Peptide) Analog in Human Hypertension. Hypertension; 78(6):1859-1867.

Crespo-Leiro MG, Anker SD, Maggioni AP, et al (2016) European Society of Cardiology Heart Failure Long-Term Registry (ESC-HF-LT): 1-year follow-up outcomes and differences across regions. Eur J Heart Fail; 18(6):613-25.

Desai AS, McMurray JJ, Packer M, et al (2015) Effect of the angiotensin-receptor-neprilysin inhibitor LCZ696 compared with enalapril on mode of death in heart failure patients. Eur Heart J; 36(30):1990-7.

Dong G, Qiu J, Wang D, et al (2018) The stratified win ratio. J Biopharm Stat; 28(4):778-96.

FDA (2021) Sponsor Responsibilities - Safety Reporting Requirements and Safety Assessment for IND and Bioavailability/Bioequivalence Studies. Guidance for Industry (draft guidance). US Department of Health and Human Services. FDA, CDER, CBER, Jun-2021. Drug Safety.

Fiuzat M, Hamo CE, Butler J, et al (2022) Optimal Background Pharmacological Therapy for Heart Failure Patients in Clinical Trials: JACC Review Topic of the Week. J Am Coll Cardiol; 79(5):504-10.

Gardner DG, Chen S, Glenn DJ, et al (2007) Molecular biology of the natriuretic peptide system: implications for physiology and hypertension. Hypertension; 49(3):419-26.

Green CP, Porter CB, Bresnahan DR, et al (2000) Development and evaluation of the Kansas City Cardiomyopathy Questionnaire: a new health status measure for heart failure. J Am Coll Cardiol; 35(5):1245-55.

Groenewegen A, Rutten FH, Mosterd A, et al (2020) Epidemiology of heart failure. Eur J Heart Fail; 22(8):1342-56.

Heidenreich PA, Bozkurt B, Aguilar D, et al (2022) 2022 AHA/ACC/HFSA Guideline for the Management of Heart Failure: A Report of the American College of Cardiology/American Heart Association Joint Committee on Clinical Practice Guidelines. J Am Coll Cardiol; 79(17):e263-e421.

Inker LA, Eneanya ND, Coresh J, et al (2021) New Creatinine- and Cystatin C-Based Equations to Estimate GFR without Race. N Engl J Med; 385(19):1737-49.

Januzzi JL, Zannad F, Anker SD, et al (2021) Prognostic Importance of NT-proBNP and Effect of Empagliflozin in the EMPEROR-Reduced Trial. J Am Coll Cardiol; 78(13):1321-32.

Lawson CA, Zaccardi F, Squire I, et al (2019) 20-year trends in cause-specific heart failure outcomes by sex, socioeconomic status, and place of diagnosis: a population-based study. Lancet Public Health; 4(8):e406-e420.

Levin ER, Gardner DG, Samson WK (1998) Natriuretic peptides. N Engl J Med; 339(5):321-8.

McDonagh TA, Metra M, Adamo M, et al (2021) 2021 ESC Guidelines for the diagnosis and treatment of acute and chronic heart failure. Eur Heart J; 42(36):3599-726.

McMurray JJ, Packer M, Desai AS, et al (2014) Angiotensin-neprilysin inhibition versus enalapril in heart failure. N Engl J Med; 371(11):993-1004.

Pandey KN (2008) Emerging Roles of Natriuretic Peptides and their Receptors in Pathophysiology of Hypertension and Cardiovascular Regulation. J Am Soc Hypertens; 2(4):210-26.

Pinheiro J, Bornkamp B, Bretz F (2006) Design and analysis of dose-finding studies combining multiple comparisons and modeling procedures. J Biopharm Stat; 16(5):639-56.

Pinheiro J, Bornkamp B, Glimm E, et al (2014) Model-based dose finding under model uncertainty using general parametric models. Stat Med; 33(10):1646-61.

Pocock SJ, Ariti CA, Collier TJ, et al (2012) The win ratio: a new approach to the analysis of composite endpoints in clinical trials based on clinical priorities. Eur Heart J; 33(2):176-82.

Roger VL (2021) Epidemiology of Heart Failure: A Contemporary Perspective. Circ Res; 128(10):1421-34.

Savarese G, Becher PM, Lund LH, et al (2022) Global burden of heart failure: A comprehensive and updated review of epidemiology. Cardiovasc Res; 118(17):3272-87.

Schmitt W, Rühs H, Burghaus R, et al (2021) NT-proBNP Qualifies as a Surrogate for Clinical End Points in Heart Failure. Clin Pharmacol Ther; 110(2):498-507.

Solomon SD, Vaduganathan M, L Claggett B, et al (2020) Sacubitril/Valsartan Across the Spectrum of Ejection Fraction in Heart Failure. Circulation; 141(5):352-61.

Tromp J, Ouwerkerk W, van Veldhuisen DJ, et al (2022) A Systematic Review and Network Meta-Analysis of Pharmacological Treatment of Heart Failure With Reduced Ejection Fraction. JACC Heart Fail; 10(2):73-84.

US Department of Health and Human Services, National Institutes of Health, National Cancer Institute. (2017) Common Terminology Criteria for Adverse Events, Version 5.0.

Vaduganathan M, Claggett B, Packer M, et al (2018) Natriuretic Peptides as Biomarkers of Treatment Response in Clinical Trials of Heart Failure. JACC Heart Fail; 6(7):564-69.

Vaduganathan M, Claggett BL, Jhund PS, et al (2020) Estimating lifetime benefits of comprehensive disease-modifying pharmacological therapies in patients with heart failure with reduced ejection fraction: a comparative analysis of three randomised controlled trials. Lancet; 396(10244):121-28.

Zile MR, Claggett BL, Prescott MF, et al (2016) Prognostic Implications of Changes in N-Terminal Pro-B-Type Natriuretic Peptide in Patients With Heart Failure. J Am Coll Cardiol; 68(22):2425-36.

NOVARTIS

Clinical Development

XXB750

CXXB750A12201 / NCT06142383

A multi-center, randomized, placebo- and active-controlled, parallel-group, 24-week proof of concept and dose-finding study to evaluate efficacy, safety, and tolerability of XXB750 in patients with heart failure

Statistical Analysis Plan (SAP)

Document type:	SAP Documentation
Document status:	Final
Release date:	30-Nov-2023
Number of pages:	46

Property of Novartis
Confidential
May not be used, divulged, published or otherwise disclosed
without the consent of Novartis

Template Version 6.0, Effective from 23-Nov-2022

Document History – Changes compared to previous final version of SAP

Date	Time point	Reason for update	Outcome for update	Section and title impacted (Current)
30-Nov-2023	Prior to FPFV	Creation of final version	N/A - First version	NA

Table of contents

Table of contents 3
List of tables 5
List of figures 5
List of abbreviations 6
1 Introduction 7
1.1 Study design 7
1.2 Study objectives, endpoints and estimands 8
1.2.1 Primary estimand(s) 9
1.2.2 Secondary estimand(s) 10
2 Statistical methods 11
2.1 Data analysis general information 11
2.1.1 General definitions 12
2.2 Analysis sets 14
2.2.1 Subgroup of interest 14
2.3 Patient disposition, demographics and other baseline characteristics 15
2.3.1 Patient disposition 15
2.3.2 Demographics and other baseline characteristics 16
2.3.3 Medical history 16
2.4 Treatments (study treatment, rescue medication, concomitant therapies, compliance) 17
2.4.1 Study treatment / compliance 17
2.4.2 Prior, concomitant and post therapies 19
2.5 Analysis supporting primary objective(s) 20
2.5.1 Primary endpoint(s) 20
2.5.2 Statistical hypothesis, model, and method of analysis 20
2.5.3 Handling of intercurrent events 23
2.5.4 Handling of missing values not related to intercurrent event 24
2.5.5 Multiplicity adjustment 24
2.5.6 Sensitivity analyses 24
2.5.7 Supplementary analyses 24
2.6 Analysis supporting secondary objectives 25
2.6.1 Secondary endpoint(s) 25
2.6.2 Statistical hypothesis, model, and method of analysis 26
2.6.3 Handling of intercurrent events 27
2.6.4 Handling of missing values not related to intercurrent event 27

2.6.5 Sensitivity analyses 27
2.6.6 Supplementary analyses 27
2.7 Safety analyses....27
2.7.1 Adverse events (AEs)....28
2.7.2 Adverse events of special interest and potential risks....30
2.7.3 Deaths....32
2.7.4 Laboratory data 32
2.7.5 Other safety data 35
2.8 Pharmacokinetic endpoints....36
2.9 PD and PK/PD analyses 37
2.10 Patient-reported outcomes 37
2.11 Biomarkers....37
2.12 Other Exploratory analyses....37
2.12.1 Exploratory variables 37
2.12.2 Analysis methods 38
2.13 Interim analysis....40
3 Sample size calculation 41
3.1 Primary endpoint (s) 41
3.2 Secondary endpoint(s) 41
4 Change to protocol specified analyses 42
5 Appendix 42
5.1 Imputation rules....42
5.1.1 Study drug....42
5.1.2 AE date imputation 42
5.1.3 Concomitant medication date imputation 42
5.2 AEs coding/grading 42
5.3 Laboratory parameters derivations 42
5.4 Statistical models....42
5.4.1 Analysis supporting primary objective(s)....42
5.4.2 Analysis supporting secondary objective(s) 42
5.5 Rule of exclusion criteria of analysis sets 43
5.6 KCCQ derivation of scores in each domain 43
6 Reference....46

List of tables

Table 1-1 Objectives and related endpoints 8
Table 2-1 Specification of subgroups 15
Table 2-2 Study treatment dose levels during randomized treatment epoch 17
Table 2-3 Allocation of AEs (randomized treatment period and safety follow-up period listed separately from randomization period) 28
Table 2-4 AESIs and potential risks with additional analysis 31
Table 2-5 Clinically notable laboratory values and vital signs 33
Table 2-6 Selecting a winner based on combination of change of KCCQ CSS and change of NYHA class 39
Table 5-1 Criteria leading to exclusion 43

List of figures

Figure 1-1 Study design 8
Figure 2-1 Dose-response curve of candidate models 21
Figure 2-2 Sequentially rejective multiple testing procedure 27

List of abbreviations

AE	Adverse Event
CRF	Case Report Form
CSR	Clinical Study Report
DMC	Data Monitoring Committee
DMS	Document Management System
FAS	Full Analysis Set
IA	Interim Analyses
MedDRA	Medical Dictionary for Drug Regulatory Affairs
PK	Pharmacokinetics
PPS	Per-Protocol Set
PRO	Patient-reported Outcomes
RAP	Reporting & Analysis Process
SAP	Statistical Analysis Plan
SAS	Statistical Analysis System
TFLs	Tables, Figures, Listings
WHO	World Health Organization

1 Introduction

The statistical analysis plan (SAP) describes the detailed methodology and implementation of the planned statistical analyses outlined in the study protocol for CXXB750A12201. The analyses following the SAP below will be used for clinical study reporting purposes.

This version of SAP details the statistical methodology for the analyses planned and agreed to at the time of finalization of the CXXB750A12201 (Version 00 – original protocol).

1.1 Study design

Study CXXB750A12201 is a multicenter, randomized, placebo- and active-controlled, parallel group phase 2 study which is comprised of three periods (Figure 1-1):

- A screening period of approximately 7 days;
- A 16-week parallel-group randomized treatment period on either
  - i) double-blind placebo-controlled treatment (XXB750 vs. placebo) or,
  - ii) open-label treatment (sacubitril/valsartan);
- An 8-week safety follow-up period.

The study will randomize adult participants with LVEF < 50% receiving ACEI/ARB/ARNI and guideline-recommended HF therapies for HFrEF or HFmrEF to three XXB750 target dose levels; a cohort of patients treated with ACEI/ARB before the study will be randomized to be converted to open-label sacubitril/valsartan in place of their pre-study ACEI/ARB. The study will randomize a total of approximately 720 patients. The percentage of patients with LVEF > 40% will be limited to approximately 25% of the total sample.

Refer to study protocol Section 6.1.2 for details on the administered doses in the different treatment arms.

A staggered approach to enrollment will be followed in the study protocol. The first group of 300 participants will be randomized to placebo (treatment arm 1), XXB750 60 mg target dose (treatment arm 2), XXB750 120 mg target dose (treatment arm 3), and conversion to sacubitril/valsartan (treatment arm 5). The independent Data Monitoring Committee (DMC) will complete a safety analysis of the XXB750 doses tested and assess if it is safe to allow the dosing of patients with XXB750 planned top dose of 240 mg (i.e., allowing enrollment in arms 4a and 4b as planned). See study protocol Section 4.6 for details on planned interim analyses and adaptive design features.

Figure 1-1 Study design

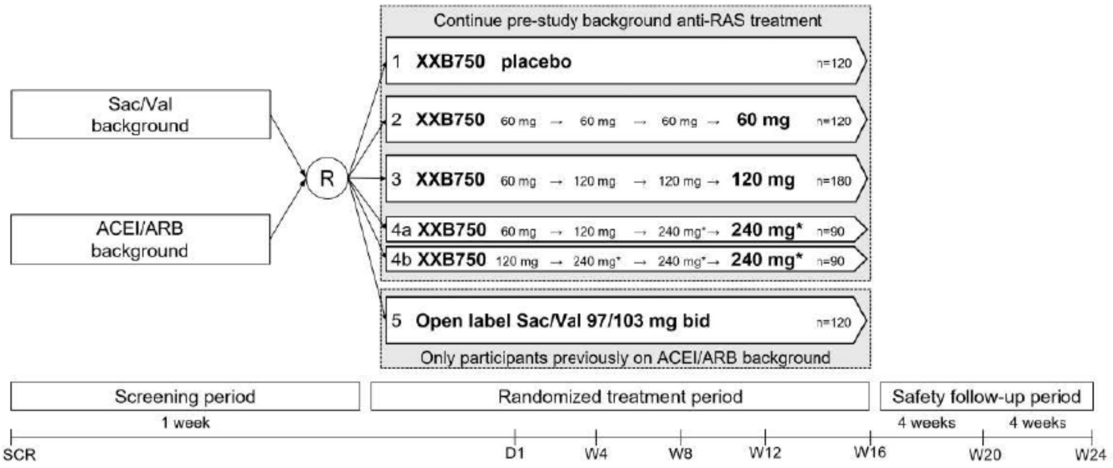

Sac/Val: Open label sacubitril/valsartan. *May be modified per adaptive design feature of the study (study protocol Section 4.6). Doses of Sac/Val, ACEI or ARB in arms 1-4 equal to dose at randomization.

The primary analysis will be performed after all participants have completed Week 16 (or discontinued prior to Week 16). A final analysis will be performed after all participants completed Week 24 (or discontinued prior to Week 24). Formal testing of the primary endpoint with full level alpha of 0.025 (1-sided) will be performed at the primary analysis time point.

1.2 Study objectives, endpoints and estimands

Table 1-1 Objectives and related endpoints

Objective(s)	Endpoint(s)
**Primary objective(s)**	**Endpoint(s) for primary objective(s)**
• To evaluate the efficacy and dose-response relationship of three XXB750 target dose levels compared to placebo in reducing NT-proBNP from baseline to Week 16 in symptomatic HF patients with LVEF < 50% treated with standard of care, including ACEI/ARB or sacubitril/valsartan.	• Change in log NT-proBNP from baseline to Week 16
**Secondary objective(s)**	**Endpoint(s) for secondary objective(s)**
• To evaluate the treatment effect of the highest XXB750 target dose level compared to placebo in reducing NT-proBNP from baseline to Week 16 in symptomatic HF patients with LVEF < 50% treated with standard of care, including ACEI/ARB or sacubitril/valsartan.	• Change in log NT-proBNP from baseline to Week 16
• To evaluate the treatment effect of combined two highest XXB750 target dose levels administered in addition to a background of ACEI/ARB versus conversion from ACEI/ARB to sacubitril/valsartan, in reducing NT-proBNP from baseline to Week 16.	• Change in log NT-proBNP from baseline to Week 16

Objective(s)	Endpoint(s)
• To evaluate the safety and tolerability of XXB750 up-titration regimens and dose levels.	• Adverse events (incidence and severity, including but not limited to, hypotension, tachycardia, bradycardia, hypersensitivity and injection site reactions), vital signs (blood pressure, pulse), safety laboratory tests, and ECG parameters from baseline to end of study (EOS).
**Exploratory objective(s)**	**Endpoint(s) for exploratory objective(s)**
• To evaluate the effects of highest XXB750 target dose level compared to placebo in improving a composite hierarchical outcome of : 1) time to all-cause death, 2) number of HF hospitalizations, 3) Kansas City Cardiomyopathy Questionnaire (KCCQ) clinical summary score (CSS) categorical change, 4) NYHA class change , 5) relative change in NT-proBNP levels between baseline and Week 16.	• Hierarchically ordered composite consisting of: 1) time to all-cause death up to Week 16; 2) number of HF hospitalizations up to Week 16; 3) categorical KCCQ CSS change from baseline to Week 16; 4) NYHA class change from baseline to Week 16; 5) relative change in NT-proBNP levels from baseline to Week 16.
• To evaluate changes in each domain of the KCCQ, including clinical summary score (CSS) and overall summary score (OSS), from baseline to Week 16.	• Change in all domains of the KCCQ from baseline to Week 16.
• To evaluate the effects of XXB750 on slowing the rate of decline in estimated glomerular filtration rate (eGFR) from baseline to Week 16.	• Estimated glomerular filtration rate (eGFR) at scheduled assessment visits.
• To evaluate the change in biomarkers related to heart failure, cardiovascular disease and renal function from baseline to Week 8 and to Week 16.	• Change from baseline in biomarkers including, but not limited to, plasma and urine cGMP, urinary cGMP-to-creatinine ratio, urinary albumin-to-creatinine ratio, BNP and hs-Troponin, at Week 8 and Week 16.
• To evaluate the change in NYHA class and in signs and symptoms of heart failure from baseline to Week 16.	• Change in NYHA class and signs and symptoms of HF from baseline to Week 16.
• To explore the population pharmacokinetic (PK) properties of XXB750 in HF patients.	• Population PK analysis results including mathematic models and parameters.
• To evaluate immunogenicity (IG) of XXB750.	• Detection of immune response markers, including anti-drug antibodies.

1.2.1 Primary estimand(s)

The primary clinical question of interest is:

Is there a dose-response signal, and if so, what is the dose-response relationship among three XXB750 target dose levels and placebo in reducing NT-proBNP from baseline to Week 16 in participants with HF (NYHA classes II-III) and LVEF < 50%, treated with standard of care, including ACEI/ARB or sacubitril/valsartan, regardless of type of baseline background HF

therapy, discontinuation from the study treatment, change in the dose of concomitant medications and receiving prohibited concomitant medication?

The justification for the primary estimand is that it will capture both the effects of XXB750 and the effects of changes in other HF medications. This will provide a clear assessment of the XXB750 treatment effect in clinical practice versus placebo.

The primary estimand is described by the following attributes:

- Population: participants with HFrEF and HFmrEF (LVEF < 50%), NYHA classes II-III and elevated NT-proBNP levels at screening while taking optimal guideline-directed HF therapy.
- Variable: change in log NT-proBNP from baseline to 16 weeks
- Treatment of interest: Treatment groups with three XXB750 target dose levels, and placebo all administered on top of ACEI/ARB or sacubitril/valsartan and other guideline recommended standard of care therapy for HF.

Handling of intercurrent events:

- Discontinuation of study treatment: ignore (treatment policy strategy)
- Change in concomitant medications: ignore (treatment policy strategy)
- Receiving prohibited concomitant medication: ignore (treatment policy strategy)
- The summary measure: difference in variable means between treatments. Since the variable in attribute 2 is equivalent to the log of ratio of Week 16 NT-proBNP over baseline NT-proBNP, and the difference in variable means between 2 treatment groups is the log of ratio of ratio to baseline of the 2 groups, therefore, the summary measure can be back-transformed by exponentiation and expressed as ratio of geometric mean of ratio to baseline of the 2 treatment groups.

1.2.2 Secondary estimand(s)

Treatment effect of the highest XXB750 target dose level compared to placebo in reducing NT-proBNP from baseline at Week 16 in HF patients treated with standard of care, including ACEI/ARB or sacubitril/valsartan

The first secondary clinical question of interest is:

What is the effect of the highest XXB750 dose level versus placebo in reducing NT-proBNP level from baseline to Week 16 in participants with heart failure (NYHA classes II-III) and LVEF < 50%, treated with standard of care, including ACEI/ARB or sacubitril/valsartan, regardless of type of baseline background HF therapy, discontinuation from study treatment, change in the dose of concomitant medications, and receiving prohibited concomitant medication?

The justification for the first secondary estimand is that it will capture both the effects of study medication and the effects of changes in guideline-directed concomitant medications. This will provide a clear assessment of the XXB750 treatment effect in clinical practice versus placebo.

The attributes and handling of intercurrent events are the same as those described in primary estimand except that the treatment of interest being the highest XXB750 dose level and placebo, instead of all XXB750 dose levels as described below:

Highest XXB750 dose level versus placebo in combination with guideline-recommended standard of care therapy for HF.

Treatment effect of combined two highest XXB750 target dose levels in addition to a background of ACEI/ARB versus conversion from ACEI/ARB to sacubitril/valsartan, in reducing NT-proBNP from baseline to Week 16

The clinical question of interest for this objective is:

What is the effect of combined two highest XXB750 dose groups in addition to a background of ACEI/ARB versus conversion from ACEI/ARB to sacubitril/valsartan in reducing NTproBNP level from baseline to Week 16 in participants with heart failure (NYHA classes II-III) and LVEF < 50% regardless of type of baseline background HF therapy, discontinuation from the study treatment, change in the dose of concomitant medications and receiving prohibited concomitant medication?

The attributes and handling of intercurrent events are the same as those described in primary estimand except that:

- Population: participants with HFrEF and HFmrEF (LVEF < 50%), NYHA classes II-III and elevated NT-proBNP levels and on ACEI/ARB at screening while taking other guideline-directed HF therapy.
- Treatment of interest: two highest XXB750 dose levels in addition to ACEI/ARB background medication versus conversion from ACEI/ARB to sacubitril/valsartan in combination with guideline-recommended standard of care therapy for HF.

Evaluation of the safety and tolerability of XXB750 up-titration regimens and target dose levels

Adverse events (AEs) and serious AEs, laboratory parameters and vital signs will be evaluated and summarized in each treatment group for different treatment periods (randomized treatment period and safety follow-up period). Please refer to Section 2.7 for analysis details.

2 Statistical methods

The following sections contain important information on detailed statistical methodology used for clinical study report (CSR) analysis and reporting purposes.

2.1 Data analysis general information

Data will be analyzed by Novartis according to the statistical analysis Section 9 of the study protocol using R 4.1.0 and SAS 9.4, unless otherwise specified. Further details on planned statistical analyses and data-driven regression diagnostics will be presented in the following section and in CSR Appendix 16.1.9. Separate analysis plan developed by the external DMC independent statistician will be used for planned interim analyses.

In general, the continuous variables will be summarized descriptively by presenting n, mean, SD, median, quartiles, minimum and maximum while categorical variables will be summarized by presenting count and percentage of participants in each category. Graphical presentation of summary data will also be provided as applicable.

The randomization in this study will be stratified by region and background medication (ACEI/ARB or sacubitril/valsartan). The stratification factors will be appropriately accounted for in the planned statistical analyses.

Interim safety analysis and regular safety monitoring (approximately every six months unless otherwise requested by DMC) will be conducted and reviewed by an external DMC as specified in the DMC charter. The relevant DMC analyses will be described in a separate DMC analysis plan and will be executed by an external independent statistician and independent programmers privileged to assess unblinded clinical trial data.

2.1.1 General definitions

Study treatment or drug

In future sections through this document, 'study treatment' or 'study drug' will be used to refer to investigational treatment assigned to a participant. Specifically, for the randomized treatment period, study treatment refers to XXB750 doses, XXB750 placebo, and sacubitril/vasartan as assigned to a participant at randomization.

Date of first or last administration of study drug/treatment

Date of first administration of study drug/treatment refers to the date when the first dose of assigned treatment is administered in randomized treatment period. Date of last administration of study drug/treatment refers to the date when the last dose of assigned treatment is administered in randomized treatment period. For participants who are randomized to XXB750 dose arms or placebo arm, on-treatment duration is defined as the last administration of double-blind study drug + 28 days, date of withdrawal of informed consent, or the date of death, whichever occurres first. For participants who are randomized to open-label sacubitril/valsartan arm, on-treatment duration is defined as the last administration of study drug, date of withdrawal of informed consent, or the date of death, whichever occurrs first.

Screening period

Screening period begins at Visit 10 until the day before the start of randomized treatment period (Visit 101) or screening disposition date for those who do not qualify to continue.

Randomized treatment period

The randomized treatment period begins at the time of randomization and ends with the end of treatment (EOT) disposition. During the randomized treatment period, participants will return for scheduled clinic visits. For all safety related analyses randomized treatment starts with the first administration of randomized study drug.

Safety follow-up period

The safety follow-up period begins one day after EOT visit date and ends on the date of EOS.

Randomization period

The randomization period is the combination of the randomized treatment period and the safety follow-up period.

Study exposure

The study exposure means the exposure during the randomization period. The duration of study exposure (days) is defined as the date of end of study minus the date of randomization plus one.

Baseline for randomized treatment period

Only assessments performed prior to first dose of randomized treatment period are considered for baseline. Unless specified otherwise, baseline for the randomized treatment period is defined as the measurement obtained at the randomization visit (Visit 101), or the measurement obtained at an earlier visit (scheduled or unscheduled) which was closest to the Visit 101, if Visit 101 measurement is missing.

Baseline for randomization period

Randomization period is the total duration of randomized treatment period and the safety follow-up period, therefore, the definition of baseline for randomization period will be the same as the baseline for randomized treatment period.

Baseline for safety follow-up period

Unless otherwise specified, baseline for safety follow-up period is defined as the measurement obtained at the EOT visit (Visit 9998) or the measurement obtained closest to the EOT visit if the measurement at EOT is missing.

Unscheduled visit

Only for the analysis of safety evaluation will unscheduled measurements be taken into account. For efficacy evaluations, measurements from unscheduled visits will generally not be used, unless otherwise specified.

On-treatment data for an efficacy endpoint

In all the analyses planned in this document, on-treatment data refer to data collected while participants are on-study-medication (regardless of treatment interruption) or within 28 days after final study drug administration date for participants randomized to XXB750 and placebo arms; and final study drug amininstration date for particpants randomization to open-label sacubitril/valsartan.

Study day

Study day of any assessment is defined as the date of assessment (event/visit) minus the date of randomization plus one unless specified otherwise.

2.2 Analysis sets

The following analysis sets will be defined for statistical analysis:

Screened set (SCR) - All participants who signed the informed consent form. The SCR includes only unique screened participants, i.e., in the re-screened participants only the chronologically last screening data is counted.

Randomized Analysis Set (RAS) - All randomized participants who received a randomization number, regardless of receiving trial medication.

Full Analysis Set (FAS) - All participants to whom study treatment has been assigned by randomization and who are not mis-randomized. Mis-randomized participants are those who have not been qualified for randomization, have been inadvertently randomized into the study and did not receive any study medication. According to the intent to treat principle, participants will be analyzed according to the treatment they have been assigned to during the randomization procedure.

Safety Set (SAF) - All randomized participants who received at least one dose of study treatment. Participants will be analyzed according to the study treatment received, where treatment received is defined as the randomized/assigned treatment if the participant took at least one dose/injection of that treatment or the first treatment received if the randomized/assigned treatment was never received.

PK analysis set (PKS) - All randomized participants with at least one available valid (i.e., not flagged for exclusion) PK concentration measurement, who received any study XXB750 treatment.

Immunogenicity prevalence set (IPS) includes all participants in the Safety set with a non-missing baseline ADA sample **or** at least one non-missing post-baseline ADA sample.

Immunogenicity incidence set (IIS) includes all participants in the Safety set with a non-missing baseline ADA sample **and** at least one non-missing post-baseline ADA sample.

Rules leading to exclusion from specific analysis sets of participants are provided in Appendix 5.5.

2.2.1 Subgroup of interest

Subgroups will be formed to explore the consistency of treatment effects and safety profiling on selected endpoints between the subgroups and the overall population.

In general, subgroups will be defined based on baseline information mentioned in Section 2.1.1.

Subgroups defined for selected endpoints in this study and the ways to derive them are listed in Table 2-1. The details about the subgroup analyses will be described in the corresponding sections as appropriate.

Table 2-1 Specification of subgroups

Subgroup	Method of derivation	Background & Demographics / Exposure	Efficacy	Safety
Age groups: (<65 vs. ≥65 years)	Screening (derived)	X	X	X
Sex (female, male)	Screening	X	X	X
Region*	Derived (pooled countries or country), using screening data	X	X	X
LVEF (≤40% vs. >40%)	Screening		X	X
Type of RAS inhibition (ACEI/ARB, sacubitril/valsartan)	Screening		X	X
Dose level of ACEI/ARB or sacubitril/valsartan	Randomization		X	X
Race (Asian or others, Black or African American, White)	Screening	X	X	X
Weight (≤ median, > median)	Screening		X	X
ADA subgroup** (Positive, Negative, PBL, Missing)	Derived			X

* ***America:*** *Brazil, United States*

Asia: *China, India, Japan, Taiwan*

Central Europe: *Bulgaria, Czechia, Hungary, Slovakia*

Western Europe and other: *Denmark, France, Germany, Italy, Netherlands, Portugal, South Africa, Spain, United Kingdom*

** Defined as positive if ADA screening test at randomization baseline (pre-dose) is negative and the test turns positive at any time post-dose during the study, negative if ADA screening test shows negative at all times during the study, PBL if positive at baseline (pre-dose), and missing otherwise.

2.3 Patient disposition, demographics and other baseline characteristics

2.3.1 Patient disposition

The number and percentage of participants screened successfully will be presented. In addition, screen failure participants will be summarized by primary reason for screen failure. For participants who are screened more than once, the information from the last screen will be used in the summary. The analysis is based on the screened set (SCR).

The number and percentage of randomized participants included in different analysis sets (Section 2.2) will be summarized by treatment group. The number and percentage as well as the reasons that participants had been excluded from RAS will be summarized by treatment.

The number and percentage of randomized participants (RAS) and those in the full analysis set (FAS) who completed the study, who discontinued the study, and the reasons for discontinuation will be presented by treatment group, respectively.

In addition, the number and percentage of participants with protocol deviations (e.g. COVID related) as well as the criteria leading to exclusion from analysis sets will be provided for the participants in randomized set (RAS). Furthermore, the number and percentage of participants randomized per region and per country will be presented for the SCR and the FAS, respectively. All disposition data will also be listed at participant level for randomization period disposition.

2.3.2 Demographics and other baseline characteristics

Summary statistics will be provided for the total number of participants pertaining to the FAS for background and demographic characteristics, disease characteristics, and cardiovascular risk factors for the randomized treatment baseline. The following parameters will be included if applicable:

- **Continuous variables**: Age (in years), weight (in kilogram), height (in centimeter), body mass index (BMI, in kg/m^2), LVEF (%), sitting office systolic blood pressure (in mmHg), sitting office diastolic blood pressure (in mmHg), sitting pulse (in bpm), cyclic GMP (plasma and urine), urine creatinine, cyclic GMP (urine)/urine creatinine, renin concentration, aldosterone, renin/aldosterone ratio, NT-proBNP, dose of standard background therapy of ACEI/ARB or sacubitril/valsartan, Kansas City Cardiomyopathy Questionnaire (KCCQ) clinical summary score (CSS) and overall summary score (OSS), and eGFR ($ml/min/1.73m^2$).
- **Categorical variables**: Age group (<65 years vs. ≥65 years), sex, race, region, history of diabetes (Yes, No), history of MI (Yes, No), history of hypertension (Yes, No), history of atrial fibrillation/flutter (Yes, No), smoking history (Yes, No), alcohol use history (0, >0 to 3, >3 drinks consumed per day in average), prior history of coronary heart disease (Yes, No), LVEF group (≤40% vs. >40%), NYHA class, etiology of HF (ischemic vs. non-ischemic), category of prior CHF medication, eGFR (< 60 vs. ≥ 60 $ml/min/1.73m^2$).

BMI will be calculated as weight (kg) / $height^2$ (m^2) from the measured height and weight at Visit 10 (Screening Visit).

The summary will be presented for FAS.

2.3.3 Medical history

Any condition entered on the relevant medical history / current medical conditions CRF will be coded using the most updated version of MedDRA dictionary at the time of clinical database lock. Medical history will be collected at Visit 10 (Screening Visit). The number and percentage of participants with each medical condition will be provided by treatment group and system of organ class for the FAS.

2.4 Treatments (study treatment, rescue medication, concomitant therapies, compliance)

2.4.1 Study treatment / compliance

Participants will be randomized to be treated during a 16-week randomized treatment period with one of the following arms targeting a final ratio of 2:2:3:3:2.

- Placebo matching XXB750
- XXB750 60 mg target dose
- XXB750 120 mg target dose
- XXB750 240 mg target dose in one of two up-titration regimens
- Sacubitril/valsartan at target doses of 97/103 mg bid (only participants previously treated with ACEI/ARB)

Treatment groups may be modified per adaptive design feature of the study design (refer to study protocol Section 4.6).

Table 2-2 Study treatment dose levels during randomized treatment epoch

Dose Level	XXB750 arms	Sacubitril/valsartan
0	Placebo or interruption	Interruption
1	XXB750 60 mg	50 (24/26) mg bid
2	XXB750 120 mg	100 (or 49/51) mg bid
3	XXB750 240 mg	200 (or 97/103) mg bid

Treatment exposure during the randomized treatment period

<u>The duration of the randomized treatment period exposure for a participant, regardless of temporary interruptions</u>, is defined as:

- For participants randomized to XXB750 dose arms or placebo arm: min(date of last study drug administration + 28 days, date of death, date of informed consent withdrawal) – first study drug administration date + 1.

- For a participant randomized to open-label sacubitril/valsartan: min(date of last study drug administration, date of death, date of informed consent withdrawal) – first study drug administration date + 1.

Duration of overall randomized treatment exposure, including temporary interruptions, will be summarized descriptively by treatment group (i.e. n, mean, standard deviation, min, Q1, median, Q3, max) and the number (percentage) of participants within the following duration categories will also be provided:

- < 4 weeks
- ≥ 4 weeks to < 8 weeks
- ≥ 8 weeks to < 12 weeks
- ≥ 12 weeks

Overall patient-years on-treatment and average patient-years on-treatment will be reported by each treatment group.

- Overall patient-years on-treatment = Sum of duration of treatment exposure (in days) from all participants/ 365.25
- Average patient-years on-treatment = Overall patient-years on-treatment / Number of participants randomized to the treatment

Number and percentage of participants at each dose level defined in Table 2-2 will also be summarized by the number of study treatment administrations, by visit and treatment. In addition, the frequency of the study drug administrations (excluding temporary interruptions) will be summarized by treatment for XXB750 dose arms and placebo arm. The durations on each dose level will also be summarized by treatment group.

The duration of total exposure to study drug (excluding temporary interruptions) will be computed as:

- For participants randomized to XXB750 dose arms or placebo arm: min(date of last study drug administration + 28 days, date of death, date of informed consent withdrawal) – first study drug administration date + 1 - *number of days of treatment interruption*.
- For a participant randomized to open-label sacubitril/valsartan: min(date of last study drug administration, date of death, date of informed consent withdrawal) – first study drug administration date + 1 - *number of days of treatment interruption*.

The duration of total exposure to study drug (excluding temporary interruptions) will be summarized by treatment group (mean, standard deviation, median, minimum and maximum) as well as frequencies per duration category defined below.

- < 4 weeks
- ≥ 4 weeks to < 8 weeks
- ≥ 8 weeks to < 12 weeks
- ≥ 12 weeks

Average dose will be summarized by treatment group during randomized treatment period and calculated as below:

$$\frac{\sum_{i=0}^{3}(\text{no. of days on dose level i}) \times (\text{dose level i*})}{\sum_{i=0}^{3}(\text{no. of days on dose level i})},$$

where dose level is the dose administered at a visit and number of days will be from the date of injection until the day before next dose administered for XXB750 and placebo treatment groups; and dose level is the total daily dose for open-label sacubitril/valsartan arm.

Dose administration records for randomized treatment period will also be listed at participant level for the participants in safety set.

Number and percentage of participants with permanent treatment discontinuations will be provided by reason for discontinuation. Time from randomization to permanent study treatment discontinuation will be summarized by treatment group by providing Kaplan-Meier estimate of cumulative rate of permanent treatment discontinuation.

All these analyses pertaining to treatment exposure during randomized treatment period will be carried out for all participants in Safety set (SAF).

2.4.2 Prior, concomitant and post therapies

Medications will be identified using Novartis Drug and Therapy Dictionary, NovDTD which is a modified Novartis internal version of the WHO Drug Dictionary Enhanced (DDE) including the Anatomical Therapeutic Chemical (ATC) code. The latest version at the time of clinical database lock will be used.

Prior medications are defined as drugs taken prior to the study medication. **Randomized treatment concomitant medications** are medications taken at any time during the randomized treatment period and prior to safety follow-up. **Safety follow-up concomitant medications** are those given at least once during safety follow-up period. Prior, randomized treatment period or safety follow-up concomitant medication will be identified based on recorded or imputed start and end dates of taking medication. The rules for imputing incomplete start and end dates are described in Section 5.1.

The concomitant medication information for the randomized treatment period and safety follow-up period will be summarized based on the SAF. The prior concomitant medications will be summarized based on SCR and SAF, separately.

Prior and Concomitant medications will be summarized by treatment group. Medications will be presented in alphabetical order by ATC codes and grouped by chemical subgroup (the 4th level of the ATC codes). Tables will also show the overall number and percentage of participants receiving at least one medication of a particular ATC code and at least one drug in a particular chemical subgroup.

The number and percentage of participants on the following classes of background medications during the study will be tabulated by treatment group for FAS and for SAF.

- Aldosterone antagonists
- Anti-diabetic drugs
    - Insulins
    - Oral anti-diabetic drugs
    - SGLT-2 inhibitors
- Antiarrhythmic agents
- Anticoagulants
- Antiplatelet agents (excl. Aspirin)
- Aspirin
- Beta blockers
- Cardiac glycosides (digoxin/digitalis glycoside)
- Calcium antagonists
- Diuretics
- Nitrates
- Other lipid lowering agents (excl. Statins)
- Statins

The search criteria for the classes of medications listed above will be defined in a separate EXCEL sheet with ATC preferred term and WHO drug codes. The EXCEL sheet will be stored in SUBWAY at the RAP level after the content is agreed upon by the Global Program Medical Director or Clinical Head (GPMD/GPCH) and before clinical database lock (CDBL).

Standard background HF medication

Standard background HF medications (or auxiliary medicinal products under EU CTR) in this study are ACEI, ARB and sacubitril/valsartan. The mean daily dose for the background HF medication will be calculated as:

Mean daily dose = [(No. of days on dose level 1)* level 1 daily dose + (No. of days on dose level 2)*level 2 daily dose + … + (No. of days on dose level K)*level K daily dose]/(No. of days in respective period of interest)

Please refer to Section 2.1.1 for the definition of each period. The summary for standard background HF medications will be presented for randomized treatment period using FAS.

2.5 Analysis supporting primary objective(s)

Please refer to study protocol Section 3 for the primary objective and the corresponding estimand and Section 9 for related statistical analyses. The primary analysis will be performed after all participants have completed Week 16 (or discontinued before Week 16). A final analysis will be performed after all participants have completed Week 24 (or discontinued before Week 24). Formal testing of the primary endpoint with full level alpha of 0.025 (1-sided) will be performed at the primary analysis timepoint.

2.5.1 Primary endpoint(s)

The primary efficacy variable is change in log NT-proBNP from baseline to Week 16, which is equivalent to the log of ratio of Week 16 NT-proBNP over baseline NT-proBNP.

Missing values at Week 16 will be imputed as described in Section 2.5.3 and Section 2.5.4.

2.5.2 Statistical hypothesis, model, and method of analysis

The primary objective of determination of a dose-response signal and to characterize the dose-response relationship in XXB750 doses compared to placebo will be evaluated using an optimally weighted contrast test following the Multiple Comparison Procedure-Modeling (MCP-MOD) methodology described in Pinheiro et al 2006 and Pinheiro et al 2014.

A candidate model set is defined corresponding to the range of the expected mean response in each of the dose groups. The candidate model set is used to generate a set of weights for the calculation of optimal contrasts vectors to test the hypothesis of a flat dose-response curve, where XXB750 dose-groups and placebo have the same group mean for the change from baseline. Multiple candidate models correspond to multiple contrast vectors, hence a multiple contrast test is performed based on using the maximum contrast test as a test statistic. A critical value for the maximum contrast test statistic is derived based on the multivariate t distribution, with correlation matrix induced by the correlation of the different contrast test statistics.

Test of the dose response signal

The null hypothesis of a flat dose-response relationship for the change from baseline in logarithm NT-proBNP compared to placebo will be tested at a 1-sided significance level of 2.5% against the alternative hypothesis of a dose-response relationship leading to a significant reduction in the NT-proBNP.

Hence, the following null and alternative hypotheses will be tested:

- **H_{10}**: there is no dose-response relationship for XXB750 (i.e. the dose response relationship is flat and all XXB750 doses have the same group mean as placebo).
- **H_{11}**: there is a dose-response relationship for XXB750 (i.e. there is more reduction in NTproBNP in XXB750 doses based on the assumed candidate models).

There are five candidate models to capture the shape of the dose-response relationship for XXB750 at the Week 16 endpoint, as depicted in Figure 2-1. The candidate models generating the contrast weights are described below:

- Model 1: Emax with ED50 at 15 mg dose level
- Model 2: Emax with ED50 at 120 mg dose level
- Model 3: Sigmoid Emax with ED50 at 60 mg dose level and hill parameter h=3
- Model 4: Sigmoid Emax with ED50 at 120 mg dose level and hill parameter h=3
- Model 5: Beta-model with delta1=0.2, delta2=0.3 and scale=288

Figure 2-1 Dose-response curve of candidate models

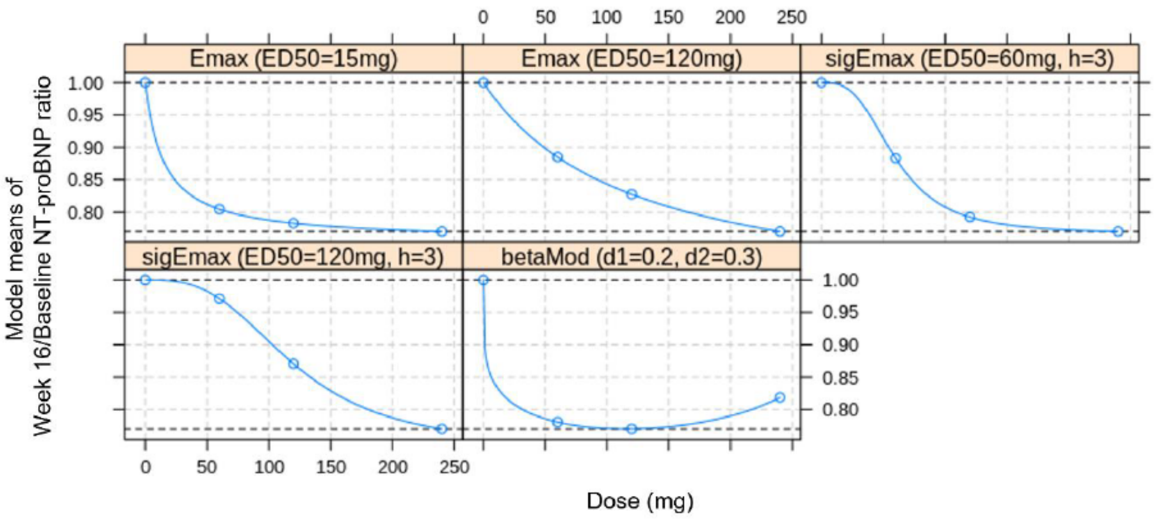

The analysis to derive the test statistics is based on an analysis of covariance (ANCOVA) model with the change from baseline logarithm of NT-proBNP to Week 16 as response variable, treatment (placebo and XXB750 dose groups), LVEF (≤ 40%, > 40%), background medication

stratification factor (ACEI/ARB, sacubitril/valsartan), and region stratification factor as factors, and baseline logarithm of NT-proBNP as a covariate.

The response variable of the change in logarithm NT-proBNP from baseline at Week 16 used in the above ANCOVA model is from an imputed dataset, where a missing Week 16 NTproBNP is imputed using the multiple imputation method as described in Section 2.5.3 and Section 2.5.4. In order to account for the imputation uncertainty, this ANCOVA model will be repeated for each imputed dataset, which results in a set of least squares (LS) mean estimate for all treatment groups and the related covariance matrices. Rubin's rule will be used to combine the multiple sets of LS mean estimates and the related covariance matrices to a single set of LS mean estimates of change of NT-proBNP at Week 16 for all treatment groups and the related covariance matrix.

The optimal contrasts derived from the candidate model sets will be applied to the combined estimated dose means and covariance matrix to obtain t-statistics for each candidate model and the common critical value $C_{0.025}$. $C_{0.025}$ is the common critical value derived from the reference multivariate t-distribution with the 5x5 correlation matrix induced by test the candidate dose response models.

The H_{10} will be rejected and the statistical significance of dose-response in NT-proBNP reduction is established if the max (t1, t2,..., t5) $\geq C_{0.025}$.

Test statistics, t1 to t5, with adjusted p-value for each candidate model will be displayed. The analysis is based on participants in FAS.

Model averaging to obtain the dose response

A parametric bootstrap-based model averaging approach will be implemented to obtain the dose response estimates according to the following steps:

1. The parametric bootstrap procedure will draw a sample of changes of log NT-proBNP from baseline to Week 16 for all doses (including placebo) from a multivariate normal distribution, with mean and covariance matrix determined using Rubin's rule, as described earlier. This sample corresponds to the mean response for each dose (including placebo).
2. Model selection will be performed as follows: general dose-response models as specified will be fit to this bootstrap sample, i.e. Emax, Sigmoid Emax and β-model will be fit to mean response data from placebo and XXB750 doses. The best model based on the gAIC criterion will be selected.
3. The dose response estimate (i.e. model-based estimate of mean change of log NT-proBNP from baseline to Week 16) will be calculated for each dose group, including placebo, using this selected model. The difference in estimated dose response between each dose and placebo will also be calculated.
4. The above procedure (steps 1-4) will be repeated 5,000 times. The mean dose-response estimates by dose group and mean differences of dose-response estimates between each XXB750 dose and placebo, the target doses of interest, as well as their 95% confidence intervals will be calculated based on the quantiles (median, 2.5th and 97.5th percentiles) of these multiple sets of dose-response and target dose estimates generated in step 4.

Dose response results (change with/without placebo substracted and corresponding 95% CI) based on model averaging over all models in the candidate sets as described will be presented. In addition, an ANCOVA model will be fitted to log NT-proBNP change from baseline at Week 16 based on imputed datasets with treatment (placebo and XXB750 dose groups), LVEF (≤ 40%, > 40%), background medication stratification factor (ACEI/ARB, sacubitril/valsartan), and region stratification factor as factors, and baseline logarithm of NT-proBNP as a covariate. Estimates (change with/without placebo substracted and corresponding 95% CI) from ANCOVA model are combined based on Rubin's rule and presented alongside model averaging estimates. A figure of dose response shape based on model averaging will also be displayed.

Summaries of absolute values and change from baseline in NT-proBNP by treatment group and visit will be presented using statistics as described in Section 2.1 additionally with geometric mean and coefficient of variation. Figures will be produced to visually show the observed mean NTproBNP level by visit over study period (randomized treatment period and safety follow-up period) for each treatment group.

The FAS will be used for the above analyses.

2.5.3 Handling of intercurrent events

Intercurrent events for the primary endpoint are defined in Section 1.2.1 and the strategy for handling of intercurrent events in the primary analysis is described below:

1. Discontinuation of study treatment: NT-proBNP data collected after discontinuation of study treatment will be used in the analysis (treatment policy strategy).
2. Unforeseen change in the dose of allowed concomitant medications: NT-proBNP data collected after unforeseen change in the dose of allowed concomitant medication will be used in the analysis (treatment policy strategy).
3. Receiving prohibited concomitant medication: NT-proBNP data collected after receiving prohibited concomitant medication will be used in the analysis (treatment policy strategy).

If a missing primary endpoint occurs after intercurrent event(s), it will be imputed based on participants with observed endpoint after same type of intercurrent event(s) in the respective treatment group. The assumed treatment effect for participants with missing primary endpoint after intercurrent event(s) is similar as those from the same treatment group who have same type of intercurrent event(s) but have observed data (retrieved dropout). Multiple imputations (MIs) based on the subset of retrieved dropouts (i.e., participants who have intercurrent event(s) but have observed measurements after intercurrent event(s)) are generated, resulting in multiple imputed datasets (100). The imputation model with fully conditional method (FCS) will include longitudinal sequence of log NT-proBNP collected at baseline, Week 4, Week 8, Week 12 and Week 16 visits, LVEF (≤ 40%, > 40%), background medication stratification factor (ACEI/ARB, sacubitril/valsartan), and region stratification factor, imputing for each treatment group separately. SAS proc MI FCS statement is used to impute missing values in the following sequence: factor variables in the increasing order of missing frequency, the log NT-proBNP at baseline, Week 4, Week 8, Week 12 and Week 16.

If there is no or very limited participants with observed endpoint after intercurrent event(s) in the respective treatment group, then the missing primary endpoint will be imputed based on the

respective treatment group combined with lower dose group(s). If there are still very limited participants with observed endpoint after intercurrent event(s), then missing will be imputed based on those with observed endpoint in the placebo group.

2.5.4 Handling of missing values not related to intercurrent event

Missing data for the primary endpoint will be imputed using a multiple imputation approach assuming that missingness mechanism can be retrieved from observed data (missing at random (MAR)). The imputation model will include the longitudinal sequence of log NT-proBNP data collected at randomization, Week 4, Week 8, Week 12, and Week 16 visits, LVEF factor (≤ 40%, > 40%), background medication stratification factor (ACEI/ARB, sacubitril/valsartan), region stratification factor, and other baseline variables as appropriate, imputing for each treatment group separately. SAS proc MI FCS statement is used to impute missing values in the following sequence: factor variables in the increasing order of missing frequency, the log NT-proBNP at baseline, Week 4, Week 8, Week 12 and Week 16.

2.5.5 Multiplicity adjustment

Please refer to Section 2.5.2 for primary analysis of MCP-MOD for multiplicity adjustment with respect to testing for a dose response signal across multiple candidate shapes. The secondary hypotheses included in the order of the hierarchical testing procedure are an evaluation and demonstration of: (1) treatment effect of highest XXB750 target dose level versus placebo; (2) combined two highest XXB750 target dose levels versus conversion to sacubitril/valsartan in participants receiving ACEI/ARB background medication. The secondary hypotheses will be tested in the order described above and statistical inference will be made only if the primary hypothesis is rejected.

2.5.6 Sensitivity analyses

The primary analysis for which missing data imputation is based on missing at random or retrieve dropouts would favor the XXB750 treatment groups when early treatment/study discontinuation is due to XXB750 related adverse events or if the participant dies. Placebo multiple imputation (MI) method will be performed, when participants randomized to XXB750 treatment groups are assumed to be similar to placebo treated participants after above mentioned events. MIs based on the participants in placebo arm are generated, resulting in multiple imputed datasets (100). The imputation model with fully conditional method (FCS) will include longitudinal sequence of log NT-proBNP collected at baseline, Week 4, Week 8, Week 12 and Week 16 visits, LVEF factor (≤ 40%, > 40%), background medication stratification factor (ACEI/ARB, sacubitril/valsartan), region stratification factor. SAS proc MI FCS statement is used to impute missing values in the following sequence: factor variables in the increasing order of missing frequency, the log NT-proBNP at baseline, Week 4, Week 8, Week 12 and Week 16.

2.5.7 Supplementary analyses

Supportive analyses for the primary analysis results are listed below:

1. Results based on the single best dose response model fit will also be reported.

2. The mixed model for repeated measure (MMRM) analysis of covariance (ANCOVA) with treatment, LVEF (≤ 40%, >40%), background medication stratification factor (ACEI/ARB, sacubitril/valsartan), and region stratification factor, visit (Week 4, Week 8, Week 12, or Week 16), and treatment-by-visit as factors, and baseline logarithm NT-proBNP as a covariate, with a common unstructured covariance matrix among visits will be used. Treatment comparisons at Week 16 will be provided. The analysis will be based on all available data up to Week 16 in the FAS and based on likelihood method with an assumption of missing at random for missing data.

3. In addition to treatment policy strategy to handle the intercurrent events in the primary analysis, a supplemental analysis based on a hypothetical strategy will be also used to handle the intercurrent events. This strategy assumes that premature discontinuations of study treatment, change in the dose of allowed concomitant medication, receiving prohibited concomitant medication or lost to follow-up/discontinue study would not have occurred in the participants. This analysis uses partial data available up to the occurrence of the intercurrent events, and missing data after the intercurrent events will be imputed as based on the pattern of other participants who do not have intercurrent events (missing at random). The imputation steps are same as described in Section 2.5.4 and the newly imputed data will be used in the analysis as described in Section 2.5.2.

4. Analysis based on the while on-treatment strategy to handle the intercurrent of premature discontinuations of study treatment will be performed. This analysis uses the data taken at the end of treatment visit as the Week 16 assessment and repeat the analysis described in Section 2.5.2.

5. Treatment grouping is based on highest dose received and repeat the analysis described in Section 2.5.2.

6. Subgroup analyses will be performed for the primary analysis. The analysis will be done using the analysis specified in Section 2.5.2 at individual subgroup level separately. Please see Section 2.2.1 for subgroup details.

Summary statistics for NT-proBNP and change from baseline will be displayed by treatment group for observed and imputed values. Figures will be produced to visually show the observed and the imputed mean changes by visit over 16 weeks randomized treatment epoch for each treatment group. FAS will be used for the analyses.

2.6 Analysis supporting secondary objectives

The secondary efficacy endpoints are described in Section 2.6.1 and are part of the hierarchical testing strategy. Analyses related to safety endpoints are described in Section 2.7.

2.6.1 Secondary endpoint(s)

There are two efficacy secondary objectives:

1. To evaluate the treatment effect of the highest XXB750 target dose level compared to placebo in reducing NT-proBNP from baseline at Week 16 in HF patients treated with standard of care, including ACEI/ARB or sacubitril/valsartan.

2. To evaluate the treatment effect of combined two highest XXB750 target dose levels in addition to a background of ACEI/ARB versus conversion from ACEI/ARB to sacubitril/valsartan, in reducing NT-proBNP from baseline to Week 16.

2.6.2 Statistical hypothesis, model, and method of analysis

The efficacy variable in secondary objective 1 is the change from baseline at Week 16 in logarithm of NT-proBNP and will be analyzed using a repeated measures ANCOVA model with treatment (highest XXB750 target dose level or placebo), LVEF (≤ 40%, >40%), background medication stratification (ACEI/ARB, sacubitril/valsartan), and region stratification factor, visit (Week 4, Week 8, Week 12, or Week 16), and treatment-by-visit as factors, and baseline logarithm of NT-proBNP as a covariate, with a common unstructured covariance matrix among visits. Within treatment estimates and between treatment comparisons at Week 16 will be provided. Please refer to Section 1.2.2 and Section 2.5.3 for the intercurrent events and its handling strategy and Section 2.5.4 for handling missing data not related to intercurrent event. The analysis will be run on each of the imputed datasets as described in Section 2.5.2. Overall results, including adjusted mean change at Week16 within each treatment arm, the difference in mean change at Week 16 between treatment arm and its 95% confidence interval (CI), are obtained by applying Rubin's rules on the estimates obtained from imputed datasets.

If the above mentioned model does not converge, the model will be modified by replacing the common unstructured covariance matrix with a compound symmetric matrix between treatment groups and provide the above mentioned estimates.

The efficacy variable in secondary objective 2 and analysis model is the same as those described above in secondary objective 1, except that treatments being compared are combined two highest XXB750 target dose levels with background medication of ACEI/ARB stratum versus conversion from ACEI/ARB to sacubitril/valsartan.

The analysis will be based on all participants in FAS.

The statistical testing procedure described below will be carried out for the multiple treatment comparisons in the primary and secondary efficacy objectives to ensure that the overall significance level for the multiple hypotheses to be tested is controlled at the one-sided 0.025 level (i.e. two-sided 0.05).

The testing hypotheses associated with secondary efficacy objectives are described below:

- **H_{20}:** No difference in the change of NT-proBNP from baseline at Week 16 between the highest XXB750 target dose level versus placebo.
- **H_{30}:** No difference in the change of NT-proBNP from baseline at Week 16 between the combined two highest XXB750 target dose levels in addition to a background of ACEI/ARB versus conversion from ACEI/ARB to sacubitrial/valsartan.

The testing procedure is to be based on the sequentially rejective multiple test procedure pre-specified in controlling overall type I error (Bretz et al 2009) across primary and secondary endpoints. The primary hypothesis H_{10} will be tested first at the one-sided 0.025 significance level. If the primary hypothesis can be rejected, the relocation of its significance level with a

weight of 1 to the other hypotheses is specified in Figure 2-2 below. The sequentially rejective multiple test procedure is continued until no more hypothesis can be further rejected.

Figure 2-2 Sequentially rejective multiple testing procedure

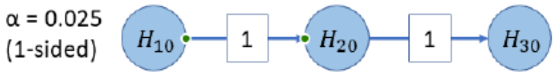

2.6.3 Handling of intercurrent events

Refer to Section 2.5.3.

2.6.4 Handling of missing values not related to intercurrent event

Refer to Section 2.5.4.

2.6.5 Sensitivity analyses

The secondary analysis using retrieved dropouts or missing at random (MAR) approaches for missing values after early treatment/study discontinuation due to adverse event(s) or death may assign values in the XXB750 dose groups that are too favorable. Therefore, a sensitivity analysis will be performed where missing values after early treatment/study discontiuation due to AE or death will be multiply imputed based on results from similar participants in the placebo group.

Placebo multiple imputation method described in Section 2.5.6 will be performed, where participants randomized to XXB750 dose groups are assumed to be similar to placebo treated participants after drop-out.

2.6.6 Supplementary analyses

The supportive analyses for the secondary analysis results are listed below:

1. Analysis based on the while on-treatment strategy to handle the intercurrent of premature discontinuations of study treatment will be performed. This analysis uses the last on-treatment data defined in Section 2.1.1 as the Week 16 assessment and repeat the analysis described in Section 2.6.2.
2. Subgroup analyses will be performed for the secondary comparisons. To explore homogeneity of treatment effects in subgroups, the estimated between treatment difference, two-sided 95% confidence interval, and p-value for the treatment-by-subgroup interaction will be provided for each of the subgroups based on the analysis model described in Section 2.6.2, in which treatment, LVEF (≤ 40%, >40%), background medication stratification (ACEI/ARB, sacubitril/valsartan), and region stratification factor, visit (Week 4, Week 8, Week 12, or Week 16), subgroup, treatment-by-subgroup and treatment-by-visit as fixed effect factors. Please see Section 2.2.1 for subgroup details.

2.7 Safety analyses

For all safety analyses, the SAF set will be used. All listings, figures and tables will be presented by treatment group.

Safety data to be analyzed are listed as below:

- Adverse events (AE) and Serious Adverse events (SAE)
- Safety topics that are adverse events of special interest (AESI): Serious hypotension, Serious hypersensitivity reactions, Serious injection site reactions, Severe tachycardia, Severe acute bradycardia, and Serious presyncope/syncope
- Safety topics that are potential risks: Hypotension, Hypersensitivity reactions, Injection site reactions, Tachycardia, Bradycardia, Immunogenicity
- Safety topics that are neither AESI nor Potential risks: Renal toxicity and Hepatotoxicity
- Laboratory assessments
- Vital signs, e.g., weight, systolic blood pressure (SBP), diastolic blood pressure (DBP), and pulse rate.

2.7.1 Adverse events (AEs)

Any AE that occurred during the study period will be included in AE summary tables by the specific treatment period as described in Table 2-3, i.e., AEs that occurred in one of the following periods: (1) randomized treatment period , (2) safety follow-up period, and (3) randomization period (consisting of randomized treatment period and safety follow-up period).

The analysis will consider all participants in the Safety set (SAF) for reported AEs during the randomization period, randomized treatment period and safety follow-up period.

Table 2-3 Allocation of AEs (randomized treatment period and safety follow-up period listed separately from randomization period)

Screening visit (V10)	Randomized treatment (V101-V9998)	Safety follow-up (V201-V202)	Phase AE to be reported in
	* Randomization period		
X			Reported by site from informed consent;
	X		Report one AE with onset date X for Randomized treatment (also randomization period);
	X, X2		Report as one AE for Randomized treatment period (* Randomization period) with corresponding onset date and severity depending on analysis
	X	X2	Report as two separate AEs:<br>One with onset date X for Randomized treatment, and one with onset date X2 for Safety follow-up;<br>* One for randomization period with corresponding onset date and severity depending on analysis
		X, X2	Report as one AE with onset date X for

Screening visit (V10)	Randomized treatment (V101-V9998)	Safety follow-up (V201-V202)	Phase AE to be reported in
	* Randomization period		
			for Safety follow-up (* Randomization period) with corresponding onset date and severity depending on analysis
X and X2 are same PT with an increased severity, with onset date of X2 being the date when the severity increases (which is reported as a separate AE entry in clinical database). Similarly, same reporting rule is used for a new AE with same PT occurs after end date of X or X2.			

Treatment emergent adverse events (new or worsened) during a specific period (randomization, randomized treatment or safety follow-up) are defined as any recorded AE with its start date (recorded or imputed) later than or equal to the start date of the specific period as defined below:

1) Randomization period: first date of study treatment injection.
2) Randomized treatment period: first date of study treatment injection.
3) Safety follow-up: end of study treatment date + 1

The incidence of treatment-emergent adverse events (new or worsened) will be summarized by primary system organ class, preferred term, severity, and relationship to study drug according to the Medical Dictionary for Regulatory Activities (MedDRA). The latest MedDRA version before clinical database lock will be used for reporting the study.

Within each reporting period (randomized treatment period, safety follow-up period or randomization period, see Table 2-3), the following rules are applicable. If a participant reported more than one adverse event with the same preferred term, the adverse event with the greatest severity will be presented. If a participant reported more than one adverse event within the same primary system organ class, the participant will be counted only once with the greatest severity at the system organ class level, where applicable. Statistical analyses, which will be performed for the randomized treatment period and other periods as appropriate, will include all AEs with onset date during the defined period and up to the analysis cut-off irrespective of how long after the last day of study treatment they occurred.

The number and percentage of participants reporting any adverse event during each reporting period will be summarized by primary system organ class, preferred term and treatment. The most common adverse events reported (≥ 5% in any group for each preferred term in the SOC-PT table) will be presented in descending frequency according to its incidence in the XXB750 highest dose group starting from the most common event.

Separate summaries for randomized treatment period will be provided for study medication related adverse events, deaths, serious adverse events, other significant adverse events leading to dose adjustment, and permanent study discontinuation.

Separate summaries for safety follow-up period will be provided for study medication related adverse events, deaths, serious adverse events, and permanent study discontinuation.

AESI and potential risks will be summarized separately in addition to the above analysis. Please see Section 2.7.2 for details.

2.7.2 Adverse events of special interest and potential risks

The following specific safety topics will be summarized separately in addition to the above analysis:

- AESI
    - Serious hypotension
    - Serious hypersensitivity reactions
    - Serious injection site reactions
    - Severe tachycardia
    - Severe acute bradycardia
    - Serious presyncope/syncope
- Potential risks:
    - Hypotension
    - Tachycardia
    - Bradycardia
    - Hypersensitivity reactions
    - Injection site reactions
    - Immunogenicity
- Other safety topics
    - Renal toxicity
    - Hepatotoxicity

The sets of terms, pertaning either to MedDRA or to NMQ or to CMQ, that are used to search the database to retrieve cases of interest that relate to the safety topics listed above are stored (or alternatively "summarized") in the Case Retrieval Strategy (eCRS). The latest version of the eCRS at the time of clinical database lock will be used.

The following standard analyses will be provided for all the listed safety topics (excluding Immunogenicity):

- Incidence (absolute and relative frequency) rates in terms of participant regardless of causal relationship to study drug for randomized treatment period and randomization period, by treatment group.
- Exposure-adjusted incidence rates per 100 patient years regardless of study drug relationship for randomized treatment period and randomization period, by treatment
- Analysis for time-to-first selected safety topic of interest by treatment group will be performed for randomized treatment period and randomization period using Kaplan-Meier estimates
- Listing of AEs by participant with AESIs and risk information.

In addition to the above, the analysis specified below will be provided for the safety topics that are listed in Table 2-4.

Table 2-4 AESIs and potential risks with additional analysis

Safety topic	Definitions	Analysis and additional criteria for characterizing selected AEs
Hypotension	Hypotension [STANDARD] (NMQ)	Distribution of TEAE by SOC and PT<br>Distribution of TEAE by pre-defined subgroups (as per Table 2-1)
Tachycardia	ADR Tachycardia [ADR_STD] (NMQ)	Distribution of TEAE by SOC and PT<br>Distribution of TEAE by pre-defined subgroups (as per Table 2-1)
Bradycardia	ADR Heart rate decreased [ADR_STD] (NMQ)	Distribution of TEAE by SOC and PT<br>Distribution of TEAE by pre-defined subgroups (as per Table 2-1)
Injection site reactions	ADR Injection site reaction [ADR_STD] (NMQ)	Distribution of TEAE by SOC and PT<br>Distribution of TEAE by pre-defined subgroups (as per Table 2-1)
Hypersensitivity reactions	(Anaphylactic/anaphylactoid shock conditions (SMQ) OR Anaphylactic reaction (SMQ) OR Hypersensitivity other than administration site reactions [XXB750] (NMQ))	Distribution of TEAE by SOC and PT<br>Distribution of TEAE by pre-defined subgroups (as per Table 2-1)
Immunogenicity	All serious and non-serious TEAE in participants with at least one positive ADA test ever	Distribution of TEAE by ADA subgroup (as per Table 2-1)
Serious hypotension	Only SAE: ADR Hypotension [ADR_STD] (NMQ)	Distribution of TEAE by SOC and PT<br>Distribution of TEAE by pre-defined subgroups (as per Table 2-1)
Serious hypersensitivity reactions	Only SAE: (Anaphylactic/anaphylactoid shock conditions (SMQ) OR Anaphylactic reaction (SMQ) OR Hypersensitivity other than administration site reactions [XXB750] (NMQ))	Distribution of TEAE by SOC and PT<br>Distribution of TEAE by pre-defined subgroups (as per Table 2-1)

Safety topic	Definitions	Analysis and additional criteria for characterizing selected AEs
Serious injection site reactions	Only SAE: ADR Injection site reaction [ADR_STD] (NMQ)	Distribution of TEAE by SOC and PT<br>Distribution of TEAE by pre-defined subgroups (as per Table 2-1)
Severe tachycardia	Only severe AE: ADR Tachycardia [ADR_STD] (NMQ)	Distribution of TEAE by SOC and PT<br>Distribution of TEAE by pre-defined subgroups (as per Table 2-1)
Severe acute bradycardia	Only severe AE: ADR Heart rate decreased [ADR_STD] (NMQ)	Distribution of TEAE by SOC and PT<br>Distribution of TEAE by pre-defined subgroups (as per Table 2-1)
Serious presyncope/syncope	Only SAE: ADR Presyncope [ADR_STD] (NMQ) OR ADR Depressed level of consciousness [ADR_STD] (NMQ) OR ADR Loss of consciousness [ADR_STD] (NMQ)	Distribution of TEAE by SOC and PT<br>Distribution of TEAE by pre-defined subgroups (as per Table 2-1)

2.7.3 Deaths

Participants that died during the study period will be reported separately for randomized treatment period and safety follow-up. Deaths will be summarized by actually received treatment group to present number and percentage of participants that died. In addition, listings will be provided for participants that died.

The analysis will consider all participants in the Safety set (SAF) for reported deaths during the randomization period.

2.7.4 Laboratory data

For each laboratory parameter, evaluations will be summarized by visit and actually received treatment group by presenting summaries (n, mean, standard deviation, median, minimum and maximum) for actual and change from baseline values for both run-in and randomization period. The summary will be provided separately for biochemistry and hematology laboratory parameters. Central laboratory data will be used for the summaries.

Shift tables based on the standard ranges for each laboratory parameter (biochemistry and hematology) will be provided by treatment group at each visit for the randomization period to present incidence of transitions from a baseline high, normal or low laboratory value to a maximum post-baseline high, normal or low value. In addition to these analyses, laboratory

parameter values will be listed for each participant by scheduled assessment visit and treatment group.

The number and percentage of participants with clinically notable laboratory results after baseline will be presented in accordance with Table 2-5, by visit and overall, for randomized treatment period and other periods as appropriate.

Table 2-5 Clinically notable laboratory values and vital signs

Category<br>Parameter (unit)	Clinically notable criteria
**Hematology**	
Red blood cell count	> 50% increase AND value > ULN<br>> 30% decrease AND value < LLN
Hemoglobin	> 50% increase AND value > ULN<br>either (> 30% decrease AND value < LLN) OR value < 8.0 g/dL
Hematocrit	> 50% increase AND value > ULN<br>> 30% decrease AND value < LLN
White blood cell count	value > 100,000 cells/mm^3<br>value < 2000 cells/mm^3
Platelet count	value < 50,000 platelets/mm^3
**Blood chemistry**	
Alkaline phosphatase	value > 5 x ULN if baseline was normal OR value > 5 x baseline if baseline was abnormal
ALT (SGPT)	value > 5 x ULN if baseline was normal OR value > 5 x baseline if baseline was abnormal
AST (SGOT)	value > 5 x ULN if baseline was normal OR value > 5 x baseline if baseline was abnormal
Total bilirubin	value > 3 x ULN if baseline was normal OR value > 3 x baseline if baseline was abnormal
BUN	≥ 50% increase
Creatinine	≥ 50% increase
Potassium	value > 6.0 mmol/L<br>value < 3.0 mmol/L
Chloride	> 115 mEq/L<br>< 90 mEq/L
Calcium	corrected serum calcium > 3.1 mmol/L<br>corrected serum calcium < 1.75 mmol/L
Uric acid	> 50% increase
Plasma glucose	> 25% increase<br>value < 40 mg/dL
**Vital signs**	
Weight (kg)	Decrease > 7% from baseline<br>Increase > 7% from baseline

Category<br>Parameter (unit)	Clinically notable criteria
Sitting office SBP (mmHg)	> 180 mmHg
	< 110 mmHg
Sitting office DBP (mmHg)	> 110 mmHg
	< 70 mmHg
Pulse (bpm)	value > 100
	1. value < 60
	2. value < 50
**Liver function tests**	
ALT (SGPT) OR AST (SGOT)	1. value > 3 x ULN
	2. value > 5 x ULN
	3. value > 8 x ULN
	1. value > 10 x ULN
	2. value > 20 x ULN
ALT (SGPT) OR AST (SGOT) AND Total bilirubin (TB)	1. ALT or AST > 3 x ULN and TB > 1.5 x ULN
	2. ALT or AST > 3 x ULN and TB > 2 x ULN
	3. ALT or AST > 5 x ULN and TB > 2 x ULN
	4. ALT or AST > 8 x ULN and TB > 2 x ULN
	5. ALT or AST > 10 x ULN and TB > 2 x ULN
	6. ALT or AST > 20 x ULN and TB > 2 x ULN
Alkaline phosphatase	1. > 2 x ULN
	2. > 3 x ULN
	3. > 5 x ULN
Total bilirubin	1. > 1.5 x ULN
	2. > 2 x ULN
	3. > 3 x ULN
Alkaline phosphatase (ALP) AND TB	1. ALP > 3 x ULN AND TB > 2 x ULN
	2. ALP > 5 x ULN AND TB > 2 x ULN
ALT (SGPT) OR AST (SGOT) AND Total bilirubin (TB) AND Alkaline phosphatase (ALP)	1. ALT OR AST > 3 x ULN AND TB > 2 x ULN AND ALP ≤ 2 x ULN
	2. ALT OR AST > 3 x ULN AND TB > 2 x ULN AND ALP ≤ 2 x ULN OR reported Hy's Law case
	3. TB > 3 x ULN AND AST OR ALT ≤ 3 x ULN AND ALP ≤ 1.5 x ULN
	4. ALP > 3 x ULN AND AST AND ALT AND TB are within normal range
Laboratorial and clinical associations	ALT OR AST > 3 x ULN AND (Nausea OR Vomiting OR Fatigue OR General malaise OR Abdominal pain OR (Rash AND Eosinophilia))

Category<br>Parameter (unit)	Clinically notable criteria
**Renal function tests**	
eGFR	1. > 25% decrease
	2. > 40% decrease
	3. > 50% decrease
	4. > 30 mL/min/1.73m^2 decrease
	5. Participants meeting any of the criteria 1 to 4 above
Creatinine	1. ≥ 0.3 mg/dL increase
	2. 1.5 to 1.9 x baseline
	3. 2.0 to 2.9 x baseline
	4. ≥ 3.0 x baseline
	5. ≥ 4.0 mg/dL
	6. Participants meeting either criterion 1 OR 2 above
	7. Participants meeting either criterion 4 OR 5 above
New onset of proteinuria	Urine protein ≥ 300 mg/dL
New onset of hematuria	Urine hemoglobin ≥ 1 mg/dL

ULN: upper limit of the normal range; LLN: lower limit of the normal range; SBP: systolic blood pressure; DBP: diastolic blood pressure; Increases and decreases of parameters as mentioned in the table are from baseline

Participants with liver function tests (ALT, AST, ALP, Total Bilirubin) and renal-related parameters falling within predefined categories of elevations (new and worsened [i.e., those existent before, and that worsened after, randomization] elevations) will be summarized by treatment group in accordance with Table 2-5 for both randomized treatment period and randomization period. Descriptive summaries by presenting count and percentage of participants with each type of Liver and Renal event in addition to graphical summaries will be displayed, as applicable.

2.7.5 Other safety data

2.7.5.1 ECG and cardiac imaging data

The following quantitative variables will be summarized: heart rate and QRS duration. Summary statistics for continuous data and number and percentage of participants for categorical data (e.g. interpretation) will be provided by treatment and visit for randomization period.

In addition, shift tables comparing the existence or not of abnormalities (atrial fibrillation, atrial flutter, LBB block, RBB block, left ventricular hypertrophy, paced rhythm, myocardial infarction, 1st degree AV block, 2nd degree AV block, 3rd degree AV block or other) on the ECG at baseline and worst-on study results (during the randomized treatment period and during the randomization period) will be provided. The categories Yes, No and Missing will be used to reflect where the abnormality of interest is present, absent or an ECG is not performed/is not available, respectively for that participant.

The analysis will be based on SAF.

2.7.5.2 Vital signs

Vital signs including weight, blood pressure and pulse measures will be summarized by treatment group and scheduled visit with standard summary statistics (mean, Q1, median, Q3, standard deviation, min, max), including changes from randomization baseline. Graphical mean plots with 95% CIs for these vital signs will also be provided. Change from baseline will only be summarized for participants with both baseline and post-baseline values and will be calculated as:

- change from baseline = post-baseline value – baseline value

The number and percentage of participants with clinically notable vital signs after baseline will be presented in accordance with Table 2-5 for randomized treatment period and randomization period, by visit and overall. The analysis will be based on SAF.

Apart from the above analyses, values for vital signs parameters will also be listed at a participant level by treatment group and visit for all participants in safety set (SAF).

Maximum and minimum post-baseline values will be summarized (mean, Q1, median, Q3, standard deviation, min, max) for blood pressure and pulse measures for randomized treatment period and for randomization period.

2.7.5.3 Immunogenicity

All immunogenicity results including anti-XXB750 antibodies (ADA) will be summarized by treatment group and listed by treatment group, participant, and visit/time. Summary statistics including prevalence and incidence of anti-XXB750 antibodies will be provided by treatment and visit/time. A shift table to summarize the proportion of participants' ADA status change over time from baseline (i.e. participants transitioning from negative to positive or vice versa) will also be provided by treatment group.

Post-Baseline category is defined as positive if ADA screening test turns positive at any time post-dose during randomization period of the study, as negative if ADA screening test is negative at all times post-dose during randomization period of the study, and as undefined otherwise. Treatment-induced ADA-positive sample is defined as ADA-positive sample post-baseline during randomization period with ADA-negative sample at baseline. Treatment-boosted ADA-positive sample is defined as ADA-positive sample post-baseline with titer that is at least the titer fold (i.e. 4-fold) change greater than the ADA-positive baseline titer.

A comparison between the counts and percentages of AE observed in ADA-positive and ADA-negative participants will be performed for each MedDRA PT.

The analysis will be based on immunogenicity prevalence set and incidence set.

2.8 Pharmacokinetic endpoints

Sparse PK samples are collected for population PK analysis. Results will be reported separately. Individual drug concentration will be listed in CSR (Clinical Study Report) for participants in PK sub study.

2.9 PD and PK/PD analyses

See Section 2.11 for PD (excluding NT-proBNP) related analyses. See Sections 2.5 and 2.6 for NT-proBNP related analyses.

2.10 Patient-reported outcomes

See Section 2.12 for Kansas City Cardiomyopathy Questionnaire (KCCQ) related analyses.

2.11 Biomarkers

To better satisfy the normality assumption, the log-transformation will be taken on each biomarker prior to statistical analysis. Biomarkers to be analyzed may include, but are not necessarily limited to: cyclic GMP (plasma and urine), urinary cyclic GMP-to-creatinine ratio, urinary albumin-to-creatinine ratio, BNP and hs-Troponin.

The change from baseline to a pre-defined time-point during randomized treatment period in logarithmic scale, i.e. log (post-baseline/baseline), will be analyzed using a repeated measures ANCOVA model with treatment, LVEF (≤ 40%, >40%), background medication stratification (ACEI/ARB, sacubitril/valsartan), and region stratification factor, visit (Week 8, Week 12, or Week 16), and treatment-by-visit as factors, and baseline logarithm baseline biomarker value as a covariate, with a common unstructured covariance matrix among visits. The estimated treatment effects in terms of ratios of geometric means, based on the least-squared means for within and between treatment groups from the ANCOVA model, and the corresponding two sided 95% confidence intervals will be provided. The analysis will be performed based on all available data in the FAS and based on likelihood method with an assumption of missing at random for missing data.

In addition, biomarker data will be summarized by treatment and time point. Descriptive summaries will include n, mean (arithmetic and geometric), SD, minimum, Q1, median, Q3, maximum, coefficient of variation, and 95% confidence interval of geometric mean. Summary statistics by treatment group will be performed for the baseline values, the post-baseline values, and the change from baseline, with baseline defined as the randomization visit. At each post baseline visit, only participants with a value at both baseline and that post-baseline visit will be included. Missing post-baseline values will not be imputed. Analyses will be based on the Full Analysis Set (FAS).

2.12 Other Exploratory analyses

2.12.1 Exploratory variables

Below are the variables for the exploratory analysis:

1. Number of winners based on the hierarchically ordered composite endpoint consisting of:
    1) time to all-cause death;
    2) number of HF hospitalizations;
    3) categorical KCCQ CSS change from baseline to Week 16;

4) NYHA class change from baseline to Week 16;
5) relative change in NT-proBNP levels from baseline to Week 16.

2. Rate of change in eGFR (eGFR slope) from baseline to Week 16;
3. Change from baseline at assessment visits in biomarkers (See Section 2.11 for analyses);
4. Change in NYHA class from baseline to Week 16;
5. Changes in HF signs and symptoms from baseline to Week 16;
6. Change in all domains of the Kansas City Cardiomyopathy Questionnaire from baseline to Week 16;
7. Population PK analysis results including mathematic models and parameters (See Section 2.8 for details);
8. Incidence of anti-XXB750 antibodies (See Section 2.7.5.3 for analyses).

2.12.2 Analysis methods

In general, exploratory variables will be analyzed in the FAS unless specified otherwise. Nominal 2-sided p-values will be presented for treatment comparisons without control for multiplicity. To better satisfy the normality assumption, the log-transformation will be taken on each biomarker prior to statistical analysis. There will be no multiplicity adjustment for any analysis of exploratory variables.

Analysis of Win-Ratio

The hierarchical ordered composite endpoint will be analyzed using the win ratio approach (Pocock et al 2012), with treatment as fixed-effect factor and stratified by region and background medication (ACEI/ARB, sacubitril/valsartan) (Dong et al 2018). Every participant in the highest XXB750 target dose level group will be compared to every participant in the placebo group within each stratum in the following order:

1. all pairs will be compared first by time to all-cause death; if there is no winner, then
2. they will be compared for the number of HF hospitalizations; if there is no winner, then
3. they will be compared according to the combination of change in KCCQ CSS and change in NYHA class from baseline to Week 16; if still there is no winner, then
4. they will be compared according to reduction in NT-proBNP levels from baseline to Week 16.
5. If the comparison between changes in NT-proBNP levels does not result in a winner, the respective pairwise comparison is tied.

When comparing the time to all-cause death and number of HF hospitalizations between two participants and at least one of the times is censored, the comparison is only performed up to the censoring time. Three categories are defined for the change of KCCQ CSS: worsened (decrease of 5 points or more), improved (increase of 5 points or more), or unchanged (neither worsened nor improved). The comparison of KCCQ CSS categorical change is in favor of the

participant in the highest XXB750 arm if the category of the participant is better compared to that in placebo arm. Similarly, three categories are defined for the change of NYHA class, worsened, improved, or unchanged; and the comparison of NYHA class change is in favor of the participant in the highest XXB750 arm if the class change of the participant is better compared to that in the placebo arm. Please refer to Table 2-6 for the example of combination of change in KCCQ CSS and change in NYHA class.

Table 2-6 Selecting a winner based on combination of change of KCCQ CSS and change of NYHA class

Example	Participant A		Participant B		Result of comparison
	Change of KCCQ CSS from BL to Week 16	Change of NYHA class from BL to Week 16	Change of KCCQ CSS from BL to Week 16	Change of NYHA class from BL to Week 16	
1	Improved	Improved	Improved	Improved	Tie
2	Improved	Worsened	Worsened	Improved	Tie
3	Worsened	Unchanged	Worsened	Worsened	A wins
4	Worsened	Worsened	Worsened	Improved	B wins

The comparison of NT-proBNP is performed based on the relative change between baseline and Week 16 and takes into account that relative changes within ±25% are considered medically irrelevant; in detail, let μ_{XXB750} and μ_{placebo} be the relative changes of participants in the highest XXB750 and the placebo arm, respectively. Then, a comparison of NT-proBNP is in favor of the participant in the highest XXB750 arm if $\mu_{\text{XXB750}} < 0.75\times\mu_{\text{placebo}}$. On the other hand, the comparison is in favor of the participant in the placebo arm if $\mu_{\text{placebo}} < 0.75\times\mu_{\text{XXB750}}$.

The win ratio is estimated by the ratio of the number of winners in the highest XXB750 treatment group and the number of winners in the placebo group with weights defined as the reciprocal of the stratum sample size.

$$\Psi = \frac{\sum_{m=1}^{M} w^{(m)} n_{XXB750}^{(m)}}{\sum_{m=1}^{M} w^{(m)} n_{placebo}^{(m)}}$$

where $n^{(m)}_{\text{XXB750}}$ and $n^{(m)}_{\text{placebo}}$ are the total of wins in the mth stratum for the highest XXB750 and placebo treatment groups, respectively; $w^{(m)}=1/N^{(m)}$ and $N^{(m)}$ is the total sample size in the mth stratum.

The estimated win ratio and the corresponding 2-sided 95% CI will be provided. The contribution to the number of winners of each component of the win ratio will be summarized.

Analysis of continuous variables

Continuous variables, which are assumed to be normally distributed, are:

• Rate of change (slope) in eGFR from baseline at Week 16;

• Change in KCCQ from baseline at Week 16.

For the rate change in eGFR, the eGFR slope will be estimated from a repeated measures ANCOVA model including treatment, LVEF (≤ 40%, > 40%), background medication stratification factor (ACEI/ARB, sacubitril/valsartan), region stratification factor, time (in weeks), and treatment-by-time as fixed effects with random intercept and slope (time) and a common unstructured covariance. The least-square means of slopes for within and between treatment groups, and the corresponding two-sided 95% confidence interval will be provided.

Changes from baseline in KCCQ scores (the clinical summary score of KCCQ and all sub-domain scores) will be analyzed based on an ANCOVA model in which treatment, LVEF (≤ 40%, >40%), background medication stratification factor (ACEI/ARB, sacubitril/valsartan) and region stratification factor will be included as factors and baseline value as a covariate. Treatment comparisons and effect size estimates at Week 16 will be provided. Details of KCCQ scores derivation are provided in Appendix Section 5.6.

In addition, KCCQ scores will be summarized by treatment and time point. Summary statistics by treatment group will be performed for the baseline values, the post-baseline values, and the change from baseline, with baseline defined as the randomization visit. At each post baseline visit, only participants with a value at both baseline and that post-baseline visit will be included.

The analysis will be performed based on participants in the FAS.

Analysis of ordinal variables

The ordinal variables include

• Change in NYHA class from baseline (for categorical analysis in NYHA class change from baseline over time, missing NYHA classification due to death will be imputed as NYHA class worsened);

• Changes in HF signs and symptoms from baseline.

The ordinal variables will be analyzed, at Week 4, Week 8, Week 12, and Week 16, using a repeated measures proportional cumulative odds model. The model will include patient as a random effect and the respective baseline category, treatment, LVEF (≤ 40%, > 40%), background medication stratification factor (ACEI/ARB, sacubitril/valsartan), region stratification factor, visit (all available post-randomization visits) and treatment-by-visit interaction as fixed effect factors. This model assumes that the treatment effect sizes across measurement categories are the same. The visit-wise effect size estimates and their 95% confidence intervals will also be provided. The analysis will be based on all available data in the FAS and likelihood method with an assumption of missing at random for missing data. The SAS procedure of GLIMMIX (SAS/STAT 9.3) can be used for the analysis.

Shift tables of NYHA class change (number and percent of participants) will be provided by treatment group at each visit for the randomization period to present.

2.13 Interim analysis

The safety interim analysis will be conducted where also data from the ongoing XXB750 phase 1b study CXXB750A12101 becomes available. This analysis will include all available data of

the study CXXB750A12101 and treatment arms 1-3 and 5 of the current study to assess the need for adapting the design of the current study.

An additional administrative interim analysis informing other programs may be conducted when approximately 430 participants are randomized into the study (approximately 40 participants each in arms 1-4) and are followed for 16 weeks to help the planning of another trial in a different development program. The results of this interim analysis will not lead to any changes in this study and will be kept blinded to anyone directly involved in the conduct of the study. More details about the DMC review process and how unblinding will be handled in the context of DMC reviews will be provided in the DMC charter.

No alpha adjustment will be made for the interim safety analysis, safety data monitoring, and the administrative interim analysis. All interim analyses and safety data monitoring will be performed by an independent statistician who will not be involved in the trial conduct. The results will be reviewed by the independent DMC.

The primary analysis may be performed after all participants have completed Week 16 (or discontinued prior to Week 16). A final analysis will be performed after all participants completed Week 24 (or discontinued prior to Week 24). Formal testing of the primary endpoint with full level alpha of 0.025 (1-sided) will be performed at the primary analysis time point.

Regular safety data monitoring, including the safety interim analysis and the administrative interim analysis, is planned as specified in Section 2.1 and study protocol Section 4.6.

3 Sample size calculation

The study is planned to randomize approximately 720 participants in total.

3.1 Primary endpoint (s)

For the primary endpoint analysis, 600 participants allocated in the ratio of 2:2:3:3 to placebo, XXB750 60 mg dose level, XXB750 120 mg dose level, and highest XXB750 dose level, respectively.

Assuming a common standard deviation of 0.67 for log NT-proBNP change from baseline and a one-sided 2.5% significance level (with adjustments for multiple comparisons using MCPMOD), a sample size of 600 participants (120 in placebo, 120 in XXB750 60 mg dose level, 180 in XXB750 120 mg dose level, and total of 180 in highest XXB750 dose level) will provide a power of 90% if the underlying true maximum NT-proBNP reduction on XXB750 vs. placebo is 23%.

3.2 Secondary endpoint(s)

A common standard deviation of 0.67 for log NT-proBNP change from baseline and a one-sided 2.5% significance level is assumed for all sample size determinations relating to secondary endpoints.

For the first secondary endpoint, a sample size of 120 participants in placebo and 180 participants in XXB750 optimal dose level, respectively, will provide a minimum power of 90% if the true NT-proBNP reduction on XXB750 vs. placebo is 23%.

For the second secondary endpoint, in participants with ACEI/ARB background medication converted to sacubitril/valsartan, a sample size of 120 participants in a group combining the two highest XXB750 target dose levels and 120 participants in sacubitril/valsartan conversion arm will provide approximately 80% power if the underlying true maximum NT-proBNP reduction with adding XXB750 vs. conversion to sacubitril/valsartan is 22%.

4 Change to protocol specified analyses

No change from protocol specified analysis was made.

5 Appendix

5.1 Imputation rules

5.1.1 Study drug

The missing or partially missing start or end dates will be handled/imputed using the Novartis ADaM Governance Board (AGB) global standard approach. Details will be provided in the study Programming Datasets Specifications (PDS) document.

5.1.2 AE date imputation

The missing or partially missing AE start or end dates will be handled/imputed using the Novartis AGB global standard approach. Details will be provided in the study PDS document.

5.1.3 Concomitant medication date imputation

The missing or partially missing concomitant medication start or end dates will be handled/imputed using the Novartis AGB global standard approach. Details will be provided in the study PDS document.

5.2 AEs coding/grading

Coding of AE will be done per MedDRA dictionary. The scale of severity grading described in section 8.6.1 of the study protocol will be used.

5.3 Laboratory parameters derivations

Details will be provided in the study PDS document.

5.4 Statistical models

5.4.1 Analysis supporting primary objective(s)

Refer to Section 2.5.

5.4.2 Analysis supporting secondary objective(s)

Refer to Section 2.6.

5.5 Rule of exclusion criteria of analysis sets

The following table presents a sample of the rules for participant classification in the analysis sets (Table 5-1).

Table 5-1 Criteria leading to exclusion

Analysis Set	Criteria that cause participants to be excluded
SCR	Not having informed consent;<br>Not having disposition page
RIS	Not in SCR;<br>Not having disposition page;<br>Not receiving single-blind run-in study medication
RAS	Not randomized
FAS	Not in RAS;<br>Mistakenly randomized and no double-blind study drug taken
SAF	Not in RAS;<br>No double-blind study drug taken
PKS	Not randomized;<br>Not receiving any study XXB750 treatment;<br>No available valid PK concenrtration measurement
IPS	Not in SAF;<br>Missing baseline ADA sample and no available post-baseline ADA sample
IIS	Not in SAF;<br>Missing baseline ADA sample or no available post-baseline ADA sample

5.6 KCCQ derivation of scores in each domain

The clinical summary score is a mean of the physical limitation and total symptom scores. The total symptom score is the mean of the symptom frequency and symptom burden scores. Each scale score (the physical limitation, symptom frequency or symptom burden) is calculated as the mean of its item scores and transformed to a 0–100 scale, with higher score indicating higher level of functioning. A score of 100 represents perfect health whereas a score of 0 represents dead. For patients who die, a worst score (score of 0) will be imputed for the clinical summary score at all subsequent scheduled visits after the date of death where the clinical summary score would have been assessed.

Below are the rules each scale score being calculated:

1. Physical Limitation

- Code responses to each of Questions 1a-f as follows:

Extremely limited = 1

Quite a bit limited = 2

Moderately limited = 3

Slightly limited = 4

Not at all limited = 5

- Limited for other reasons or did not do = <missing value>

If at least three of Questions 1a-f are not missing, then compute

- Physical Limitation Score = 100*[(mean of Questions 1a-f actually answered) – 1]/4

Note: references to "means of questions actually answered" imply the following. If there are n questions in a scale, and the subject must answer m to score the scale, but the subject answers only n-i, where n-i >= m, calculate the mean of those questions as

(sum of the responses to those n-i questions) / (n-i)

not

(sum of the responses to those n-i questions) / n

2. Symptom Frequency

- Code responses to Questions 3, 5, 7 and 9 as follows:

Question 3

Every morning = 1

3 or more times a week but not every day = 2

1-2 times a week = 3

Less than once a week = 4

Never over the past 2 weeks = 5

Questions 5 and 7

All of the time = 1

Several times a day = 2

At least once a day = 3

3 or more times a week but not every day = 4

1-2 times a week = 5

Less than once a week = 6

Never over the past 2 weeks = 7

Question 9

Every night = 1

3 or more times a week but not every day = 2

1-2 times a week = 3

Less than once a week = 4

Never over the past 2 weeks = 5

If at least two of Questions 3, 5, 7 and 9 are not missing, then compute:

S3 = [(Question 3) – 1]/4

S5 = [(Question 5) – 1]/6

S7 = [(Question 7) – 1]/6

S9 = [(Question 9) – 1]/4

Symptom frequency score = 100*(mean of S3, S5, S7 and S9)

3. Symptom burden
    - Code responses to each of Questions 4, 6 and 8 as follows:

Extremely bothersome = 1

Quite a bit bothersome = 2

Moderately bothersome = 3

Slightly bothersome = 4

Not at all bothersome = 5

I've had no swelling/fatigue/shortness of breath = 5

If at least one of Questions 4, 6 and 8 is not missing, then compute

Symptom burden score = 100*[(mean of Questions 4, 6 and 8 actually answered) – 1]/4

4. Total symptom score = mean of the following available summary scores:
    - Symptom frequency score
    - Symptom burden score
5. Clinical summary score = mean of the following available summary scores:
    - Physical limitation score
    - Total symptom score

6 Reference

Bretz, F., Maurer, W., Brannath, W. and Posch, M. (2009), A graphical approach to sequentially rejective multiple test procedures. Statist. Med., 28: 586-604.

Dong G, Qiu J, Wang D, et al (2018) The stratified win ratio. J Biopharm Stat; 28(4):778-96.

ICH E9(R1) Harmonized Guideline: addendum on estimands and sensitivity analysis in clinical trials to the guideline on statistical principles for clinical trials. Final version on 20 November 2019.

Pinheiro J, Bornkamp B, Bretz F (2006) Design and analysis of dose-finding studies combining multiple comparisons and modeling procedures. J Biopharm Stat; 16(5):639-56.

Pinheiro J, Bornkamp B, Glimm E, et al (2014) Model-based dose finding under model uncertainty using general parametric models. Stat Med; 33(10):1646-61.

Pocock SJ, Ariti CA, Collier TJ, et al (2012) The win ratio: a new approach to the analysis of composite endpoints in clinical trials based on clinical priorities. Eur Heart J; 33(2):176-82.

NOVARTIS

Clinical Development

XXB750

CXXB750A12201

A multi-center, randomized, placebo- and active-controlled, parallel-group, 24-week proof of concept and dose-finding study to evaluate efficacy, safety, and tolerability of XXB750 in patients with heart failure

Statistical Analysis Plan (SAP)

Document type:	SAP Documentation
Document status:	Amendment 1, Final
Release date:	04-Dec-2024
Number of pages:	35

Property of Novartis
Confidential
May not be used, divulged, published or otherwise disclosed
without the consent of Novartis

Template Version 6.0, Effective from 23-Nov-2022

Document History – Changes compared to previous final version of SAP

Date	Time point	Reason for update	Outcome for update	Section and title impacted (Current)
30-Nov-2023	Prior to FPFV	Creation of final version	N/A - First version	NA
04-Dec-2024	Prior to DBL	Study early termination	Analysis will be done descriptively. No inferential statistical testing or statistical modelling will be implemented.	All sections

Table of contents

Table of contents 3
List of tables 5
List of figures 5
List of abbreviations 6
1 Introduction 7
1.1 Study design 7
1.2 Study objectives, endpoints and estimands 8
1.2.1 Primary estimand(s) 10
1.2.2 Secondary estimand(s) 10
2 Statistical methods 11
2.1 Data analysis general information 11
2.1.1 General definitions 11
2.2 Analysis sets 13
2.2.1 Subgroup of interest 14
2.3 Patient disposition, demographics and other baseline characteristics 14
2.3.1 Patient disposition 14
2.3.2 Demographics and other baseline characteristics 15
2.3.3 Medical history 15
2.4 Treatments (study treatment, rescue medication, concomitant therapies, compliance) 16
2.4.1 Study treatment / compliance 16
2.4.2 Prior, concomitant and post therapies 17
2.5 Analysis supporting primary objective(s) 18
2.5.1 Primary endpoint(s) 19
2.5.2 Statistical hypothesis, model, and method of analysis 19
2.5.3 Handling of intercurrent events 19
2.5.4 Handling of missing values not related to intercurrent event 19
2.5.5 Multiplicity adjustment 19
2.5.6 Sensitivity analyses 19
2.5.7 Supplementary analyses 19
2.6 Analysis supporting secondary objectives 19
2.6.1 Secondary endpoint(s) 19
2.6.2 Statistical hypothesis, model, and method of analysis 20
2.6.3 Handling of intercurrent events 20
2.6.4 Handling of missing values not related to intercurrent event 20

2.6.5 Sensitivity analyses 20
2.6.6 Supplementary analyses 20
2.7 Safety analyses 20
2.7.1 Adverse events (AEs) 20
2.7.2 Adverse events of special interest and potential risks 21
2.7.3 Deaths 24
2.7.4 Laboratory data 24
2.7.5 Other safety data 27
2.8 Pharmacokinetic endpoints 28
2.9 PD and PK/PD analyses 28
2.10 Patient-reported outcomes 28
2.11 Biomarkers 28
2.12 Other Exploratory analyses 28
2.12.1 Exploratory variables 28
2.12.2 Analysis methods 29
2.13 Interim analysis 29
3 Sample size calculation 29
3.1 Primary endpoint (s) 29
3.2 Secondary endpoint(s) 30
4 Change to protocol specified analyses 30
5 Appendix 30
5.1 Imputation rules 30
5.1.1 Study drug 30
5.1.2 AE date imputation 30
5.1.3 Concomitant medication date imputation 30
5.2 AEs coding/grading 30
5.3 Laboratory parameters derivations 31
5.4 Statistical models 31
5.4.1 Analysis supporting primary objective(s) 31
5.4.2 Analysis supporting secondary objective(s) 31
5.5 Rule of exclusion criteria of analysis sets 31
5.6 KCCQ derivation of scores in each domain 32
6 Reference 35

List of tables

Table 1-1 Original objectives and related endpoints 8
Table 2-1 Specification of subgroups 14
Table 2-2 Study treatment dose levels during randomized treatment epoch 16
Table 2-3 AESIs and potential risks with additional analysis 22
Table 2-4 Clinically notable laboratory values and vital signs 24
Table 5-1 Criteria leading to exclusion 31

List of figures

Figure 1-1 Study design 8

List of abbreviations

AE	Adverse Event
CRF	Case Report Form
CSR	Clinical Study Report
DMC	Data Monitoring Committee
DMS	Document Management System
FAS	Full Analysis Set
IA	Interim Analyses
MedDRA	Medical Dictionary for Drug Regulatory Affairs
PK	Pharmacokinetics
PPS	Per-Protocol Set
PRO	Patient-reported Outcomes
RAP	Reporting & Analysis Process
SAP	Statistical Analysis Plan
SAS	Statistical Analysis System
TFLs	Tables, Figures, Listings
WHO	World Health Organization

1 Introduction

The statistical analysis plan (SAP) describes the detailed methodology and implementation of the planned statistical analyses outlined in the study protocol for CXXB750A12201. The analyses following the SAP below will be used for clinical study reporting purposes.

This version of SAP details the statistical methodology for the analyses planned and agreed to at the time of finalization of the CXXB750A12201 (Version 02 – amended protocol dated 23-Feb-2024).

As per the urgent safety communication (9-Aug-2024) followed by Novartis decision dated 26-Sep-2024, study drug was suspended on 9-Aug-2024 and a decision to terminate the study was taken on 26-Sep-2024. Hence, only an abbreviated clinical study report (CSR) will be created for this study.

1.1 Study design

Study CXXB750A12201 is a multicenter, randomized, placebo- and active-controlled, parallel group phase 2 study which is comprised of three periods (Figure 1-1):

- A screening period of approximately 7 days;
- A 16-week parallel-group randomized treatment period on either
  - i) double-blind placebo-controlled treatment (XXB750 vs. placebo) or,
  - ii) open-label treatment (sacubitril/valsartan);
- An 8-week safety follow-up period.

The study will randomize adult participants with LVEF < 50% receiving ACEI/ARB/ARNI and guideline-recommended HF therapies for HFrEF or HFmrEF to three XXB750 target dose levels; a cohort of patients treated with ACEI/ARB before the study will be randomized to be converted to open-label sacubitril/valsartan in place of their pre-study ACEI/ARB. The study will randomize a total of approximately 720 patients. The percentage of patients with LVEF > 40% will be limited to approximately 25% of the total sample.

Refer to study protocol Section 6.1.2 for details on the administered doses in the different treatment arms.

A staggered approach to enrollment will be followed in the study protocol. The first group of 300 participants will be randomized to placebo (treatment arm 1), XXB750 60 mg target dose (treatment arm 2), XXB750 120 mg target dose (treatment arm 3), and conversion to sacubitril/valsartan (treatment arm 5). The independent Data Monitoring Committee (DMC) will complete a safety analysis of the XXB750 doses tested and assess if it is safe to allow the dosing of patients with XXB750 planned top dose of 240 mg (i.e., allowing enrollment in arms 4a and 4b as planned). See study protocol Section 4.6 for details on planned interim analyses and adaptive design features.

However, due to safety concerns, an urgent safety communication (9-Aug-2024) was issued to suspend study drug and a decision to terminate the study was taken on 26-Sep-2024. Only first group of participants are available for analysis, including placebo (treatment arm 1), XXB750

60 mg target dose (treatment arm 2), XXB750 120 mg target dose (treatment arm 3), and conversion to sacubitril/valsartan (treatment arm 5).

Figure 1-1 Study design

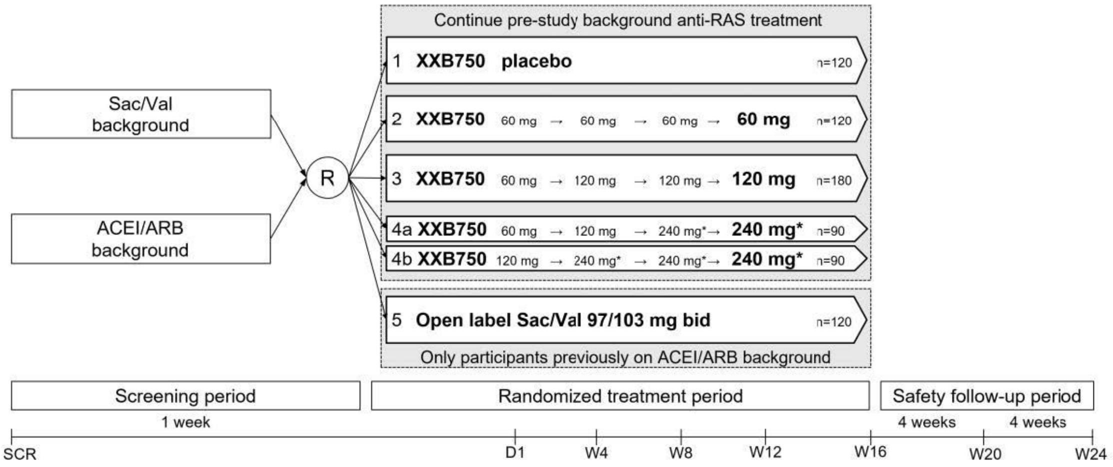

Sac/Val: Open label sacubitril/valsartan. *May be modified per adaptive design feature of the study (study protocol Section 4.6). Doses of Sac/Val, ACEI or ARB in arms 1-4 equal to dose at randomization.

The primary and final analysis will be performed after all participants have completed their final study visits.

1.2 Study objectives, endpoints and estimands

As per the urgent safety communication notification dated 9-Aug-2024 and Novartis decision dated 26-Sep-2024, the original objectives are no longer applicable due to permanent discontinuation of study treatment and early study termination. No statistical modelling will be done, and no formal statistical hypothesis tests will be performed. Table 1-1 indicates the original planned objectives, endpoints and which of those will be summarized descriptively for the CSR.

Table 1-1 Original objectives and related endpoints

Original objective(s)	Original endpoint(s)	Will be reported in CSR
**Primary objective(s)**	**Endpoint(s) for primary objective(s)**	
• To evaluate the efficacy and dose-response relationship of three XXB750 target dose levels compared to placebo in reducing NT-proBNP from baseline to Week 16 in symptomatic HF patients with LVEF < 50% treated with standard of care, including ACEI/ARB or sacubitril/valsartan.	• Change in log NT-proBNP from baseline to Week 16	• NT-proBNP

Original objective(s)	Original endpoint(s)	Will be reported in CSR
**Secondary objective(s)**	**Endpoint(s) for secondary objective(s)**	
• To evaluate the treatment effect of the highest XXB750 target dose level compared to placebo in reducing NT-proBNP from baseline to Week 16 in symptomatic HF patients with LVEF < 50% treated with standard of care, including ACEI/ARB or sacubitril/valsartan.	• Change in log NT-proBNP from baseline to Week 16	• NT-proBNP
• To evaluate the treatment effect of combined two highest XXB750 target dose levels administered in addition to a background of ACEI/ARB versus conversion from ACEI/ARB to sacubitril/valsartan, in reducing NT-proBNP from baseline to Week 16.	• Change in log NT-proBNP from baseline to Week 16	• NT-proBNP
• To evaluate the safety and tolerability of XXB750 up-titration regimens and dose levels.	• Adverse events (incidence and severity, including but not limited to, hypotension, tachycardia, bradycardia, hypersensitivity and injection site reactions), vital signs (blood pressure, pulse), safety laboratory tests, and ECG parameters from baseline to end of study (EOS).	• Adverse events (incidence and severity, including but not limited to, hypotension, tachycardia, bradycardia, hypersensitivity and injection site reactions), vital signs (blood pressure, pulse), safety laboratory tests, and ECG parameters
**Exploratory objective(s)**	**Endpoint(s) for exploratory objective(s)**	
• To evaluate the effects of highest XXB750 target dose level compared to placebo in improving a composite hierarchical outcome of : 1) time to all-cause death, 2) number of HF hospitalizations, 3) Kansas City Cardiomyopathy Questionnaire (KCCQ) clinical summary score (CSS) categorical change, 4) NYHA class change , 5) relative change in NT-proBNP levels between baseline and Week 16.	• Hierarchically ordered composite consisting of: 1) time to all-cause death up to Week 16; 2) number of HF hospitalizations up to Week 16; 3) categorical KCCQ CSS change from baseline to Week 16; 4) NYHA class change from baseline to Week 16; 5) relative change in NT-	•

Original objective(s)	Original endpoint(s)	Will be reported in CSR
	proBNP levels from baseline to Week 16.	
• To evaluate changes in each domain of the KCCQ, including clinical summary score (CSS) and overall summary score (OSS), from baseline to Week 16.	• Change in all domains of the KCCQ from baseline to Week 16.	• KCCQ clinical summary score (CSS) and overall summary score (OSS)
• To evaluate the effects of XXB750 on slowing the rate of decline in estimated glomerular filtration rate (eGFR) from baseline to Week 16.	• Estimated glomerular filtration rate (eGFR) at scheduled assessment visits.	• Estimated glomerular filtration rate (eGFR)
• To evaluate the change in biomarkers related to heart failure, cardiovascular disease and renal function from baseline to Week 8 and to Week 16.	• Change from baseline in biomarkers including, but not limited to, plasma and urine cGMP, urinary cGMP-to-creatinine ratio, urinary albumin-to-creatinine ratio, BNP and hs-Troponin, at Week 8 and Week 16.	• Biomarkers including, but not limited to, plasma and urine cGMP, urinary cGMP-to-creatinine ratio, urinary albumin-to-creatinine ratio, BNP and hs-Troponin
• To evaluate the change in NYHA class and in signs and symptoms of heart failure from baseline to Week 16.	• Change in NYHA class and signs and symptoms of HF from baseline to Week 16.	• NYHA class and signs and symptoms of HF
• To explore the population pharmacokinetic (PK) properties of XXB750 in HF patients.	• Population PK analysis results including mathematic models and parameters.	• Population PK. See Section 2.8 for details.
• To evaluate immunogenicity (IG) of XXB750.	• Detection of immune response markers, including anti-drug antibodies.	• Immune response markers, including anti-drug antibodies. See Section 2.7.5.3 for details.

1.2.1 Primary estimand(s)

Not applicable due to early termination.

1.2.2 Secondary estimand(s)

Not applicable to secondary estimands due to early termination.

Adverse events (AEs) and serious AEs, laboratory parameters and vital signs will be evaluated and summarized in each treatment group for randomization period. Please refer to Section 2.7 for analysis details.

2 Statistical methods

The following sections contain important information on detailed statistical methodology used for clinical study report (CSR) analysis and reporting purposes.

Due to early study termination, the original planned analysis of primary, secondary and exploratory endpoints to demonstrate efficacy and dose range finding are no longer applicable.

2.1 Data analysis general information

Data will be analyzed by Novartis using SAS 9.4, unless otherwise specified. Details on planned statistical analyses and data-driven regression diagnostics (if any) will be presented in the following section and in CSR Appendix 16.1.9.

In general, the continuous variables will be summarized descriptively by presenting n, mean, SD, median, quartiles, minimum and maximum while categorical variables will be summarized by presenting count and percentage of participants in each category.

The randomization in this study will be stratified by region and background medication (ACEI/ARB or sacubitril/valsartan).

Regular safety monitoring (approximately every six months unless otherwise requested by DMC) will be conducted and reviewed by an external DMC as specified in the DMC charter. The relevant DMC analyses will be described in a separate DMC analysis plan and will be executed by an external independent statistician and independent programmers privileged to assess unblinded clinical trial data.

2.1.1 General definitions

Study treatment or drug

In future sections through this document, ‘study treatment’ or ‘study drug’ will be used to refer to investigational treatment assigned to a participant. Specifically, for the randomized treatment period, study treatment refers to XXB750 doses, XXB750 placebo, and sacubitril/vasartan as assigned to a participant at randomization.

Due to early termination, only one group of participants initiated, and all analysis will be performed by treatment group within group 1 (i.e., XXB750 placebo, XXB750 60 mg, XXB750 120 mg, and conversion to sacubitril/valsartan).

Date of first or last administration of study drug/treatment

Date of first administration of study drug/treatment refers to the date when the first dose of assigned treatment is administered in randomized treatment period. Date of last administration of study drug/treatment refers to the date when the last dose of assigned treatment is administered in randomized treatment period. For participants who are randomized to XXB750 dose arms or placebo arm, end of treatment (EOT) is at Visit 9998 (Week 16) for participants who complete randomized treatment period; and for participants who prematurely discontinue randomized treatment is defined as max(last administration of double-blind study drug + 28 days, treatment disposition date), date of withdrawal of informed consent, or the date of death,

whichever occurres first. For participants who are randomized to open-label sacubitril/valsartan arm, EOT is defined as the last administration of study drug, date of withdrawal of informed consent, or the date of death, whichever occurrs first.

Screening period

Screening period begins at Visit 10 until the day before the start of randomized treatment period (Visit 101) or screening disposition date for those who do not qualify to continue.

Randomized treatment period

The randomized treatment period begins at the time of randomization and ends with the EOT disposition. During the randomized treatment period, participants will return for scheduled clinic visits. For all safety related analyses randomized treatment starts with the first administration of randomized study drug.

Safety follow-up period

The safety follow-up period begins one day after EOT visit date and ends on the date of EOS.

Randomization period

The randomization period is the combination of the randomized treatment period and the safety follow-up period, unless otherwise specified.

Study exposure

The study exposure means the exposure during the randomization period. The duration of study exposure (days) is defined as the date of end of study minus the date of randomization plus one.

Baseline for randomized treatment period

Only assessments performed prior to first dose of randomized treatment period are considered for baseline. Unless specified otherwise, baseline for the randomized treatment period is defined as the measurement obtained at the randomization visit (Visit 101), or the measurement obtained at an earlier visit (scheduled or unscheduled) which was closest to the Visit 101, if Visit 101 measurement is missing.

Baseline for randomization period

Randomization period is the total duration of randomized treatment period and the safety follow-up period, therefore, the definition of baseline for randomization period will be the same as the baseline for randomized treatment period.

Baseline for safety follow-up period

Unless otherwise specified, baseline for safety follow-up period is defined as the measurement obtained at the EOT visit (Visit 9998) or the measurement obtained closest to the EOT visit if the measurement at EOT is missing.

Unscheduled visit

Only for the analysis of safety evaluation will unscheduled measurements be taken into account. For efficacy evaluations, measurements from unscheduled visits will generally not be used, unless otherwise specified.

On-treatment data for an efficacy endpoint

In all the analyses planned in this document, on-treatment data refer to data collected while participants are on-study-medication (regardless of treatment interruption) or within 28 days after final study drug administration date for participants randomized to XXB750 and placebo arms; and final study drug amininstration date for particpants randomization to open-label sacubitril/valsartan.

Study day

Study day of any assessment is defined as the date of assessment (event/visit) minus the date of randomization plus one unless specified otherwise.

2.2 Analysis sets

The following analysis sets will be defined for statistical analysis:

Screened Set (SCR) - All participants who signed the informed consent form. The SCR includes only unique screened participants, i.e., in the re-screened participants only the chronologically last screening data is counted.

Randomized Analysis Set (RAS) - All randomized participants who received a randomization number, regardless of receiving trial medication.

Full Analysis Set (FAS) - All participants to whom study treatment has been assigned by randomization and who are not mis-randomized. Mis-randomized participants are those who have not been qualified for randomization, have been inadvertently randomized into the study and did not receive any study medication. According to the intent to treat principle, participants will be analyzed according to the treatment they have been assigned to during the randomization procedure.

Safety Set (SAF) - All randomized participants who received at least one dose of study treatment. Participants will be analyzed according to the study treatment received, where treatment received is defined as the randomized/assigned treatment if the participant took at least one dose/injection of that treatment or the first treatment received if the randomized/assigned treatment was never received.

Per-Protocol Set (PPS) – A subset of the FAS that consisted of 1) participants randomized to XXB750 dose arms or placebo arm who received at least 3 doses/injections as per schedule; and 2) participants randomized to open-label sacubitril/valsartan arm who have been followed for at least 8 weeks post randomization as per schedule. This supplemental efficacy set is to support the efficacy analysis results on key biomarkers of interest.

PK analysis set (PKS) - All randomized participants with at least one available valid (i.e., not flagged for exclusion) PK concentration measurement, who received any study XXB750 treatment.

Immunogenicity prevalence set (IPS) includes all participants in the Safety set with a non-missing baseline ADA sample **or** at least one non-missing post-baseline ADA sample.

Immunogenicity incidence set (IIS) includes all participants in the Safety set with a non-missing baseline ADA sample **and** at least one non-missing post-baseline ADA sample.

Rules leading to exclusion from specific analysis sets of participants are provided in Appendix 5.5.

2.2.1 Subgroup of interest

Subgroups will be formed to explore the consistency of efficacy and safety profiling on selected endpoints between the subgroups and the overall population.

In general, subgroups will be defined based on baseline information mentioned in Section 2.1.1.

Subgroups defined for selected endpoints in this study and the ways to derive them are listed in Table 2-1. The details about the subgroup analyses will be described in the corresponding sections as appropriate.

Table 2-1 Specification of subgroups

Subgroup	Method of derivation	Background & Demographics / Exposure	Efficacy*	Safety
LVEF (≤40% vs. >40%)	Screening	X	X**	X
Type of RAS inhibition (ACEI/ARB, sacubitril/valsartan)	Screening	X	X	X
ADA subgroup*** (Positive, Negative, PBL, Missing)	Derived			X

* Efficacy refers to descriptive analysis for key efficacy biomarkers of interest.

** LVEF (≤40% vs. >40%) subgroup analysis will be performed only for primary endpoint.

*** Defined as positive if ADA screening test at randomization baseline (pre-dose) is negative and the test turns positive at any time post-dose during the study, negative if ADA screening test shows negative at all times during the study, PBL if positive at baseline (pre-dose), and missing otherwise.

2.3 Patient disposition, demographics and other baseline characteristics

2.3.1 Patient disposition

The number and percentage of participants screened successfully will be presented. In addition, screen failure participants will be summarized by primary reason for screen failure. For participants who are screened more than once, the information from the last screen will be used in the summary. The analysis is based on the screened set (SCR).

The number and percentage of randomized participants included in different analysis sets (Section 2.2) will be summarized by treatment group. The number and percentage as well as the reasons that participants had been excluded from RAS will be summarized by treatment. The number and percentage of randomized participants (RAS) and those in the full analysis set (FAS) who completed the study, who discontinued the study, and the reasons for discontinuation will be presented by treatment group, respectively.

In addition, the number and percentage of participants with protocol deviations as well as the criteria leading to exclusion from analysis sets will be provided for the participants in randomized set (RAS). Furthermore, the number and percentage of participants randomized per region and per country will be presented for the SCR and the FAS, respectively. All disposition data will also be listed at participant level for randomization period disposition.

2.3.2 Demographics and other baseline characteristics

Summary statistics will be provided for the total number of participants pertaining to the FAS for background and demographic characteristics, disease characteristics, and cardiovascular risk factors for the randomized treatment baseline. The following parameters will be included if applicable:

- **Continuous variables**: Age (in years), weight (in kilogram), height (in centimeter), body mass index (BMI, in kg/m^2), LVEF (%), sitting office systolic blood pressure (in mmHg), sitting office diastolic blood pressure (in mmHg), sitting pulse (in bpm), cyclic GMP (plasma and urine), urinary cGMP-to-creatinine ratio, urinary albumin-to-creatinine ratio, urine creatinine, cyclic GMP (urine)/urine creatinine, NT-proBNP, dose of standard background therapy of ACEI/ARB or sacubitril/valsartan, Kansas City Cardiomyopathy Questionnaire (KCCQ) clinical summary score (CSS) and overall summary score (OSS), and eGFR (ml/min/1.73m^2).
  - **Categorical variables**: Age group (<65 years vs. ≥65 years), sex, race, region, history of diabetes (Yes, No), history of MI (Yes, No), history of hypertension (Yes, No), history of atrial fibrillation/flutter (Yes, No), smoking history (Yes, No), alcohol use history (0, >0 to 3, >3 drinks consumed per day in average), prior history of coronary heart disease (Yes, No), LVEF group (≤40% vs. >40%), NYHA class, etiology of HF (ischemic vs. non-ischemic), category of prior CHF medication, eGFR (< 60 vs. ≥ 60 ml/min/1.73m^2).

BMI will be calculated as weight (kg) / height^2 (m^2) from the measured height and weight at Visit 10 (Screening Visit).

The summary will be presented for FAS.

2.3.3 Medical history

Any condition entered on the relevant medical history / current medical conditions CRF will be coded using the most updated version of MedDRA dictionary at the time of clinical database lock. Medical history will be collected at Visit 10 (Screening Visit). The number and percentage of participants with each medical condition will be provided by treatment group and system of organ class for the FAS.

2.4 Treatments (study treatment, rescue medication, concomitant therapies, compliance)

2.4.1 Study treatment / compliance

Due to early termination, only group 1 was initiated and participants are randomized into one of the following arms targeting a ratio of 1:1:2:1.

- Placebo matching XXB750
- XXB750 60 mg target dose
- XXB750 120 mg target dose
- Sacubitril/valsartan at target doses of 97/103 mg bid (only participants previously treated with ACEI/ARB)

Table 2-2 Study treatment dose levels during randomized treatment epoch

Dose Level	XXB750 arms	Sacubitril/valsartan
0	Placebo or interruption	Interruption
1	XXB750 60 mg	50 (24/26) mg bid
2	XXB750 120 mg	100 (or 49/51) mg bid
3		200 (or 97/103) mg bid

Treatment exposure during the randomized treatment period

The duration of the randomized treatment period exposure for a participant, is defined as follows:

- For participants randomized to XXB750 dose arms or placebo arm:
    - If the participant completes the randomized treatment period, it is defined as the Week 16 (EOT) visit date – first study drug administration date +1;
    - If the participant prematurely discontinues the study treatment, it is defined as min(max(date of last study drug administration + 28 days, treatment disposition date), date of death, date of informed consent withdrawal) – first study drug administration date + 1.
- For a participant randomized to open-label sacubitril/valsartan: min(date of last study drug administration, date of death, date of informed consent withdrawal) – first study drug administration date + 1.

Duration of overall randomized treatment exposure, including temporary interruptions, will be summarized descriptively by treatment group (i.e. n, mean, standard deviation, min, Q1, median, Q3, max) and the number (percentage) of participants within the following duration categories will also be provided:

- $<$ 4 weeks
- $\geq$ 4 weeks to $<$ 8 weeks
- $\geq$ 8 weeks to $<$ 12 weeks
- $\geq$ 12 weeks

Overall patient-weeks on-treatment and average patient-weeks on-treatment will be reported by each treatment group.

- Overall patient-weeks on-treatment = Sum of duration of treatment exposure (in days) from all participants/ 7
- Average patient-weeks on-treatment = Overall patient-weeks on-treatment / Number of participants randomized to the treatment

Number and percentage of participants at each dose level defined in Table 2-2 will also be summarized by the number of study treatment administrations, by visit and treatment.

Average dose will be summarized by treatment group during randomized treatment period and calculated as below:

$$\frac{\sum_{i=0}^{3}(\text{no. of days on dose level i}) \times (\text{dose level i*})}{\sum_{i=0}^{3}(\text{no. of days on dose level i})},$$

where dose level is the dose administered at a visit and number of days will be from the date of injection until the day before next dose administered for XXB750 and placebo treatment groups; and dose level is the total daily dose for open-label sacubitril/valsartan arm.

Dose administration records for randomized treatment period will also be listed at participant level for the participants in safety set.

Number and percentage of participants with permanent treatment discontinuations will be provided by reason for discontinuation.

All these analyses pertaining to treatment exposure during randomized treatment period will be carried out for all participants in Safety set (SAF).

2.4.2 Prior, concomitant and post therapies

Medications will be identified using Novartis Drug and Therapy Dictionary, NovDTD which is a modified Novartis internal version of the WHO Drug Dictionary Enhanced (DDE) including the Anatomical Therapeutic Chemical (ATC) code. The latest version at the time of clinical database lock will be used.

Prior medications are defined as drugs taken prior to the study medication. **Randomized treatment concomitant medications** are medications taken at any time during the randomized treatment period and prior to safety follow-up. **Safety follow-up concomitant medications** are those given at least once during safety follow-up period. Prior, randomized treatment period or safety follow-up concomitant medication will be identified based on recorded or imputed start and end dates of taking medication. The rules for imputing incomplete start and end dates are described in Section 5.1.

The concomitant medication information for the randomization period will be summarized based on the SAF. The prior concomitant medications will be summarized based on SCR and SAF, separately. Specifically for concomitant medication summary, the start of randomization period, the start of the period is defined as the first study drug administration date for XXB750 treatment or placebo arm; for open-label Sac/Val arm, the start of the period is defined as 1 day after the first study drug administration date.

Prior and Concomitant medications will be summarized by treatment group. Medications will be presented in alphabetical order by ATC codes and grouped by chemical subgroup (the 4th level of the ATC codes). Tables will also show the overall number and percentage of participants receiving at least one medication of a particular ATC code and at least one drug in a particular chemical subgroup.

The number and percentage of participants on the following classes of background medications during the study will be tabulated by treatment group for FAS and for SAF.

- Aldosterone antagonists
- SGLT-2 inhibitors
- Antiarrhythmics, Class I and III
- Oral anticoagulants
- Antiplatelet agents (including aspirin)
- Beta blockers
- Digitalis glycosides
- Calcium antagonists
- Loop and thiazide or thiazide-like diuretics
- Statins

The search criteria for the classes of medications listed above will be defined in a separate EXCEL sheet with ATC preferred term and WHO drug codes. The EXCEL sheet will be stored in document management/archival system at the RAP level after the content is agreed upon by the Global Program Medical Director or Clinical Head (GPMD/GPCH) and before clinical database lock (CDBL).

Standard background HF medication

Standard background HF medications (or auxiliary medicinal products under EU CTR) in this study are ACEI, ARB and sacubitril/valsartan. The mean daily dose for the background HF medication will be calculated as:

Mean daily dose = [(No. of days on dose level 1)* level 1 daily dose + (No. of days on dose level 2)*level 2 daily dose + … + (No. of days on dose level K)*level K daily dose]/(No. of days in respective period of interest)

Please refer to Section 2.1.1 for the definition of each period. However, for background HF medication summray, the start of randomization period is defined as the first study drug administration date for XXB750 treatment or placebo arm; for open-label Sac/Val arm, the start of the period is defined as 1 day after the first study drug administration date. The summary for standard background HF medications will be presented for randomization period using FAS. For each medication class, the summary table will present the most frequent drug names of interest in the FAS set.

2.5 Analysis supporting primary objective(s)

The primary and final analysis will be performed after all participants have completed their final visits. No formal statistical hypothesis testing nor statistical modelling will be performed.

2.5.1 Primary endpoint(s)

The primary efficacy variable is change in NT-proBNP during randomization period.

2.5.2 Statistical hypothesis, model, and method of analysis

Summaries of absolute values and change from baseline in NT-proBNP by treatment group and visit will be presented using statistics as described in Section 2.1 additionally with geometric mean and corresponding 95% CI. Figures will be produced to visually show the observed mean NT-proBNP level by visit over randomization period for each treatment group.

The FAS and PPS will be used for the above analyses.

2.5.3 Handling of intercurrent events

Not applicable.

2.5.4 Handling of missing values not related to intercurrent event

The missing values will be treated as missing. Unless otherwise stated, missing data will not be imputed for the analysis.

2.5.5 Multiplicity adjustment

Not applicable as no formal statistical hypothese test is planned for primary or secondary objectives.

2.5.6 Sensitivity analyses

No sensitivity analysis will be performed.

2.5.7 Supplementary analyses

Subgroup analyses will be performed for the primary analysis. The analysis will be done using the analysis specified in Section 2.5.2 at individual subgroup level separately. Please see Section 2.2.1 for subgroup details.

2.6 Analysis supporting secondary objectives

2.6.1 Secondary endpoint(s)

Due to early study termination, the original planned analysis of secondary endpoints to demonstrate efficacy is no longer applicable.

Only group 1 was initiated, which include placebo (treatment arm 1), XXB750 60 mg target dose (treatment arm 2), XXB750 120 mg target dose (treatment arm 3), and conversion to sacubitril/valsartan (treatment arm 5). In addition, as specified in Section 2.5.7, subgroup analysis will provide summary statistics for NT-proBNP during randomization period by treatment group for each type of RAS inhibition (ACEI/ARB, sacubitril/valsartan) at screening.

2.6.2 Statistical hypothesis, model, and method of analysis

Not applicable due to early termination.

2.6.3 Handling of intercurrent events

Not applicable.

2.6.4 Handling of missing values not related to intercurrent event

Not applicable.

2.6.5 Sensitivity analyses

Not applicable.

2.6.6 Supplementary analyses

Not applicable.

2.7 Safety analyses

For all safety analyses, the SAF set will be used. All listings, figures and tables will be presented by treatment group.

Safety data to be analyzed are listed as below:

- Adverse events (AE) and Serious Adverse events (SAE)
- Safety topics that are adverse events of special interest (AESI): Serious hypotension, Serious hypersensitivity reactions, Serious injection site reactions, Severe tachycardia, Severe acute bradycardia, and Serious presyncope/syncope
- Safety topics that are potential risks: Hypotension, Hypersensitivity reactions, Injection site reactions, Tachycardia, Bradycardia, Immunogenicity
- Safety topics that are neither AESI nor Potential risks: Renal toxicity and Hepatotoxicity
- Laboratory assessments
- Vital signs, e.g., weight, systolic blood pressure (SBP), diastolic blood pressure (DBP), and pulse rate.

2.7.1 Adverse events (AEs)

Any AE that occurred during the study period will be included in AE summary tables during the randomization period, i.e., consisting of randomized treatment period and safety follow-up period.The analysis will consider all participants in the Safety set (SAF) for reported AEs during the randomization period.

Treatment emergent adverse events (new or worsened) during randomization period are defined as any recorded AE with its start date (recorded or imputed) later than or equal to the start date of the randomization period as defined below:

1) Randomization period: first date of study treatment injection.

The incidence of treatment-emergent adverse events (new or worsened) will be summarized by primary system organ class, preferred term, severity, and relationship to study drug according to the Medical Dictionary for Regulatory Activities (MedDRA). The latest MedDRA version before clinical database lock will be used for reporting the study.

If a participant reported more than one adverse event with the same preferred term, the adverse event with the greatest severity will be presented. If a participant reported more than one adverse event within the same primary system organ class, the participant will be counted only once with the greatest severity at the system organ class level, where applicable. Statistical analyses, as appropriate, will include all AEs with onset date during the randomization period and up to the analysis cut-off irrespective of how long after the last day of study treatment they occurred.

The number and percentage of participants reporting any adverse event during each reporting period will be summarized by primary system organ class, preferred term and treatment. The most common adverse events reported (≥ 5% in any group for each preferred term in the SOC-PT table) will be presented in descending frequency according to its incidence in the XXB750 highest dose group starting from the most common event.

Summaries for randomization period will be provided for study medication related adverse events, deaths, serious adverse events, other significant adverse events leading to permanent study discontinuation.

AESI and potential risks will be summarized in addition to the above analysis. Please see Section 2.7.2 for details.

2.7.2 Adverse events of special interest and potential risks

The following specific safety topics will be summarized separately in addition to the above analysis:

- AESI
    - Serious hypotension
    - Serious hypersensitivity reactions
    - Serious injection site reactions
    - Severe tachycardia
    - Severe acute bradycardia
    - Serious presyncope/syncope
- Potential risks:
    - Hypotension
    - Tachycardia
    - Bradycardia
    - Hypersensitivity reactions
    - Injection site reactions

    - Immunogenicity
- Other safety topics
    - Renal toxicity
    - Hepatotoxicity

The sets of terms, pertaning either to MedDRA or to NMQ or to CMQ, that are used to search the database to retrieve cases of interest that relate to the safety topics listed above are stored (or alternatively "summarized") in the Case Retrieval Strategy (eCRS). The latest version of the eCRS at the time of clinical database lock will be used.

The following standard analyses will be provided for all the listed safety topics (excluding Immunogenicity):

- Incidence (absolute and relative frequency) rates in terms of participant regardless of causal relationship to study drug for randomization period, by treatment group.
- Exposure-adjusted incidence rates per 100 patient years regardless of study drug relationship for randomization period, by treatment
- Analysis for time-to-first selected safety topic of interest by treatment group will be performed for randomization period using Kaplan-Meier estimates
- Listing of AEs by participant with AESIs and risk information.

In addition to the above, the analysis specified below will be provided for the safety topics that are listed in Table 2-3.

In the urgent safety communication (9-Aug-2024), it mentions that “the DMC identified an imbalance in the frequency of worsening heart failure events in patients receiving XXB750 in this study”. Thus, a further safety topic “Cardiac Failure” will be added in analysis, which is defined as MedDRA SMQ Cardiac Failure (broad).

Table 2-3 AESIs and potential risks with additional analysis

Safety topic	Definitions	Analysis and additional criteria for characterizing selected AEs
Hypotension	Hypotension [STANDARD] (NMQ)	Distribution of TEAE by SOC and PT<br>Distribution of TEAE by pre-defined subgroups (as per Table 2-1)
Tachycardia	ADR Tachycardia [ADR_STD] (NMQ)	Distribution of TEAE by SOC and PT<br>Distribution of TEAE by pre-defined subgroups (as per Table 2-1)
Bradycardia	ADR Heart rate decreased [ADR_STD] (NMQ)	Distribution of TEAE by SOC and PT

Safety topic	Definitions	Analysis and additional criteria for characterizing selected AEs
		Distribution of TEAE by pre-defined subgroups (as per Table 2-1)
Injection site reactions	ADR Injection site reaction [ADR_STD] (NMQ)	Distribution of TEAE by SOC and PT<br>Distribution of TEAE by pre-defined subgroups (as per Table 2-1)
Hypersensitivity reactions	(Anaphylactic/anaphylactoid shock conditions (SMQ) OR Anaphylactic reaction (SMQ) OR Hypersensitivity other than administration site reactions [XXB750] (NMQ))	Distribution of TEAE by SOC and PT<br>Distribution of TEAE by pre-defined subgroups (as per Table 2-1)
Immunogenicity	All serious and non-serious TEAE in participants with at least one positive ADA test ever	Distribution of TEAE by ADA subgroup (as per Table 2-1)
Serious hypotension	Only SAE: ADR Hypotension [ADR_STD] (NMQ)	Distribution of TEAE by SOC and PT<br>Distribution of TEAE by pre-defined subgroups (as per Table 2-1)
Serious hypersensitivity reactions	Only SAE: (Anaphylactic/anaphylactoid shock conditions (SMQ) OR Anaphylactic reaction (SMQ) OR Hypersensitivity other than administration site reactions [XXB750] (NMQ))	Distribution of TEAE by SOC and PT<br>Distribution of TEAE by pre-defined subgroups (as per Table 2-1)
Serious injection site reactions	Only SAE: ADR Injection site reaction [ADR_STD] (NMQ)	Distribution of TEAE by SOC and PT<br>Distribution of TEAE by pre-defined subgroups (as per Table 2-1)
Severe tachycardia	Only severe AE: ADR Tachycardia [ADR_STD] (NMQ)	Distribution of TEAE by SOC and PT<br>Distribution of TEAE by pre-defined subgroups (as per Table 2-1)
Severe acute bradycardia	Only severe AE: ADR Heart rate decreased [ADR_STD] (NMQ)	Distribution of TEAE by SOC and PT<br>Distribution of TEAE by pre-defined subgroups (as per Table 2-1)

Safety topic	Definitions	Analysis and additional criteria for characterizing selected AEs
Serious presyncope/syncope	Only SAE: ADR Presyncope [ADR_STD] (NMQ) OR ADR Depressed level of consciousness [ADR_STD] (NMQ) OR ADR Loss of consciousness [ADR_STD] (NMQ)	Distribution of TEAE by SOC and PT<br>Distribution of TEAE by pre-defined subgroups (as per Table 2-1)

2.7.3 Deaths

Participants that died during the study randomization period will be reported. Deaths will be summarized by actually received treatment group to present number and percentage of participants that died. In addition, listings will be provided for participants that died.

The analysis will consider all participants in the Safety set (SAF) for reported deaths during the randomization period.

2.7.4 Laboratory data

For each laboratory parameter, evaluations will be summarized by visit and actually received treatment group by presenting summaries (n, mean, standard deviation, median, minimum and maximum) for actual and change from baseline values for randomization period. The summary will be provided separately for biochemistry and hematology laboratory parameters. Central laboratory data will be used for the summaries.

Shift tables based on the standard ranges for each laboratory parameter (biochemistry and hematology) will be provided by treatment group at each visit for the randomization period to present incidence of transitions from a baseline high, normal or low laboratory value to a maximum post-baseline high, normal or low value. In addition to these analyses, laboratory parameter values will be listed for each participant by scheduled assessment visit and treatment group.

The number and percentage of participants with clinically notable laboratory results after baseline will be presented in accordance with Table 2-4, by visit and overall, for randomization period.

Table 2-4 Clinically notable laboratory values and vital signs

**Category**<br>Parameter (unit)	Clinically notable criteria
**Hematology**	
Red blood cell count	> 50% increase AND value > ULN<br>> 30% decrease AND value < LLN
Hemoglobin	> 50% increase AND value > ULN<br>either (> 30% decrease AND value < LLN) OR value < 8.0 g/dL
Hematocrit	> 50% increase AND value > ULN<br>> 30% decrease AND value < LLN

Category<br>Parameter (unit)	Clinically notable criteria
White blood cell count	value > 100,000 cells/mm^3<br>value < 2000 cells/mm^3
Platelet count	value < 50,000 platelets/mm^3
**Blood chemistry**	
Alkaline phosphatase	value > 5 x ULN if baseline was normal OR value > 5 x baseline if baseline was abnormal
ALT (SGPT)	value > 5 x ULN if baseline was normal OR value > 5 x baseline if baseline was abnormal
AST (SGOT)	value > 5 x ULN if baseline was normal OR value > 5 x baseline if baseline was abnormal
Total bilirubin	value > 3 x ULN if baseline was normal OR value > 3 x baseline if baseline was abnormal
BUN	≥ 50% increase
Creatinine	≥ 50% increase
Potassium	value > 6.0 mmol/L<br>value < 3.0 mmol/L
Chloride	> 115 mEq/L<br>< 90 mEq/L
Calcium	corrected serum calcium > 3.1 mmol/L<br>corrected serum calcium < 1.75 mmol/L
Uric acid	> 50% increase
Plasma glucose	> 25% increase<br>value < 40 mg/dL
**Vital signs**	
Weight (kg)	Decrease > 7% from baseline<br>Increase > 7% from baseline
Sitting office SBP (mmHg)	> 180 mmHg<br>< 110 mmHg
Sitting office DBP (mmHg)	> 110 mmHg<br>< 70 mmHg
Pulse (bpm)	value > 100<br>1. value < 60<br>2. value < 50
**Liver function tests**	
ALT (SGPT) OR AST (SGOT)	1. value > 3 x ULN<br>2. value > 5 x ULN<br>3. value > 8 x ULN<br>1. value > 10 x ULN<br>2. value > 20 x ULN

Category<br>Parameter (unit)	Clinically notable criteria
ALT (SGPT) OR AST (SGOT) AND Total bilirubin (TB)	1. ALT or AST > 3 x ULN and TB > 1.5 x ULN
	2. ALT or AST > 3 x ULN and TB > 2 x ULN
	3. ALT or AST > 5 x ULN and TB > 2 x ULN
	4. ALT or AST > 8 x ULN and TB > 2 x ULN
	5. ALT or AST > 10 x ULN and TB > 2 x ULN
	6. ALT or AST > 20 x ULN and TB > 2 x ULN
Alkaline phosphatase	1. > 2 x ULN
	2. > 3 x ULN
	3. > 5 x ULN
Total bilirubin	1. > 1.5 x ULN
	2. > 2 x ULN
	3. > 3 x ULN
Alkaline phosphatase (ALP) AND TB	1. ALP > 3 x ULN AND TB > 2 x ULN
	2. ALP > 5 x ULN AND TB > 2 x ULN
ALT (SGPT) OR AST (SGOT) AND Total bilirubin (TB) AND Alkaline phosphatase (ALP)	1. ALT OR AST > 3 x ULN AND TB > 2 x ULN AND ALP ≤ 2 x ULN
	2. ALT OR AST > 3 x ULN AND TB > 2 x ULN AND ALP ≤ 2 x ULN OR reported Hy's Law case
	3. TB > 3 x ULN AND AST OR ALT ≤ 3 x ULN AND ALP ≤ 1.5 x ULN
	4. ALP > 3 x ULN AND AST AND ALT AND TB are within normal range
Laboratorial and clinical associations	ALT OR AST > 3 x ULN AND (Nausea OR Vomiting OR Fatigue OR General malaise OR Abdominal pain OR (Rash AND Eosinophilia))
**Renal function tests**	
eGFR	1. ≥ 25% decrease
	2. ≥ 50% decrease
Creatinine	≥ 0.3 mg/dL increase
New onset of proteinuria	Urine protein ≥ 300 mg/dL
New onset of hematuria	Urine hemoglobin ≥ 1 mg/dL

ULN: upper limit of the normal range; LLN: lower limit of the normal range; SBP: systolic blood pressure; DBP: diastolic blood pressure; Increases and decreases of parameters as mentioned in the table are from baseline

Participants with liver function tests (ALT, AST, ALP, Total Bilirubin) and renal-related parameters falling within predefined categories of elevations (new and worsened [i.e., those existent before, and that worsened after, randomization] elevations) will be summarized by treatment group in accordance with Table 2-4 for randomization period. Descriptive summaries

by presenting count and percentage of participants with each type of Liver and Renal event in addition to graphical summaries will be displayed, as applicable.

2.7.5 Other safety data

2.7.5.1 ECG and cardiac imaging data

The following quantitative variables will be summarized: heart rate and QRS duration. Summary statistics for continuous data and number and percentage of participants for categorical data (e.g. interpretation) will be provided by treatment and visit for randomization period.

In addition, shift tables comparing the existence or not of abnormalities (atrial fibrillation, atrial flutter, LBB block, RBB block, left ventricular hypertrophy, paced rhythm, myocardial infarction, 1st degree AV block, 2nd degree AV block, 3rd degree AV block or other) on the ECG at baseline and worst-on study results (during the randomization period) will be provided. The categories Yes, No and Missing will be used to reflect where the abnormality of interest is present, absent or an ECG is not performed/is not available, respectively for that participant.

The analysis will be based on SAF.

2.7.5.2 Vital signs

Vital signs including weight, blood pressure and pulse measures will be summarized by treatment group and scheduled visit with standard summary statistics (mean, Q1, median, Q3, standard deviation, min, max), including changes from randomization baseline. Graphical mean plots with 95% CIs for these vital signs will also be provided. Change from baseline will only be summarized for participants with both baseline and post-baseline values and will be calculated as:

- change from baseline = post-baseline value – baseline value

The number and percentage of participants with clinically notable vital signs after baseline will be presented in accordance with Table 2-4 for randomization period, by visit and overall. The analysis will be based on SAF.

Apart from the above analyses, values for vital signs parameters will also be listed at a participant level by treatment group and visit for all participants in safety set (SAF).

Maximum and minimum post-baseline values will be summarized (mean, Q1, median, Q3, standard deviation, min, max) for blood pressure and pulse measures for randomization period.

2.7.5.3 Immunogenicity

All immunogenicity results including anti-XXB750 antibodies (ADA) will be summarized by treatment group and listed by treatment group, participant, and visit/time. Summary statistics including prevalence and incidence of anti-XXB750 antibodies will be provided by treatment and visit/time. A shift table to summarize the proportion of participants' ADA status change over time from baseline (i.e. participants transitioning from negative to positive or vice versa) will also be provided by treatment group.

Post-Baseline category is defined as positive if ADA screening test turns positive at any time post-dose during randomization period of the study, as negative if ADA screening test is negative at all times post-dose during randomization period of the study, and as undefined otherwise. Treatment-induced ADA-positive sample is defined as ADA-positive sample post-baseline during randomization period with ADA-negative sample at baseline. Treatment-boosted ADA-positive sample is defined as ADA-positive sample post-baseline with titer that is at least the titer fold (i.e. 4-fold) change greater than the ADA-positive baseline titer.

A comparison between the counts and percentages of AE observed in ADA-positive and ADA-negative participants will be performed for each MedDRA PT.

The analysis will be based on immunogenicity prevalence set and incidence set.

2.8 Pharmacokinetic endpoints

Sparse PK samples are collected for population PK analysis. Results will be reported separately. Individual drug concentration will be listed in CSR (Clinical Study Report) for participants in PK sub study.

2.9 PD and PK/PD analyses

See Section 2.11 for PD (excluding NT-proBNP) related analyses. See Sections 2.5 and 2.6 for NT-proBNP related analyses.

2.10 Patient-reported outcomes

See Section 2.12 for Kansas City Cardiomyopathy Questionnaire (KCCQ) related analyses.

2.11 Biomarkers

Biomarkers to be analyzed may include, but are not necessarily limited to: cyclic GMP (plasma and urine), urinary cyclic GMP-to-creatinine ratio, urinary albumin-to-creatinine ratio, BNP and hs-Troponin.

Biomarker data will be summarized by treatment and time point. Descriptive summaries will include n, mean (arithmetic and geometric), SD, minimum, Q1, median, Q3, maximum, geometric mean, and 95% confidence interval of geometric mean. Summary statistics by treatment group will be performed for the baseline values, the post-baseline values, and the change from baseline, with baseline defined as the randomization visit. At each post baseline visit, only participants with a value at both baseline and that post-baseline visit will be included. Missing post-baseline values will not be imputed. Analyses will be based on the Full Analysis Set (FAS) and Per-Protocol Set (PPS).

2.12 Other Exploratory analyses

2.12.1 Exploratory variables

Below are the variables for the exploratory analysis:

1. eGFR during randomization period;

2. Biomarkers (See Section 2.11 for analyses);
3. NYHA class during randomization period ;
4. HF signs and symptoms during randomization period ;
5. Kansas City Cardiomyopathy Questionnaire clinical summary score (CSS) and overall summary score (OSS) during randomization period ;
6. Population PK analysis results including mathematic models and parameters (See Section 2.8 for details);
7. Incidence of anti-XXB750 antibodies (See Section 2.7.5.3 for analyses).

2.12.2 Analysis methods

In general, exploratory variables will be analyzed in the FAS unless specified otherwise. Only descriptive statistics will be provided as appropriate, and no formal statistical hypothesis testing nor statistical modelling will be performed.

For continuous variables, unless otherwise specified, summary tables will be provided by treatment and time point. Summary statistics by treatment group will be performed for the baseline values, the post-baseline values, and the change from baseline, with baseline defined as the randomization visit. At each post baseline visit, only participants with a value at both baseline and that post-baseline visit will be included.

For ordinal variables, unless otherwise specified, shift tables of class change (number and percent of participants) will be provided by treatment group at each visit for the randomization period to present.

2.13 Interim analysis

Regular safety data monitoring was planned as specified in Section 2.1 and study protocol Section 4.6. However, no formal interim analysis has been conducted during the study.

3 Sample size calculation

The study is planned to randomize approximately 720 participants in total, and the following calculation is based on the study design before the early study termination.

3.1 Primary endpoint (s)

For the primary endpoint analysis, 600 participants allocated in the ratio of 2:2:3:3 to placebo, XXB750 60 mg dose level, XXB750 120 mg dose level, and highest XXB750 dose level, respectively.

Assuming a common standard deviation of 0.67 for log NT-proBNP change from baseline and a one-sided 2.5% significance level (with adjustments for multiple comparisons using MCPMOD), a sample size of 600 participants (120 in placebo, 120 in XXB750 60 mg dose level, 180 in XXB750 120 mg dose level, and total of 180 in highest XXB750 dose level) will provide a power of 90% if the underlying true maximum NT-proBNP reduction on XXB750 vs. placebo is 23%.

3.2 Secondary endpoint(s)

A common standard deviation of 0.67 for log NT-proBNP change from baseline and a one-sided 2.5% significance level is assumed for all sample size determinations relating to secondary endpoints.

For the first secondary endpoint, a sample size of 120 participants in placebo and 180 participants in XXB750 optimal dose level, respectively, will provide a minimum power of 90% if the true NT-proBNP reduction on XXB750 vs. placebo is 23%.

For the second secondary endpoint, in participants with ACEI/ARB background medication converted to sacubitril/valsartan, a sample size of 120 participants in a group combining the two highest XXB750 target dose levels and 120 participants in sacubitril/valsartan conversion arm will provide approximately 80% power if the underlying true maximum NT-proBNP reduction with adding XXB750 vs. conversion to sacubitril/valsartan is 22%.

4 Change to protocol specified analyses

Due to early study termination, all analysis will be done descriptively. No inferential statistical testing or statistical modelling will be implemented.

5 Appendix

5.1 Imputation rules

5.1.1 Study drug

The missing or partially missing start or end dates will be handled/imputed using the Novartis ADaM Governance Board (AGB) global standard approach. Details will be provided in the study Programming Datasets Specifications (PDS) document.

5.1.2 AE date imputation

The missing or partially missing AE start or end dates will be handled/imputed using the Novartis AGB global standard approach. Details will be provided in the study PDS document.

5.1.3 Concomitant medication date imputation

The missing or partially missing concomitant medication start or end dates will be handled/imputed using the Novartis AGB global standard approach. Details will be provided in the study PDS document.

5.2 AEs coding/grading

Coding of AE will be done per MedDRA dictionary. The scale of severity grading described in section 8.6.1 of the study protocol will be used.

5.3 Laboratory parameters derivations

Details will be provided in the study PDS document.

5.4 Statistical models

5.4.1 Analysis supporting primary objective(s)

Refer to Section 2.5.

5.4.2 Analysis supporting secondary objective(s)

Refer to Section 2.6.

5.5 Rule of exclusion criteria of analysis sets

The following table presents a sample of the rules for participant classification in the analysis sets (Table 5-1).

Table 5-1 Criteria leading to exclusion

Analysis Set	Criteria that cause participants to be excluded
SCR	Not having informed consent;<br>Not having disposition page
RIS	Not in SCR;<br>Not having disposition page;
RAS	Not randomized
FAS	Not in RAS;<br>Mistakenly randomized and no double-blind study drug taken
SAF	Not in RAS;<br>No double-blind study drug taken
PPS	Not in FAS;<br>Not receiving at least three doses/injection of double-blind study drug;<br>Not followed for at least 8 weeks post randomization for open-label arm
PKS	Not randomized;<br>Not receiving any study XXB750 treatment;<br>No available valid PK concenrtration measurement
IPS	Not in SAF;<br>Missing baseline ADA sample and no available post-baseline ADA sample
IIS	Not in SAF;

Analysis Set	Criteria that cause participants to be excluded
	Missing baseline ADA sample or no available post-baseline ADA sample

5.6 KCCQ derivation of scores in each domain

The clinical summary score is a mean of the physical limitation and total symptom scores. The total symptom score is the mean of the symptom frequency and symptom burden scores. Each scale score (the physical limitation, symptom frequency or symptom burden) is calculated as the mean of its item scores and transformed to a 0–100 scale, with higher score indicating higher level of functioning. A score of 100 represents perfect health whereas a score of 0 represents dead. For patients who die, a worst score (score of 0) will be imputed for the clinical summary score at all subsequent scheduled visits after the date of death where the clinical summary score would have been assessed.

Below are the rules each scale score being calculated:

1. Physical Limitation

- Code responses to each of <u>Questions 1a-f</u> as follows:

Extremely limited = 1

Quite a bit limited = 2

Moderately limited = 3

Slightly limited = 4

Not at all limited = 5

- Limited for other reasons or did not do = <missing value>

If at least three of Questions 1a-f are not missing, then compute

- Physical Limitation Score = 100*[(mean of Questions 1a-f actually answered) – 1]/4

Note: references to "means of questions actually answered" imply the following. If there are n questions in a scale, and the subject must answer m to score the scale, but the subject answers only n-i, where n-i >= m, calculate the mean of those questions as

(sum of the responses to those n-i questions) / (n-i)

not

(sum of the responses to those n-i questions) / n

2. Symptom Frequency

- Code responses to Questions 3, 5, 7 and 9 as follows:

<u>Question 3</u>

Every morning = 1

3 or more times a week but not every day = 2

1-2 times a week = 3

Less than once a week = 4

Never over the past 2 weeks = 5

<u>Questions 5 and 7</u>

All of the time = 1

Several times a day = 2

At least once a day = 3

3 or more times a week but not every day = 4

1-2 times a week = 5

Less than once a week = 6

Never over the past 2 weeks = 7

<u>Question 9</u>

Every night = 1

3 or more times a week but not every day = 2

1-2 times a week = 3

Less than once a week = 4

Never over the past 2 weeks = 5

If at least two of Questions 3, 5, 7 and 9 are not missing, then compute:

S3 = [(Question 3) – 1]/4

S5 = [(Question 5) – 1]/6

S7 = [(Question 7) – 1]/6

S9 = [(Question 9) – 1]/4

Symptom frequency score = 100*(mean of S3, S5, S7 and S9)

3. Symptom burden

   - Code responses to each of Questions 4, 6 and 8 as follows:

Extremely bothersome = 1

Quite a bit bothersome = 2

Moderately bothersome = 3

Slightly bothersome = 4

Not at all bothersome = 5

I've had no swelling/fatigue/shortness of breath = 5

If at least one of Questions 4, 6 and 8 is not missing, then compute

Symptom burden score = 100*[(mean of Questions 4, 6 and 8 actually answered) – 1]/4

4. Total symptom score = mean of the following available summary scores:
    - Symptom frequency score
    - Symptom burden score
5. Self-Efficacy score

    If at least one of Questions 10 and 11 is not missing, then compute

    Self-Efficacy score = 100*[(mean of Questions 10 and 11 actually answered) – 1]/4
6. Quality of Life
    - Code responses to Questions 12, 13, 14 as follows:

<u>Question 12</u>

It has extremely limited my enjoyment of life = 1

It has limited my enjoyment of life quite a bit = 2

It has moderately limited my enjoyment of life = 3

It has slightly limited my enjoyment of life = 4

It has not limited my enjoyment of life at all = 5

<u>Question 13</u>

Completely dissatisfied = 1

Mostly dissatisfied = 2

Somewhat satisfied = 3

Mostly satisfied = 4

Completely satisfied = 5

<u>Question 14</u>

I have felt that way all of the time = 1

I have felt that way most of the time = 2

I have occasionally felt that way = 3

I have rarely felt that way = 4

I have never felt that way = 5

If at least one of Questions 12, 13 and 14 is not missing, then compute

Quality of Life = 100*[(mean of Questions 12, 13 and 14 actually answered) – 1]/4

7. Social Limitation

- Code responses to each of Questions 15a-d as follows:

  Extremely limited = 1

  Quite a bit limited = 2

  Moderately limited = 3

  Slightly limited = 4

  Not at all limited = 5

  Limited for other reasons or did not do = <missing value>

If at least two of Questions 15a-d are not missing, then compute

Social Limitation Score = 100*[(mean of Questions 15a-d actually answered) – 1]/4

8. Clinical summary score = mean of the following available summary scores:
   - Physical limitation score
   - Total symptom score
9. Overall summary score = mean of the following available summary scores:
   - Physical limitation score
   - Total symptom score
   - Quality of Life
   - Social Limitation

6 Reference

ICH E9(R1) Harmonized Guideline: addendum on estimands and sensitivity analysis in clinical trials to the guideline on statistical principles for clinical trials. Final version on 20 November 2019.

NOVARTIS

Novartis Research and Development

Clinical Trial Protocol Title:

A multi-center, randomized, placebo- and active-controlled, parallel-group, 24-week proof of concept and dose-finding study to evaluate efficacy, safety, and tolerability of XXB750 in patients with heart failure

Clinical Trial Protocol Number: CXXB750A12201

Version Number: 02 (Amended Protocol) (Clean)

Compound: XXB750

Brief Title: A proof of concept and dose-finding study of XXB750 in patients with heart failure

Study Phase: II

Sponsor Name: Novartis

Regulatory Agency Identifier Number(s): EU-CT Number 2023-504678-39-00

Approval Date: 23-Feb-2024

Property of Novartis
Confidential
May not be used, divulged, published, or otherwise disclosed
without the consent of Novartis

Clinical Trial Protocol Template Version 5.0 dated 14-Jan-2022

Table of contents

Table of contents 2
List of tables 6
List of figures 7
Amendment 02 (23-Feb-2024) 8
Amendment 01 (06-Feb-2024) 9
1 Protocol summary 11
1.1 Summary 11
1.2 Schema 15
1.3 Schedule of activities (SoA) 15
2 Introduction 19
2.1 Study rationale 19
2.2 Background 19
2.3 Benefit/Risk assessment 21
3 Objectives, endpoints, and estimands 23
3.1 Primary estimands 24
3.2 Secondary estimands 25
4 Study design 26
4.1 Overall design 26
4.1.1 Screening Period 26
4.1.2 Randomized Treatment Period 27
4.1.3 Safety Follow-up Period 28
4.2 Scientific rationale for study design 29
4.2.1 Rationale for target study population 30
4.2.2 Rationale for the primary endpoint 31
4.3 Justification for dose 31
4.3.1 Rationale for choice of background therapy 32
4.4 Rationale for choice of control drugs (comparator/placebo) or combination drugs 32
4.5 Rationale for public health emergency mitigation procedures 33
4.6 Purpose and timing of interim analyses/design adaptations 33
4.7 End of study definition 35
5 Study population 35
5.1 Inclusion criteria 36
5.2 Exclusion criteria 36
5.3 Screen failures 38
5.3.1 Replacement policy 39

5.3.2 Participant numbering 39
6 Study treatment(s) and concomitant therapy 39
6.1 Study treatment(s)....................39
6.1.1 Additional study treatments 40
6.1.2 Treatment arms/group 40
6.1.3 Treatment duration 41
6.2 Preparation, handling, storage, and accountability....................41
6.2.1 Handling of study treatment....................42
6.2.2 Handling of other treatment 43
6.2.3 Instruction for prescribing and taking study treatment 43
6.3 Measures to minimize bias: randomization and blinding....................44
6.3.1 Treatment assignment, randomization 44
6.3.2 Treatment blinding 44
6.3.3 Emergency breaking of assigned treatment code....................46
6.4 Study treatment compliance 47
6.4.1 Recommended treatment of adverse events....................47
6.5 Dose modification....................47
6.5.1 Definitions of dose limiting toxicities (DLTs)....................48
6.5.2 Follow-up for toxicities....................49
6.6 Continued access to study treatment after the end of the study....................50
6.6.1 Post trial access 50
6.7 Treatment of overdose 50
6.7.1 Reporting of study treatment errors including misuse/abuse....................51
6.8 Concomitant and other therapy....................51
6.8.1 Concomitant therapy 51
6.8.2 Prohibited medication 52
6.8.3 Rescue medicine....................53
7 Discontinuation of study treatment and participant discontinuation/withdrawal....................53
7.1 Discontinuation of study treatment....................53
7.2 Participant discontinuation from the study 54
7.3 Withdrawal of informed consent and exercise of participants' data privacy rights 54
7.4 Lost to follow-up 55
7.5 Early study termination by the Sponsor....................55
8 Study Assessments and Procedures....................55
8.1 Screening 55

8.2 Participant demographics/other baseline characteristics ... 56
8.3 Efficacy assessments ... 56
8.3.1 NT-proBNP ... 56
8.3.2 Secondary efficacy assessments ... 57
8.3.3 Appropriateness of efficacy assessments ... 57
8.4 Safety assessments ... 57
8.4.1 Physical examinations ... 57
8.4.2 Vital signs ... 57
8.4.3 Height and weight ... 58
8.4.4 Electrocardiograms ... 58
8.4.5 Clinical safety laboratory tests ... 58
8.4.6 Pregnancy testing ... 59
8.4.7 Other safety evaluations ... 60
8.4.8 Appropriateness of safety measurements ... 61
8.5 Additional assessments ... 61
8.5.1 Clinical Outcome Assessments (COAs) ... 61
8.5.2 Other assessments ... 62
8.6 Adverse events (AEs), serious adverse events (SAEs), and other safety reporting ... 63
8.6.1 Adverse events ... 63
8.6.2 Serious adverse events ... 66
8.6.3 SAE reporting ... 67
8.6.4 Pregnancy ... 69
8.6.5 Disease-related events and/or disease-related outcomes not qualifying as AEs or SAEs ... 69
8.6.6 Adverse events of special interest ... 70
8.7 Pharmacokinetics ... 70
8.8 Biomarkers ... 70
8.9 Immunogenicity assessments ... 72
8.10 Medical resource utilization and health economics ... 72
9 Statistical considerations ... 72
9.1 Analysis sets ... 72
9.2 Statistical analyses ... 73
9.2.1 General considerations ... 73
9.2.2 Participant demographics and other baseline characteristics ... 73
9.2.3 Treatments ... 74
9.3 Primary endpoint(s)/estimand(s) analysis ... 74

9.3.1 Definition of primary endpoint(s) 74
9.3.2 Statistical model, hypothesis, and method of analysis 74
9.3.3 Handling of intercurrent events of primary estimand (if applicable) 76
9.3.4 Handling of missing values not related to intercurrent event 77
9.3.5 Multiplicity adjustment (if applicable) 77
9.3.6 Sensitivity analyses 77
9.3.7 Supplementary analysis 77
9.4 Secondary endpoint(s)/estimand(s) analysis 78
9.4.1 Efficacy and/or pharmacodynamic endpoint(s) 78
9.4.2 Safety endpoints 79
9.5 Exploratory endpoint(s)/estimand(s) analysis 80
9.5.1 Exploratory variables 81
9.5.2 Analysis methods 81
9.5.3 Pharmacokinetics 83
9.5.4 Biomarkers 84
9.5.5 Immunogenicity 84
9.5.6 Patient reported outcomes 84
9.6 Other safety analyses 84
9.7 Other analyses 84
9.7.1 Subgroup analyses 84
9.7.2 DNA 85
9.8 Interim analysis 85
9.9 Sample size determination 85
9.9.1 Primary endpoint(s) 86
9.9.2 Secondary endpoint(s) 86
10 Supporting documentation and operational considerations 86
10.1 Appendix 1: Regulatory, ethical, and study oversight considerations 86
10.1.1 Regulatory and ethical considerations 86
10.1.2 Informed consent process 88
10.1.3 Data protection 89
10.1.4 Committees structure 90
10.1.5 Data quality assurance 90
10.1.6 Source documents 92
10.1.7 Publication policy 92
10.1.8 Protocol adherence and protocol amendments 93
10.2 Appendix 2: Abbreviations and definitions 94

10.2.1 List of abbreviations 94
10.2.2 Definitions 97
10.3 Appendix 3: Clinical laboratory tests 100
10.3.1 Clinically notable laboratory values and vital signs 100
10.4 Appendix 4: Participant Engagement 102
10.5 Appendix 5: Liver safety monitoring 103
10.5.1 Liver event and laboratory trigger definitions & follow-up requirements 103
10.6 Appendix 6: Renal safety monitoring 106
10.6.1 Specific Renal Alert Criteria and Actions and Event Follow-up 106
10.7 Appendix 7: Managing hypotension 107
10.8 Appendix 8: Managing hyperkalemia 109
11 References 111

List of tables

Table 1-1 Assessment Schedule 17
Table 3-1 Objectives and related endpoints 23
Table 4-1 Minimum required pre-study daily doses of ACEIs and ARBs 27
Table 4-2 Overview of the planned staggered enrollment strategy 34
Table 6-1 Investigational and control drug 39
Table 6-2 Auxiliary treatments 40
Table 6-3 Treatment arms, dose levels, and planned doses administered at dispensing visits during the Randomized treatment period 41
Table 6-4 Blinding and unblinding plan 45
Table 6-5 Diagnostic assessments to determine alternative causes of observed LFT abnormalities 49
Table 8-1 Clinical Safety Laboratory Tests 59
Table 8-2 MedDRA PT related to the underlying heart failure 68
Table 9-1 Selecting a winner based on combination of change of KCCQ CSS and change of NYHA class 82
Table 10-1 Clinically notable hematology abnormalities 100
Table 10-2 Clinically notable blood chemistry abnormalities 100
Table 10-3 Liver event and laboratory trigger definitions 103
Table 10-4 Follow up requirements for liver laboratory triggers - ALT, AST, TBL 104
Table 10-5 Follow-up requirements for liver laboratory triggers based on isolated elevations in TBL 105

Table 10-6 Renal event criteria and corresponding actions to be taken upon their occurrence 106

List of figures

Figure 1-1 Study design 15
Figure 6-1 Modification of injectable study medication 48
Figure 9-1 Dose-response curve of candidate models 75
Figure 10-1 Flow chart for managing hypotension 108

Amendment 02 (23-Feb-2024)

Amendment rationale

The purpose of this amendment is to make minor editorial modifications to the protocol.

Changes to the protocol

Changes to specific sections of the protocol are shown in the track changes version of the protocol using strikethrough red font for deletions and red underline for insertions.

List of changes to the protocol are listed below:

Section 3.2 Secondary estimands

- The formatting of the paragraph describing the treatment of interest of the second secondary estimand was corrected to match the formatting of the original protocol.

Section 5.2 Exclusion criteria

- The formatting for Exclusion criterion #26 was corrected to clarify that each subpoint is a component of the main criterion rather than a stand-alone one.

IRB/IEC

A copy of this amended protocol will be sent to the Institutional Review Boards (IRBs)/Independent Ethics Committees (IECs) and Health Authorities.

The changes described in this amended protocol require IRB/IEC approval prior to implementation.

Amendment 01 (06-Feb-2024)

Amendment rationale

The purpose of this amendment is to make modifications to the protocol that were requested by the EU Health Authorities.

In addition, minor editorial changes are made to correct typographical errors, to make some clarifications, and to resolve some minor inconsistencies in various sections of the protocol.

Changes to the protocol

Changes to specific sections of the protocol are shown in the track changes version of the protocol using strikethrough red font for deletions and red underline for insertions.

List of changes to the protocol are listed below:

Section 1.1 Summary

- A footnote was added to table 'Objectives, Endpoints, and Estimands' to clarify that if the treatment arms 4a and 4b are eliminated (see Section 4.6), the second secondary objective will evaluate only the 120 mg XXB750 arm to sacubitril/valsartan.
- The inclusion criterion was modified to require that other evidence-based therapy for heart failure should also be considered e.g., cardiac resynchronization therapy and implantable cardioverter-defibrillator in selected patients, as recommended by guidelines in selecting patients for the study.
- The exclusion criterion was updated to exclude patients with previous intolerability to any ARB (as per the investigator's judgement).

Section 3 Objectives, endpoints and estimands

- Table 3-1 was updated to include a footnote clarifying that in the event treatment arms 4a and 4b are eliminated (see Section 4.6), the second secondary objective will evaluate only the 120 mg XXB750 arm to sacubitril/valsartan.

Section 3.2 Secondary estimands

- This section was updated to reflect the estimand of the second secondary endpoint in the event treatment arms 4a and 4b are eliminated.

Section 4.1.2 Randomized Treatment Period

- This section was modified to clarify the distribution of participants across the different treatment arms according to their background anti-RAS treatment (i.e., ACEI/ARB vs. ARNI).

Section 4.6 Purpose and timing of interim analyses/design adaptations

- This section was modified to clarify that in case of elimination of treatment arms 4a and 4b, 180 participants will be allocated to treatment arm 3 from Group 2 for a total/overall sample size of 300 participants in this arm.
- A statement was added to confirm that the sponsor will inform the local health authorities as per the local regulations in case of any adaptations to study design based on feedback from the DMC.

Section 5.1 Inclusion criteria

- Inclusion criterion #7 was modified to require that other evidence-based therapy for heart failure should also be considered, e.g., cardiac resynchronization therapy and implantable cardioverter-defibrillator in selected patients, as recommended by guidelines in selecting patients for the study. Also, it was clarified that guideline recommended HF medications should be stable in dose for at least 4 weeks before screening.

Section 5.2 Exclusion criteria

- Exclusion criterion #6 was updated to exclude patients with previous intolerability to any ARB (as per the investigator's judgment). Exclusion criterion #13 was updated to exclude patients with known right ventricular dysfunction that is deemed to increase susceptibility to the hypotensive effects of therapy in the opinion of the investigator. Exclusion criterion #15 was updated to exclude patients with CRT implantation within 3 months of screening. Exclusion criterion #18 was updated to exclude patients with planned/anticipated major cardiac surgery during the involvement of the participant in the study.

Section 6.1.1 Additional study treatments

- Table 6-2 Auxillary treatments was updated to delete the statement "Each packaging will be labeled as per country requirements" since none of the participating countries have such requirements.

Section 9.4.1 Efficacy and/or pharmacodynamic endpoint(s)

- This section was updated to note that in the event treatment arms 4a and 4b are eliminated, the comparison made in the second secondary endpoint will be between only the 120 mg XXB750 and the sacubitril/valsartan arms.

Section 9.9.1 Primary endpoint(s)

- This section was updated to note the sample size and statistical power for evaluating the primary endpoint in the event treatment arms 4a and 4b are eliminated.

Section 9.9.2 Secondary endpoint(s)

- This section was updated to note the sample size and statistical power for evaluating the first and second secondary endpoints in the event treatment arms 4a and 4b are eliminated.

Miscellaneous corrections of typographical errors were made throughout the protocol.

IRB/IEC

A copy of this amended protocol will be sent to the Institutional Review Boards (IRBs)/Independent Ethics Committees (IECs) and Health Authorities.

The changes described in this amended protocol require IRB/IEC approval prior to implementation.

1 Protocol summary

1.1 Summary

Protocol Title:

A multicenter, randomized, placebo- and active-controlled, parallel-group, 24-week proof-of-concept and dose-finding study to evaluate efficacy, safety, and tolerability of XXB750 in patients with heart failure

Brief Title:

A proof-of-concept and dose-finding study of XXB750 in patients with heart failure

Purpose:

The purpose of this phase 2 study is to investigate the efficacy, safety, and tolerability of different doses of XXB750 given in different dosing regimens compared to placebo in heart failure (HF) patients with New York Heart Association classification II-III (NYHA class II-III) with left ventricular ejection fraction (LVEF) < 50% receiving standard of care (SoC) therapy. This study will also compare different dose levels of XXB750 versus a conversion to sacubitril/valsartan for the same outcomes. The results of this study will provide information needed for designing the XXB750 phase 3 HF program.

Study Indication /Medical Condition:

Heart failure

Treatment type:

Biological

Study type:

Interventional

Objectives, Endpoints, and Estimands:

Objectives	Endpoints
**Primary**	
• To evaluate the efficacy and dose-response relationship of three XXB750 target dose levels compared to placebo in reducing N-terminal prohormone B-type natriuretic peptide (NT-proBNP) from baseline to Week 16 in symptomatic HF patients with LVEF < 50% treated with standard of care, including ACEI/ARB or sacubitril/valsartan.<br>The primary clinical question of interest is: Is there a dose-response signal, and if so, to characterize the dose-response relationship of three XXB750 target dose levels versus placebo in reducing NT-proBNP from baseline to Week 16 in participants with heart	• Change in log NT-proBNP from baseline to Week 16

failure (NYHA classes II-III) with LVEF <50% regardless of type of baseline background HF therapy, discontinuation from study treatment, change in the dose of concomitant medications and use of prohibited concomitant medication.	
**Secondary**	
• To evaluate the treatment effect of the highest XXB750 target dose level compared to placebo in reducing NT-proBNP from baseline to Week 16 in symptomatic HF patients with LVEF < 50% treated with standard of care, including ACEI/ARB or sacubitril/valsartan.<br>The estimand of this first secondary objective will further demonstrate the efficacy of highest XXB750 target dose level compared with placebo, regardless of type of baseline background HF therapy, discontinuation from study treatment, change in the dose of background HF medications and use of prohibited concomitant medication; it will provide an estimate with a p-value in a hierarchical hypothesis testing procedure with overall type I error control after a dose-response signal is established in the primary objective.<br>• To evaluate the treatment effect of combined two highest XXB750 target dose levels administered in addition to a background of ACEI/ARB versus conversion from ACEI/ARB to sacubitril/valsartan, in reducing NT-proBNP from baseline to Week 16.*<br>• To evaluate the safety and tolerability of XXB750 up-titration regimens and dose levels.	• Change in log NT-proBNP from baseline to Week 16<br>• Adverse events, safety laboratory parameters, and vital signs from baseline to end of study (EOS)

*In the event treatment arms 4a and 4b are eliminated (see Section 4.6), the second secondary objective will evaluate only the 120 mg XXB750 arm to sacubitril/valsartan arm.

Trial Design:

Study CXXB750A12201 is a multicenter, randomized, placebo- and active-controlled, parallel-group study which is comprised of three periods:

- A 7-days screening period
- A 16-week parallel-group randomized treatment period, on either
  i) double-blind placebo-controlled treatment (XXB750 vs. placebo) or
  ii) open-label treatment (sacubitril/valsartan)
- An 8-week safety follow-up period

The study will enroll participants with HF (NYHA class II-III), elevated NT-proBNP levels and LVEF < 50% receiving SoC HF therapy.

Brief Summary:

The purpose of this study is to assess the efficacy and safety of different target doses of XXB750 given in different up-titration regimens on NT-proBNP change from baseline at week 16 compared to placebo and to sacubitril/valsartan in participants with symptomatic HF with LVEF < 50%.

XXB750 is a fully human monoclonal antibody with potent agonistic activity at the natriuretic peptide receptor 1 (NPR1). NPR1 catalyzes cyclic guanosine monophosphate (cGMP) synthesis, thereby enhancing natriuresis, diuresis and vasodilation as well as protecting from cardiac fibrosis. Consistent with its mechanism of action, XXB750 was shown to decrease cardiac hypertrophy and NT-proBNP levels in mice lacking atrial natriuretic peptide.

Patients with symptomatic HF (NYHA class II-III), elevated NT-proBNP levels and a LVEF < 50% despite receiving an ACEI, an ARB, or an angiotensin receptor neprilysin inhibitor (ARNI), and other guideline-recommended therapies for HFrEF and HFmrEF, will be recruited into the study. Participants must be on ACEI/ARB doses equivalent to enalapril 10 mg/day or higher (see Table 4-1) or sacubitril/valsartan doses of 49/51 mg twice daily (bid) or higher. Participants who successfully complete the Screening period are planned to be randomized to one of three target dose levels of XXB750 (60 mg, 120 mg or 240 mg) or matching placebo. In addition, a cohort of patients receiving an ACEI/ARB will be randomly selected to be converted to receive open-label sacubitril/valsartan in the randomized treatment period, replacing their pre-study ACEI/ARB. Participants who receive an ACEI and are randomized to receive open-label sacubitril/valsartan must undergo a 36-hr ACEI-free wash-out period before starting to take the randomized treatment (open-label sacubitril/valsartan) to minimize the risk of occurrence of angioedema. All patients will continue to receive their other background HF medication. Overall, a total of approximately 720 participants will be randomized into this study.

The randomized treatment period will last 16 weeks. Participants randomized to XXB750 or its matching placebo will receive 1 subcutaneous injection every 4 weeks, with a total of 4 injections during the 16-week treatment period. Participants randomized to conversion to sacubitril/valsartan will receive sacubitril/valsartan for 16 weeks. At the end of the 16-week randomized treatment period, all assigned study treatments will be discontinued and the participants will enter an 8-week safety follow-up period during which they will be treated according to the investigator's clinical judgment. Assessment of NT-proBNP will occur at baseline, week 4, week 8, week 12, week 16 (timepoint of primary endpoint), week 20 and week 24. Safety, efficacy and/or pharmacokinetic assessments will be performed serially throughout all three study periods.

Study Duration:

24 weeks

Treatment Duration:

16 weeks

Visit Frequency:

Every 1-4 weeks

Treatment of Interest

XXB750 target dose levels of 60 mg, 120 mg, and 240 mg or matching placebo or sacubitril/valsartan. Adaptive design features of the protocol may impact the planned target dose levels (Section 4.6).

Treatment Groups

Participants will be randomized to be treated during a 16-week randomized treatment period with:

- Placebo matching XXB750
- XXB750 60 mg target dose
- XXB750 120 mg target dose
- XXB750 240 mg target dose in one of two up-titration regimens
- Sacubitril/valsartan at target doses of 97/103 mg bid (only participants previously treated with ACEI/ARB)

Treatment groups may be modified per adaptive design feature of the study design (Section 4.6).

Number of Participants:

720 participants female or male participants aged 18 years or older with symptomatic HF and LVEF < 50% will be randomized to study intervention. Participants with LVEF > 40% will be limited to approximately 25% of the total randomized participants.

Key Inclusion Criteria

- Written informed consent must be obtained before any assessment is performed.
- Male and female outpatients who are ≥ 18 years old.
- Symptom(s) of HF NYHA class II-III at Screening.
- LVEF < 50% (most recent local measurement, made within 6 months prior to or during Screening using echocardiography, MUGA, CT scanning, MRI or ventricular angiography is acceptable, provided no subsequent measurement ≥ 50%).
- NT-proBNP ≥ 600 pg/ml if in sinus rhythm or
  NT-proBNP ≥ 900 pg/ml if in atrial fibrillation/flutter at Screening.
- Receiving an ACEI or an ARB at a stable dose of at least enalapril 10 mg/d or equivalent for at least 4 weeks before Screening or receiving sacubitril/valsartan at a stable dose of at least 49/51 mg bid for at least 4 weeks before Screening.
- Receiving other guideline recommended HF therapies as deemed appropriate by the investigator and that are stable in dose for at least 4 weeks before Screening, unless contraindicated, not tolerated, or not available to patient. Other evidence-based therapy for heart failure should also be considered, e.g., cardiac resynchronization therapy and an implantable cardioverter-defibrillator in selected patients, as recommended by guidelines.

Key Exclusion Criteria

- Current acute decompensated HF (exacerbation of chronic HF manifested by signs and symptoms that may require intravenous therapy) or hospitalization for HF within 3 months prior to screening.
- Office systolic blood pressure (SBP) ≥ 180 mmHg or < 105 mmHg at screening or at randomization.

- In subjects with ACEI/ARB medication at screening, previous inability to tolerate any dose of sacubitril/valsartan or any ARB (as per the investigator's judgment).
- Serum potassium > 5.4 mmol/L at screening.
- Estimated GFR (eGFR) < 30 mL/min/1.73m^2 at screening as measured by the CKD-EPI formula.
- Known history of angioedema.

Data Monitoring/Other Committee:

Yes (see Section 10.1.4 Committees Structure)

Key words

Heart failure, randomized, double-blind, dose finding, XXB750

1.2 Schema

Figure 1-1 Study design

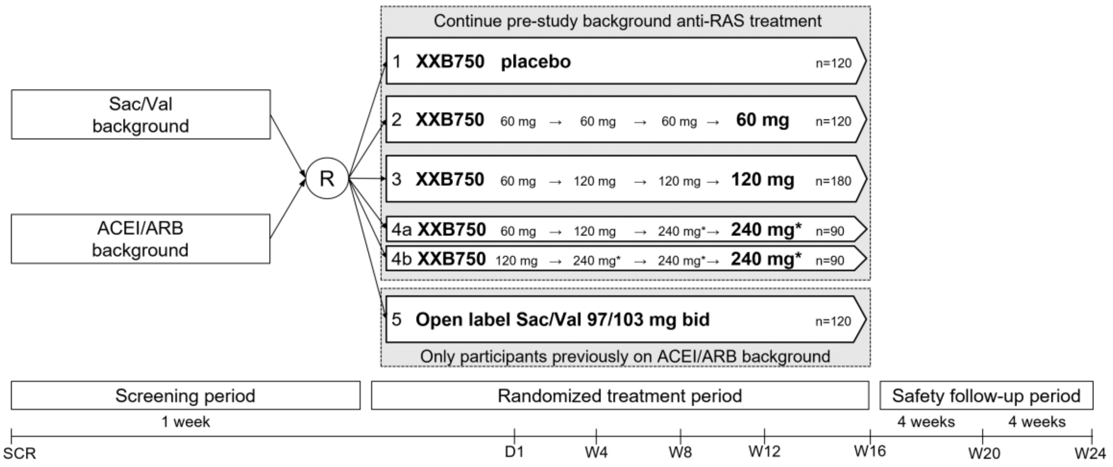

Sac/Val: Open-label sacubitril/valsartan. *May be modified per adaptive design feature of the study (Section 4.6). Doses of Sac/Val, ACEI or ARB in arms 1-4 equal to dose at randomization.

1.3 Schedule of activities (SoA)

Table 1-1 lists all scheduled assessments throughout the study. In addition to the protocol-required visits, participants may be seen at any time throughout the study at the discretion of the investigator to follow-up on any new laboratory abnormalities or adverse events (AEs).

Participants should be seen for all visits/assessments as outlined in the SoA or as close to the designated day/time as possible. Day 1 will be considered the reference visit for all study visits during the randomized treatment period, regardless of occurrence of any unscheduled visits. If one visit is missed, postponed, or brought forward, it should not result in the next visit being postponed or brought forward. For details on administration of injectable study medication in

this situation, please see Section 6.1.2. The next visit, if at all possible, should adhere to the original time schedule.

Participants who discontinue from study treatment are to complete the end of treatment visit as soon as possible, and continue to attend the follow-up visits as indicated in the SoA.

Participants who discontinue from the study should be scheduled for a final evaluation visit if they agree, as soon as possible, at which time all the assessments listed for the final visit of the randomized treatment period will be performed. At this final visit, all dispensed study treatment should be reconciled, and the adverse events and concomitant medications not previously reported must be recorded on the appropriate CRF.

Patient Reported Outcome (PRO) measure(s) must be completed by the participant before any assessments are performed at any given visit.

Throughout the randomized treatment and safety follow-up periods, participants will undergo safety and biomarker laboratory assessments as well as pharmacokinetic (PK) sampling per the schedule shown in Table 1-1. After randomization, all laboratory evaluations for planned visits will be performed through the central laboratory or other authorized laboratory. Unscheduled laboratory assessments can be performed locally as necessary or as permitted by the protocol.

As per Section 4.5, during a public health emergency as declared by local or regional authorities i.e., pandemic, epidemic or natural disaster that limits or prevents on-site study visits, alternative methods of providing continuing care may be implemented by the Investigator as the situation dictates. If allowable by a local health authority, national and local regulations and depending on operational capabilities, phone calls, virtual contacts (e.g., tele consultation) or visits by site staff/off-site healthcare professional(s) staff to the participant´s home, can replace certain protocol assessments, for the duration of the disruption until it is safe for the participant to visit the site again. If the Investigator delegates tasks to an off-site healthcare professional, the Investigator must ensure the individual(s) is/are qualified and appropriately trained to perform assigned duties. The Investigator must oversee their conduct and remains responsible for the evaluation of the data collected.

Table 1-1 Assessment Schedule

Epoch	Screening	Randomized Treatment										Safety Follow-up	
**Visit Name**	**Screening**	**Randomization**	**Day 2 Call**	**Week 2**	**Week 4**	**Day 30 Call**	**Week 8**	**Day 58 Call**	**Week 12**	**Day 86 Call**	**Week 16 EOT**	**Week 20**	**Week 24 EOS**
**Visit Numbers[1]**	**10**	**101**	**102**	**103**	**104**	**105**	**106**	**107**	**108**	**109**	**9998**	**201**	**202**
**Days**	**-7 to 1**	**1**	**2**	**14**	**28**	**30**	**56**	**58**	**84**	**86**	**112**	**140**	**168**
Informed consent	X												
Genetic consent (optional)	X												
Inclusion / Exclusion criteria	X	X											
Demography	X												
Medical history/current medical conditions	X												
Alcohol and smoking history	X												
Prior/Concomitant Medications	X	X	X	X	X	X	X	X	X	X	X	X	X
Complete Physical Examination	S										S		S
Abbreviated physical examination		S		S	S		S		S			S	
Body Height (H) and Weight (W)	H/W	W		W	W		W		W		W	W	W
Electrocardiogram (ECG)	X										X		X
Echocardiogram (optional)	S												
Vital Signs	X	X		X	X		X		X		X	X	X
Central lab blood chemistry	X			X[2]	X[2]		X[2]		X[2]		X	X[2]	X
Hematology	X										X		X
Coagulation Panel	X			X	X		X		X		X	X	X
NT-proBNP	X	X			X[3]		X[3]		X[3]		X	X	X
NYHA classification (HF signs and symptoms)	X	X			X		X		X		X	X	X
IWRS/IRT Registration	X	X		X	X		X		X		X	X	X
Study drug dispensation/administration[4]		X		X	X		X		X				
Study medication compliance[5]				S	S		S		S		S		

Epoch	Screening	Randomized Treatment										Safety Follow-up	
**Visit Name**	**Screening**	**Randomization**	**Day 2 Call**	**Week 2**	**Week 4**	**Day 30 Call**	**Week 8**	**Day 58 Call**	**Week 12**	**Day 86 Call**	**Week 16 EOT**	**Week 20**	**Week 24 EOS**
**Visit Numbers[1]**	**10**	**101**	**102**	**103**	**104**	**105**	**106**	**107**	**108**	**109**	**9998**	**201**	**202**
**Days**	**-7 to 1**	**1**	**2**	**14**	**28**	**30**	**56**	**58**	**84**	**86**	**112**	**140**	**168**
Adverse Events		X	X	X	X	X	X	X	X	X	X	X	X
Biomarker urine collection		X[3]					X[3]		X[3]		X		
Biomarker blood collection		X[3,6]					X[3]		X[3]		X		
PK blood collection[7]		X[8]			X[3,8]				X[3,8]		X		X
Anti-drug antibody[7]		X[3]			X[3]				X[3]		X		X
Exploratory DNA Sampling (optional)[9]		X											
Urinalysis	X	X		X	X		X		X		X	X	X
Assessment of fertility	S												
Pregnancy Test (serum)	X										X		X
Pregnancy Test (urine)		S			S		S		S			S	
KCCQ questionnaire		X									X		
Study period completion information	X										X		X

[X] Assessment to be recorded in the clinical database or received electronically from a vendor
[S] Assessment to be recorded in the source documentation only
[1] Visit structure given for internal programming purpose only
[2] Abbreviated safety panel (Please refer to Table 8-1)
[3] Samples must be taken prior to study drug administration.
[4] For participants on ACEI and randomized to treatment arm 5, a 36-hour washout period must be observed: Open-label sacubitril/valsartan to be started in evening of Day 2. Dispensing visit at Week 2 is for only patients randomized to receive open-label sacubitril/valsartan, which can be used as a dose titration visit.
[5] Only applicable to participants randomized to conversion to open-label sacubitril/valsartan (treatment arm 5)
[6] Additional collection with P100 tube. Please refer to Section 8.8.
[7] Not applicable to participants randomized to conversion to open-label sacubitril/valsartan (treatment arm 5)
[8] Sample to be collected 2-4h post dose.
[9] To be collected only for participants signing optional DNA consent.

2 Introduction

2.1 Study rationale

The purpose of this study is to investigate the efficacy, safety, and tolerability of three dose levels of XXB750 in HF patients (NYHA class II-III) with LVEF < 50% receiving standard of care (SoC) HF therapies. This study will provide evidence to understand the dose-response relationship of XXB750 in reducing NT-proBNP, a prognostic biomarker of HF clinical outcomes, and to choose the optimal dosing regimen to investigate in future phase 3 studies. In addition, this study will help to assess the benefit of XXB750 relative to placebo when given on top of SoC HF therapy, as well as its benefit in ACEI/ARB treated patients compared to converting them to sacubitril/valsartan (i.e., direct comparison of XXB750 vs sacubitril/valsartan).

2.2 Background

Heart failure (HF) is a major public health problem associated with a high mortality rate, frequent hospitalizations, and poor quality of life (QoL). Worldwide, an estimated 64.3 million people were living with heart failure in 2017, with a prevalence between 1-3% of the general adult population in developed countries (Groenewegen et al 2020, Savarese et al 2022). In the United States, the prevalence of HF is 6 million individuals, which is approximately 1.8% of the total US population (Roger 2021). Heart failure is associated with frequent hospitalizations, with 30-40% of patients having a history of hospitalization for HF (Crespo-Leiro et al 2016) and 22% of patients being re-admitted for HF within 1 year of their initial diagnosis (Lawson et al 2019).

Heart failure is a complex clinical syndrome that most commonly causes structural or functional impairment of ventricular filling or emptying, ultimately resulting in the heart's inability to meet the body's need for adequate supplies of oxygenated blood. The natural course of HF includes deterioration in symptoms, often leading to repeated hospitalizations for acute decompensations, and eventually death most frequently due to arrythmia (i.e., sudden death) or progressive pump failure (Desai et al 2015).

Heart failure with reduced left ventricular ejection fraction (HFrEF) is regarded as a common type of HF and is characterized by an LVEF ≤ 40%; it is the best studied type of HF. The current standard of care of pharmacologic treatment for HFrEF is comprised of four main drug classes: blockers of the renin angiotensin system (RAS) with either angiotensin converting enzyme inhibitors (ACEI), anangiotensin II receptor blockers (ARB), or angiotensin receptor neprilysin inhibitor (ARNI), β-blockers, mineralocorticoid receptor antagonists (MRAs) and sodium-glucose cotransporter 2 (SGLT2) inhibitors. These four classes have been shown to reduce morbidity and mortality in patients with HFrEF and are recommended as standard-of-care treatment as per recently published guidelines for the treatment of HF (McDonagh et al 2021, Heidenreich et al 2022). Adjacently situated to HFrEF on the LVEF spectrum is heart failure with mildly reduced ejection fraction (HFmrEF), which is characterized by an LVEF > 40% but < 50%.

Evidence based on analyses of large outcome trials suggests that drug classes effective in HFrEF may also be effective in HFmrEF patients, thereby supporting recommendations for using them

in this HF population in recent international HF treatment guideline updates (McDonagh et al 2021, Heidenreich et al 2022).

Despite the treatment of HFrEF patients with quadruple therapy as recommended by guidelines, it is projected that lifetime morbidity and mortality remain high (Vaduganathan et al 2020, Tromp et al 2022). In a cross-trial analysis, while overall mean survival was estimated to be prolonged with quadruple therapy as compared to conventional therapy, survival remains reduced by at least 10 years compared to the population's average life expectancy (Vaduganathan et al 2020). Similarly, a recent network meta-analysis (Tromp et al 2022) found prolonged survival with quadruple therapy but with even higher mortality rates than in the cross-trial analysis cited above. Thus, with considerable residual morbidity and mortality rates, the need for better therapies for HF remains unmet and high.

Disease development and progression of HF is intimately linked to an excessive neurohormonal response, resulting in an imbalance between detrimental pathogenic factors and compensatory mechanisms. Overactivation of the RAS and sympathetic nervous system (SNS) is believed to be harmful in the long-term because they result in sodium retention, fluid overload, increased blood pressure, and cardiac fibrosis and hypertrophy, especially in the absence of adequate levels of natriuretic peptides (NPs), which are regarded as a beneficial compensatory response via their direct vasodilator and natriuretic actions, as well as their antiproliferative and antihypertrophic effects (Levin et al 1998, Gardner et al 2007, Pandey 2008). This has been demonstrated by sacubitril/valsartan, an ARNI that simultaneously blocks angiotensin II, while enhancing NP action by blocking NP metabolism via the enzyme neprilysin, resulting in improved survival and reduced morbidity (McMurray et al 2014).

However, elevating NP levels by blocking neprilysin-mediated degradation (NEPi) may not fully leverage the beneficial potential of the NP system as the extent of NEPi-mediated NP agonism is limited by the amount of endogenous ANP and/or BNP levels. Also, neprilysin inhibition blocks only one pathway of NP breakdown, leaving other NP metabolic pathways, such as enzymatic degradation by dipeptidyl peptidase 4 and deactivation via NPR3 receptor binding, unaffected.

Direct natriuretic peptide type 1 receptor (NPR1) agonism may offer a more efficient way of targeting the NP system. In a recently published first-in-human (FIH) study, the NPR1 agonist M-atrial natriuretic peptide (MANP), an analog of human ANP, was shown to increase cGMP levels and natriuresis and to lower aldosterone levels and blood pressure when administered subcutaneously (SC) to hypertensive patients (Chen et al 2021).

XXB750 is a fully human monoclonal IgG1 antibody agonist of the NPR1. The action of XXB750 is specific for NPR1, with XXB750 selectively binding to and activating NPR1, while it does not bind to NPR3 or activate NPR2 in vitro. XXB750 replicates the action of ANP (increasing plasma cGMP, lowering BP and increasing diuresis) in non-clinical pharmacology studies. It effectively substitutes for ANP in ANP knockout mice, reversing both cardiac hypertrophy and elevations in NT-proBNP. XXB750 also increases plasma cGMP and lowers BP in cynomolgus monkeys.

XXB750 has a half-life of ~16 days in healthy volunteers and has been shown to be safe and well tolerated in single SC doses up to 450 mg in healthy volunteers with mildly elevated blood pressure. Pharmacodynamic effects (cGMP increase, blood pressure lowering) of XXB750 (for

doses ≥ 120 mg) were of greater magnitude than what was seen with sacubitril in healthy volunteers. Also, single 120 mg SC doses of XXB750 have been tested in HF patients with ACEI/ARB and other background HF medications. XXB750 was found to have an acceptable safety and tolerability profile. This data, together with evidence of the beneficial effects of ANP and other ANP mimetics in heart failure patients, provides a scientific rationale to develop XXB750 for the treatment of heart failure. Refer to the XXB750 investigator's brochure for more detailed information on the safety, pharmacokinetics, and pharmacodynamic biomarker data available on this biologic.

The current study is a combined phase 2a/2b study which aims to establish proof-of-concept and dose range relationship for XXB750 in reducing NT-proBNP levels in HF patients (NYHA class II-III) with LVEF < 50%, to inform the dose regimen of XXB750 to be tested in Phase 3, and to further characterize the benefit-risk profile of XXB750 in patients with HF.

2.3 Benefit/Risk assessment

Participants are expected to benefit from extensive monitoring of their HF condition and symptoms during the course of the study. Participants will continue to receive their background standard of care HF medications, including ACEIs/ARBs or ARNI, during the duration of the study. Some patients treated with ACEIs will be randomly assigned to receive open-label the ARNI sacubitril/valsartan, which is considered by international HF treatment guidelines to be preferred over ACEIs in HFrEF due to improved efficacy in reducing long-term morbidity and mortality (Fiuzat et al 2022, Heidenreich et al 2022, McDonagh et al 2021). Participants randomized to the investigational treatment XXB750 may potentially benefit from improved augmentation of the natriuretic peptide system.

During the total study duration of 24 weeks, a total of 8 in-person visits will be conducted at the site, with 3 of the visits lasting up to several hours. Study visit procedures include blood draws to obtain blood samples totally approximately 183 mL to 207 mL over the duration of the study, depending on which sub-studies the patient participates in. Patients included in this study are expected to have no background illnesses that will prevent them from producing sufficient new blood to compensate for the blood obtained for the study over its duration. Some patients may experience bruising from the phlebotomy needed in obtaining the blood samples. Study participants may also experience some discomfort associated with some study procedures, such as blood pressure measurements and urine sample collection.

Due to the mode of action of XXB750 and other background medications the patient may be taking for HF and other comorbidities, there is a risk of occurrence of hypotension and hypotension-related symptoms, such as dizziness, lightheadedness, orthostasis, postural symptoms, pre-syncope or syncope. Reflex rise in heart rate has also been reported with XXB750. In patients randomized to XXB750 the risk of symptomatic hypotension is greatest in the first few days after dosing but can potentially happen at any time during the study. Participants will receive a blood-pressure monitoring device to enable BP assessments at home as needed and per the investigator's request in the event they experience potential hypotension-related AEs. Additionally, participants will be contacted 2-3 days after each dose administration via a scheduled telephone call to monitor their tolerability and inquire about their general well-being. The current protocol includes a guidance on how to address symptomatic hypotension in the context of this study (Section 10.7).

All injections of XXB750 and its matching placebo will be administered during site visits as single SC injections of 1.6 mL, which may cause injection site reactions or bruising. Like many monoclonal antibodies, XXB750 may result in formation of anti-drug antibodies (ADA). Although available data on ADA formation do not indicate that they result in any safety problems or interfere with the PK or PD of XXB750, they will be studied carefully in this study and other ongoing XXB750 studies to better understand their significance. Hypersensitivity reactions are a potential risk for XXB750, although it has not been observed so far in XXB750 clinical studies. Hypersensitivity occurrence and its clinical significance will be monitored during the current the study. Participants will be monitored at the site for a minimum of 20 minutes after each injection. For additional information on XXB750 and its available safety information, please refer to the latest edition of the XXB750 investigator's brochure.

Women of child-bearing potential must be informed that taking the study treatment may involve unknown risks to the fetus if pregnancy were to occur during the study and agree that in order to participate in the study, they must adhere to the contraception requirements outlined in the exclusion criteria. If there is any question that the participant will not reliably comply with the recommendations of contraception, they should not be entered or continue in the study. In general, the levels of a monoclonal antibody (mAb) in semen are expected to be low, and therefore, the subsequent levels in a female partner would be negligible. Based on this principle, the probability that XXB750 would cause teratogenicity is very low. Therefore, no restrictions will be placed on male participants with regard to sexual activity or condom use.

Patients randomized to receive open-label sacubitril/valsartan in place of their background ACEI/ARB may experience hypotension/hypotension-related side effects. They may also experience renal dysfunction and hyperkalemia, although the latest published evidence indicates that sacubitril/valsartan results in a lower incidence of these side effects that ACEIs/ARBs. Angioedema has also been reported with sacubitril/valsartan. The risk of angioedema will be minimized by observing a 36-hour ACEI- and sacubitril/valsartan-free washout period whenever patients are switched from an ACEI to open-label sacubitril/valsartan (such as at the start of the randomized treatment period and the start of the safety follow-up period, as applicable). Also, patients with history of angioedema will be excluded from the trial. For more potential information and side effects of sacubitril/valsartan, please refer to your locally approved sacubitril/valsartan label.

The risk to participants in the study will be minimized by compliance with the eligibility criteria, close clinical monitoring, including home BP measurement and a follow-up call after each injection of study drug. Eligible participants with eGFR $\geq$ 30 ml/min/1.73m^2 will enter the study with close monitoring of renal function and serum potassium at every scheduled study site visit to ensure their safety. All participants will be carefully monitored by the investigators for satisfactory risk-to-benefit ratio.

A periodic review of the safety data by an independent Data Monitoring Committee (DMC) will further enhance safety for the study participants. Additionally, enrollment of participants to treatment arms with doses of XXB750 higher than 120 mg will only start after the benefit-risk balance of XXB750 administration is deemed to be acceptable by the DMC. This will be evaluated in an interim safety analysis that includes data of the lower dose arms from this study along with additional data from the ongoing phase 1b study administrating XXB750 up to 240 mg in patients with HF.

Should a participant develop clinically significant AEs during the course of the study, the blinded study treatment may, as per the investigator's discretion, be temporarily or permanently discontinued to minimize the risk to participants, and they will be managed by the treating physicians according to local standard of care.

Given the close monitoring, criteria for patient selection, and the known safety and tolerability profile of the drug in humans, it is considered that overall risks for participants in this study are acceptable.

3 Objectives, endpoints, and estimands

Table 3-1 Objectives and related endpoints

Objective(s)	Endpoint(s)
**Primary objective(s)**	**Endpoint(s) for primary objective(s)**
• To evaluate the efficacy and dose-response relationship of three XXB750 target dose levels compared to placebo in reducing NT-proBNP from baseline to Week 16 in symptomatic HF patients with LVEF < 50% treated with standard of care, including ACEI/ARB or sacubitril/valsartan.	• Change in log NT-proBNP from baseline to Week 16
**Secondary objective(s)**	**Endpoint(s) for secondary objective(s)**
• To evaluate the treatment effect of the highest XXB750 target dose level compared to placebo in reducing NT-proBNP from baseline to Week 16 in symptomatic HF patients with LVEF < 50% treated with standard of care, including ACEI/ARB or sacubitril/valsartan.	• Change in log NT-proBNP from baseline to Week 16
• To evaluate the treatment effect of combined two highest XXB750 target dose levels administered in addition to a background of ACEI/ARB versus conversion from ACEI/ARB to sacubitril/valsartan, in reducing NT-proBNP from baseline to Week 16.*	• Change in log NT-proBNP from baseline to Week 16
• To evaluate the safety and tolerability of XXB750 up-titration regimens and dose levels.	• Adverse events (incidence and severity, including but not limited to, hypotension, tachycardia, bradycardia, hypersensitivity and injection site reactions), vital signs (blood pressure, pulse), safety laboratory tests, and ECG parameters from baseline to end of study (EOS).
**Exploratory objective(s)**	**Endpoint(s) for exploratory objective(s)**
• To evaluate the effects of highest XXB750 target dose level compared to placebo in improving a composite hierarchical outcome of: 1) time to all-cause death, 2) number of HF hospitalizations, 3) Kansas City Cardiomyopathy Questionnaire (KCCQ) clinical summary score (CSS) categorical change, 4) NYHA class change , 5) relative change in NT-proBNP levels between baseline and Week 16.	• Hierarchically ordered composite consisting of: 1) time to all-cause death up to Week 16; 2) number of HF hospitalizations up to Week 16; 3) categorical KCCQ CSS change from baseline to Week 16; 4) NYHA class change from baseline to Week 16; 5) relative change in NT-proBNP levels from baseline to Week 16.
• To evaluate changes in each domain of the KCCQ, including clinical summary score (CSS) and overall summary score (OSS), from baseline to Week 16.	• Change in all domains of the KCCQ from baseline to Week 16.
• To evaluate the effects of XXB750 on slowing the rate of decline in estimated glomerular filtration rate (eGFR) from baseline to Week 16.	• Estimated glomerular filtration rate (eGFR) at scheduled assessment visits.

Objective(s)	Endpoint(s)
• To evaluate the change in biomarkers related to heart failure, cardiovascular disease and renal function from baseline to Week 8 and to Week 16.	• Change from baseline in biomarkers including, but not limited to, plasma and urinary cGMP, urinary cGMP-to-creatinine ratio, urinary albumin-to-creatinine ratio, BNP and hs-Troponin, at Week 8 and Week 16.
• To evaluate the change in NYHA class and in signs and symptoms of heart failure from baseline to Week 16.	• Change in NYHA class and signs and symptoms of HF from baseline to Week 16.
• To explore the population pharmacokinetic (PK) properties of XXB750 in HF patients.	• Population PK analysis results including mathematic models and parameters.
• To evaluate immunogenicity (IG) of XXB750.	• Detection of immune response markers, including anti-drug antibodies.

*In the event treatment arms 4a and 4b are eliminated (see Section 4.6), the second secondary objective will evaluate only the 120 mg XXB750 arm to sacubitril/valsartan arm.

3.1 Primary estimands

The primary clinical question of interest is:

Is there a dose-response signal, and if so, what is the dose-response relationship among three XXB750 target dose levels and placebo in reducing NT-proBNP from baseline to Week 16 in participants with HF (NYHA classes II-III) and LVEF < 50%, treated with standard of care, including ACEI/ARB or sacubitril/valsartan, regardless of type of baseline background HF therapy, discontinuation from the study treatment, change in the dose of concomitant medications and receiving prohibited concomitant medication?

The justification for the primary estimand is that it will capture both the effects of XXB750 and the effects of changes in other HF medications. This will provide a clear assessment of the XXB750 treatment effect in clinical practice versus placebo.

The primary estimand is described by the following attributes:

1. Population: participants with HFrEF and HFmrEF (LVEF < 50%), NYHA classes II-III and elevated NT-proBNP levels at screening while taking optimal guideline-directed HF therapy.
2. Variable: change in log NT-proBNP from baseline to 16 weeks
3. Treatment of interest: Treatment groups with three XXB750 target dose levels, and placebo all administered on top of ACEI/ARB or sacubitril/valsartan and other guideline-recommended standard of care therapy for HF.

Handling of intercurrent events:

- Discontinuation of study treatment: ignore (treatment policy strategy)
- Change in concomitant medications: ignore (treatment policy strategy)
- Receiving prohibited concomitant medication: ignore (treatment policy strategy)

4. The summary measure: difference in variable means between treatments. Since the variable in attribute 2 is equivalent to the log of ratio of Week 16 NT-proBNP over baseline NT-proBNP, and the difference in variable means between 2 treatment groups is the log of ratio of ratio to baseline of the 2 groups, therefore, the summary measure can be

back-transformed by exponentiation and expressed as ratio of geometric mean of ratio to baseline of the 2 treatment groups.

3.2 Secondary estimands

Treatment effect of the highest XXB750 target dose level compared to placebo in reducing NT-proBNP from baseline at Week 16 in HF patients treated with standard of care, including ACEI/ARB or sacubitril/valsartan

The first secondary clinical question of interest is:

What is the effect of the highest XXB750 dose level versus placebo in reducing NT-proBNP level from baseline to Week 16 in participants with heart failure (NYHA classes II-III) and LVEF < 50%, treated with standard of care, including ACEI/ARB or sacubitril/valsartan, regardless of type of baseline background HF therapy, discontinuation from study treatment, change in the dose of concomitant medications, and receiving prohibited concomitant medication?

The justification for the first secondary estimand is that it will capture both the effects of study medication and the effects of changes in guideline-directed concomitant medications. This will provide a clear assessment of the XXB750 treatment effect in clinical practice versus placebo.

The attributes and handling of intercurrent events are the same as those described in primary estimand except that the treatment of interest being the highest XXB750 dose level and placebo, instead of all XXB750 dose levels as described below:

Highest XXB750 dose level versus placebo in combination with guideline-recommended standard of care therapy for HF.

Treatment effect of combined two highest XXB750 target dose levels in addition to a background of ACEI/ARB versus conversion from ACEI/ARB to sacubitril/valsartan, in reducing NT-proBNP from baseline to Week 16

The clinical question of interest for this objective is:

What is the effect of combined two highest XXB750 dose groups in addition to a background of ACEI/ARB versus conversion from ACEI/ARB to sacubitril/valsartan in reducing NT-proBNP level from baseline to Week 16 in participants with heart failure (NYHA classes II-III) and LVEF < 50% regardless of type of baseline background HF therapy, discontinuation from the study treatment, change in the dose of concomitant medications and receiving prohibited concomitant medication?

The attributes and handling of intercurrent events are the same as those described in primary estimand except that:

- Population: participants with HFrEF and HFmrEF (LVEF < 50%), NYHA classes II-III and elevated NT-proBNP levels and on ACEI/ARB at screening while taking other guideline-directed HF therapy.
- Treatment of interest: two highest XXB750 dose levels in addition to ACEI/ARB background medication versus conversion from ACEI/ARB to sacubitril/valsartan in

combination with guideline-recommended standard of care therapy for HF. In the event treatment arms 4a and 4b are eliminated (see Section 4.6), treatment of interest will be only the 120 mg dose XXB750 dose level in addition to ACEI/ARB background medication versus conversion from ACEI/ARB to sacubitril/valsartan in combination with guideline-recommended standard of care therapy of HF.

Evaluation of the safety and tolerability of XXB750 up-titration regimens and target dose levels

Adverse events (AEs) and serious AEs, laboratory parameters and vital signs will be evaluated and summarized in each treatment group for different treatment periods (randomized treatment period and safety follow-up period). Please refer to Section 9.4.2 for analysis details.

4 Study design

4.1 Overall design

Study CXXB750A12201 is a multicenter, randomized, placebo- and active-controlled, parallel-group phase 2 study which is comprised of three periods (Figure 1-1):

- A screening period of approximately 7 days
- A 16-week parallel-group randomized treatment period on either
  i) double-blind placebo-controlled treatment (XXB750 vs. placebo) or
  ii) open-label treatment (sacubitril/valsartan)
- An 8-week safety follow-up period

The study will randomize adult participants with LVEF < 50% receiving ACEI/ARB/ARNI and guideline-recommended HF therapies for HFrEF or HFmrEF to three XXB750 target dose levels; a cohort of patients treated with ACEI/ARB before the study will be randomized to be converted to open-label sacubitril/valsartan in place of their pre-study ACEI/ARB. The study will randomize a total of approximately 720 patients. The percentage of patients with LVEF > 40% will be limited to approximately 25% of the total sample.

Refer to Figure 1-1 for an overview of study design, and to Section 6.1.2 for details on the administered doses in the different treatment arms.

A staggered approach to enrollment will be followed in this protocol. The first group of 300 participants will be randomized to placebo (treatment arm 1), XXB750 60 mg target dose (treatment arm 2), XXB750 120 mg target dose (treatment arm 3), and conversion to sacubitril/valsartan (treatment arm 5). The independent Data monitoring Committee will complete a safety analysis of the XXB750 doses tested and if it is safe to allow the dosing of patients with XXB750 planned top dose of 240 mg (i.e., allowing enrollment in arms 4a and 4b as planned). See Section 4.6 for details on planned interim analyses and adaptive design features.

4.1.1 Screening Period

The purpose of the screening period is to assess participants' eligibility to enter into the randomized treatment period of the study. At the Screening visit, participants will be asked to

review and sign the inform consent form (ICF) before performing any study-related assessments. The inclusion and exclusion criteria will be assessed to verify the candidate's eligibility for enrollment into the study.

HF, cardiovascular (CV) and other medical history will be collected. An ECG will be performed. Protocol-specified biochemical criteria, including the required eGFR, potassium, and NT-proBNP levels will be assessed using a central laboratory. The sample obtained to assess NT-proBNP for study eligibility must be collected on the same day as performing the ECG. Concomitant medications will be recorded. In order to be eligible, participants are required to receive sacubitril/valsartan or ACEI/ARB for their HF at study entry, with minimum doses of sacubitril/valsartan 49/51 mg bid or an ACEI/ARB equivalent to enalapril 10 mg/day that have been stable/unchanged for at least 4 weeks. Table 4-1 lists total daily doses of commonly used ACEIs and ARBs with doses considered equivalent to enalapril 10 mg/day for the purpose of eligibility.

Table 4-1 Minimum required pre-study daily doses of ACEIs and ARBs

ACEIs	Minimum daily dose	ARBs	Minimum daily dose
Enalapril	10 mg	Valsartan	160 mg
Lisinopril	10 mg	Candesartan	16 mg
Benazepril	20 mg	Azilsartan	40 mg
Captopril	100 mg	Eprosartan	400 mg
Cilazapril	2.5 mg	Fimasartan	60 mg
Delapril	15 mg	Irbesartan	150 mg
Fosinopril	20 mg	Losartan	50 mg
Imidapril	10 mg	Olmesartan	10 mg
Moexipril	7.5 mg	Telmisartan	40 mg
Perindopril	4 mg		
Quinapril	20 mg		
Ramipril	5 mg		
Temocapril	2 mg		
Trandolapril	2 mg		
Zofenopril	30 mg		

Other HF medication and their doses will be recorded. If the participant is not taking other classes of HF medications that are recommended per locally applicable guidelines and available locally such as beta-blockers, MRAs and/or SGLT2 inhibitors, the investigator must provide a qualifying reason for that on the appropriate electronic case report form (eCRF). The participant will continue his/her therapy, including his/her HF medications with no change during the screening period.

Refer to Table 1-1 and Section 8.1 for a full list and details of screening procedures, respectively.

4.1.2 Randomized Treatment Period

Only participants who successfully complete the screening period by meeting all inclusion criteria and none of the exclusion criteria (refer to Section 5 for a full list of inclusion and

exclusion criteria) will be eligible for randomization and invited to return to the site to complete the procedures of the Randomization visit (Day 1) as indicated in Table 1-1.

Baseline values for vital signs, biomarkers (including NT-proBNP), PK, HF signs and symptoms (as well as NYHA class), and KCCQ questionnaire will be established at this visit.

At randomization participants will be stratified by pre-study anti-RAS treatment of ACEI/ARB vs. ARNI. Participants in the pre-study ACEI/ARB stratum (N = 320) will be randomized at a final 4:4:6:3:3:12 ratio to receive either placebo (n = 40; 12.5% of participants) or XXB750 at a target dose of 60 mg, 120 mg, 240 mg with low starting dose, or 240 mg with a high starting dose (n = 160; 50% of participants across XXB750 arms) in a double-blind fashion (all given every four weeks) or be converted to receive sacubitril/valsartan at a target dose of 97/103 mg bid (n= 120; 37.5% of participants). Participants in the pre-study ARNI stratum (N = 400) will be randomized at a final 4:4:6:3:3 ratio to receive either placebo (n= 80; 20% of participants) or XXB750 at a target dose of 60 mg, 120 mg, 240 mg with low starting dose, or 240 mg with a high starting dose (n = 320; 80% of participants across XXB750 arms) in a double-blind fashion. Thus, in participants randomized to injectable study medication (treatment arms 1,2,3,4a, 4b), it is anticipated to result in a 1:2 ratio of ACEI/ARB vs. ARNI background anti-RAS treatment. All study medications will be taken in addition to other background medications.

For participants on prior ACEI treatment and randomized to treatment arm 5 (open-label sacubitril/valsartan), an ACEI-free wash-out period of 36 hours is mandatory to minimize the potential risk of angioedema. For example, if a participant's last dose of pre-study ACEI was on Wednesday morning, he/she should be instructed to take the first dose of the open-label study sacubitril/valsartan on Thursday evening. Please refer to Section 6.1.2 for details on treatment arms, the up-titration regimens, and the doses the participant will receive at the respective visits.

Participants will undergo blood sampling and other assessments at each site visit and be contacted two days after each injection of study medication as outlined in Table 1-1 and described in Section 8.

The modification of participants background heart failure therapy should be avoided if at all possible during the randomized treatment period, which is planned to last for 16 weeks, unless it is truly required to ensure patient safety. The dosing schedule of the study medications should be adhered to as instructed by the protocol.

Please refer to Section 6.4.1 for recommendations for management of select adverse events and to Section 6.5 for guidance on dose modification of the study medication should the need arise.

4.1.3 Safety Follow-up Period

Because XXB750 has a half-life of ~16 days and might be detectable in the blood for approximately 90 days after administration of the last dose, the safety follow-up period is included in the study design to monitor the safety of participants while the study medication is tapering off. After completing the procedures of the Week 16 visit, the investigator may modify the participant’s medication regimen as necessary for the patient's HF status and as per guidelines. This may include the replacement of the oral study treatment sacubitril/valsartan with commercially available sacubitril/valsartan, ACEI, or ARB per the investigator's clinical

judgment and local and international HF treatment guidelines and according to locally approved medication label. If at the start of the safety follow-up period the investigator wishes to place patients who were previously randomized to open-label sacubitril/valsartan on an ACEI, a 36 hour sacubitril/valsartan-free washout period must be observed before the patient begins to take the ACEI in order to minimize the likelihood of occurrence of angioedema. For example, if a participant's last dose of open-label study sacubitril/valsartan is on Wednesday evening, the first dose of the ACEI should be taken on Friday morning.

Two study visits will be conducted to collect safety data, NT-proBNP, and PK laboratory assessments and to monitor the participant's overall health status (i.e., Week 20 and Week 24 visits).

Participants who prematurely discontinue the study medication should complete similar safety follow-up procedures, which should be scheduled 8 and 12 weeks after the last dose administration of XXB750 or its matching placebo, respectively.

4.2 Scientific rationale for study design

The current study aims to investigate several aspects of the efficacy and safety of XXB750 in HF patients with LVEF < 50%. The primary objective of the study aims to characterize the dose-response pattern of XXB750 when added to treatment with ACEI/ARB or sacubitril/valsartan in patients with LVEF < 50%. A secondary objective of the study aims to determine the relative efficacy of modulating the NP system via NPR1 agonism compared to modulating it via neprilysin inhibition in reducing NT-proBNP in this patient population. Moreover, since XXB750 is the first injectable long-acting NPR1 agonist, the study aims to identify an optimal dosing regimen that best balances risks with benefits of XXB750.

With all the above in mind, the current study design is considered to be optimal for accomplishing its objectives and for arriving to sound and reliable answers to the scientific questions of the study, while minimizing the burden to the participants and to the investigators and ensuring patient safety. Regarding the primary objective, i.e., the dose-response finding (DRF) aspect of the study, this is a randomized, double-blind, and placebo-controlled study. This design is typical for DRF studies as it compares the effects of three doses of XXB750 to placebo (all given SC every 4 weeks) to help form a dose-response curve with change in NT-proBNP level as the endpoint. Patients will be randomized to receive XXB750 or its matching placebo in a blinded manner on top of other background guideline-direct HF therapy. As a result, this design maximizes the chances of producing reliable and internally and externally valid results to support sound conclusions about the efficacy and safety of the experimental treatment (i.e., XXB750 in this study). A multicenter setting has been chosen to ensure adequate enrollment into the study and to include an internationally representative sample that accounts for regional and local differences in practice norms.

Based on extrapolation from data established for sacubitril/valsartan, it is expected that the full effect of NT-proBNP reduction will be achieved in all treatment groups after 8 weeks of study treatment at target dose levels. Thus, considering that target dose levels are reached at Week 8 in all treatment arms, the 16-week duration of the randomized treatment period is expected to be sufficient to examine the effect of each target dose level of XXB750 to lower NT-proBNP.

The current design is also adequate for assessing the relative effect of the investigational modality of NP modulation, i.e., NPR1 agonism with XXB750, compared to the NP modulation mechanism currently available in clinical practice, i.e., neprilysin inhibition using sacubitril, in reducing NT-proBNP. Because the only available neprilysin inhibitor is commercially delivered via a compound with valsartan, the study is designed to include sufficient ACEI/ARB-treated participants who will be randomized to receive either XXB750 at different target dose levels (i.e., NPR1 agonist + ACEI/ARB cohort) or be converted to receive open-label sacubitril/valsartan at the approved target dose in place of their ACEI/ARB (i.e., neprilysin inhibitor + ARB cohort). The open-label design of the sacubitril/valsartan cohort optimally balances the degree of burden to patients and complexity of the study, while ensuring the conclusions based on this cohort are scientifically robust. Indeed, the primary endpoint of the study is change in NT-proBNP, which is an objective measure.

To better explore the safety profile of XXB750 and the most optimal regimen of its administration, patients assigned to the highest XXB750 target dose will be further assigned to one of two up-titration regimens to be administered in a blinded manner. The regimens differ in the initial starting dose and will allow for understanding whether a slower, more gradual dosing regimen will result in improved tolerability and higher adherence rate to the target dose than a more condensed regimen that requires fewer up-titration steps.

4.2.1 Rationale for target study population

The current study will include subjects with symptomatic HF (NYHA class II-III) with LVEF < 50%, i.e., HFrEF and HFmrEF subjects, who are being treated with guideline-directed therapy. The percentage of patients with LVEF > 40% will be limited to approximately 25% of the total sample, which is in line with the reported relative prevalence of HFrEF and HFmrEF (McDonagh et al 2021). This population was chosen because it is a population that continues to have a projected unacceptably high rate of morbidity and mortality despite the latest approved treatment modalities.

Despite recently introduced recommendations for a quadruple pharmacological therapy for the treatment of HFrEF, including ARNI, beta-blockers, MRA and SGLT2 inhibitors (McDonagh et al 2021, Heidenreich et al 2022), outcomes on this comprehensive therapy are projected to leave significant room for improvement (Vaduganathan et al 2020, Tromp et al 2022). Thus, there remains an unmet need for further therapies improving mortality and morbidity in patients with HFrEF.

Treatment options for HFmrEF patients are less well-studied and, until relatively recently, recommendations for treatment of patients with HFmrEF were mostly based on post-hoc and subset analyses from HFrEF and HFpEF trials including patients with HFmrEF (Heidenreich et al 2022). More recently, the approved labels of the ARNI sacubitril/valsartan as well as of the SGLT2 inhibitors dapagliflozin and empagliflozin were expanded to include treatment of HFmrEF patients in some regions of the world. Nonetheless, a need for identifying novel treatments that will lower morbidity and mortality in HFmrEF patients remains unmet. Given that medications thought to be beneficial in HFrEF tend to also be beneficial in HFmrEF, it is deemed appropriate to study XXB750 in a combined sample of HFrEF and HFmrEF patients. Moreover, given that sacubitril/valsartan and XXB750 are both NP modulators, albeit via differing mechanisms with differing potencies, it is reasonable to

expect that XXB750 may also provide benefit in HFmrEF patients. This provides a strong rationale for the choice of studying the effects of XXB750 in patients with LVEF up to 50%.

The study will include patients who have been already treated with guideline-direct HF therapy so that the incremental benefit of the investigational treatment XXB750 will be clearly measured to better project the extent of improvements it can provide on top of the current standard of care. All eligible participants must be receiving an anti-RAS agent, i.e., an ACEI, ARB, or the ARNI sacubitril/valsartan. Participants should also be receiving beta blockers MRAs and SGLT2 inhibitors, unless contraindicated, not tolerated, not available to the patient or not recommended per local practice standards.

4.2.2 Rationale for the primary endpoint

The primary endpoint of this study is the change in log NT-proBNP from baseline to Week 16.

Aggregate clinical data has demonstrated that NT-proBNP not only provides value for the diagnosis, and helps the management of patients with HF, but also predicts clinical outcome (mortality and morbidity). Studies suggest that NT-proBNP levels strongly correlate with adverse clinical outcomes in HF patients including death and hospital admission for chronic HF (Zile et al 2016, Januzzi et al 2021, Schmitt et al 2021). More importantly, changes in natriuretic peptides were significantly associated with treatment effects on HF hospitalization across contemporary HF trials evaluating RAS inhibition (Vaduganathan et al 2018). With regard to ARNI, in a landmark analysis based on the PARADIGM-HF trial data, adjusted for baseline values, a 50% reduction in NT-proBNP levels was associated with approximately 32% reduction in the composite of CV death or HF hospitalization (Zile et al 2016).

In view of XXB750's mode of action, change of NT-proBNP is thus considered an appropriate surrogate endpoint for clinical outcomes of HF in this short-term study in patients with HF. The results will be used to determine the projected benefit of XXB750 on long-term HF outcomes when added to SoC guideline-directed HF therapy.

4.3 Justification for dose

Doses and administration regimens of XXB750 were selected based on the safety, tolerability, and pharmacodynamic profile observed in first interpretable results (FIR) from the FIH trial of XXB750 (study CXXB750A02101) and data observed in a phase 1b study in patients with HFrEF/HFmrEF (study CXXB750A12101).

Study CXXB750A02101 investigated the safety, tolerability, PK, and pharmacodynamics of single SC doses of XXB750 ranging from 1 mg to 600 mg in healthy volunteers without (1 mg, 3 mg, 10 mg, 30 mg, 60 mg, 120 mg, and 240 mg) and with elevated blood pressure (450 mg and 600 mg). The highest single tolerated dose was found to be 450 mg. There was no observed distinguishable effect on plasma cGMP or BP at doses of 1 mg, 3 mg, and 10 mg and placebo injection. XXB750 showed cGMP elevation and BP lowering effect at doses of ≥ 30 mg, which appeared to be greater than the effect of a placebo injection. For these doses, plasma cGMP elevation reached its maximum at day 2 post-dose, while it was lower than peak effect but still elevated compared to placebo at day 28 post-dose. Blood pressure lowering achieved maximum effect by day 3 and showed a gradual decline over the following weeks. At day 28 post-dose, the BP lowering effect was still present with doses ≥ 120 mg (SBP change from baseline of

~7 mmHg and ~5 mmHg for 120 mg and 240 mg dose cohorts, respectively). In the phase 1b study in HF patients with LVEF <50% receiving ACEI/ARBs and other background HF medications (cohort 1), a single XXB750 SC dose of 120 mg resulted in a maximum decrease in SBP of 7 mmHg, which occurred on day 2 post-dosing and was found to be well tolerated. Please refer to the XXB750 investigators brochure for detailed data on studies CXXB750A02101 and CXXB750A12101).

Given the sustained cGMP elevation and acceptable safety and tolerability profile, the 120 mg dose has been selected as the highest starting dose to be tested in this study. Based on pharmacometric modeling of the data from study CXXB750A02101, XXB750 doses of 60 mg, 120 mg and 240 mg are selected to be evaluated as target dose levels, to further investigate efficacy and safety of XXB750 in HF patients, and to support its use in subsequent studies as a once every 4 weeks SC administration regimen.

In the open-label sacubitril/valsartan cohort the target dose will be 97/103 mg bid, which is the target dose shown to be effective in reducing morbidity and mortality in HFrEF patients (McMurray et al 2014). The effectiveness of this dose extends to HF patients with higher LVEF with the greatest benefit occurring in patients with LVEF below normal, including patients with HFmrEF (Solomon et al 2020).

For more information about studies CXXB750A02101 and CXXB750A12101, please refer to the XXB750 Investigator's Brochure.

4.3.1 Rationale for choice of background therapy

The current study aims that all participants are treated with guideline-directed heart failure therapy as recommended per recently published guidelines (McDonagh et al 2021, Heidenreich et al 2022). To reduce variability across the studied sample and to ensure patients are treated with reasonably effective doses of background anti-RAS therapy, eligible patients must be receiving approximately half the target dose of their pre-study ACEI/ARB/ARNI or higher per international treatment guidelines and locally approved labels.

Other components of guideline-directed therapies for HFrEF and HFmrEF are recommended as background therapy at individually optimized doses, unless not tolerated or not available to patients.

Overall, these requirements will ensure that participants are treated according to current guideline recommendations including sacubitril/valsartan or an ACEI/ARB.

To avoid an influence of dose adjustments on baseline NT-proBNP levels, all background heart failure therapy must be given at a stable dose for at least 4 weeks prior to screening.

4.4 Rationale for choice of control drugs (comparator/placebo) or combination drugs

The primary objective of this study is to evaluate the dose-response effect of three dose levels of XXB750 on lowering NT-proBNP when administered on top of standard of care therapy recommended for patients with HFrEF (McDonagh et al 2021, Heidenreich et al 2022). As such, a placebo is considered the appropriate control for achieving this objective. A placebo

comparator is also appropriate for evaluating the safety and tolerability of XXB750 when given at the three dose levels.

Another objective of this study is to assess the benefit of NPR1 agonism with XXB750 on reduction in NT-proBNP relative to neprilysin inhibition. Thus, sacubitril/valsartan, a compound that delivers neprilysin inhibition and angiotensin receptor blockade, is the only clinically available and appropriate control. To ensure this objective is properly achieved, a sufficient number of ACEI/ARB treated participants will be randomized to either receive XXB750 while continuing to take their ACEI/ARB (i.e., receive NPR1 agonism + anti-RAS treatment in treatment arms 2, 3, 4a and 4b) or stop their pre-study ACEI/ARB and be converted to sacubitril/valsartan (i.e., receive neprilysin inhibition + anti-RAS treatment in treatment arm 5).

4.5 Rationale for public health emergency mitigation procedures

During a public health emergency as declared by local or regional authorities e.g., pandemic, epidemic, or natural disaster, mitigation procedures to ensure participant safety and trial integrity may be implemented. Notification of the public health emergency as declared by local or regional authorities should be discussed among investigators and Novartis. All procedures adapted to the situation must be submitted, if required as per local regulations, through a protocol amendment for approval by local or regional Health Authorities and Ethics Committees prior to implementation of mitigation procedures.

4.6 Purpose and timing of interim analyses/design adaptations

Limited safety data is currently available for XXB750, and it is thus prudent to dose the medication cautiously in participants until more is learned about its side effects.

Staggered enrollment and the safety interim analysis

To help guard the safety of study participants, randomization will initially exclude the planned highest target dose of XXB750 (i.e., arms 4a and 4b) until an interim analysis is conducted to evaluate the safety of the lower two target doses and that a sufficient safety margin exists to allow patients to be dosed with the highest target dose arms.

The randomized participant sample will be divided into two groups. Group 1 will consist of the first approximately 300 participants who will be randomized to receive placebo, XXB750 target dose of 60 mg every 4 weeks, XXB750 target dose of 120 mg every 4 weeks, or sacubitril/valsartan at a target dose of 97/103 mg bid in a 1:1:2:1 ratio (see Table 4-2).

Group 2 will consist of the remaining approximately 420 participants who are planned to be randomized to receive placebo, XXB750 target dose of 60 mg q 4 weeks, XXB750 target dose of 120 mg q 4 weeks, XXB750 target dose of 240 mg q 4 weeks at a starting dose of 60 mg, XXB750 target dose of 240 mg at a starting dose of 120 mg, or sacubitril/valsartan 97/103 mg bid in a 2:2:2:3:3:2 ratio (see Table 4-2).

The DMC will conduct an early safety interim analysis of the data of Group 1 when approximately 85 participants have completed at least 9 weeks of randomized treatment to assess the safety profile of repeat doses of XXB750 up to 120 mg in the study participants.

Additionally, safety data from cohort 2 of the ongoing XXB750 phase 1b study CXXB750A12101 (i.e., safety profile of two XXB750 120 mg doses followed by one 240 mg dose all given 4 weeks apart and followed for 12 weeks after the last dose) will be reviewed to provide further insight into the tolerability of the planned 240 mg dose of XXB750 in HF patients. If the totality of available safety evidence is deemed acceptable by the DMC to allow for dosing of XXB750 at doses of 240 mg, enrollment of Group 2 will start once Group 1 enrollment is completed.

In addition to the planned interim safety analysis summarized above, there will be safety data reviews conducted regularly by the DMC approximately twice annually. Refer to Section 10.1.4 for more information about the role of the DMC in this study.

Table 4-2 Overview of the planned staggered enrollment strategy

Treatment arm	Target dose, up-titration scheme	Group 1 n	Group 2 n	Total n
1	Placebo	60	60	120
2	XXB750 60 mg	60	60	120
3	XXB750 120 mg	120	60	180
4a*	XXB750 240 mg, initial dose 60 mg	0	90	90
4b*	XXB750 240 mg, initial dose 120 mg	0	90	90
5	Sacubitril/valsartan 97/103 mg bid	60	60	120
Total		300	420	720

*Highest target XXB750 dose may potentially be changed to be 180 mg. Refer to the adaptive design features section below for details.

Adaptive design features

If at the DMC early safety review described above, it is deemed inappropriate or unadvisable to continue the study as planned, i.e., to allow patients to be randomized to as planned into groups 4a and 4b at a target dose of 240 mg every 4 weeks, one of the following adaptations to the design of the study may be implemented per the recommendation of the DMC:

1. Change the highest target dose in the study from the planned XXB750 240 mg every 4 weeks to 180 mg every 4 weeks.
   In Group 2, participants randomized to treatment arm 4a will receive XXB750 60 mg SC at the Randomization followed by XXB750 120 mg SC at Week 4 and XXB750 180 mg SC at Week 8 and Week 12. Participants randomized to treatment arm 4b will receive XXB750 120 mg SC at the Randomization followed by XXB750 180 mg SC at Week 4, Week 8, and Week 12. The planned dosing schedule of all other treatment arms will remain unmodified. The sample size allotted to all treatment arms will remain unchanged.
2. Eliminate treatment arm 4b (top dose either XXB750 240 mg or 180 mg every 4 weeks). In this case the highest target dose will be reached after a starting dose of 60 mg (treatment arm 4a). In Group 2, all participants previously allocated to treatment arm 4b will be reallocated to treatment 4a. Thus, the 420 participants in Group 2 will be randomized to receive placebo, XXB750 target dose of 60 mg every 4 weeks, XXB750 target dose of 120 mg every 4 weeks, XXB750 target dose of 240 mg (or 180 mg) every 4

weeks at a starting dose of 60 mg, or sacubitril/valsartan 97/103 mg bid in a 1:1:1:3:1 ratio.

3. Eliminate treatment arms 4a and 4b.
   In this case the highest target dose in the study will be XXB750 120 mg every 4 weeks (treatment arm 3) and 180 participants will be allocated to treatment arm 3 from Group 2 for a total/overall sample size of 300 participants in this arm. Thus, Group 2 will include 360 participants who will be randomized to receive placebo, XXB750 target dose of 60 mg every 4 weeks, XXB750 target dose of 120 mg every 4 weeks, or sacubitril/valsartan 97/103 mg bid in a 1:1:3:1 ratio. The planned dosing schedule of these treatment arms will remain unmodified. In this case the total sample size of the study will be approximately 660 participants.

Participating investigators will be informed promptly of any of the adaptations to the design of the study shown above that are to be implemented in consultation with the DMC, so that they can inform the participants and ethics committees/IRBs as required by local regulations. The sponsor will inform the local health authorities as per local regulations.

Administrative interim analysis informing other programs

An additional administrative interim analysis to inform other development programs and studies may be conducted when approximately 430 participants are randomized into the study (at least 40 participants in each of arms 1-4) and are followed for at least 16 weeks. The results of this administrative interim analysis will not lead to any changes in this study and its results will be kept blinded for anyone who is directly involved in the conduct of this study. More details about the DMC review process and how unblinding will be handled in the context of DMC reviews will be provided in the DMC charter.

4.7 End of study definition

The end of the study is defined as the date of the last visit of the last participant in the study globally.

Study completion is defined as when the last participant finishes his/her Week 16 visit and any repeat assessments associated with this study visit have been documented and followed-up appropriately by the Investigator.

All randomized participants will have a safety follow-up after last administration of study treatment as outlined in Section 1.3 SoA and Section 4.1.3. Serious adverse event (SAE) reporting continues during this time period as described in Section 8.6.3. Documentation of attempts to contact the participant are required to be recorded in the source documentation.

5 Study population

The study population will consist of male and female participants 18 years old or older with symptomatic HFrEF or HFmrEF as defined in the inclusion criteria. A total of approximately 720 participants will be randomized into this study. The percentage of patients with LVEF > 40% will be limited to approximately 25% of the total sample.

5.1 Inclusion criteria

Participants eligible for inclusion in this study must meet **all** of the following criteria:

1. Signed informed consent must be obtained prior to participation in the study.
2. Male and female outpatients who are ≥ 18 years old at screening.
3. Current symptom(s) of HF NYHA class II-III.
4. LVEF < 50% (any local measurement, made within the past 6 months prior to or during screening using echocardiography, MUGA, CT scanning, MRI or ventricular angiography is acceptable, provided there is no subsequent measurement ≥ 50%).
5. NT-proBNP ≥ 600 pg/mL if in sinus rhythm or
   NT-proBNP ≥ 900 pg/ml if in atrial fibrillation/flutter at screening.
6. Receiving an ACEI or an ARB at a stable dose of at least enalapril 10 mg/d or equivalent for at least 4 weeks before screening or receiving sacubitril/valsartan at a stable dose of at least 49/51 mg bid for at least 4 weeks before screening.
7. Receiving other guideline recommended HF therapies as deemed appropriate by the investigator and that are stable in dose for at least 4 weeks before screening, unless contraindicated, not tolerated, or not available to patient. Other evidence-based therapy for heart failure should also be considered, e.g., cardiac resynchronization therapy (CRT) and an implantable cardioverter-defibrillator in selected patients, as recommended by guidelines.

5.2 Exclusion criteria

Participants meeting **any** of the following criteria are **not** eligible for inclusion in this study.

1. Current acute decompensated HF (exacerbation of chronic HF manifested by signs and symptoms that may require intravenous therapy) or hospitalization for HF within 3 months prior to screening.
2. Current symptomatic hypotension (for example dizziness/presyncope).
3. Office systolic blood pressure (SBP) ≥ 180 mmHg or < 105 mmHg at screening or at randomization.
4. History of hypersensitivity to XXB750, sacubitril/valsartan, or any of the study drug's excipients or to any other biological drugs.
5. Received a monoclonal antibody or immunoglobulin-based agent within 1 year of screening.
6. In subjects with ACEI/ARB medication at screening, previous inability to tolerate any dose of sacubitril/valsartan or any ARB (as per the investigator's judgment).
7. Known history of angioedema (as per the investigator's judgment).
8. Serum potassium > 5.4 mmol/L (or equivalent plasma potassium value) at screening.
9. Estimated GFR < 30 mL/min/1.73m^2 as measured by the CKD-EPI formula at screening.
10. Presence of known functionally significant bilateral renal artery stenosis at screening.
11. Evidence of urinary obstruction, or difficulty in voiding at screening, or of congenital renal abnormalities with known effect on renal function.
12. Clinically significant congenital heart disease that could be the cause of the patient’s symptoms and signs of HF.

13. Presence of hemodynamically significant valvular heart disease other than functional mitral insufficiency or known right ventricular dysfunction that is deemed to increase susceptibility to the hypotensive effects of therapy in the opinion of the investigator.
14. Clinically significant cardiac arrhythmias (e.g., ventricular tachycardia), high-grade atrio-ventricular (AV) block (e.g., Mobitz type II and third-degree AV block in the absence of a pacemaker) or symptomatic bradycardia within 6 months of screening according to the investigator's judgement.
15. Acute myocardial infarction (AMI) or unstable angina, any history of ischemic or hemorrhagic stroke, or any percutaneous coronary intervention (PCI) or coronary artery bypass graft (CABG) within 6 months of screening, or CRT implantation within 3 months of screening.
16. History or current diagnosis of severe pulmonary disease (e.g. COPD) requiring chronic supplemental oxygen, corticosteroid, or nebulizer therapy or pulmonary arterial hypertension (WHO group 1 or 4) requiring pharmacology treatment at screening.
17. At screening, symptomatic anemia or hemoglobin < 10 g/dL in male subjects or < 9 g/dL in female subjects.
18. Prior major organ transplant, intention to transplant (i.e. on transplant list) or planned/anticipated major cardiac surgery during the involvement of the participant in the study.
19. History or presence of any other disease where the life expectancy is less than 3 years.
20. History of malignancy of any organ system (other than localized basal or squamous cell carcinoma of the skin or localized prostate cancer), treated or untreated, within the past 3 years, regardless of whether there is evidence of local recurrence or metastases.
21. Evidence of hepatic disease as determined by any one of the following: aspartate aminotransferase (AST; serum glutamic-oxaloacetic transaminase, SGOT) or alanine aminotransferase (ALT; serum glutamic-pyruvic transaminase, SGPT) values exceeding 3x the upper limit of normal (ULN), or total bilirubin (TBL) > 1.5 mg/dL at screening.
22. History or current diagnosis of drug abuse or alcohol dependency.
23. Lacking the ability to comprehend or follow instructions, or for any reason in the opinion of the investigator, a patient that would be unlikely or unable to comply with study protocol.
24. Concurrent enrollment in any other investigational drug or device trial (participation in non-interventional registries is acceptable).
25. Use of other investigational drugs at the time of enrollment, or within 30 days or 5 half-lives of enrollment, whichever is longer.
26. Pregnant, nursing or planning to become pregnant (documented negative pregnancy test required within a maximum of 7 days prior to enrollment of all women of childbearing potential). Documentation of highly effective contraception is also required for women of childbearing potential (see below).

    Women of child-bearing potential are defined as all women physiologically capable of becoming pregnant, **unless** they are using highly effective methods of contraception while taking study treatment and for 3 months after last double-blind injectable study treatment

or for 7 days after the last dose of oral study treatment (if randomized to receive open-label sacubitril/valsartan). Highly effective contraception methods include:

- Total abstinence (when this is in line with the preferred and usual lifestyle of the participant). Periodic abstinence (e.g. calendar, ovulation, symptom-thermal, post-ovulation methods) and withdrawal are not acceptable methods of contraception.
- Female sterilization (have had surgical bilateral oophorectomy with or without hysterectomy, total hysterectomy, bilateral salpingectomy, or bilateral tubal ligation) at least six weeks before taking study treatment. In case of oophorectomy alone, only when the reproductive status of the woman has been confirmed by follow-up hormone level assessment.
- Male sterilization (vasectomy) of the male partner(s) of the female participant (at least 6 months prior to screening). For female participants on the study, the vasectomized male partner(s) should be the sole partner(s) for that participant.
- Use of oral, (estrogen and progesterone), injected, or implanted hormonal methods of contraception or placement of an intrauterine device (IUD) or intrauterine system (IUS), or other forms of hormonal contraception that have comparable efficacy (failure rate < 1%), for example hormone vaginal ring or transdermal hormone contraception.

In case of use of oral contraception, women should have been stable on the same pill for a minimum of 3 months before taking study treatment.

Women are considered post-menopausal if they have had 12 months of natural (spontaneous) amenorrhea with an appropriate clinical profile (e.g., age-appropriate history of vasomotor symptoms). Women are considered not of childbearing potential if they are post-menopausal or have had surgical bilateral oophorectomy (with or without hysterectomy), total hysterectomy, bilateral salpingectomy or bilateral tubal ligation at least six weeks before enrollment on study. In the case of oophorectomy alone, only when the reproductive status of the woman has been confirmed by follow up hormone level assessment is not she considered to be of childbearing potential.

If local regulations deviate from the contraception methods listed above to prevent pregnancy, local regulations apply and will be described in the ICF.

5.3 Screen failures

A screen failure occurs when a participant who consents to participate in the clinical study is subsequently found to be ineligible and therefore does not enter the Randomized treatment period of the study. A minimal set of screen failure information is required to ensure transparent reporting of screen failure participants to meet the Consolidated Standards of Reporting Trials (CONSORT) publishing requirements and to respond to queries from regulatory authorities. Minimal information includes demography, reason(s) for screen failure, eligibility criteria, adverse events related to AxMPs and any SAE.

The reason for screen failure should be recorded on the appropriate CRF. The demographic information, informed consent, and inclusion/exclusion pages must also be completed for screen failure participants. Screening laboratory assessments will also be collected and entered into the clinical database. No other data will be entered into the clinical database for participants who are screen failures, unless the participant experienced a serious adverse event during the

screening period (see Section 8.6.3 for SAE reporting details). If the participant fails to be randomized, the IWRT (Interactive Web Response Technology)/IRT (Interactive Response Technology) system must be updated with the screen fail that the participant will not be randomized. Data and samples collected from participants prior to screen failure may still be analyzed.

Individuals who do not meet the criteria for participation in this study (screen failure), will have one opportunity to be re-screened again for this study, ≥ 2 weeks after the original screening date.

5.3.1 Replacement policy

Not applicable.

5.3.2 Participant numbering

Each participant is identified in the study by a Participant Number (Participant No.), that is assigned when the participant is enrolled for screening and is retained for the participant throughout his/her participation in the trial. The Participant No. consists of the Site Number (Site No.) (as assigned by Novartis to the investigative site) with a sequential participant number suffixed to it, so that each participant's participation is numbered uniquely across the entire database. Upon signing the informed consent form, the participant is assigned to the next sequential Participant No. available.

A new ICF must be signed if the investigator chooses to re-screen the participant after a participant has screen failed, and the participant will be assigned a new Participant Number. Additional details regarding Screening are provided in Section 8.1.

6 Study treatment(s) and concomitant therapy

6.1 Study treatment(s)

Study treatment includes investigational drugs XXB750, its matching placebo, and sacubitril/valsartan.

Oral study medication refers to sacubitril/valsartan as investigational/control drug.

XXB750 or matching placebo will be administered as a SC injection every 4 weeks during the randomized treatment period (Randomization, Week 4, Week 8 and Week 12). Table 6-1 lists investigational and control drug.

Table 6-1 Investigational and control drug

**Treatment Title**	XXB750 150 mg/mL	XXB750 0 mg/mL (placebo)	Sacubitril/valsartan
**Treatment Description**	Liquid in Vial	Liquid in Vial	Solid
**Type**	Biologic	Placebo	Drug
**Dose Formulation**	Concentrate for solution for injection	Solution for injection	Tablet
**Unit Dose Strength(s)**	150 mg/ml	0 mg/ml	97/103 mg, 49/51 mg and 24/26 mg

**Dosage Level(s)**	as per Table 6-3	as per Table 6-3	as per Table 6-3
**Route of Administration**	Subcutaneous use	Subcutaneous use	Oral
**Use**	Experimental	Placebo	Active comparator
**IMP**	Yes	Yes	Yes
**Sourcing**	Novartis (global)	Novartis (global)	Provided locally
**Packaging and Labeling**	Study treatment will be provided in vials. Each vial will be labeled as per country requirement. Open-label packs	Study treatment will be provided in vials. Each vial will be labeled as per country requirement. Open-label packs	As per local packaging. Each package will be labeled as per country requirement. Open-label packs

6.1.1 Additional study treatments

Auxiliary medicinal products (under EU CTR) in this trial are ACEI, ARB and sacubitril/valsartan. When participants enter the randomized treatment period, they will remain on the same background ACEI or ARB or on sacubitril/valsartan (as applicable) they had been receiving for their usual care before randomization without change in dose, source, or type of ACEI/ARB/ARNI (per local requirements). Exception is made to those participants that are randomized to arm 5, who will be switched from their pre-study ACEI or ARB to open-label sacubitril/valsartan, which will be provided by Novartis during the 16 weeks randomized treatment period.

Table 6-2 Auxiliary treatments

Treatment Title	ACEI	ARB	Sacubitril/valsartan
**Treatment Description**	Once or twice daily	Once or twice daily	Twice daily
**Type**	Drug	Drug	Drug
**Dose Formulation**	Tablet	Tablet	Tablet
**Route of Administration**	Oral	Oral	Oral
**Use**	Background medication	Background medication	Background medication
**Authorization status of the AMP in EEA**	Yes	Yes	Yes
**Sourcing**	Provided locally as per country requirement	Provided locally as per country requirement	Provided locally as per country requirement
**Packaging and Labeling**	Auxiliary treatment will be provided in commercially available packaging.	Auxiliary treatment will be provided in commercially available packaging.	Auxiliary treatment will be provided in commercially available packaging.

6.1.2 Treatment arms/group

Table 6-3 summarizes planned study treatment arms and their dosing schedule. Please refer to Section 4.6 for potential adaptive design features that may potentially affect the treatment arms at the DMC early safety review.

Table 6-3 Treatment arms, dose levels, and planned doses administered at dispensing visits during the Randomized treatment period

<table>
<tr><th>Treatment arm</th><th>Target dose level</th><th>Titration scheme</th><th>Rando-mization</th><th>Week 2</th><th>Week 4</th><th>Week 8</th><th>Week 12</th></tr>
<tr><td>**Arm 1**</td><td>**XXB750 placebo**</td><td></td><td>Placebo</td><td></td><td>Placebo</td><td>Placebo</td><td>Placebo</td></tr>
<tr><td>**Arm 2**</td><td>**XXB750 60 mg**</td><td></td><td>60 mg</td><td></td><td>60 mg</td><td>60 mg</td><td>60 mg</td></tr>
<tr><td>**Arm 3**</td><td>**XXB750 120 mg**</td><td></td><td>60 mg</td><td></td><td>120 mg</td><td>120 mg</td><td>120 mg</td></tr>
<tr><td rowspan="2">**Arm 4**</td><td rowspan="2">**XXB750 240 mg***</td><td>**4a**</td><td>60 mg</td><td></td><td>120 mg</td><td rowspan="2">240 mg*</td><td rowspan="2">240 mg*</td></tr>
<tr><td>**4b**</td><td>120 mg</td><td></td><td>240 mg*</td></tr>
<tr><td rowspan="2">**Arm 5**</td><td rowspan="2">**Sacubitril/valsartan open-label, 97/103 mg bid**</td><td rowspan="2">Starting dose as per label and investigator's judgment</td><td>49/51 mg bid</td><td rowspan="2">49/51 mg bid</td><td rowspan="2">97/103 mg bid</td><td rowspan="2">97/103 mg bid</td><td rowspan="2">97/103 mg bid</td></tr>
<tr><td>24/26 mg bid</td></tr>
</table>

* May be lowered to a target dose of 180 mg, determined after interim safety analysis

In treatment arms 1 through 4, all patients will receive SC injections of XXB750 or matching placebo. Refer to Section 6.5 for instructions on adjusting the dose of XXB750 in the context of management of adverse events.

In treatment arm 5, restricted to patients on ACEI/ARB, the pre-study ACEI/ARB medication will be discontinued and replaced by open-label sacubitril/valsartan. Participants on pre-study ACEIs must be instructed to observe an ACEI-free washout wash-out period of 36 hours is mandatory prior to the first dose of open-label sacubitril/valsartan to minimize the potential risk of angioedema. Refer to Section 4.1.2 for a description of how to implement the ACEI-free washout.

Patients randomized to open-label sacubitril/valsartan may be initiated on 24/26 mg to 49/51 mg bid per the investigator's clinical judgment (taking into account the pre-study ACEI/ARB dose) and must be gradually force-titrated to the target dose of 97/103 mg bid by Week 4. Every attempt should be made to maximize the dose of the study open-label sacubitril/valsartan by modifying other background medications, such as diuretics, nitrates, and calcium channel blockers, as per the investigator's clinical judgment. The dose of open-label sacubitril/valsartan may be down-titrated if the participant experiences dose-limiting side effects that cannot be managed by modifications of the patient's concomitant medications.

6.1.3 Treatment duration

The planned duration of randomized treatment is 16 weeks. After the 16-week randomized treatment period, participants will enter an 8-week safety follow-up period in which participants may be treated at the discretion of the investigator taking into account that their heart failure clinical status may still be affected by the study treatment for some time after its discontinuation.

6.2 Preparation, handling, storage, and accountability

Each study site will be supplied with study treatment as described under Section 6.1 in Table 6-1 (investigational and control drugs).

A unique medication number is printed on the study medication label (applicable only for globally sourced investigational and control drug, i.e. XXB750 or matching placebo).

Unblinded qualified and properly trained members of the study team at the site will identify the IMP kits to be administered or be dispensed to the participants by contacting the IRT system and obtaining the medication number(s). Drug accountability and reconciliation data are recorded in the IRT system.

For locally sourced open-label oral study medication, according to the treatment assigned to the participant, Investigator staff will select the study medication to be dispensed to the participant. Site personnel will record the locally sourced supply dispensation into the participant's source document.

As per Section 4.5, during a public health emergency as declared by local or regional authorities i.e., pandemic, epidemic or natural disaster, that limits or prevents on-site study visits, delivery of injectable IMP directly to a participant's home is not possible because XXB750 is to be administered subcutaneously by a trained and certified personnel or nurse, and because other essential assessments and PK sampling etc. requires the patient to be on site.

Supply, preparation, dispensation and return of IMP will be described in the Pharmacy Manual.

6.2.1 Handling of study treatment

Study treatment must be received by the unblinded person at the study site, handled and stored safely and properly and kept in a secured location which only designated site personnel have access to. Upon receipt, all study treatment must be stored according to the instructions specified in the product information(s) and in the Investigator's Brochure and in accordance with Good Manufacturing Practice (GMP) and Good Clinical Practice (GCP) procedures.

Clinical supplies are to be dispensed only in accordance with the protocol. Technical complaints are to be reported to the respective Novartis Country Organization Quality Assurance.

The subcutaneous study treatment of XXB750 or respective placebo needs to be prepared by unblinded qualified site personnel (in accordance with local regulations, e.g. pharmacist) in order to ensure treatment blinding. Details about handling, storage and administration will be provided in a separate Pharmacy Manual.

Medication labels will be in the local language and comply with the legal requirements of each country. They will include storage conditions for the study treatment but no information about the participant except for the medication number.

The designated site staff must maintain an accurate record of the shipment and dispensing of IMP in the IRT drug accountability log. Monitoring of drug accountability will be performed by field monitors during site or remote monitoring visits, and at the completion of the trial. The investigator must provide accountability also for locally sourced study materials/treatment used for administration (e.g. i.v. syringes).

The site may destroy and document destruction of unused study treatment, drug labels and packaging, as appropriate in compliance with site processes, monitoring processes, and per local regulation/guidelines. Otherwise, the investigator will return all unused study treatment, packaging, drug labels, and a copy of the completed drug accountability log to the Novartis monitor or to the Novartis address provided in the investigator folder at each site.

Oral study medication (i.e. open-label sacubitril/valsartan as active comparator) will be dispensed in an open-label fashion at scheduled site visits. Participants will be asked to return all unused oral study medication and packaging at each site visit or at the time of discontinuation of oral study medication. Site personnel will record the locally sourced supply dispensation into the participant's source document.

6.2.2 Handling of other treatment

The participant's use of other medications will be monitored (dose, dose changes and reasons for changes) throughout the whole duration of the study by documentation of all medications at each visit.

6.2.3 Instruction for prescribing and taking study treatment

All study medications will be administered in addition to other background medications the participant is taking, including other background HF medications.

Table 6-3 shows how the study treatments should be prescribed at each dispensing visit during the randomized treatment period. Please refer to Section 6.5 for dose modification algorithm to manage adverse events that may be contributed to the injectable study medication.

All kits of study treatment assigned by IRT will be recorded in the IRT system.

XXB750 Injectable Study Medication

Participants in treatment arms 1-4 will receive an SC injection of XXB750/matching placebo according to the participant’s randomized treatment assignment. The unblinded, qualified site personnel will prepare the study drug for injection in a blinded way according to the Pharmacy Manual; qualified blinded study staff will inject the study drug subcutaneously in the upper arm or abdomen. Injections should not be given into areas of active skin disease or injury such as sunburns, skin rashes, inflammation, or skin infections. After injection, the participant will be observed for potential hypersensitivity reactions for ≥20 minutes, before he/she can leave the site.

Only the unblinded qualified site personnel in charge of preparing the study medication will be aware of the contents of the administered injectable study medication. Refer to Section 6.2.1 for more details regarding the preparation of the study medications for dispensing.

Oral Study Medication

At every dispensing visit during the randomized treatment period, the open-label sacubitril/valsartan oral study medication will be dispensed to participants in treatment arm 5 in sufficient quantities to last until the next scheduled visit (Section 6.1.2, Table 6-1, and Table 6-3).

- Participants should be instructed to take the oral open-label sacubitril/valsartan study medication twice daily at approximately the same times of each day except for study site visit days. On these days, participants should bring their oral study medication to the site and take it after blood sampling for this visit has been performed.

- Participants should take the oral study medication without regard to food. Each dose may be taken with a glass of water.
- If vomiting occurs during the course of treatment, participants should not take the oral study treatment again before the next scheduled dose.
- If the patient misses taking any study drug dose, he/she should take it as soon as possible, unless if it is almost time for the following scheduled dose. In this case, the patient should skip the missed dose and return back to his/her regular study drug administration schedule.

6.3 Measures to minimize bias: randomization and blinding

6.3.1 Treatment assignment, randomization

At the randomization visit, all eligible participants will be randomized via IRT to one of the treatment arms (see Section 1.2 and Table 6-3). The investigator or his/her delegate will contact the IRT after confirming that the participant fulfills all the inclusion/exclusion criteria. The IRT will assign a randomization number to the participant, which will be used to link the participant to a treatment arm and will specify a unique medication number for the injectable product to be dispensed to the participant.

The randomization numbers will be generated using the following procedure to ensure that treatment assignment is unbiased and concealed from participants and investigator staff. A participant randomization list will be produced by the IRT provider using a validated system that automates the random assignment of participant numbers to randomization numbers. These randomization numbers are linked to the different treatment arms, which in turn are linked to medication numbers. A separate medication list will be produced by or under the responsibility of Novartis Global Clinical Supply (GCS) using a validated system that automates the random assignment of medication numbers to packs containing the investigational product.

Randomization will be stratified by region, and ACEI/ARB or sacubitril/valsartan background medication, respectively. Stratification will serve to prevent a potential imbalance of regions and ACEI/ARB or sacubitril/valsartan background medication, across the arms. In addition, stratified randomization will make sure that arms 1-4 will have 1:2 ratio of participants on ACEI/ARB or sacubitril/valsartan background medication, respectively, and arm 5 will include only participants on prior ACEI/ARB background medication. The randomization scheme for participants will be reviewed and approved by a member of the Randomization Office.

6.3.2 Treatment blinding

Participants, investigator staff, persons performing the assessments, and the Novartis Clinical Trial Team (CTT) will remain blinded to the identity of the treatment from the time of randomization until database lock.

The following methods will be used to maintain the blind:

1. Randomization data will be kept strictly confidential until the time of final database lock and will not be accessible by anyone else involved in the study with the following exceptions:

- Unblinded staff (i.e. pharmacist/nurse) or other designated qualified site personnel who prepare the double-blind study medication doses at the study site and unblinded CRA monitoring the sites who will monitor study medication preparation at the site.
- The bioanalyst for PK analyses (to avoid the unnecessary analysis of samples belonging to participants randomized to placebo treatment)
- The DMC and an external independent analysis team who need to prepare efficacy interim and safety monitoring analysis reports for the DMC. For details on the DMC, please refer to Section 10.1.4.1.

These personnel will not be involved in any other trial activities.

2. The identity of the treatments will be concealed by the use of study treatments that are all identical in packaging, labeling, schedule of administration, and appearance. All XXB750/matching placebo injections will be prepared such that they will all have the same injection volumes regardless of dose to maintain the blinding. Blinded label supply (also mention respective drug name) will be provided at sites to the unblinded site pharmacist to prepare study drug and to conceal treatment code from participants and the investigator staff administering the study injections/dispensing the study medications and performing the assessments.
3. Data with unblinding potential, such as PK concentrations and immunogenicity data collected after the randomization visit, will be kept blinded to all personnel included in study conduct activities until the time of final database lock.
4. Sponsor staff responsible for decision making at the clinical program development level (the Novartis decision making team) will receive the aggregated unblinded results at the treatment group level at the time of the administrative interim analysis informing other programs, as outlined in Table 6-4. The team will not have access to the individual participant treatment codes.

The DMC will review unblinded data reports created by an external independent analysis team. More details about the DMC review process and how unblinding will be handled in the context of DMC reviews will be provided in the DMC charter.

Table 6-4 Blinding and unblinding plan

Role	Time or Event					
	**Randomization list generated**	**Treatment allocation & dosing**	**Safety event (single subject unblinded)**	**Safety monitoring review**	**IA for safety**	**Administrative IA to inform other programs**
Participants	B	B	UI	B	B	B
Site staff	B	B	UI	B	B	B
Unblinded site personnel involved in dose preparation	B	UI	B	B	B	B
Global Clinical Supply	UG/UI	UG/UI	UG/UI	UG/UI	B	B
Randomization Office	UI	UI	UI	UI	UI	UI

Role	Time or Event					
	**Randomization list generated**	**Treatment allocation & dosing**	**Safety event (single subject unblinded)**	**Safety monitoring review**	**IA for safety**	**Administrative IA to inform other programs**
Unblinded Sponsor staff, e.g., for study treatment re-supply, unblinded monitor(s), sample analyst(s)	UG	UI	B	UG	B	B
Unblinded Pharmacovigilance Sponsor staff	B	B	UI	B	B	B
DMC	UI	UI	UI	UI	UI	UI
External independent Statistician/statistical programmer/ data analysts	UI	UI	UI	UI	UI	UI
Biomarker analyst	B	B	B	B	B	B
PK analyst	UI	UI	B	UI	B	B
Sponsor CTT	B	B	B	B	B	B
Novartis decision making team for IAs	B	B	B	B	B	UG
All other Sponsor staff not identified above (i.e. management, support functions)	B	B	B	B	B	B

B: Complete blinded

UG: Unblinded at the group level (i.e. has access to unblinded group level summary results, but not to the individual participant treatment codes)

UI: Unblinded to individual participant treatment codes

6.3.3 Emergency breaking of assigned treatment code

Emergency code breaks must only be undertaken when it is required to in order to treat the participant safely.

Most often, discontinuation from study treatment and knowledge of the possible treatment assignments are sufficient to treat a study participant who presents with an emergency condition. Emergency treatment code breaks are performed using the IRT. When the Investigator contacts the system to break a treatment code for a participant, he/she must provide the requested participant identifying information and confirm the necessity to break the treatment code for the participant. The Investigator will then receive details of the investigational drug treatment for the specified participant and a fax or email confirming this information. The system will automatically inform the Novartis monitor for the site and the study team that the code has been broken.

It is the Investigator's responsibility to ensure that there is a dependable procedure in place to allow access to the IRT/code break cards at any time in case of emergency. The Investigator will provide:

- protocol number
- participant number

In addition, oral and written information to the participant must be provided on how to contact his/her backup in cases of emergency, or when he/she is unavailable, to ensure that un-blinding can be performed at any time.

In case of emergency unblinding, the participants should be discontinued from study treatment and continue with the study visit as scheduled without study medication administration.

6.4 Study treatment compliance

Injections of XXB750 or matching placebo will be administered at the site. All study treatment administered, dispensed and returned must be recorded in the Drug Accountability and Returns Management functionality in the IRT system. The dispensed and returned data recorded electronically in the IRT database will be considered the source record.

For participants randomized to arm 5, the investigator must promote adherence to treatment by instructing the participant to take the study medication exactly as prescribed and by stating that compliance is necessary for the participant's safety and the validity of the study. The participant must also be instructed to contact the investigator if he/she is unable for any reason to take the study treatment as prescribed. Compliance will be assessed by the investigator and/or study personnel at each visit using information provided by the participant. This information should be captured in the source document at each visit.

Pharmacokinetic parameters (measures of treatment exposure) will be determined in all participants treated with XXB750, as detailed in Section 8.7.

6.4.1 Recommended treatment of adverse events

Recommendations for handling liver events are described in Appendix 5.

Recommendations for handling potential drug induced liver injury (DILI) cases are described in Section 6.5.2.1.

Recommendations for handling hypotension are described in Appendix 7.

Recommendations for handling hyperkalemia are described in Appendix 8.

Recommendations for handling renal events are described in Section 10.6.1.

Medication used to treat adverse events (AEs) must be recorded on the appropriate CRF.

6.5 Dose modification

For participants who do not tolerate the protocol-specified dosing schedule, dose adaptations are recommended in order to allow participants to continue the study treatment.

In general, every effort should be made to resolve AEs that may be attributed to study medication and/or foundational background HF treatments before the next dose of study

medication can be administered. Detailed recommendations for dose modifications of concomitant non-HF treatments, diuretics, and foundational background HF treatments (i.e., ACEI/ARB, sacubitril/valsartan, MRA, SGLT2 inhibitor and/or beta-blocker) in case of hypotension or hyperkalemia are provided in Appendix 7 (Section 10.7, Figure 10-1) and Appendix 8 (Section 10.8), respectively.

For dosing of the injectable study medication, the patient should be evaluated at the scheduled study visit when the study medication is due to be administered. The flow chart in Figure 6-1 shows how to evaluate the participant based on the following principles:

- If by the next dosing visit the participant's foundational background HF treatments are fully resumed at pre-study doses, the injectable study medication will be administered as planned.
- If the dose of at least one foundational background HF treatment is still required to be reduced relative to the pre-study dose, the injectable study medication will be administered at the same dose as at the previous visit (i.e., no dose escalation).
- If one of the foundational background HF treatments had been stopped and cannot be resumed due to the AE not being resolved, the injectable study medication dose should be skipped and administered at the next dosing visit at the same dose level as previously administered to the participant if the patient has been able to resume taking the foundational background HF treatment previously stopped.

Figure 6-1 Modification of injectable study medication

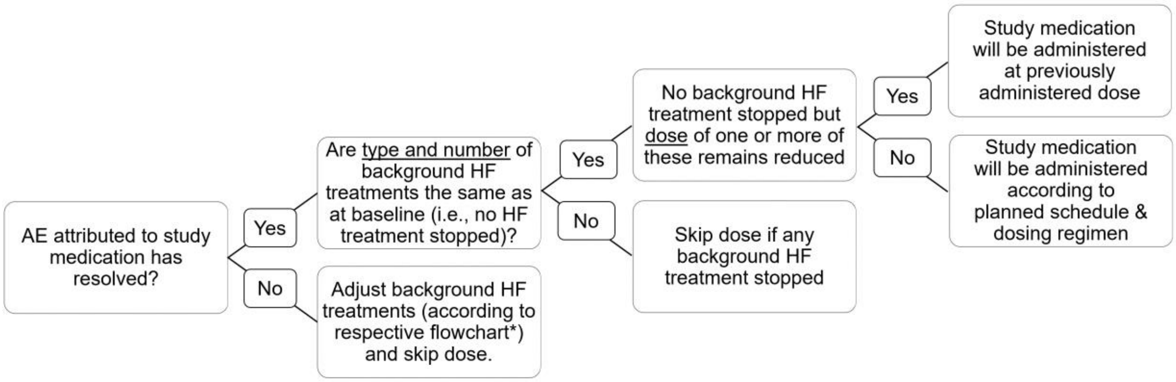

*Figure 10-1

Evaluate foundational background HF treatments (i.e., ACEI/ARB/ARNI, MRA, SGLT2i, beta blocker) relative to baseline with respect to type of drug and dose. Adverse events, especially events indicative of symptomatic hypotension, should be evaluated and potentially require action as outlined in the respective appendix. For the evaluation of planned study drug administration, only AEs related to study drug or foundational background HF treatments should be considered.

Safety criteria determining dose interruption or discontinuation based on liver or renal events are listed in Appendix 5 (Section 10.5) and Appendix 6 (Section 10.6), respectively.

All dose changes must be recorded on the appropriate CRF.

6.5.1 Definitions of dose limiting toxicities (DLTs)

Not applicable.

6.5.2 Follow-up for toxicities

Refer to Appendix 5 (Section 10.5) for the follow-up of liver events and Appendix 6 (Section 10.6) for the follow-up of renal events.

6.5.2.1 Follow up on potential drug-induced liver injury (DILI) cases

Transaminase increases combined with total bilirubin increases may be indicative of potentially severe DILI and should be considered as clinically important events and assessed appropriately to establish the diagnosis. The required clinical information, as detailed below, should be sought to obtain the medical diagnosis of the most likely cause of the observed laboratory abnormalities.

The threshold for potential DILI may depend on the participant's baseline aspartate aminotransferase (AST), alanine aminotransferase (ALT) and total bilirubin (TBL) values; participants meeting any of the following criteria will require further follow-up as outlined below:

- For participants with normal AST and ALT and TBL values at baseline: AST or ALT > 3.0 x ULN combined with TBL > 2.0 x ULN
- For participants with elevated AST or ALT or TBL values at baseline: [AST or ALT > 2 x baseline level] OR [AST or ALT > 300 U/L] (whichever occurs first) combined with [TBL > 2 x baseline level AND > 2.0 x ULN]

As DILI is essentially a diagnosis of exclusion, other causes of abnormal liver tests should be considered and their role clarified before DILI is assumed to be the cause of liver injury.

A detailed history, including relevant information such as review of ethanol consumption, concomitant medications, herbal remedies, supplement consumption, history of any pre-existing liver conditions or risk factors, should be collected.

Laboratory tests should include AST, ALT, TBL, direct and indirect bilirubin, gamma-glutamyl transferase (GGT), prothrombin time (PT)/international normalized ratio (INR), alkaline phosphatase, albumin, and creatine kinase. If available, testing of glutamate dehydrogenase (GLDH) is additionally recommended. Refer to Section 10.5.1 for further guidance.

Perform relevant examinations (ultrasound or MRI, endoscopic retrograde cholangiopancreatography [ERCP]) as appropriate, to rule out an extrahepatic cause of cholestasis.

Table 6-5 provides guidance on specific clinical and diagnostic assessments which can be performed to rule out possible alternative causes of observed liver function test (LFT) abnormalities.

Table 6-5 Diagnostic assessments to determine alternative causes of observed LFT abnormalities

Disease	Assessment
Hepatitis A, B, C, E	• IgM anti-HAV; HBsAg, IgM & IgG anti-HBc, HBV DNA; anti-HCV, HCV RNA, IgM & IgG anti-HEV, HEV RNA
CMV, HSV, EBV infection	• IgM & IgG anti-CMV, IgM & IgG anti-HSV; IgM & IgG anti-EBV

Disease	Assessment
Autoimmune hepatitis	● Antinuclear Antibodies (ANA) & Anti-Smooth Muscle Antibody (ASMA) titers, total IgM, IgG, IgE, IgA
Alcoholic hepatitis	● Ethanol history, GGT, MCV, CD-transferrin
Nonalcoholic steatohepatitis	● Ultrasound or MRI
Hypoxic/ischemic hepatopathy	● Medical history: acute or chronic congestive heart failure, hypotension, hypoxia, hepatic venous occlusion. Ultrasound or MRI
Biliary tract disease	● Ultrasound or MRI, ERCP as appropriate
Wilson disease (if < 40 years old)	● Cæruloplasmin
Hemochromatosis	● Ferritin, transferrin
Alpha-1-antitrypsin deficiency	● Alpha-1-antitrypsin

MCV: mean corpuscular volume; ERCP: Endoscopic retrograde cholangiopancreatography; HAV: hepatitis A virus; HBV: hepatitis B virus; HCV: hepatitis C virus; HEV: hepatitis E virus; CMV: cytomegalovirus; EBV: Epstein Barr virus; CD-transferrin: carbohydrate-deficient transferrin; MRI: magnetic resonance imaging; HBsAg: Hepatitis B virus surface antigen; HSV: Herpes simplex virus

Other causes should also be considered based upon participants' medical history such as hyperthyroidism (causing thyrotoxic hepatitis where T3, T4 and thyroid-stimulating hormone [TSH] should be tested), CV disease (causing ischemic hepatitis where an ECG could be performed, and the history of prior hypotensive episodes should be inquired), and Type 1 diabetes mellitus (causing glycogenic hepatitis).

Obtain PK sample to determine exposure to study treatment and metabolites.

Following the appropriate causality assessments, as outlined above, the causality of the treatment is estimated as "probable" (i.e. > 50% likely), if it appears greater than all other possible causes of liver injury combined. The term "treatment-induced" indicates probably caused by the treatment, not by something else, and only such a case can be considered a DILI case and should be reported as an SAE.

All cases confirmed by repeat testing meeting the laboratory criteria defined above, with no other alternative cause for LFT abnormalities identified, should be considered as "medically significant" and thus, meet the definition of SAE and should be reported as a SAE using the term "potential treatment-induced liver injury." All events should be followed-up with the outcome clearly documented.

6.6 Continued access to study treatment after the end of the study

After Week 16, regular standard of care for heart failure should be provided to the participant by the investigator and/or referring physician as per the local standard of care and applicable guidelines.

6.6.1 Post trial access

No post-trial access program is planned for this trial. Participants who complete or prematurely discontinue participation in this trial will switch to standard of care treatment.

6.7 Treatment of overdose

In the event of an overdose, the investigator should:

- Contact the medical monitor immediately.
- Evaluate the participant to determine, in consultation with the medical monitor, whether study treatment should be interrupted or whether the dose should be reduced.
- Closely monitor the participant for any AE/SAE and laboratory abnormalities as required according to the pharmacodynamic and pharmacokinetic characteristics of the drug concerned.
- Obtain a plasma sample for PK analysis within 6 days from the date of the last dose of injectable study treatment if requested by the medical monitor (determined on a case-by-case basis).
- Document the quantity of the excess dose as well as the duration of the overdose.
- Follow the recommendations of the approved product label about overdose in case this involves sacubitril/valsartan study medication.

6.7.1 Reporting of study treatment errors including misuse/abuse

Study treatment errors are unintentional errors in the prescribing, dispensing, administration or monitoring of study treatment.

Study treatment misuse refers to situations where the study treatment is intentionally and inappropriately used not in accordance with the protocol.

Study treatment abuse corresponds to the persistent or sporadic, intentional excessive use of study treatment, which is accompanied by harmful physical or psychological effects.

Study treatment errors and uses outside of what is foreseen in the protocol, including misuse or abuse, must be reported on the AE (or SAE, if the event meets the definition of an SAE) CRF.

6.8 Concomitant and other therapy

6.8.1 Concomitant therapy

All medications, procedures, and significant non-drug therapies (including physical therapy and blood transfusions) administered after the participant entered the study must be recorded on the appropriate CRFs.

Each concomitant drug must be regularly assessed, based on the locally applicable label, against all entry criteria, prohibited medication and comparator reference labels (i.e. labels selected as Reference Safety Information). If in doubt, the investigator should contact the Novartis medical monitor before randomizing a participant or allowing a new medication to be started. If the participant is already enrolled, contact Novartis to determine if the participant should continue participation in the study. The participant should be instructed and encouraged to report any new medications/therapies he/she takes at any point in the study.

Heart failure therapy

As described in earlier sections of the protocol, all participants will receive the study medication on top of the guideline-directed background heart failure therapy in arms 1-4 or as a replacement of ACEI/ARB therapy in arm 5. Once the participants enters the study, every effort should be

made to avoid dose modifications of all heart failure therapy components, unless absolutely necessary to manage AEs.

If the participant experiences AEs and non-pharmacological interventions were not effective in relieving these AEs, the investigator can down-titrate or stop concomitant medications or oral study medications to rectify the situation, per his/her clinical judgment and reflecting the nature of the AE.

Before adjustment(s) are made to doses of foundational background HF treatments (i.e., ACEI/ARB/ARNI, MRA, beta blocker, and SGLT2 inhibitor), consideration should be given to modifications of other medications (e.g., in the case of hypotension, to adjust diuretics, calcium channel blockers (CCBs), alpha-blockers or nitrates).

If adjustment of background heart failure treatments are considered the only possibility to manage AEs or tolerability, the investigator should down-titrate or temporarily stop these medications (i.e. ACEI/ARB, sacubitril/valsartan, beta-blocker, MRA or SGLT2 inhibitor) or sacubitril/valsartan if this is the randomized study medication (i.e., participant randomized to arm 5) as per his/her clinical judgment. Background HF treatments should be reinstated at their pre-study dose as soon as possible to ensure proper care per the investigator's judgment.

Please refer to Section 10.7 pertaining to hypotension and to Section 10.8 pertaining to hyperkalemia for instructions on the steps to be taken to address these if they occur.

6.8.1.1 Permitted concomitant therapy requiring caution and/or action

Anti-hypertensive medication

Anti-hypertensive medication not directly targeting the RAS (including but not limited to CCBs, alpha-blockers, centrally acting agents, etc.) should be used with caution during screening and randomized treatment periods due to the theoretically increased possibility of occurrence of hypotension.

Phosphodiesterase-5 (PDE-5) inhibitors, oral nitrates and vericiguat

PDE-5 inhibitors, oral nitrates and vericiguat should be used with caution during screening and randomized treatment periods due to the increased possibility of the occurrence of hypotension.

Intravenous (IV) nitrates, nesiritide and carperitide

The concomitant administration of XXB750 with IV nitrates, nesiritide, and carperitide has not been studied. In the event a study participant requires the concomitant administration of one of these IV medications with the study medications, the investigator should consider starting them at a low dose or a slow infusion rate while monitoring the participant's BP carefully.

6.8.2 Prohibited medication

Other than the guideline-directed heart failure treatment mandated in the study, use of the treatments described below are not allowed during the randomized treatment period.

For participants in treatment arm 5, the concomitant use of an ACEI/ARB is strictly prohibited while the patient is receiving study medication during the randomized treatment period. If the

investigator believes that treatment with an ACEI/ARB is necessary, the open-label sacubitril/valsartan study drug must be discontinued. Study medication should be stopped 36 hours prior to the first dose of an ACEI. Similarly, if study medication (i.e. sacubitril/valsartan) is to be restarted, the background treatment with an ACEI should be discontinued 36 hours prior to resuming the open-label sacubitril/valsartan study medication.

6.8.3 Rescue medicine

Not applicable.

7 Discontinuation of study treatment and participant discontinuation/withdrawal

7.1 Discontinuation of study treatment

Discontinuation of study treatment for a participant occurs when study treatment is permanently stopped for any reason (prior to the planned completion of study treatment administration, if any) and can be initiated by either the participant or the Investigator.

The Investigator must discontinue study treatment for a given participant if, he/she believes that continuation would negatively impact the participant's well-being.

Discontinuation from study treatment is required under the following circumstances :

- Participant/guardian decision
- Pregnancy
- Use of prohibited treatment as per recommendations in the prohibited treatment section
- Any situation in which continued study participation might result in a safety risk to the participant
- Following emergency unblinding

If discontinuation from study treatment occurs, the Investigator should make a reasonable effort to understand the primary reason for the participant's discontinuation from study treatment and record this information.

Participants who discontinue from study treatment agree to return for the end of treatment and follow-up visits and undergo all study procedures indicated in Section 1.3 Schedule of Activities. Thus, discontinuation of study medication does not imply discontinuation from the study as a whole, unless the participant withdraws his/her consent for participation. If the participant cannot or is unwilling to attend any visit(s), the site staff should maintain regular telephone contact with the participant, or with a person pre-designated by the participant. This telephone contact should preferably be done according to the study visit schedule.

After discontinuation from study treatment, at a minimum, in abbreviated visits, the following data should be collected at clinic visits or via telephone/email contact:

- New / concomitant treatments
- Adverse Events / Serious Adverse Events

The Investigator must also contact the IRT to register the participant's discontinuation from study treatment.

7.2 Participant discontinuation from the study

Discontinuation from study is when the participant permanently stops receiving the study treatment, and further protocol-required assessments or follow-up, for any reason.

If the participant agrees, a final evaluation at the time of the participant's study discontinuation should be made as detailed in Section 1.3 Schedule of Activities.

7.3 Withdrawal of informed consent and exercise of participants' data privacy rights

Withdrawal of consent/opposition to use of data and/or biological samples occurs in countries where the legal justification to collect and process the data is consent and when a participant:

- Explicitly requests to stop use of their data

  and
- No longer wishes to receive study treatment

  and
- Does not want any further visits or assessments (including further study-related contacts)

This request should be as per local regulations (e.g. in writing) and recorded in the source documentation.

Withdrawal of consent impacts ability to further contact the participant, collect follow-up data (e.g. to respond to data queries) and potentially other country-specific restrictions. It is therefore very important to ensure accurate recording of withdrawal vs. discontinuation based on the protocol definitions of these terms.

In this situation, the Investigator should make a reasonable effort (e.g. telephone, e-mail, letter) to understand the primary reason for the participant's decision to withdraw their consent/exercise data privacy rights and record this information. The Investigator shall clearly document if the participant has withdrawn his/her consent for the use of data in addition to a study discontinuation.

Study treatment must be discontinued and no further assessments conducted, and the data that would have been collected at subsequent visits will be considered missing.

Further attempts to contact the participant are not allowed unless safety findings require communicating or follow-up.

If the participant agrees, a final evaluation at the time of the participant's withdrawal of consent/exercise data privacy rights should be made as detailed in Section 1.3 Schedule of Activities.

Further details on withdrawal of consent or the exercise of participants' data privacy rights are included in the corresponding informed consent form.

7.4 Lost to follow-up

For participants whose status is unclear because they fail to appear for study visits or fail to respond to any site attempts to contact them without stating an intention to discontinue from study treatment or discontinue from study or withdraw consent (or exercise other participants' data privacy rights), the Investigator must show "due diligence" by documenting in the source documents steps taken to contact the participant, e.g. dates of telephone calls, registered letters, etc. A participant should not be considered as lost to follow-up until due diligence has been completed.

7.5 Early study termination by the Sponsor

The study can be terminated by Novartis at any time.

Reasons for early termination (but not limited to) :

- Unexpected, significant, or unacceptable safety risk to participants enrolled in the study.
- Decision based on recommendations from applicable board(s) after review of safety and efficacy data
- Discontinuation of study treatment development

In taking the decision to terminate, Novartis will always consider participant welfare and safety. Should early termination be necessary, participants must be seen as soon as possible and treated as a participant who discontinued from study treatment. Participants should be invited for EOT visit and Investigator should ensure the participant will be appropriately switched to standard of care therapy. The Investigator may be informed of additional procedures to be followed in order to ensure that adequate consideration is given to the protection of the participant's interests. The Investigator or Novartis depending on local regulation will be responsible for informing IRBs/IECs of the early termination of the trial.

8 Study Assessments and Procedures

The Assessment Schedule (Table 1-1) lists all the assessments and when they are performed. All data obtained from these assessments must be supported in the participant's source documentation. Adherence to the study design requirements, including those specified in the Assessment Schedule, are essential and required for study conduct.

8.1 Screening

Once the participant has signed the informed consent form, the participant may enter the screening period.

The initial screening period can last approximately 7 days. Upon verification of eligibility inclusion/exclusion criteria, the participant will be eligible to enter the randomized treatment period. Concomitant medication information will be collected to ensure patient's eligibility for the trial. No changes in the patient's background anti-RAS treatment should be made after screening.

LVEF measurements required for eligibility will be based on the most recent locally obtained echocardiograms, multiple gated acquisition scanning (MUGA), cardiac computed

tomographic angiography (CTA), magnetic resonance imaging (MRI), or ventricular angiographies performed within the last 6 months provided that no subsequent measurements were ≥ 50%. If an LVEF measurement from the last 6 months is not available, the patient may enter the trial based on a LVEF < 50% obtained during the screening period, i.e., before assigning any study medication. If a patient has an implanted cardiac resynchronization therapy device or a myocardial infarction, the LVEF value used to qualify for the study must be obtained at least three months after the implantation of that device or the reference myocardial infarction.

Blood samples obtained for assessing eligibility NT-proBNP must be obtained on the same day as the screening ECG. In the case where a safety laboratory assessment at screening is outside of the allowed range specified in the eligibility criteria, the assessment may be repeated once per the investigator's discretion to confirm the patient's eligibility for continuing in the study. If the repeat value remains outside of the specified range, the participant must be screen failed. Refer to Section 5.3 (Screen failures) for further details.

8.2 Participant demographics/other baseline characteristics

Country-specific regulations should be considered for the collection of demographic and baseline characteristics in alignment with CRF.

Participant demographics: full date (only if required and permitted) or year of birth or age, sex, race/predominant ethnicity (if permitted) and relevant medical history/current medical conditions (until date of signature of informed consent) will be recorded in the eCRF. Where possible, the diagnosis and not symptoms should be recorded. Participant race/ethnicity data are collected and analyzed to assess the degree of diversity of the study population as required by Health Authorities and to identify any differences in the safety and/or efficacy profile of the treatment due to these characteristics.

All prescription medications, over-the-counter drugs and significant non-drug therapies used within 30 days before the start of the study must be documented. See the protocol Section 6.8 for further details on what information must be recorded on the appropriate page of the eCRF.

8.3 Efficacy assessments

Planned time points for all efficacy assessments are provided in the Assessment Schedule (Section 1.3).

8.3.1 NT-proBNP

NT-proBNP will be assessed in all participants by using the central laboratory at the screening visit to determine eligibility for the trial and at time points outlined in the Schedule of Assessments (see Table 1-1). These samples will be obtained before participants take their morning study drug dose (if applicable per assignment to treatment arm 5).

Only the screening NT-proBNP value will be reported to the investigator for the purpose of determining eligibility for inclusion in the study. Samples collected for determining NT-proBNP levels at Randomization and thereafter will be analyzed in batches to ensure uniformity of analytical processes and reagents. Post-Screening NT-proBNP values will not be reported to investigators to avoid the potential introduction of bias. Moreover, investigators are encouraged

to avoid assessing NT-proBNP levels from local laboratories as much as feasible to avoid introduction of bias.

8.3.2 Secondary efficacy assessments

Not applicable.

8.3.3 Appropriateness of efficacy assessments

NT-proBNP is considered the best available surrogate marker when evaluating NP system modulation in heart failure and thus used to assess efficacy in this study. Reduction in NT-proBNP levels has been linked to improved risk of long-term HF outcomes. Refer to Section 4.2.2 for more details.

8.4 Safety assessments

Safety assessments are specified below with Section 1.3 Schedule of Activities detailing when each assessment is to be performed.

For details on AE collection and reporting, refer to Section 8.6.

As per Section 4.5, during a public health emergency as declared by local or regional authorities i.e. pandemic, epidemic or natural disaster, that limits or prevents on-site study visits, regular phone or virtual calls can occur for safety monitoring and discussion of the participant´s health status until it is safe for the participant to visit the site again.

8.4.1 Physical examinations

Physical examinations will be performed by the investigator or his/her designee who are qualified to do so per local regulations/standards. Information about all physical examinations performed must be included in the source documentation at the study site. Clinically relevant findings that are present before signing informed consent must be recorded on the appropriate CRF page that captures medical history. Significant findings made after signing informed consent which meet the definition of an AE must be recorded as an AE.

A **complete physical** examination will include examination of the general appearance, vital signs (blood pressure [SBP and DBP] and pulse), skin, neck (including thyroid), eyes, ears, nose, throat, lungs, heart, abdomen, back, lymph nodes, extremities, the vascular, and the neurological systems. If indicated based on medical history and/or symptoms, rectal, external genitalia, breast, and pelvic exams will be performed.

An **abbreviated physical** examination will include examination of the general appearance and vital signs (blood pressure [SBP and DBP] and pulse).

Refer to Assessment Schedule Table 1-1 for details of the type and frequency of the physical examination.

8.4.2 Vital signs

Vital signs include BP and pulse measurements. BP will be measured in the sitting position after 5 minutes of rest using an automated validated device (e.g., OMRON) or a standard

sphygmomanometer with an appropriately sized cuff on the non-dominant arm. Guidelines for the management of hypotension are provided in Section 10.7.

8.4.3 Height and weight

Height in centimeters (cm) will be measured at screening.

Body weight (to the nearest 0.1 kilogram (kg) in indoor clothing without shoes) will be measured at screening, randomization and every visit thereafter.

8.4.4 Electrocardiograms

Single local 12-lead ECGs will be collected at Screening, Week 16 and Week 24 visits.

ECGs will be locally collected and evaluated. Interpretation of the tracing must be made by a qualified physician and documented in source and on the appropriate CRF. Each ECG tracing should be labeled with the study number, participant initials (where regulations permit), participant number, date and kept in the source documents at the study site. Clinically significant abnormalities present at randomization should be documented as medical history/current medical conditions on the appropriate CRF. New or worsened clinically significant findings occurring after entering the randomized treatment period must be recorded as adverse events.

The original ECGs on non-heat-sensitive paper or a certified copy on non-heat sensitive paper, appropriately signed, must be archived at the study site.

Additionally, unscheduled safety ECGs may be repeated at the discretion of the investigator at any time during the study as clinically indicated. For any ECGs that raises a safety concern, two additional ECGs must be performed to confirm the safety finding. ECG safety monitoring, or a review process (as per the investigator's discretion), should be in place for clinically significant ECG findings at baseline before administration of study treatment and during the study.

8.4.5 Clinical safety laboratory tests

A central laboratory will be used for analysis of all specimens collected. Details on the collection, shipment of samples and reporting of results by the central laboratory are provided to investigators in the laboratory manual. Please refer to Table 1-1.

If participants cannot visit the site for safety lab assessments conducted through central labs during a public health emergency as declared by local or regional authorities, i.e., pandemic, epidemic or natural disaster, that limits or prevents on-site study visits or as deemed appropriate by the investigator in the course of study conduct, an alternative (local) lab collection site may be used.

For China, POCT (e.g. urinalysis, urine pregnancy) is to be analyzed at local laboratories. When study-required testing is not available at a local site, a central lab could be used.

Clinically notable laboratory findings are defined in Section 10.3.1. Clinically significant abnormalities must be recorded as either medical history/current medical conditions or adverse events as appropriate.

Table 8-1 lists the clinical safety laboratory tests performed during the study. The tests with an asterisk (*) beside their names are part of the abbreviated panel of clinical safety laboratory tests.

Table 8-1 Clinical Safety Laboratory Tests

Test Category	Test Name
Hematology	Hematocrit, Hemoglobin, Mean Corpuscular Hemoglobin, Mean Corpuscular HGB Concentration, Mean Corpuscular Volume, Platelets, Erythrocytes, Leukocytes, Differential (% Basophils, Eosinophils, Lymphocytes, Monocytes, Neutrophils), Hemoglobin A1c
Chemistry	Albumin*, Alkaline phosphatase*, ALT*, AST*, Gamma-glutamyl-transferase (GGT)*, Lactate Dehydrogenase (LDH), Calcium*, Magnesium, Phosphate, Chloride, Sodium*, Potassium*, Creatinine (enzymatic)*, Creatine Kinase*, Direct Bilirubin*, Total Bilirubin, Lipid Panel (Total Cholesterol, LDL Cholesterol, High-density lipoprotein [HDL] Cholesterol, Triglycerides [random]), Total Protein, Blood Urea Nitrogen (BUN)*, eGFR*, Uric Acid, Amylase, Lipase, Glucose (random)
Urinalysis	Macroscopic (Dipstick) Panel* (Color, Bilirubin, Occult Blood, Macroscopic Blood, Glucose, Ketones, Leukocyte Esterase, Nitrite, pH, Protein, Specific Gravity, Urobilinogen)<br>Microscopic Panel** (Erythrocytes, Leukocytes, Casts, Crystals, Bacteria, Epithelial cells)
Coagulation	Prothrombin Time/INR*
Pregnancy Test	Highly sensitive serum or urine human chorionic gonadotropin (hCG) pregnancy test (as needed for women of childbearing potential)
Assessments of Fertility	Follicle-stimulating hormone and estradiol (as needed in women of non-child bearing potential only)
*Test is part of the abbreviated panel of clinical safety laboratory tests<br>**Microscopic Panel only to be tested in the event that the Macroscopic (Dipstick) results were positive	

8.4.6 Pregnancy testing

All women of childbearing potential will undergo pregnancy testing. Additional pregnancy testing might be performed if requested by local requirements.

Serum pregnancy testing with beta-human chorionic gonadotropin (β-hCG) must be done at screening, at Week 16 (EOT) visit and at Week 24 (EOS) visit. Urine pregnancy test should be performed at randomization visit (before randomization), at Week 4, Week 8, Week 12, and Week 20. A negative Screening serum pregnancy test result (not older than 7 days) **and** a negative Randomization urine pregnancy test result, must be available before the subject is randomized. A positive urine pregnancy test must be confirmed with a serum pregnancy test. If the latter is also positive, the participant must be discontinued from study treatment.

As per Section 4.5, during a public health emergency as declared by local or regional authorities' i.e. pandemic, epidemic or natural disaster, that limits or prevents on-site study

visits, if participants cannot visit the site to have serum pregnancy tests, urine pregnancy test kits may be used. Relevant participants can perform the urine pregnancy test at home and report the result to the site. It is important that participants are instructed to perform the urine pregnancy test first and only if the test result is negative proceed with the administration of the study treatment. A communication process should be established with the participant so that the site is informed and can verify the pregnancy test results (e.g. following country specific measures).

In general, the levels of a mAb like XXB750 in semen are expected to be low; therefore, the subsequent levels in female partner would be negligible. Based on this principle, the probability that XXB750 would cause teratogenicity is very low. Therefore, no restrictions will be placed on male participants with regard to sexual activity or condom use.

Assessments of fertility

Assessment of fertility must be performed at screening. A woman is considered of childbearing potential from menarche and until becoming post-menopausal unless permanently sterile. Permanent sterilization methods include hysterectomy, bilateral tubal ligation, bilateral salpingectomy and bilateral oophorectomy. In the case of oophorectomy alone, only when the reproductive status of the woman has been confirmed by follow-up hormone level assessment is she considered to be not of child-bearing potential. Medical documentation of permanent method of sterilization must be retained as source documents.

A postmenopausal state is defined as no menses for 12 months without an alternative medical cause and an appropriate clinical profile.

In absence of the medical documentation confirming permanent sterilization, or if the post-menopausal status is not clear, the investigator should use his medical judgment to appropriately evaluate the fertility state of the woman and document it in the source document. Also, in these cases, follicle-stimulating hormone (FSH) testing is required, at screening, for any female participant regardless of reported reproductive/menopausal status.

8.4.7 Other safety evaluations

8.4.7.1 Liver safety monitoring

To ensure participant safety and enhance reliability in determining the hepatotoxic potential of an investigational drug, a standardized process for identification, monitoring and evaluation of liver events has to be followed.

Please refer to Table 10-1 in Section 10.5.1 for complete definitions of liver laboratory triggers.

Once a participant is exposed to study treatment, every liver event defined in Table 10-1 should be followed up by the investigator or designated personnel at the trial site, as summarized below. Additional details on actions required in case of liver events are outlined in Table 10-2 and Table 10-3. Repeat liver chemistry tests (i.e., SGOT, SGPT, total bilirubin (TBL), PT/INR, alkaline phosphatase (ALP) and GGT) should be done to confirm elevation.

These liver chemistry repeats will be performed using the central laboratory. If results will not be available from the central laboratory, then the repeats can also be performed at a local

laboratory to monitor the safety of the participant. If a liver event is subsequently reported, any local liver chemistry tests previously conducted that are associated with this event should have results recorded on the appropriate CRF.

If the initial elevation is confirmed, close observation of the participant will be initiated, including consideration of treatment interruption if deemed appropriate.

If a liver AE is reported that corresponds to these liver events, an assessment of the causal role of each study medication in the origin of the liver AE must be provided.

The following actions should be considered:

- Discontinuation of the investigational drug (refer to the discontinuation of study treatment, Section 7.1), if appropriate
- Hospitalization of the participant if appropriate
- Causality assessment of the liver event
- Thorough follow-up of the liver event should include, as per the Investigator's discretion: obtaining more details on the history of the liver event (including its symptoms), as well as on prior and concurrent diseases, concomitant drug use, serology tests, imaging and pathology assessments, hepatologist's consultancy, ruling out any underlying liver disease.

In addition, the follow-up actions described in Section 6.5.2.1 should be performed. All follow-up information and findings from consultations and procedures must be recorded as appropriate in the CRFs.

8.4.7.2 Renal safety monitoring

Once a participant is exposed to study treatment, renal laboratory alerts or renal safety events should be monitored and followed up by the investigator or designated trial staff as summarized in Section 10.6.1.

8.4.7.3 Immunogenicity

Immunogenicity testing will be performed on each participant. The results will be analyzed to determine, among others, any impact of anti-drug antibodies (ADA) on the efficacy of XXB750 and on the occurrence of adverse events. Refer to Section 8.9 for more details on immunogenicity testing.

8.4.8 Appropriateness of safety measurements

The safety assessments selected are standard for this indication/participant population. PK sampling and immunogenicity assessments performed periodically during the study will allow to determine relationships between drug blood concentrations, existence of ADA (neutralizing or not) and the occurrence of certain adverse events.

8.5 Additional assessments

8.5.1 Clinical Outcome Assessments (COAs)

As per Section 4.5, during a public health emergency as declared by local or regional authorities i.e. pandemic, epidemic or natural disaster, that limits or prevents on-site study visits, COA data

may be collected remotely (e.g. web portal, telephone interviews) depending on local regulations, technical capabilities, and following any applicable training in the required process.

Patient reported outcomes (PRO)

The participant should be made aware that completed measure(s) are not reviewed by the investigator/study personnel.

The KCCQ is a self-administered questionnaire and requires, on average, 4-6 minutes to complete. It contains 23 items, covering physical function, clinical symptoms, social function, self-efficacy and knowledge, and Quality of Life (QoL), each with different Likert scaling wording, including limitations, frequency, bother, change in condition, understanding, levels of enjoyment and satisfaction. A change of 5 points on the scale scores, either as a group mean difference or an intra-individual change appears to be clinically significant, based on comparisons of changes in the scale scores to clinical indicators and patient global reports of change. The KCCQ is a valid, reliable and responsive health status measure for patients with CHF and may serve as a clinically meaningful outcome in CV clinical research, patient management and quality assessment (Green et al 2000).

The HF symptoms and physical limitation domains scores show the best correlation for improvements following a CHF exacerbation (Green et al 2000). Thus, the clinical summary score based on the HF symptoms and physical limitation domains scores of the KCCQ will be one exploratory endpoint next to the overall summary score when the instruments will be evaluated as a whole.

The KCCQ questionnaire will be completed at randomization and Week 16 visits, before any other study procedures and regardless of discontinuation of study treatment.

The KCCQ is available in a number of validated translations. However, participants in whose language a validated translation of the KCCQ is not available will be exempt from completing this instrument.

8.5.2 Other assessments

8.5.2.1 Estimated glomerular filtration rate (eGFR)

The eGFR to determine eligibility of the patient for screening into the trial will be calculated at screening from the serum creatinine concentration which will be measured centrally. The eGFR will be further measured as part of the abbreviated safety laboratory samples at each visit to guide the investigator to take any appropriate action as necessary (e.g., stopping study treatment) in case of eGFR decrease.

Estimated GFR will only be calculated using the following version of the CKD-EPI formula (Inker et al 2021):

$eGFR_{cr} = 142 \times \min(S_{cr}/\kappa, 1)^{\alpha} \times \max(S_{cr}/\kappa, 1)^{-1.200} \times 0.9938^{Age} \times 1.012$ [if female]

where $\kappa = 0.7$ (female) or 0.9 (male) and $\alpha = -0.241$ (female) or -0.302 (male)

8.5.2.2 Urinary cGMP to creatinine ratio

Urinary cGMP as a measure of XXB750's engagement with its target NPR1 will be assessed in spot urine samples at visits as outlined in Table 1-1. To allow indexing of urinary biomarkers, creatinine in spot urine will also be measured. Samples will be obtained before taking the morning study medication (as applicable per patient's treatment assignment).

8.5.2.3 Urine albumin to creatinine ratio (UACR)

Spot urine samples will be collected before taking the morning study drug dose (as applicable per assigned study medication) at the visits indicated in Table 1-1 for analysis of urinary albumin and creatinine (measured as UACR).

8.6 Adverse events (AEs), serious adverse events (SAEs), and other safety reporting

The definitions of adverse events (AEs) and serious adverse events (SAEs) can be found in Section 8.6.

AEs will be reported by the participant (or, when appropriate, by a caregiver, surrogate, or the participant's legally authorized representative).

The Investigator and any qualified designees are responsible for detecting, documenting, and recording events that meet the definition of an AE or SAE and remain responsible for following up all AEs (see Section 7).

For the investigational product, information about adverse drug reactions and how to manage them can be found in the Investigator's Brochure (IB) or equivalent documentation. Information about adverse drug reactions can also be found in the product information for marketed products.

The method of recording, evaluating, and assessing causality of AEs and SAEs and the procedures for completing and transmitting SAE reports are provided in Section 8.6.3.

8.6.1 Adverse events

An adverse event (AE) is any untoward medical occurrence (e.g., any occurrence of unfavorable and unintended sign(s), symptom(s) or medical condition, including abnormal laboratory findings, or worsening of any pre-existing sign(s), symptom(s) or medical condition) in a participant after providing written informed consent for participation in the study. Therefore, an AE may or may not be temporally or causally associated with the use of any treatment used in this study. This includes events reported by the participant (or, when appropriate, by a caregiver, surrogate, or the participant's legally authorized representative).

Abnormal laboratory values or test results constitute AEs only if they fulfill at least one of the following criteria:

- they induce clinical signs or symptoms
- they are considered clinically significant
- they require therapy

Clinically significant abnormal laboratory values or test results must be identified through a review of values outside of normal ranges/clinically notable ranges, significant changes from baseline or the previous visit, or values considered to be non-typical in participant with the underlying disease. Alert ranges for laboratory and other test abnormalities are included in Section 10.3.1.

The Investigator has the responsibility for managing the safety of individual participant and identifying adverse events.

Novartis qualified medical personnel will be readily available to advise on trial-related medical questions or problems.

The occurrence of adverse events must be sought by non-directive questioning of the participant at each visit during the study. Adverse events also may be detected when they are volunteered by the participant during or between visits or through physical examination findings, laboratory test findings, or other assessments, excluding Patient-Reported Outcomes (which are related to the underlying heart failure).

Adverse events must be recorded under the signs, symptoms, or diagnosis associated with them, accompanied by the following information (as far as possible) (if the event is serious refer to Section 8.6.2):

1. The severity grade.
   - Mild: A type of AE that is usually transient and may require only minimal treatment or therapeutic intervention. The event does not generally interfere with usual activities of daily living.
   - Moderate: A type of AE that is usually alleviated with additional specific therapeutic intervention. The event interferes with usual activities of daily living, causing discomfort but poses no significant or permanent risk of harm to the research participant.
   - Severe: A type of adverse event that interrupts usual activities of daily living, or significantly affects clinical status, or may require intensive therapeutic intervention
2. Causality
   - The investigator is obligated to assess the relationship between any treatment used in the study (study treatment, AxMP(s)) and each occurrence of each AE. The investigator will use clinical judgment to determine the relationship. A reasonable possibility of a relationship conveys that there are facts, evidence, and/or arguments to suggest a causal relationship, rather than a relationship cannot be ruled out. Alternative causes, such as underlying disease(s), concomitant therapy, and other risk factors, as well as the temporal relationship of the event to study intervention administration, will be considered and investigated. For causality assessment, the investigator will also consult the IB and/or product information, for marketed products. The causality assessment is one of the criteria used when determining regulatory reporting requirements.
3. Its duration (start and end dates or ongoing) and the outcome must be reported.
4. Whether it constitutes a SAE (see Section 8.6.2 for definition of SAE) and which seriousness criteria have been met.

5. Action taken regarding with study treatment. All adverse events must be treated appropriately. Treatment may include one or more of the following:
   - Dose not changed
   - Dose Reduced/increased
   - Drug interrupted/permanently discontinued
6. Its outcome

Conditions that were already present at the time of informed consent should be recorded in medical history of the participant.

Adverse events (including lab abnormalities that constitute AEs) should be described using a diagnosis whenever possible, rather than individual underlying signs and symptoms.

Adverse event monitoring should be continued at least until Visit Week 24 (EOS) or until 3 months after the last dose of injectable study medication, whichever is longer.

Once an adverse event is detected, it must be followed until its resolution or until it is judged to be not recovered/not resolved (e.g. continuing at the end of the study), and assessment must be made at each visit (or more frequently, if necessary) of any changes in severity, the suspected relationship to the interventions required to treat it, and the outcome.

Information about adverse drug reactions for the investigational drug can be found in the Investigator's Brochure (IB).

Abnormal laboratory values or test results constitute adverse events only if they fulfill at least one of the following criteria:

- they induce clinical signs or symptoms
- they are considered clinically significant
- they require therapy

Clinically significant abnormal laboratory values or test results must be identified through a review of values outside of normal ranges/clinically notable ranges, significant changes from baseline or the previous visit, or values which are considered to be non-typical in participant with the underlying disease. Alert ranges for laboratory and other test abnormalities are included in Section 10.3.

Reporting of AEs related to AxMP(s)

All AEs related to any authorized AxMP used in this study must be reported to Novartis.

In assessing causality, the investigators will use the points above.

If a suspicion that a medical occurrence could be related to study treatment (and/or interaction with study treatment) cannot be ruled out, the reporting rules for study treatment apply.

Handling of AEs

All adverse events must be treated appropriately. More information about how to manage AEs can be found in the IB. Information about adverse drug reactions can also be found in product information for marketed products.

Once an AE is detected, the Investigator must pro-actively followed up the participant, until resolution of the AE, or until it is judged to be not recovered/not resolved (e.g., continuing at the end of the study), or until stabilization, or until the participant is lost to follow-up. Any change in severity or suspected relationship to study treatment must be assessed at each visit (or more frequently, if necessary).

8.6.2 Serious adverse events

An SAE is defined as any adverse event [appearance of (or worsening of any pre-existing)] undesirable sign(s), symptom(s), or medical conditions(s) which meets any one of the following criteria:

- fatal
- life-threatening

Life-threatening in the context of a SAE refers to a reaction in which the participant was at risk of death at the time of the reaction; it does not refer to a reaction that hypothetically might have caused death if it were more severe (please refer to the ICH-E2D Guidelines).

- results in persistent or significant disability/incapacity
- constitutes a congenital anomaly/birth defect, fetal death or a congenital abnormality or birth defect
- requires inpatient hospitalization or prolongation of existing hospitalization, unless hospitalization is for:
    - routine treatment or monitoring of the studied indication, not associated with any deterioration in condition
    - elective or pre-planned treatment for a pre-existing condition that is unrelated to the indication under study and has not worsened since signing the informed consent
    - social reasons and respite care in the absence of any deterioration in the participant's general condition
    - treatment on an emergency outpatient basis for an event not fulfilling any of the definitions of a SAE given above and not resulting in hospital admission
- is medically significant, e.g. defined as an event that jeopardizes the participant or may require medical or surgical intervention to prevent one of the outcomes listed above

Medical and scientific judgment should be exercised in deciding whether other situations should be considered serious reactions, such as important medical events that might not be immediately life-threatening or result in death or hospitalization but might jeopardize the participant or might require intervention to prevent one of the other outcomes listed above. Such events should be considered as "medically significant." Examples of such events are intensive treatment in an emergency room or at home for allergic bronchospasm, blood dyscrasias, or convulsions that do not result in hospitalization or development of dependency or abuse (please refer to the ICH-E2D Guidelines).

All new malignant neoplasms will be assessed as serious under "medically significant" if other seriousness criteria are not met.

All reports of intentional misuse and abuse of the product are also considered serious adverse events irrespective of whether a clinical event has occurred.

Any suspected transmission via a medicinal product of an infectious agent is also considered a serious adverse reaction.

8.6.3 SAE reporting

To ensure participant safety, every SAE, regardless of causality, occurring after the participant has provided informed consent and until End of Study visit must be reported to Novartis safety immediately, without undue delay, but under no circumstances later than within 24 hours of obtaining knowledge of the events (Note: If more stringent, local regulations regarding reporting timelines prevail). Detailed instructions regarding the submission process and requirements are to be found in the Investigator folder provided to each site. Information about all SAEs is collected and recorded on the electronic Serious Adverse Event Report Form with paper backup (as further detailed in the CRF Completion Guideline). All applicable sections of the form must be completed in order to provide a clinically thorough report.

SAE occurring between the time the participant has provided informed consent until either EOS visit or until 3 months after the last dose of injectable study medication (whichever is longer) must be reported to Novartis. Any SAE experienced by participants who discontinue study treatment prematurely but remain in the study for follow-up, should continue to be reported until either EOS visit or until 3 months after the last dose of injectable study medication (whichever is longer).

All follow-up information for the SAE including information on complications, progression of the initial SAE and recurrent episodes must be reported as follow-up to the original episode immediately, without undue delay, but under no circumstances later than within 24 hours of the Investigator receiving the follow-up information (Note: If more stringent, local regulations regarding reporting timelines prevail). An SAE occurring at a different time interval or otherwise considered completely unrelated to a previously reported one must be reported separately as a new event.

Any SAEs experienced after the EOS visit or 3 months after the last dose of injectable study medication (whichever is longer) should only be reported to Novartis Safety if the Investigator suspects a causal relationship to study treatment, unless otherwise specified by local law/regulations.

The investigator must review and provide an assessment of causality for each SAE. There may be situations in which an SAE has occurred, and the investigator has minimal information to include in the initial report to Novartis. However, it is very important that the investigator always makes an assessment of causality for every event before the initial transmission of the SAE data to Novartis. The investigator may change his/her opinion of causality in light of follow-up information and send an SAE follow-up report with the updated causality assessment.

Reporting of Suspected Unexpected Serious Adverse Reactions (SUSARs)

If a SAE is not previously documented in the IB or product information for marketed products and is thought to be related to any study treatment, Novartis may urgently require further information from the investigator for HA reporting. Novartis may need to issue an Investigator

Notification (IN) to inform all investigators involved in any study with the same study treatment. SUSARs will be reported to the competent authorities and relevant ethics committees in accordance with national regulatory requirements in participating countries, including EU Clinical Trial Regulation 536/2014.

Study-specific unblinding rules for suspected unexpected serious adverse reactions (SUSARs) that are disease-related

In clinical trials evaluating treatments for high morbidity and/or high mortality disease states, SAEs that are known consequences of the underlying disease or condition under investigation, or events common in the study population, are anticipated to occur with some frequency during the course of the trial, regardless of drug exposure. While the investigator must still report all SAEs according to the instructions provided in Section 8.6.2 and Section 8.6.3, SUSARs containing terms that are considered consistent with the MedDRA Preferred Terms listed in Table 8-2 will not be unblinded and will not be reported in an expedited timeframe to regulatory agencies, ethic committees or investigators during the course of the study (FDA 2021). Investigator notifications will not be issued for these reports. An external independent DMC will review data of the ongoing trial on a regular basis (Section 10.1.4.1). The opinion and the recommendations of the DMC will be notified by Novartis as soon as possible to the competent authorities and the EC's where they qualify for expedited reporting. If specifically requested by a local Health Authority (HA), SUSARs reporting the terms in Table 8-2 will be expedited to this Health Authority as blinded reports and Investigator notifications will not be issued for these events. Alternatively, if specifically requested by a local HA, SUSARs reporting the terms in Table 8-2 that occur in participants under the jurisdiction of the requesting HA will be expedited to that HA as unblinded reports and investigator notifications will be issued for these events to investigators under the jurisdiction of this HA. These events will be presented in the clinical study report at the end of the study. Any other SUSAR not reporting any term included in Table 8-2 will be unblinded and reported to the competent authorities and relevant ECs. In these cases, IN will be issued when applicable.

Table 8-2 MedDRA PT related to the underlying heart failure

Cardiac output decreased	Pulmonary arterial wedge pressure increased	Cardiogenic shock	Ventricular failure	Pulmonary oedema
Left ventricular end-diastolic pressure decreased	Pulmonary arterial wedge pressure abnormal	Cardiac failure high output	Low cardiac output syndrome	Left ventricular failure
Ejection fraction abnormal	Cardiac failure	Cardiac failure congestive	Cardio-respiratory distress	Acute left ventricular failure
Ejection fraction decreased	Cardiac failure acute	Cardiac failure chronic	Acute pulmonary oedema	

Reporting of SAEs related to AxMP(s)

All SAEs related to any AxMP (whether authorized or not) used in this study must be reported to Novartis within 24 hours of the site becoming aware of it. In assessing causality, the investigators will use the points above. If a suspicion that the medical occurrence could be

related to study treatment (or and interaction with study treatment) cannot be ruled out, the reporting rules for study treatment apply.

8.6.4 Pregnancy

- Details of all pregnancies in female participants will be collected after the start of study treatment and until 3 months after the last dose of injectable study medication .
- While pregnancy itself is not considered to be an AE or SAE, any pregnancy complication or elective termination of a pregnancy for medical reasons will be reported as an AE or SAE.
- Abnormal pregnancy outcomes (e.g. spontaneous abortion, fetal death, stillbirth, congenital anomalies, ectopic pregnancy) are considered SAEs and will be reported as such.
- Any post study pregnancy-related SAE considered reasonably related to the study treatment by the Investigator will be reported to Novartis as described in Section 8.6.3. While the Investigator is not obligated to actively seek this information in former study participants, he or she may learn of an SAE through spontaneous reporting.
- Any female participant who becomes pregnant while participating in the study will discontinue study treatment. This participant may stay in the study and follow the assessments, if she wishes to do so. All assessments that are considered as a risk during pregnancy must not be performed. The participant may continue all other protocol assessments.

Pregnancies

If a female trial participant becomes pregnant, the study treatment should be stopped, and the pregnancy consent form should be presented to the trial participant. The participant must be given adequate time to read, review and sign the pregnancy consent form. This consent form is necessary to allow the Investigator to collect and report information regarding the pregnancy. To ensure participant safety, each pregnancy occurring after signing the informed consent must be reported to Novartis within 24 hours of learning of its occurrence. The pregnancy should be followed up to determine outcome, including spontaneous or voluntary termination, details of the birth, and the presence or absence of any birth defects, congenital abnormalities, or maternal and/or newborn complications.

Pregnancy should be recorded and reported by the Investigator to the Novartis PS&PV Department. Pregnancy follow-up should be recorded on the same form and should include an assessment of the possible relationship to the study treatment of any pregnancy outcome. Any SAE experienced during pregnancy must be reported.

After consent is provided, the pregnancy reporting will occur up to one year after the estimated date of delivery. Post-natal follow-up should occur at 1, 3 and 12 months after delivery.

8.6.5 Disease-related events and/or disease-related outcomes not qualifying as AEs or SAEs

No disease-related events not qualifying as AEs or SAEs are specified in this study.

8.6.6 Adverse events of special interest

Adverse events of special interest (AESI) are defined as events which are of scientific and medical interest specific to Novartis's product or program, for which ongoing monitoring may be appropriate. Such events may require further investigation in order to characterize and understand them. AESI are defined on the basis of potential safety risks for the product, class effects, and data from preclinical studies. The following are currently defined as AESI for XXB750 in this study: serious hypotension (i.e., meeting any of the seriousness criteria listed in Section 8.6.2), serious hypersensitivity reactions (including, but not limited to, allergic reactions, anaphylaxis or anaphylactoid reactions, cytokine release syndrome, serum sickness or serum sickness-like reactions), serious injection site reactions, severe tachycardia, severe acute bradycardia and serious presyncope/syncope.

8.7 Pharmacokinetics

A population pharmacokinetics (PK) sub-study will be conducted in approximately 200 participants who are randomized to either XXB750 or matching placebo. Pharmacokinetics samples will be collected in these participants at the visits defined in the assessment schedule at randomization (2-4 hours post dose), Week 4 (prior to AND 2-4 hours post dose), Week 12 (prior to AND 2-4 hours post dose), Week 16 and Week 24. Follow instructions outlined in the laboratory manual regarding sample collection, numbering, processing and shipment. In order to better define the PK profile, the timing of the PK sample collection may be altered based on emergent data. The number of samples/blood draws and total blood volume collected will not exceed those stated in the protocol.

Pharmacokinetic samples will be obtained in all eligible participants enrolled at sites participating in this sub-study, but evaluated only in participants treated with XXB750. XXB750 concentration will be determined by a validated method; the anticipated Lower Limit of Quantification (LLOQ) is 50 ng/mL. Concentrations will be expressed in mass per volume units. Concentrations below the LLOQ will be reported as "zero" and missing data will be labeled as such in the Bioanalytical Data Report. The individual data will be listed. Population PK analysis of XXB750 will be conducted and the results of this analysis will be reported separately.

8.8 Biomarkers

As described in the Assessment Schedule (Table 1-1), blood and urine will be collected for biomarker assessments from all participants (where feasible). The sample collection information, including the exact time and date of collection, must be entered on the appropriate sample collection eCRF page(s).

Biomarkers related to heart failure and/or XXB750 mechanism of action or treatment effect will be analyzed. In addition to NT-proBNP, plasma and urine cGMP, BNP and hs-Troponin will be analyzed. Biomarkers related to the renin-angiotensin-aldosterone system may also be tested using biobanked samples. ANP will be tested at baseline to determine if ANP levels at the start of XXB750 treatment modify its NT-proBNP effect. Urine creatinine will be analyzed to allow indexing of urine cGMP and other urine biomarkers. Proteins (via SomaScan profiling) or

metabolites that may be associated with heart failure development or progression, study drug treatment response or predict response to treatment will also be analyzed.

Due to both posture and time of collection affecting levels of several of the planned biomarkers, the laboratory manual will include instructions for: 1) participants to be calm and seated for 15 minutes prior to blood biomarker sampling and 2) urine and blood biomarker samples be collected in the morning with no greater than a 1-hour difference between collection times at the post-randomization visits compared to the time at the randomization visit. For a detailed description regarding sample collection, numbering, processing, and shipment, please refer to the instructions provided in the laboratory manual.

Biobank samples (where permitted) will be collected to allow the list of biomarkers to change during the study due to new assay availability, technical issues or as more relevant biomarkers/proteins are determined during conduct of the XXB750 program or clinical research in heart failure. Thus, additional biomarkers related to XXB750 or heart failure, may be tested during the study or after study completion. Additional biomarkers may include those related to fibrosis such as soluble ST2 or collagen synthesis or degradation biomarkers. Except for NT-proBNP levels at screening, biomarker results will remain blinded and will not be available to investigators or the Novartis study team during the study. The Novartis biomarker bioanalyst, who is not involved in study management, will have access to unblinded biomarker data to assess for the need of re-analyzing samples due to suspected technical issue. The Novartis biomarker bioanalyst will remain blinded to the patient level and group level treatment assignments during the entire study.

While the goal of the biomarker assessments is to provide supportive data for the clinical study, there may be circumstances when a decision is made to stop a collection, or to not perform or discontinue an analysis due to either practical or strategic reasons (e.g., inadequate sample number, issues related to the quality of the sample or issues related to the assay that preclude analysis, impossibility to perform correlative analyses, etc.). Therefore, depending on the results obtained during the study, sample analysis for specific biomarkers or specific participants may be omitted at the discretion of Novartis.

Exploratory DNA sampling

The study includes an optional genetic research component which requires a separate informed consent signature if the participant agrees to participate. As permitted by local governing regulations and by IRB/EC, it is required as part of this protocol that the investigator presents these options to the participant.

The optional DNA sample is to be collected at randomization or later, as described in Assessment Schedule (Table 1-1). Instructions for sample collection and shipment will be provided in the laboratory manual. The sample will be sent to the Central Lab for DNA extraction and stored by the Central Lab or Novartis until genetic analyses are performed during the study or after the study end.

The purpose of genetic research may be to better understand the safety and efficacy of XXB750, to learn more about human diseases, or to help develop ways to detect, monitor and treat diseases. As technology changes over time, the most appropriate technology will be used at the

time the exploratory genetic research is performed. This may include the study of the entire genome.

The use of DNA to search for biomarkers of disease and drug action is exploratory. Any results from this DNA study will not be placed in the participant's medical records. To maximize confidentiality, all samples will be double-coded to prevent the exposure of the participant's information and identity. This double-coding process allows Novartis to go back and destroy the sample at the participant's request. In addition, sample information is stored in one secured database while genetic data is stored in an independent secured database.

8.9 Immunogenicity assessments

Anti-drug antibody (ADA) samples will be obtained in participants randomized to injectable study medication (i.e. treatment arms 1-4) according to Section 1.3 Schedule of Activities. A validated ligand binding assay (i.e. ELISA, MSD, etc.) will be used for the detection of potential anti-XXB750 antibodies. Confirmed immunogenicity positive samples may be further analyzed for presence of neutralizing antibodies using a validated method.

In case of a suspected allergic hypersensitivity reaction, samples to measure ADA and a panel of immunoglobulins (IGs), as well as samples to measure tryptase (if sampling occurs within 4 hours following a suspected hypersensitivity reaction), are also to be collected.

If the hypersensitivity reaction is observed at the site following study drug administration, samples for ADA, the IG panel, and tryptase, should be collected as soon as possible after the reaction (and no longer than 4 hours after the reaction commenced). In the event the reaction occurs off-site, the ADA and IG panel samples should be collected at the time of the next site visit. Additionally, the ADA and IG panel samples should also be collected at the final visit from participants who discontinued study treatment or were withdrawn from the study. IG panel testing is planned for drug-specific IgE and other immunoglobulins (e.g. IgA/IgG1/IgG2/IgG3/IgG4/IgM). Depending on the results of the IG panel, antibody epitope mapping, immunogenicity profiling, and serological profiling may also be performed using backup IG panel and backup pre-dose PK samples.

Follow instructions outlined in the Laboratory Manual regarding sample collection, numbering, processing, and shipment.

The detailed methods for immunogenicity assessment will be described in the Bioanalytical Data Report.

8.10 Medical resource utilization and health economics

Medical resource utilization and health economics parameters are not evaluated in this study.

9 Statistical considerations

9.1 Analysis sets

The following analysis sets will be defined for statistical analysis:

Screened Set (SCR) - All participants who signed the informed consent form. The SCR includes only unique screened participants, i.e., in the re-screened participants only the chronologically last screening data is counted.

Randomized Analysis Set (RAS) - All randomized participants who received a randomization number, regardless of receiving trial medication.

Full Analysis Set (FAS) - All participants to whom study treatment has been assigned by randomization and who are not mis-randomized. Mis-randomized participants are those who have not been qualified for randomization, have been inadvertently randomized into the study and did not receive any double-blind study medication. According to the intent to treat principle, participants will be analyzed according to the treatment they have been assigned to during the randomization procedure.

Safety Set (SAF) - All randomized participants who received at least one dose of study treatment. Participants will be analyzed according to the study treatment received, where treatment received is defined as the randomized/assigned treatment if the participant took at least one dose of that treatment or the first treatment received if the randomized/assigned treatment was never received.

PK Analysis Set (PKS) - All randomized participants with at least one available valid (i.e., not flagged for exclusion) PK concentration measurement, who received any study XXB750 treatment.

Immunogenicity Prevalence Set (IPS) - All participants in the safety set with a non-missing baseline ADA sample **or** at least one non-missing post-baseline ADA sample.

Immunogenicity Incidence Set (IIS) - All participants in the safety set with a non-missing baseline ADA sample **and** at least one non-missing post-baseline ADA sample.

9.2 Statistical analyses

9.2.1 General considerations

Data analyses are described in Section 9 of the study protocol with more details available in statistical analysis plan (SAP).

Unless specified otherwise, baseline is defined as the measurement obtained at the randomization visit, or the measurement obtained at an earlier visit (scheduled or unscheduled) which was closest to the randomization visit, if the randomization visit measurement is missing.

All efficacy analyses will be based on FAS and all safety analyses will be based on SAF unless specified otherwise. In descriptive summaries, continuous variables will be summarized using n, mean, standard deviation (SD), median, minimum, 25th percentile (Q1), 75th percentile (Q3), and maximum; categorical variables will be summarized using frequency and percentage.

9.2.2 Participant demographics and other baseline characteristics

Demographic and other baseline data including disease history and standard background medications will be summarized descriptively by treatment group.

Relevant medical histories and current medical conditions at baseline will be summarized by system organ class and preferred term, by treatment group.

9.2.3 Treatments

The duration of exposure in day(s) to XXB750/matching placebo and sacubitril/valsartan as well as the dose intensity (computed as the ratio of actual cumulative dose received and actual duration of exposure) and the relative dose intensity (computed as the ratio of dose intensity and planned dose intensity) will be summarized by means of descriptive statistics and treatment group using the safety set.

Concomitant medications and significant non-drug therapies prior to and after the start of the study treatment will be summarized, by treatment group.

9.3 Primary endpoint(s)/estimand(s) analysis

Primary estimands are described in Section 3.1. The primary endpoint and statistical analysis will be described in this Section.

9.3.1 Definition of primary endpoint(s)

The primary efficacy variable is change in log NT-proBNP from baseline to Week 16, which is equivalent to the log of ratio of Week 16 NT-proBNP over baseline NT-proBNP.

Missing values at Week 16 will be imputed as described in Section 9.3.3 and Section 9.3.4.

9.3.2 Statistical model, hypothesis, and method of analysis

The primary objective of determination of a dose-response signal and to characterize the dose-response relationship in XXB750 doses compared to placebo will be evaluated using an optimally weighted contrast test following the Multiple Comparison Procedure-Modeling (MCP-MOD) methodology described in Pinheiro et al 2006 and Pinheiro et al 2014.

A candidate model set is defined corresponding to the range of the expected mean response in each of the dose groups. The candidate model set is used to generate a set of weights for the calculation of optimal contrasts between the responses in the studied dose groups and the placebo group. A statistical test comparing all doses in the different dose groups simultaneously to the placebo is used, hence a multiplicity adjustment is applied that accounts for the multiple possible dose response behavior considered as well as the common placebo between dose groups. A critical value is derived from a multivariate t-distribution using the correlation matrix induced by the correlations between the weights corresponding to the candidate sets as well as the correlation between the tests of shapes in the dosing groups to the placebo group.

Test of the dose response signal

The null hypothesis of a flat dose-response relationship for the change from baseline in logarithm NT-proBNP compared to placebo will be tested at a 1-sided significance level of 2.5% against the alternative hypothesis of a dose-response relationship leading to a significant reduction in the NT-proBNP.

Hence, the following null and alternative hypotheses will be tested:

- **H_{10}**: there is no dose-response relationship for XXB750 (i.e. the dose response relationship is flat).
- **H_{11}**: there is a dose-response relationship for XXB750 (i.e. there is more reduction in NT-proBNP in XXB750 doses based on the assumed candidate models).

There are five candidate models to capture the shape of the dose-response relationship for XXB750 at the Week 16 endpoint, as depicted in Figure 9-1. The candidate models generating the contrast weights are described below:

- Model 1: Emax with ED50 at 15 mg dose level
- Model 2: Emax with ED50 at 120 mg dose level
- Model 3: Sigmoid Emax with ED50 at 60 mg dose level and hill parameter h=3
- Model 4: Sigmoid Emax with ED50 at 120 mg dose level and hill parameter h=3
- Model 5: Beta-model with delta1=0.2, delta2=0.3 and scale=288

Figure 9-1 Dose-response curve of candidate models

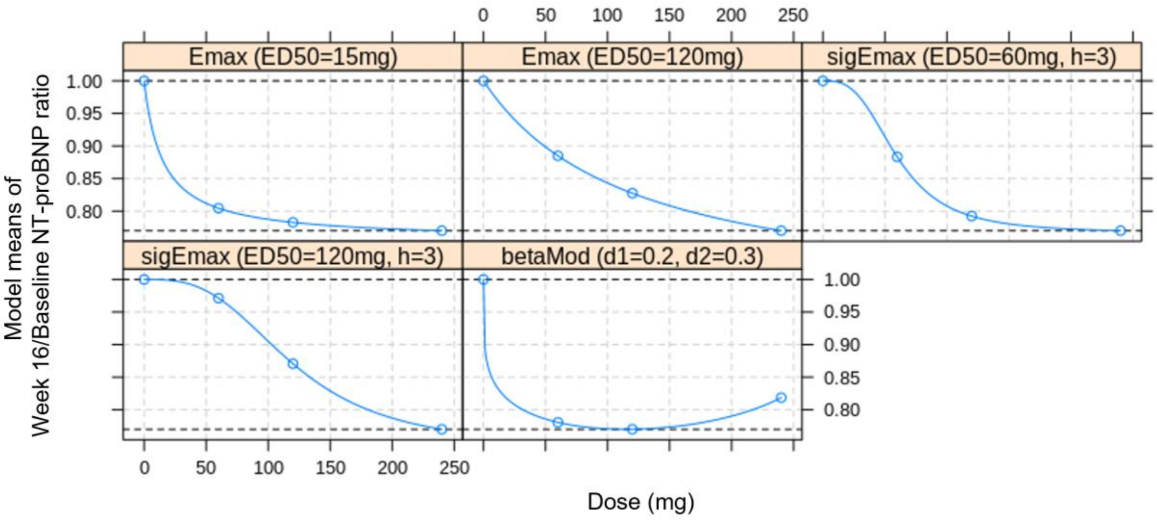

The analysis to derive the test statistics is based on an analysis of covariance (ANCOVA) model with the change from baseline logarithm of NT-proBNP to Week 16 as response variable, treatment (placebo and XXB750 dose groups), LVEF (≤ 40%, > 40%), background medication stratification factor (ACEI/ARB, sacubitril/valsartan), and region stratification factor as factors, and baseline logarithm of NT-proBNP as a covariate.

The response variable of the change in logarithm NT-proBNP from baseline at Week 16 used in the above ANCOVA model is from an imputed dataset, where a missing Week 16 NT-proBNP is imputed using the multiple imputation method as described in Section 9.3.3 and Section 9.3.4. In order to account for the imputation uncertainty, this ANCOVA model will be repeated for each imputed dataset, which results in a set of least squares (LS) mean estimate for all treatment groups and the related covariance matrices. Rubin's rule will be used to combine the multiple sets of LS mean estimates and the related covariance matrices to a single

set of LS mean estimates of change of NT-proBNP at Week 16 for all treatment groups and the related covariance matrix.

The optimal contrasts derived from the candidate model sets will be applied to the combined estimated dose means and covariance matrix to obtain t-statistics for each candidate model and the common critical value $C_{0.025}$. $C_{0.025}$ is the common critical value derived from the reference multivariate t-distribution with the 5x5 correlation matrix induced by test the candidate dose response models with respect to comparing all XXB750 dose groups to placebo.

The H_{10} will be rejected and the statistical significance of dose-response in NT-proBNP reduction is established if the max (t1, t2,..., t5) $\geq C_{0.025}$.

Model averaging to obtain the dose response

The response data in each imputed dataset, including relevant covariates, will be used to fit the models in the candidate set. The estimated dose-response will be derived by using model averaging methods on a subset of candidate models, for which the associated contrast tests are statistically significant. If there are more than three candidate models that are statistically significant, the top three models with largest t-statistics as calculated above will be selected as basis for the model averaging.

Model averaging will be carried out for each imputed dataset, and the resulting mean efficacy estimates and confidence intervals will be derived using the combination variance that accounts for the uncertainty of the imputed data using Rubin's rules. Comparisons between XXB750 doses and placebo will be simultaneously derived for the model averaged estimates together with confidence intervals reflecting the imputation procedure applied.

Selection of the optimal dose will be based on the model averaged dose response estimates of NT-proBNP reduction of XXB750 over the dose range studied.

Summaries of absolute values and change from baseline in NT-proBNP by treatment group and visit will be presented using statistics as described in Section 9.2.1 additionally with geometric mean and coefficient of variation. Figures will be produced to visually show the mean NT-proBNP level by visit over study period (randomized treatment period and safety follow-up period) for each treatment group.

The FAS will be used for the above analyses.

9.3.3 Handling of intercurrent events of primary estimand (if applicable)

Intercurrent events for the primary endpoint are defined in Section 3.1 and the strategy for handling of intercurrent events in the primary analysis is described below:

1. Discontinuation of study treatment: NT-proBNP data collected after discontinuation of study treatment will be used in the analysis (treatment policy strategy).
2. Unforeseen change in the dose of allowed concomitant medications: NT-proBNP data collected after unforeseen change in the dose of allowed concomitant medication will be used in the analysis (treatment policy strategy).
3. Receiving prohibited concomitant medication: NT-proBNP data collected after receiving prohibited concomitant medication will be used in the analysis (treatment policy strategy).

If a missing primary endpoint occurs after intercurrent event(s), it will be imputed based on participants with observed endpoint after same type of intercurrent event(s) in the respective treatment group. If there is no or very limited participants with observed endpoint after intercurrent event(s) in the respective treatment group, then the missing primary endpoint will be imputed based on the respective treatment group combined with lower dose group(s). if there are still very limited participants with observed endpoint after intercurrent event(s), then missing will be imputed based on those with observed endpoint in the placebo group.

9.3.4 Handling of missing values not related to intercurrent event

Missing data for the primary endpoint will be imputed using a multiple imputation approach assuming that missingness mechanism can be retrieved from observed data (missing at random (MAR)). The imputation model will include the longitudinal sequence of NT-proBNP data collected at randomization, Week 4, Week 8, Week 12, and Week 16 visits, LVEF factor (≤ 40%, > 40%), background medication stratification factor (ACEI/ARB, sacubitril/valsartan), region stratification factor, and other baseline variables as appropriate, imputing for each treatment group separately.

The full detailed information about the multiple imputation algorithms will be specified in a separate statistical analysis plan.

9.3.5 Multiplicity adjustment (if applicable)

Please refer to Section 9.3.2 for primary analysis of MCP-MOD for multiplicity adjustment with respect to testing for a dose response signal across multiple candidate shapes. The secondary hypotheses included in the order of the hierarchical testing procedure are an evaluation and demonstration of: (1) treatment effect of highest XXB750 target dose level versus placebo; (2) combined two highest XXB750 target dose levels versus conversion to sacubitril/valsartan in participants receiving ACEI/ARB background medication. The secondary hypotheses will be tested in the order described above and statistical inference will be made only if the primary hypothesis is rejected.

9.3.6 Sensitivity analyses

The primary analysis for which missing data imputation is based on missing at random or retrieve dropouts would favor the XXB750 treatment groups when early treatment/study discontinuation is due to XXB750 related adverse events or if the participant dies. Placebo multiple imputation method will be performed, when participants randomized to XXB750 treatment groups are assumed to be similar to placebo treated participants after above mentioned events.

9.3.7 Supplementary analysis

Supportive analyses for the primary analysis results are listed below:

1. Results based on the single best dose response model fit will also be reported.
2. The mixed model for repeated measure (MMRM) analysis of covariance (ANCOVA) with treatment, LVEF (≤40%, >40%), background medication stratification factor (ACEI/ARB, sacubitril/valsartan), and region stratification factor, visit (Week 4, Week 8, Week 12, or Week 16), and treatment-by-visit as factors, and baseline logarithm NT-proBNP as a

covariate, with a common unstructured covariance matrix among visits will be used. Treatment comparisons at Week 16 will be provided. The analysis will be based on all available data up to Week 16 in the FAS and based on likelihood method with an assumption of missing at random for missing data.

3. In addition to treatment policy strategy to handle the intercurrent events in the primary analysis, a supplemental analysis based on a hypothetical strategy will be also used to handle the intercurrent events. This strategy assumes that premature discontinuations of study treatment, change in the dose of allowed concomitant medication, receiving prohibited concomitant medication or lost to follow-up/discontinue study would not have occurred in the participants. This analysis uses partial data available up to the occurrence of the intercurrent events, and missing data after the intercurrent events will be imputed as based on the pattern of other participants who do not have intercurrent events (missing at random). The imputation steps are same as described in Section 9.3.4 and the newly imputed data will be used in the analysis as described in Section 9.3.2.
4. Analysis based on the while on-treatment strategy to handle the intercurrent of premature discontinuations of study treatment will be performed. This analysis uses the data taken at the end of treatment visit as the Week 16 assessment and repeat the analysis described in Section 9.3.2.
5. Treatment grouping is based on highest dose received and repeat the analysis described in Section 9.3.2.
6. Subgroup analyses will be performed for the primary analysis. The analysis will be done using the analysis specified in Section 9.3.2 at individual subgroup level separately. Please see Section 9.7 for subgroup details.

Summary statistics for NT-proBNP and change from baseline will be summarized by treatment group for observed and imputed values. Figures will be produced to visually show the observed and the imputed mean changes by visit over 16 weeks randomized treatment epoch for each treatment group. FAS will be used for the analyses.

9.4 Secondary endpoint(s)/estimand(s) analysis

The secondary efficacy endpoints are described in Section 9.4.1 and are part of the hierarchical testing strategy. Analyses related to safety endpoints are described in Section 9.4.2.

9.4.1 Efficacy and/or pharmacodynamic endpoint(s)

There are two efficacy secondary objectives:

1. To evaluate the treatment effect of the highest XXB750 target dose level compared to placebo in reducing NT-proBNP from baseline at Week 16 in HF patients treated with standard of care, including ACEI/ARB or sacubitril/valsartan.
2. To evaluate the treatment effect of combined two highest XXB750 target dose levels in addition to a background of ACEI/ARB versus conversion from ACEI/ARB to sacubitril/valsartan, in reducing NT-proBNP from baseline to Week 16.

The efficacy variable in secondary objective 1 is the change from baseline at Week 16 in logarithm of NT-proBNP and will be analyzed using a repeated measures ANCOVA model with treatment (highest XXB750 target dose level or placebo), LVEF (≤40%, >40%),

background medication stratification (ACEI/ARB, sacubitril/valsartan), and region stratification factor, visit (Week 4, Week 8, Week 12, or Week 16), and treatment-by-visit as factors, and baseline logarithm of NT-proBNP as a covariate, with a common unstructured covariance matrix among visits. Within treatment estimates and between treatment comparisons at Week 16 will be provided. Please refer to Section 3.2 and Section 9.3.3 for the intercurrent events and its handling strategy and Section 9.3.4 for handling missing data not related to intercurrent event. The analysis will be run on each of the imputed datasets as described in Section 9.3.2. Overall results are obtained by applying Rubin's rules on the estimates obtained from imputed datasets.

The efficacy variable in secondary objective 2 and analysis model is the same as those described above in secondary objective 1, except that treatments being compared are combined two highest XXB750 target dose levels with background medication of ACEI/ARB stratum versus conversion from ACEI/ARB to sacubitril/valsartan. In the event treatment arms 4a and 4b are eliminated, the comparison will be between only the 120 mg XXB750 and the sacubitril/valsartan treatment arms.

9.4.2 Safety endpoints

For all safety analyses, the safety set will be used unless specified otherwise. All listings and tables will be presented by treatment group.

Adverse events

All information obtained on adverse events will be displayed by treatment group and participant.

The number (and percentage) of participants with treatment emergent adverse events (events started after the first dose of study medication or events present prior to start of double-blind treatment but increased in severity based on preferred term) will be summarized in the following ways:

- by treatment, primary system organ class and preferred term.
- by treatment, primary system organ class, preferred term and maximum severity.
- by treatment, Standardized MedDRA Query (SMQ) and preferred term.

Separate summaries will be provided for study medication related adverse events, death, serious adverse events, other significant adverse events leading to discontinuation, and adverse events leading to dose adjustment.

The number (and proportion) of participants with adverse events of special interest and related to potential risks (as per the Investigator's Brochure) will be summarized by treatment.

A participant with multiple adverse events within a primary system organ class is only counted once towards the total of the primary system organ class.

Adverse events which will be counted for a specific treatment period are those which are treatment emergent. These events are those with an onset after the start of the treatment period, or which were present prior to the start of the treatment period but increased in severity, changed

from being not suspected to being suspected of study treatment relationship, or developed into SAEs after the start of the treatment period.

Vital signs

Summary statistics will be provided by treatment and visit for all vital signs, as well as for the proportion of participants presenting with clinically notable abnormal values. Graphical displays of the values of selected vital signs over time may be provided by treatment group.

More details on the analysis of vital signs will be described in the Statistical Analysis Plan (SAP).

12-lead ECG

A standard 12 lead ECG will be performed at Randomization, Week 16, and Week 24. Interpretation of the tracing must be made by a qualified physician and documented on the ECG section of the CRF. Each ECG tracing should be labeled with the study and subject number, date, and kept in the source documents at the study site. Clinically significant abnormalities should also be recorded on the Medical History/AE CRF page.

Summary Statistics for continuous data and number and percentage of participants for categorical data (e.g. interpretation) will be provided by treatment and visit.

More details on the analysis of ECG parameters will be described in the SAP.

Clinical laboratory evaluations

Laboratory data collected during the study will be summarized by presenting shift tables using extended normal ranges (baseline to most extreme post-baseline value), summary statistics of raw data and change from baseline by treatment and visit. The number of participants meeting pre-defined clinically notably laboratory values and percent changes will also be summarized by treatment.

Immunogenicity

All immunogenicity results including (the incidence of ADA and/or neutralizing antibodies) will be summarized by treatment and listed by treatment group, participant, and visit/time. IPS and IIS will be used for analyses.

More details on the analysis of immunogenicity parameters will be described in the SAP.

9.5 Exploratory endpoint(s)/estimand(s) analysis

Exploratory objectives are defined in Section 3.

Analyses of the exploratory efficacy variables will be based on the Full Analysis Set (FAS). Summary statistics by treatment group will be performed for the baseline values, the post-baseline values, and the change from baseline, with baseline defined as the randomization visit (or the measurement obtained at an earlier visit (scheduled or unscheduled) which is closest to the randomization visit, if the randomization visit measurement is missing). At each post baseline visit, only participants with a value at both baseline and that post-baseline visit will be

included in the change from baseline summary and missing post-baseline values will not be imputed.

9.5.1 Exploratory variables

Below are the variables for the exploratory analysis:

1. Number of winners based on the hierarchically ordered composite endpoint consisting of: 1) time to all-cause death; 2) number of HF hospitalizations; 3) categorical KCCQ CSS change; 4) NYHA class change; 5) relative change in NT-proBNP levels from baseline to Week 16;
2. Rate of change in eGFR (eGFR slope) from baseline to Week 16;
3. Change from baseline at assessment visits in biomarkers (details are provided in Section 9.5.4);
4. Change in NYHA class from baseline to Week 16;
5. Changes in HF signs and symptoms from baseline to Week 16;
6. Change in all domains of the Kansas City Cardiomyopathy Questionnaire from baseline to Week 16;
7. Population PK analysis results including mathematic models and parameters (details are provided in Section 9.5.3);
8. Incidence of anti-XXB750 antibodies (details are provided in Section 9.5.5).

9.5.2 Analysis methods

In general, exploratory variables will be analyzed in the FAS unless specified otherwise. Statistical tests will be performed at the two-sided significance level of 0.05. To better satisfy the normality assumption, the log-transformation will be taken on each biomarker prior to statistical analysis. There will be no multiplicity adjustment for any analysis of exploratory variables.

Win ratio analysis

The hierarchical ordered composite endpoint will be analyzed using the win ratio approach (Pocock et al 2012), with treatment as fixed-effect factor and stratified by region and background medication (ACEI/ARB, sacubitril/valsartan) (Dong et al 2018). Every participant in the highest XXB750 target dose level group will be compared to every participant in the placebo group within each stratum in the following order:

1. all pairs will be compared first by time to all-cause death; if there is no winner, then
2. they will be compared for the number of HF hospitalizations; if there is no winner, then
3. they will be compared according to the combination of change in KCCQ CSS and change in NYHA class from baseline to Week 16; if still there is no winner, then
4. they will be compared according to reduction in NT-proBNP levels from baseline to Week 16.
5. If the comparison between changes in NT-proBNP levels does not result in a winner, the respective pairwise comparison is tied.

When comparing the time to all-cause death and number of HF hospitalizations between two participants and at least one of the times is censored, the comparison is only performed up to the censoring time. Three categories are defined for the change of KCCQ CSS: worsened (decrease of 5 points or more), improved (increase of 5 points or more), or unchanged (neither worsened nor improved). The comparison of KCCQ CSS categorical change is in favor of the participant in the highest XXB750 arm if the category of the participant is better compared to that in placebo arm. Similarly, three categories are defined for the change of NYHA class, worsened, improved, or unchanged; and the comparison of NYHA class change is in favor of the participant in the highest XXB750 arm if the class change of the participant is better compared to that in the placebo arm. Please refer to Table 9-1 for the example of combination of change in KCCQ CSS and change in NYHA class.

Table 9-1 Selecting a winner based on combination of change of KCCQ CSS and change of NYHA class

Example	Participant A		Participant B		Result of comparison
	Change of KCCQ CSS from baseline to Week 16	Change of NYHA Class from baseline to Week 16	Change of KCCQ CSS from baseline to Week 16	Change of NYHA Class from baseline to Week 16	
1	Improved	Improved	Improved	Improved	Tie
2	Improved	Worsened	Worsened	Improved	Tie
3	Worsened	Unchanged	Worsened	Worsened	A wins
4	Worsened	Worsened	Worsened	Improved	B wins

The comparison of NT-proBNP is performed based on the relative change between baseline and Week 16 and takes into account that relative changes within ±25% are considered medically irrelevant; in detail, let μ_{XXB750} and $\mu_{placebo}$ be the relative changes of participants in the highest XXB750 and the placebo arm, respectively. Then, a comparison of NT-proBNP is in favor of the participant in the highest XXB750 arm if $\mu_{XXB750} < 0.75\ \mu_{placebo}$. On the other hand, the comparison is in favor of the participant in the placebo arm if $\mu_{placebo} < 0.75\ \mu_{XXB750}$.

The win ratio is estimated by the ratio of the number of winners in the highest XXB750 treatment group and the number of winners in the placebo group with weights defined as the reciprocal of the stratum sample size.

$$\Psi = \frac{\sum_{m=1}^{M} w^{(m)} n_{XXB750}^{(m)}}{\sum_{m=1}^{M} w^{(m)} n_{placebo}^{(m)}}$$

where $n_{XXB750}^{(m)}$ and $n_{placebo}^{(m)}$ are the total of wins in the mth stratum for the highest XXB750 and placebo treatment groups, respectively; $w^{(m)}=1/N^{(m)}$ and $N^{(m)}$ is the total sample size in the mth stratum.

The estimated win ratio and the corresponding 2-sided 95% CI will be provided. The contribution to the number of winners of each component of the win ratio will be summarized.

Analysis of continuous variables

Continuous variables, which are assumed to be normally distributed, are:

- Rate of change (slope) in eGFR from baseline at Week 16;
- Change in KCCQ from baseline at Week 16.

For the rate change in eGFR, the eGFR slope will be estimated from a repeated measures ANCOVA model including treatment, LVEF (≤40%, >40%), background medication stratification factor (ACEI/ARB, sacubitril/valsartan), region stratification factor, time (when the eGFR is assessed in weeks), and treatment-by-time as fixed effects with random intercept and slope (time) and a common unstructured covariance. The least-square means of slopes for within and between treatment groups, and the corresponding two-sided 95% confidence interval will be provided.

Changes from baseline in KCCQ scores will be analyzed based on an ANCOVA model in which treatment, LVEF (≤40%, >40%), background medication stratification factor (ACEI/ARB, sacubitril/valsartan) and region stratification factor will be included as factors and baseline value as a covariate. Treatment comparisons and effect size estimates at Week 16 will be provided. The analysis will be performed based on all available data up to Week 16 in the FAS.

Analysis of ordinal variables

The ordinal variables include

- Change in NYHA class from baseline;
- Changes in HF signs and symptoms from baseline.

The ordinal variables will be analyzed, at Week 4, Week 8, Week 12, and Week 16, using a repeated measures proportional cumulative odds model. The model will include patient as a random effect and the respective baseline category, treatment, LVEF (≤40%, >40%), background medication stratification factor (ACEI/ARB, sacubitril/valsartan), region stratification factor, visit (all available post-randomization visits) and treatment-by-visit interaction as fixed effect factors. This model assumes that the treatment effect sizes across measurement categories are the same. The visit-wise effect size estimates and their 95% confidence intervals will also be provided. The analysis will be based on all available data in the FAS and likelihood method with an assumption of missing at random for missing data.

For population PK analysis and incidence of anti-XXB750 antibodies, please refer to Section 9.5.3 and Section 9.5.5, respectively.

Analysis methods for biomarkers are described in Section 9.5.4 with more details available in the statistical analysis plan (SAP).

All exploratory endpoints will be summarized over 16 weeks randomized treatment epoch for each treatment group. Additional summary over safety follow-up will be provided as appropriate. FAS will be used for analyses.

9.5.3 Pharmacokinetics

Sparse PK samples are collected for population PK analysis. Results will be reported separately. Individual drug concentration will be listed in Clinical Study Report (CSR).

9.5.4 Biomarkers

Both blood and urine samles will be collected at all sites where geographically feasible at Randomization, Week 8, Week 12, and Week 16 as described in Table 1-1. Biomarkers to be analyzed may include, but are not necessarily limited to: cyclic GMP (plasma and urine), urinary cGMP-to-creatinine ratio, urinary albumin-to-creatinine ratio, BNP and hs-Troponin (in addition to primary endpoint NT-proBNP).

The change from baseline to a pre-defined time-point in logarithmic scale will be analyzed using a repeated measures ANCOVA model with treatment, LVEF (≤40%, >40%), background medication stratification factor (ACEI/ARB, sacubitril/valsartan), and region stratification factor, visit (Week 8, Week 12, or Week 16), and treatment-by-visit as factors, and respective baseline in logarithm scale as a covariate, with a common unstructured covariance matrix among visits will be used. Estimates of treatment difference and their two-sided 95% confidence intervals will be provided. These estimates and confidence intervals will be back-transformed for presentation.

In addition, biomarker data will be summarized by treatment and time point. Further displays of biomarker data may be considered and will be described separately in the Statistical Analysis Plan (SAP).

9.5.5 Immunogenicity

All immunogenicity results including the incidence of ADA and/or neutralizing antibodies will be summarized by treatment and listed by treatment group, participant, and visit/time. IPS and IIS will be used for analyses.

More details on the analysis of immunogenicity parameters will be described in the SAP.

9.5.6 Patient reported outcomes

The change in HF symptoms and physical limitation scores assessed by the clinical summary score of the KCCQ and the change in other domains of KCCQ is one of the exploratory efficacy endpoints. Please refer to Section 9.5.2 for the analysis method.

9.6 Other safety analyses

Please refer to statistical analysis plan for details.

9.7 Other analyses

9.7.1 Subgroup analyses

The subgroups of interest for primary analysis and selected secondary/exploratory analyses include but are not limited to:

- Age (< 65, ≥ 65)
- Sex (female, male)
- Region
- LVEF (≤ 40%, > 40%)

- Type of RAS inhibition (ACEI/ARB, sacubitril/valsartan)
- Dose level of ACEI/ARB or sacubitril/valsartan at randomization.

Please refer to statistical analysis plan for more details.

9.7.2 DNA

Exploratory DNA studies are designed to investigate the association between genetic factors (genotypes) and clinical assessments (phenotypes) which are collected during the clinical trial. Without prior evidence of a strong association, a number of possible associations are evaluated with exploratory analyses. A range of statistical tests are used for the analyses. Additional data, from other clinical trials, are often needed to confirm associations. Alternatively, if the number of participants enrolled in the study is too small to complete proper statistical analyses, the data may be combined, as appropriate, with those from other studies to enlarge the dataset for analysis.

Data generated on hypothesis-free platforms will be reported separately outside of CSR.

9.8 Interim analysis

One safety interim analysis will be conducted when data from the ongoing XXB750 phase 1b study CXXB750A12101 becomes available. This analysis will include all available data of the study CXXB750A12101 and treatment arms 1-3 and 5 of the current study to assess the need for adapting the design of the current study.

An additional administrative interim analysis informing other programs may be conducted when approximately 430 participants are randomized into the study (approximately 40 participants each in arms 1-4) and are followed for 16 weeks to help the planning of another trial in a different development program. The results of this interim analysis will not lead to any changes in this study and will be kept blinded to anyone directly involved in the conduct of the study. More details about the DMC review process and how unblinding will be handled in the context of DMC reviews will be provided in the DMC charter.

No alpha adjustment will be made for the interim safety analysis, safety data monitoring, and the administrative interim analysis. All interim analyses and safety data monitoring will be performed by an independent statistician who will not be involved in the trial conduct. The results will be reviewed by the independent DMC.

The primary analysis will be performed after all participants have completed Week 16 (or discontinued prior to Week 16). A final analysis will be performed after all participants completed Week 24 (or discontinued prior to Week 24). Formal testing of the primary endpoint with full level alpha of 0.025 (1-sided) will be performed at the primary analysis time point.

Regular safety data monitoring, including the safety interim analysis and the administrative interim analysis, is planned as specified in Section 4.6.

9.9 Sample size determination

The study is planned to randomize 720 participants in total.

9.9.1 Primary endpoint(s)

For the primary endpoint analysis, 600 participants allocated in the ratio of 2:2:3:3 to placebo, XXB750 60 mg dose level, XXB750 120 mg dose level, and highest XXB750 dose level, respectively.

Assuming a common standard deviation of 0.67 for log NT-proBNP change from baseline and a one-sided 2.5% significance level (with adjustments for multiple comparisons using MCP-MOD), a sample size of 600 participants (120 in placebo, 120 in XXB750 60 mg dose level, 180 in XXB750 120 mg dose level, and total of 180 in highest XXB750 dose level) or, in the event treatment arms 4a and 4b are eliminated (see Section 4.6), a sample size of 540 participants (120 in placebo, 120 in XXB750 60 mg dose level, and 300 in XXB750 120 mg dose level) will provide a power of at least 90% if the underlying true maximum NT-proBNP reduction on XXB750 vs. placebo is 23%.

9.9.2 Secondary endpoint(s)

A common standard deviation of 0.67 for log NT-proBNP change from baseline and a one-sided 2.5% significance level is assumed for all sample size determinations relating to secondary endpoints.

For the first secondary endpoint, a sample size of 120 participants in placebo and 180 participants in XXB750 optimal dose level, respectively, will provide a minimum power of 90% if the true NT-proBNP reduction on XXB750 vs. placebo is 23%. In the case of when treatment arms 4a and 4b are eliminated after safety interim analysis, a sample size of 120 participants in placebo and 300 participants in XXB750 120 mg dose level will provide a power of approximately 95% if the true NT-proBNP reduction on XXB750 vs. placebo is 23%.

For the second secondary endpoint, in participants with ACEI/ARB background medication converted to sacubitril/valsartan, a sample size of 120 participants in a group combining the two highest XXB750 target dose levels and 120 participants in sacubitril/valsartan conversion arm will provide approximately 80% power if the underlying true maximum NT-proBNP reduction with adding XXB750 vs. conversion to sacubitril/valsartan is 22%. In the case when treatment arms 4a and 4b are eliminated, a sample size of 100 participants in XXB750 120 mg dose level and 120 participants in sacubitril/valsartan conversion arm will provide approximately 77% power if the underlying true maximum NT-proBNP reduction with adding XXB750 vs. conversion to sacubitril/valsartan is 22%.

10 Supporting documentation and operational considerations

10.1 Appendix 1: Regulatory, ethical, and study oversight considerations

10.1.1 Regulatory and ethical considerations

This study will be conducted in accordance with the protocol and with the following:

- Consensus ethical principles derived from international guidelines including the Declaration of Helsinki and Council for International Organizations of Medical Sciences (CIOMS) international ethical guidelines
- Applicable ICH Good Clinical Practice (GCP) guidelines
- Applicable laws and regulations

The protocol, protocol amendments, ICF, Investigator's Brochure, [IDFU], and other relevant documents (e.g. advertisements) must be submitted to an IRB/IEC by the Investigator and reviewed and approved by the IRB/IEC before the study is initiated.

Any amendments to the protocol will require IRB/IEC approval before implementation of changes made to the study design, except for changes necessary to eliminate an immediate hazard to study participants.

Protocols and any substantial amendments/modifications to the protocol will require health authority approval prior to initiation except for changes necessary to eliminate an immediate hazard to study participants.

The Investigator will be responsible for the following:

Signing a protocol signature page confirming his/her agreement to conduct the study in accordance with these documents and all of the instructions and procedures found in this protocol and to give access to all relevant data and records to Novartis monitors, auditors, Novartis Quality Assurance representatives, designated agents of Novartis, IRBs/IECs, and regulatory authorities as required

Providing written summaries of the status of the study to the IRB/IEC annually or more frequently in accordance with the requirements, policies, and procedures established by the IRB/IEC

Notifying the IRB/IEC of SAEs or other significant safety findings as required by IRB/IEC procedures

Providing oversight of the conduct of the study at the site and adherence to requirements of 21 CFR, ICH guidelines, the IRB/IEC, European regulation 536/2014 for clinical studies (if applicable), European Medical Device Regulation 2017/745 for clinical device research (if applicable), and all other applicable local regulations

Inform Novartis immediately if an inspection of the clinical site is requested by a regulatory authority.

This clinical study was designed and shall be implemented, executed and reported in accordance with the ICH Harmonized Tripartite Guidelines for Good Clinical Practice, with applicable local regulations (including European Directive 2001/20/EC or European Clinical Trial Regulation 536/2014, US CFR 21), and with the ethical principles laid down in the Declaration of Helsinki.

10.1.2 Informed consent process

The Investigator or his/her representative will explain the nature of the study, including the risks and benefits, to the patient or their legally authorized representative and answer all questions regarding the study.

Patients must be informed that their participation is voluntary. Patients or their legally authorized representatives will be required to sign a statement of informed consent that meets the requirements of 21 CFR 50, local regulations, ICH guidelines, privacy and data protection requirements, where applicable, and the IRB/IEC or study center.

Informed consent must be obtained before conducting any study-specific procedures (e.g. all of the procedures described in the protocol). The process of obtaining informed consent must be documented in the participant source documents.

The medical record must include a statement that written informed consent was obtained before the participant was enrolled in the study and the date the written consent was obtained. The authorized person obtaining the informed consent must also sign the ICF.

A copy of the ICF(s) must be provided to the participant or their legally authorized representative.

Patients who are re-screened are required to sign a new ICF.

The ICF will contain a separate section that addresses the use of remaining mandatory samples for optional additional research, e.g. genetic analysis. The Investigator or authorized designee will explain to each participant the objectives of the additional research. Participants will be told that they are free to refuse to participate and may withdraw their consent at any time and for any reason during the storage period. A separate signature will be required to document a participant's agreement to allow any remaining specimens to be used for additional research. Participants who decline to participate in this optional additional research will document this.

Eligible patients may only be included in the study after providing (witnessed, where required by law or regulation), IRB/IEC-approved informed consent.

If applicable, in cases where the participant's representative(s) gives consent (if allowed according to local requirements), the participant must be informed about the study to the extent possible given his/her level of understanding. If the participant is capable of doing so, he/she must indicate agreement by personally signing and dating the written informed consent document.

Information about common side effects already known about the investigational treatment can be found in the Investigator's Brochure (IB). This information will be included in the participant informed consent and should be discussed with the participant upon obtaining consent and also during the study as needed. Any new information regarding the safety profile of the investigational drug that is identified between IB updates will be communicated as appropriate, for example, via an Investigator notification or an aggregate safety finding. New information might require an update to the informed consent and then must be discussed with the participant.

The following informed consents are included in this study:

- Main study consent, which also included:

    - A subsection that requires a separate signature for the 'Optional Consent for Additional Research' to allow future research on data/samples collected during this study
    - Optional consent for activities that may be done outside of the study site
- As applicable, Pregnancy Outcomes Reporting Consent for female participants or the female partners of any male participants who took study treatment
- Patient information sheet for female partners of any male participants who took study treatment
- Optional Genetics Consent to provide a sample for exploratory DNA studies

The study includes an optional sub-study/ DNA component which requires a separate signature if the participant agrees to participate. It is required as part of this protocol that the Investigator presents this option to the participants, as permitted by local governing regulations. The process for obtaining consent should be exactly the same as described above for the main informed consent.

Declining to participate in these optional assessments (DNA) will in no way affect the participant's ability to join the main research study.

A copy of the approved version of all consent forms must be provided to Novartis after IRB/IEC approval.

As per Section 4.5, during a public health emergency as declared by local or regional authorities i.e. pandemic, epidemic or natural disaster, that may challenge the ability to obtain a standard written informed consent due to limits that prevent an on-site visit, Investigator may conduct the informed consent discussion remotely (e.g. telephone, videoconference) if allowable by a local health authority.

Guidance issued by local regulatory bodies on this aspect prevail and must be implemented and appropriately documented (e.g. the presence of an impartial witness, sign/dating separate ICFs by trial participant and person obtaining informed consent, etc.).

10.1.3 Data protection

Participants will be assigned a unique identifier by Novartis. Any participant records or datasets that are transferred to Novartis will contain the identifier only; participant names or any information which would make the participant identifiable will not be transferred.

The participant must be informed that his/her personal study-related data will be used by Novartis in accordance with local data protection law. The level of disclosure must also be explained to the participant who will be required to give consent for their data to be used as described in the informed consent.

The participant must be informed that his/her medical records may be examined by Clinical Quality Assurance auditors or other authorized personnel appointed by Novartis, by appropriate IRB/IEC members, and by inspectors from regulatory authorities.

Novartishas appropriate processes and policies in place to handle personal data breaches according to applicable privacy laws.

10.1.4 Committees structure

In this study, an Executive Committee will support development and conduct of the study, while an independent, program-wide Data Monitoring Committee will oversee patient safety and frequently review patient safety data as outlined below.

10.1.4.1 Data Monitoring Committee

This study will include a data monitoring committee (DMC) which will function independently of all other individuals associated with the conduct of this clinical trial, including the site investigators participating in the study. The DMC will assess at defined intervals the progress of a clinical trial, safety data, and critical efficacy variables and recommend to Novartis whether to continue, modify, or terminate a trial.

At the time of the interim analysis for safety in order to open enrollment into treatment arms 4a and 4b (see Section 4.6), the DMC will have 3 options to recommend based on the safety profile:

- To continue the study as per protocol (i.e. to open enrollment into both arms 4a and 4b as planned).
- To not open both treatment arms 4a and 4b, or to not open treatment arm 4b.
- To replace the originally planned 240 mg target dose with a 180 mg target dose as the highest target dose (at either low and high starting dose or only a low starting dose).

The DMC may also conduct a planned administrative interim analysis to inform other programs. Details on the DMC early safety interim analysis and the administrative interim analysis to inform other programs are described in Section 4.6 and Section 9.8.

More details regarding composition, responsibilities, data monitoring, and meeting frequency, and documentation of DMC reports, minutes, and recommendations will be described in a separate charter that is established between Novartis and the DMC.

10.1.4.2 Executive Committee

The Executive Committee (EC) will be established comprising of outside medical experts i.e. not being members of the DMC and Novartis representatives from the Clinical Trial Team.

The EC will ensure transparent management of the study according to the protocol through recommending and approving modifications as circumstances require. The EC will review protocol amendments as appropriate. Together with the clinical trial team, the EC will also develop recommendations for publications of study results including authorship rules.

10.1.5 Data quality assurance

Monitoring details describing strategy, including definition of study critical data items and processes (e.g. risk-based initiatives in operations and quality such as risk management and mitigation strategies and analytical risk-based monitoring), methods, responsibilities, and requirements, including handling of noncompliance issues and monitoring techniques (central, remote, or on-site monitoring) are provided in the monitoring plan.

Records and documents, including signed ICFs, pertaining to the conduct of this study must be retained by the Investigator for 15 years after study completion unless local regulations or institutional policies require a longer retention period. No records may be destroyed during the retention period without the written approval of Novartis. No records may be transferred to another location or party without written notification to Novartis.

10.1.5.1 Data collection

Designated Investigator staff will enter the data required by the protocol into the Electronic Case Report Forms (eCRF). The eCRFs have been built using fully validated secure web-enabled software that conforms to 21 CFR Part 11 requirements. Investigator site staff will not be given access to the EDC system until they have been trained. Automatic validation programs check for data discrepancies in the eCRFs, allow modification and/or verification of the entered data by the Investigator staff.

The Investigator/designee is responsible for assuring that the data (recorded on CRFs) (entered into eCRF) is complete, accurate, and that entry and updates are performed in a timely manner. The Investigator must certify that the data entered are complete and accurate.

After final database lock, the Investigator will receive copies of the participant data for archiving at the investigational site.

All data should be recorded, handled, and stored in a way that allows its accurate reporting, interpretation, and verification.

10.1.5.2 Database management and quality control

Novartis personnel (or designated CRO) will review the data entered by investigational staff for completeness and accuracy. Electronic data queries stating the nature of the problem and requesting clarification will be created for discrepancies and missing values and sent to the investigational site via the EDC system. Designated Investigator site staff are required to respond promptly to queries and to make any necessary changes to the data.

Concomitant treatments and prior medications entered into the database will be coded using the World Health Organization (WHO) Drug Reference List, which employs the Anatomical Therapeutic Chemical classification system. Medical history/current medical conditions and adverse events will be coded using the Medical Dictionary for Regulatory Activities (MedDRA) terminology.

Dates of screenings, randomizations, screen failures and study completion, as well as randomization codes and data about all study treatment (s) dispensed to the participant and all dosage changes will be tracked using an Interactive Response Technology (IRT). The system will be supplied by a vendor, who will also manage the database. The data will be sent electronically to Novartis (or a designated CRO) at specific timelines.

Each occurrence of a code break via IRT will be reported to the clinical team and monitor. The code break functionality will remain available until study shut down or upon request of Novartis.

Once all the necessary actions have been completed and the database has been declared to be complete and accurate, it will be locked **and the treatment codes will be unblinded** and made

available for data analysis for the CSR at the conclusion of the study. Any changes to the database after that time can only be made after written agreement by Novartis development management.

10.1.6 Source documents

Source documents provide evidence for the existence of the participant and substantiate the integrity of the data collected. Source documents are filed at the Investigator’s site.

Data reported on the CRF or entered in the eCRF that are transcribed from source documents must be consistent with the source documents or the discrepancies must be explained. The Investigator may need to request previous medical records or transfer records, depending on the study. Also, current medical records must be available.

The Investigator must give the monitor access to all relevant source documents to confirm their consistency with the data capture and/or data entry. The Investigator must also keep the original informed consent form signed by the participan*t* (a signed copy is given to the participant). Definition of what constitutes source data and its origin can be found in monitoring guidelines.

The Investigator must maintain accurate documentation (source data) that supports the information entered in the CRF. Study monitors will perform ongoing source data verification to confirm that data entered into the CRF by authorized site personnel are accurate, complete, and verifiable from source documents; that the safety and rights of participants are being protected; and that the study is being conducted in accordance with the currently approved protocol and any other study agreements, ICH GCP, and all applicable regulatory requirements.

Key study personnel must be available to assist the field monitor during all visits. Continuous remote monitoring of each site’s data may be performed by a centralized Novartis / CRA organization. Additionally, a central analytics organization may analyze data & identify risks & trends for site operational parameters, and provide reports to Novartis clinical teams to assist with trial oversight.

10.1.7 Publication policy

The protocol will be registered in a publicly accessible database such as clinicaltrials.gov and as required in EudraCT . In addition, after study completion and finalization of the study report the results of this trial will be submitted for publication and posted in a publicly accessible database of clinical trial results, such as the Novartis clinical trial results website and all required health authority websites (e.g. Clinicaltrials.gov, EudraCT etc.).

For details on the Novartis publication policy including authorship criteria, please refer to the Novartis publication policy training materials that were provided to you at the trial Investigator meetings.

Any data analysis carried out independently by the Investigator should be submitted to Novartis before publication or presentation.

Summary results of primary and secondary endpoints will be disclosed based upon the global Last Participant Last Visit (LPLV) date, since multinational studies are locked and reported based upon the global LPLV.

10.1.8 Protocol adherence and protocol amendments

This protocol defines the study objectives, the study procedures and the data to be collected on study participants. Additional assessments required to ensure safety of participants should be administered as deemed necessary on a case by case basis. Under no circumstances including incidental collection is an Investigator allowed to collect additional data or conduct any additional procedures for any purpose involving any investigational drugs under the protocol, other than the purpose of the study. If despite this interdiction prohibition, data, information, observation would be incidentally collected, the Investigator shall immediately disclose it to Novartis and not use it for any purpose other than the study, except for the appropriate monitoring on study participants.

Investigators ascertain they will apply due diligence to avoid protocol deviations. If an Investigator feels a protocol deviation would improve the conduct of the study this must be considered a protocol amendment, and unless such an amendment is agreed upon by Novartis and approved by the IRB/IEC and health authorities, where required, it cannot be implemented.

10.1.8.1 Protocol amendments

Any change or addition to the protocol can only be made in a written protocol amendment that must be approved by Novartis, health authorities where required, and the IRB/IEC prior to implementation.

Only amendments that are required for participant safety may be implemented immediately provided the health authorities are subsequently notified by protocol amendment and the reviewing IRB/IEC is notified.

Notwithstanding the need for approval of formal protocol amendments, the Investigator is expected to take any immediate action required for the safety of any participant included in this study, even if this action represents a deviation from the protocol. In such cases, Novartis should be notified of this action and the IRB/IEC at the study site should be informed according to local regulations.

10.2 Appendix 2: Abbreviations and definitions

10.2.1 List of abbreviations

ACEI(s)	Angiotensin converting enzyme inhibitor(s)
AE	Adverse Event
AESI	Adverse Events of Special Interest
ALP	Alkaline Phosphatase
ALT	Alanine Aminotransferase
ANA	Antinuclear Antibodies
ANCOVA	Analysis of covariance
ARB(s)	Angiotensin receptor blocker(s)
ARNI	Angiotensin receptor neprilysin inhibitor
ASCT	Autologous Stem Cell Transplant
ASMA	Anti-smooth muscle antibody
AST	Aspartate Aminotransferase
AV	Atrio-ventricular
AxMP	Auxiliary Medicinal Product
bid	bis in die/twice a day
BLRM	Bayesian Logistic Regression Model
BMA	Bone Modifying Agents
BMI	Body Mass Index
BP	Blood pressure
BUN	Blood Urea Nitrogen
CABG	Coronary artery bypass graft
CDS	Core Data Sheet
cGMP	Cyclic guanosine monophosphate
CK	Creatine Kinase
CO	Country Organization
COA	Clinical Outcome Assessment
CQA	Clinical Quality Assurance
CRF	Case Report/Record Form (paper or electronic)
CRO	Contract Research Organization
CRT	Cardiac resynchronization therapy
CSR	Clinical study report
CTT	Clinical Trial Team
CV	Cardiovascular
DBP	Diastolic Blood Pressure
DIN	Drug Inducted Nephrotoxicity
DLT	Dose Limiting Toxicity
DMC	Data Monitoring Committee
ECG	Electrocardiogram
EDC	Electronic Data Capture
eGFR	Estimated glomerular filtration rate
EOS	End of study
EOT	End of treatment

ERCP	Endoscopic retrograde cholangiopancreatography
eSAE	Electronic Serious Adverse Event
eSource	Electronic Source
FDA	Food and Drug Administration
FIH	First in Human
FSH	Follicle Stimulating Hormone
GCP	Good Clinical Practice
GCS	Global Clinical Supply
GGT	Gamma-glutamyl transferase
GLDH	Glutamate Dehydrogenase
GMP	Good Manufacturing Practice
h	Hour
HBsAg	Hepatitis B virus surface antigen
HBV	Hepatitis B Virus
HCV	Hepatitis C Virus
HED	Human Equivalent Dose
HF	Heart failure
HFmrEF	Heart failure with mildly reduced ejection fraction
HFrEF	Heart failure with reduced ejection fraction
HSCT	Hematopoietic Stem Cell Transplant
i.v.	intravenous
IB	Investigator’s Brochure
ICF	Informed Consent Form
ICH	International Council for Harmonization of Technical Requirements for Pharmaceuticals for Human Use
IDFU	Investigational directions for use
IEC	Independent Ethics Committee
IN	Investigator Notification
INR	International Normalized Ratio
IRB	Institutional Review Board
IRT	Interactive Response Technology
KCCQ	Kansa City Cardiomyopathy Questionnaire
LCZ696	Sacubitril/valsartan
LDH	lactate dehydrogenase
LFT	Liver function test
LLN	lower limit of normal
LLOQ	lower limit of quantification
LVEF	Left ventricular ejection fraction
MedDRA	Medical dictionary for regulatory activities
mg	milligram(s)
mL	milliliter(s)
MRA(s)	Mineralcorticoid receptor antagonist(s)
Nab	Neutralizing antibody
NCDS	Novartis Clinical Data Standards
NP(s)	Natriuretic petide(s)
NPR1	Natriuretic Peptide Receptor 1

NT-proBNP	N-terminal prohormone B-type natriuretic peptide
NYHA	New York Heart Association
OHP	Off-site Healthcare Professional
p.o.	Oral(ly)
PA	Posteroanterior
PCI	Percutaneous coronary intervention
PD	Pharmacodynamic(s)
PK	Pharmacokinetic(s)
PKS	PK analysis set
POCT	Point of Care Testing
PRO	Patient Reported Outcomes
PS&PV	Patient Safety and Pharmacovigilance
PSD	Premature Subject Discontinuation
PT	prothrombin time
q4w	Every 4 weeks
QD	Once a day
QoL	Quality of life
R Value	ALT/ALP x ULN
RAAS	Renin-angiotensin-aldosterone system
RD	Recommended Dose
RU	Resource Utilization
s.c.	subcutaneous
SAE	Serious Adverse Event
SAP	Statistical Analysis Plan
SBP	Systolic Blood Pressure
SD	standard deviation
SGLT2I(s)	Sodium Glucose Co-transporter-2 Inhibitor(s)
SGOT	Serum Glutamic Oxaloacetic Transaminase
SGPT	Serum Glutamic Pyruvic Transaminase
SoA	Schedule of Activities
SUSAR	Suspected Unexpected Serious Adverse Reaction
TBL	Total bilirubin level
ULN	upper limit of normal
WHO	World Health Organization

10.2.2 Definitions

Additional treatment	Medicinal products that may be used during the clinical trial as described in the protocol, but not as an investigational medicinal product (e.g. any background therapy)
Assessment	A procedure used to generate data required by the study
Auxiliary medicinal product	Medicinal product used for the needs of a clinical trial as described in the protocol, but not as an investigational medicinal product (e.g. rescue medication, challenge agents, background treatment or medicinal products used to assess end-points in the clinical trial).<br>Concomitant therapy is not considered as AMP.
Biologic Samples	A biological specimen including, for example, blood (plasma, serum), saliva, tissue, urine, stool, etc. taken from a study participant
Clinical Outcome Assessment (COA)	A measure that describes or reflects how a participant feels, functions, or survives
Clinical Trial Team	A group of people responsible for the planning, execution and reporting of all clinical trial activities. Examples of team members include the Study Lead, Medical Monitor, Trial Statistician etc.
Coded Data	Personal Data which has been de-identified by the investigative center team by replacing personal identifiers with a code.
Discontinuation from study	Point/time when the participant permanently stops receiving the study treatment and further protocol required assessments or follow-up, for any reason. No specific request is made to stop the use of their samples or data.
Discontinuation from study treatment	Point/time when the participant permanently stops receiving the study treatment for any reason (prior to the planned completion of study intervention administration, if any). Participant agrees to the other protocol required assessments including follow-up. No specific request is made to stop the use of their samples or data.
Dosage	Dose of the study treatment given to the participant in a time unit (e.g. 100 mg once a day, 75 mg twice a day)
Electronic Data Capture (EDC)	Electronic data capture (EDC) is the electronic acquisition of clinical study data using data collection systems, such as Web-based applications, interactive voice response systems and clinical laboratory interfaces. EDC includes the use of Electronic Case Report Forms (eCRFs) which are used to capture data transcribed from source data/documents used at the point of care
End of the clinical trial	The end of the clinical trial is defined as the last visit of the last participant.
Enrollment	Point/time of participant entry into the study at which informed consent must be obtained. The action of enrolling one or more participants
eSource (DDE)	eSource Direct Data Entry (DDE) refers to the capture of clinical study data electronically, at the point of care. eSource Platform/Applications combines source documents and case report forms (eCRFs) into one application, allowing for the real time collection of clinical trial information to Novartis and other oversight authorities, as appropriate
Estimand	As defined in the ICH E9(R1) addendum, estimand is a precise description of the treatment effect reflecting the clinical question posed by the trial objective. It summarizes at a population-level what the outcomes would be in the same participants under different treatment conditions being compared. Attributes of an estimand include the population, variable (or endpoint) and treatment of interest, as well as the specification of how the remaining intercurrent events are addressed and a population-level summary for the variable.
Intercurrent events	Events occurring after treatment initiation that affect either the interpretation or the existence of the measurements associated with the clinical question of interest.
Investigational drug/ treatment	The drug whose properties are being tested in the study
Investigational Product/	A pharmaceutical form of an active ingredient or placebo being tested or used as a reference (such as an active comparator) in a clinical trial, including a product with a

Investigational Medicinal product	marketing authorization when used or assembled (formulated or packaged) in a way different from the approved form, or when used for an unapproved indication, or when used to gain further information about an approved use.
Medication number	A unique identifier on the label of medication kits
Mis-randomized participants	Mis-randomized participants are those who were not qualified for randomization and who did not take study treatment, but have been inadvertently randomized into the study or the participant allocated to an invalid stratification factor
Off-site	Describes trial activities that are performed at remote location by an off-site healthcare professional, such as procedures performed at the participant's home.
Off-site healthcare Professional (OHP)	A qualified healthcare professional, who performs certain protocol procedures for the participant in an off-site location such as a participant's home.
Orthostatic hypotension	A reduction in SBP of ≥20 mmHg or in DBP of ≥10 mmHg within 3 min of standing
Other treatment	Treatment that may be needed/allowed during the conduct of the study (i.e. concomitant or rescue therapy)
Part	A sub-division of a study used to evaluate specific objectives or contain different populations. For example, one study could contain a single dose part and a multiple dose part, or a part in participants with established disease and in those with newly-diagnosed disease
Participant	A trial participant (can be a healthy volunteer or a patient). "Participant" terminology is used in the protocol whereas term "Subject" is used in data collection
Participant number	A unique number assigned to each participant upon signing the informed consent. This number is the definitive, unique identifier for the participant and should be used to identify the participant throughout the study for all data collected, sample labels, etc.
Patient-Reported Outcome (PRO)	A measurement based on a report that comes directly from the participant about the status of a participant's health condition without amendment or interpretation of the participant's report by a clinician or anyone else
Period	The subdivisions of the trial design (e.g. Screening, Treatment, Follow-up) which are described in the Protocol. Periods define the study phases and will be used in clinical trial database setup and eventually in analysis
Perpetrator drug	A drug which affects the pharmacokinetics of the other drug
Personal data	Participant information collected by the Investigator that is coded and transferred to Novartis/Sponsors for the purpose of the clinical trial. This data includes participant identifier information, study information and biological samples.
Randomization	The process of assigning trial participants to investigational drug or control/comparator drug using an element of chance to determine the assignments in order to reduce bias.
Randomization number	A unique identifier assigned to each randomized participant
Remote	Describes any trial activities performed at a location that is not the investigative site.
Rescreening	If a participant fails the initial screening and is considered as a Screen Failure, he/she can be invited once for a new Screening visit after medical judgment and as specified by the protocol
Screen Failure	A participant who did not meet one or more criteria that were required for participation in the study
Source Data/Document	Source data refers to the initial record, document, or primary location from where data comes. The data source can be a database, a dataset, a spreadsheet or even hard-coded data, such as paper or eSource
Start of the clinical trial	The start of the clinical trial is defined as the signature of the informed consent by the first participant.
Study treatment	Any drug or combination of drugs or intervention administered to the study participants as part of the required study procedures; includes investigational drug(s), control(s) or background therapy

Tele-visit	Procedures or communications conducted using technology such as telephone or video-conference, whereby the participant is not at the investigative site where the Investigator will conduct the trial.
Treatment arm/group	A treatment arm/group defines the dose and regimen or the combination, and may consist of 1 or more cohorts.
Treatment of interest	The treatment of interest and, as appropriate, the alternative treatment to which comparison will be made. These might be individual interventions, combinations of interventions administered concurrently, e.g. as add-on to standard of care, or might consist of an overall regimen involving a complex sequence of interventions. This is the treatment of interest used in describing the related clinical question of interest, which might or might not be the same as the study treatment.
Variable (or endpoint)	The variable (or endpoint) to be obtained for each participant that is required to address the clinical question. The specification of the variable might include whether the participant experiences an intercurrent event.
Withdrawal of consent	Withdrawal of consent from the study occurs when the participant explicitly requests to stop use of their data and/or biological samples AND no longer wishes to receive study treatment, AND does not agree to further protocol required assessments. This request should be in writing (depending on local regulations) and recorded in the source documentation.<br>This request should be distinguished from a request to discontinue the study. Other study participant's privacy rights are described in the corresponding informed consent form.

10.3 Appendix 3: Clinical laboratory tests

10.3.1 Clinically notable laboratory values and vital signs

Clinically notable laboratory abnormalities for selected tests performed by Central Laboratory (based on: 1) proportional or absolute changes from baseline; 2) the limit of the normal range established by the Central Laboratory; 3) cut-off values used to define Grade 3 as established by the Common Terminology Criteria for Adverse Events [CTCAE] Version 5.0 (US Department of Health and Human Services 2017) or higher).

Hematology

Table 10-1 Clinically notable hematology abnormalities

Parameter	Shift	Criteria
Red blood cell count	Increase<br>Decrease	> 50% increase AND value > ULN<br>> 30% decrease AND value < LLN
Hemoglobin	Increase<br>Decrease	> 50% increase AND value > ULN<br>either (> 30% decrease AND value < LLN) OR value < 8.0 g/dL
Hematocrit	Increase<br>Decrease	> 50% increase AND value > ULN<br>> 30% decrease AND value < LLN
White blood cell count	Increase<br>Decrease	value > 100,000 cells/mm^3<br>value < 2000 cells/mm^3
Platelet count	Decrease	value < 50,000 platelets/mm^3
ULN: upper limit of the normal range; LLN: lower limit of the normal range		

Blood chemistry

Table 10-2 Clinically notable blood chemistry abnormalities

Parameter	Shift	Criteria
Alkaline phosphatase	Increase	value > 5 x ULN if baseline was normal OR value > 5 x baseline if baseline was abnormal
ALT (SGPT)	Increase	value > 5 x ULN if baseline was normal OR value > 5 x baseline if baseline was abnormal
AST (SGOT)	Increase	value > 5 x ULN if baseline was normal OR value > 5 x baseline if baseline was abnormal
Total bilirubin	Increase	value > 3 x ULN if baseline was normal OR value > 3 x baseline if baseline was abnormal
BUN	Increase	≥ 50% increase
Creatinine	Increase	≥ 50% increase
Potassium	Increase	value > 6.0 mmol/L
	Decrease	value < 3.0 mmol/L
Chloride	Increase	> 115 mEq/L
	Decrease	< 90 mEq/L

**Parameter**	**Shift**	**Criteria**
Calcium	Increase	corrected serum calcium > 3.1 mmol/L
	Decrease	corrected serum calcium < 1.75 mmol/L
Uric acid	Increase	> 50% increase
Plasma glucose	Increase	> 25% increase
	Decrease	value < 40 mg/dL
ULN: upper limit of the normal range		

Along with investigators, the clinical trial team from Novartis will receive alerts for clinically notable values via fax/e-portal (or appropriate means agreed with central labs and Novartis).

10.4 Appendix 4: Participant Engagement

The following participant engagement initiatives are included in this study and will be provided, as available, for distribution to study participants at the time points indicated. If compliance is impacted by cultural norms or local laws and regulations, sites may discuss modifications to these requirements with Novartis.

- Thank You letter
- Plain language trial summary - after CSR publication

10.5 Appendix 5: Liver safety monitoring

10.5.1 Liver event and laboratory trigger definitions & follow-up requirements

Table 10-3 Liver event and laboratory trigger definitions

	Definition/threshold
If ALT or AST normal at baseline	
Liver laboratory triggers	· 3 x ULN < ALT or AST ≤ 5 x ULN<br>· TBL > ULN*
Liver events	· ALT or AST > 5 × ULN<br>· ALP > 2 × ULN (in the absence of known bone pathology)<br>· Total bilirubin > 3 × ULN (in the absence of known Gilbert syndrome)<br>· ALT or AST > 3 × ULN AND (TBL > 2 x ULN OR INR > 1.5)<br>· Potential Hy's Law cases (defined as ALT or AST > 3 × ULN and Total bilirubin > 2 × ULN [mainly conjugated fraction] without notable increase in ALP to > 2 × ULN)<br>· Any clinical event of jaundice (or equivalent term)<br>· ALT or AST > 3 × ULN accompanied by (general) malaise, fatigue, abdominal pain, nausea, or vomiting, or rash with eosinophilia<br>· Any adverse event potentially indicative of a liver toxicity
If ALT or AST elevated at baseline:	
Liver laboratory triggers	· ALT or AST > 2 x baseline or > 200 U/L (whichever occurs first)<br>· TBL > ULN*
Liver events	· ALT or AST > 3 x baseline or > 300 U/L (whichever occurs first)<br>· (ALT or AST > 2 × baseline or > 200 U/L [whichever occurs first]) AND (TBL > 2 x ULN OR INR > 1.5)<br>· ALT or AST > 5 × ULN<br>· ALP > 2 × ULN (in the absence of known bone pathology)<br>· Total bilirubin > 3 × ULN (in the absence of known Gilbert syndrome)<br>· Potential Hy's Law cases (defined as ALT or AST > 3 × ULN and Total bilirubin > 2 × ULN [mainly conjugated fraction] without notable increase in ALP to > 2 × ULN)<br>· Any clinical event of jaundice (or equivalent term)<br>· (ALT or AST > 2 × baseline or > 200 U/L [whichever occurs first]) accompanied by (general) malaise, fatigue, abdominal pain, nausea, or vomiting, or rash with eosinophilia<br>· Any adverse event potentially indicative of a liver toxicity

* Fractionate bilirubin, evaluate for cholestatic liver injury (ALP) or alternative causes of bilirubin elevation. Treat alternative causes according to local institutional guidelines

Table 10-4 Follow up requirements for liver laboratory triggers - ALT, AST, TBL

	ALT	TBL	Liver Symptoms	Action
**ALT increase without bilirubin increase:**				
	**If normal at baseline:**<br>ALT > 3 x ULN<br>**If elevated at baseline:**<br>ALT > 2 x baseline<br>or > 200 U/L (whichever occurs first)	Normal<br>For participants with Gilbert's syndrome: No change in baseline TBL	None	● **No change to study treatment**<br>● Measure ALT, AST, ALP, GGT, TBL, INR, albumin, CK, and GLDH in 48-72 hours.<br>● Follow-up for symptoms.
	**If normal at baseline:**<br>ALT > 5 x ULN for more than two weeks<br>**If elevated at baseline:**<br>ALT > 3 x baseline<br>or > 300 U/L (whichever occurs first) for more than two weeks	Normal<br>For participants with Gilbert's syndrome: No change in baseline TBL	None	● **Interrupt study treatment**<br>● Measure ALT, AST, ALP, GGT, TBL, INR, albumin, CK, and GLDH in 48-72 hours.<br>● Follow-up for symptoms.<br>● Initiate close monitoring and workup for competing etiologies.<br>● Study treatment can be restarted only if another etiology is identified and liver enzymes return to baseline.
	**If normal at baseline:**<br>ALT > 8 x ULN	Normal	None	
**ALT increase with bilirubin increase:**				
	**If normal at baseline:**<br>ALT > 3 x ULN<br>**If elevated at baseline:**<br>ALT > 2 x baseline<br>or > 200 U/L (whichever occurs first)	TBL > 2 x ULN (or INR > 1.5)<br>For participants with Gilbert's syndrome: Doubling of direct bilirubin	None	
	**If normal at baseline:**<br>ALT > 3 x ULN<br>**If elevated at baseline:**<br>ALT > 2 x baseline<br>or > 200 U/L (whichever occurs first)	Normal or elevated	Severe fatigue, nausea, vomiting, right upper quadrant pain	

Table 10-5 Follow-up requirements for liver laboratory triggers based on isolated elevations in TBL

Criterion	Actions required	Follow-up monitoring
**Total Bilirubin (isolated)**		
Any elevation > ULN	Fractionate bilirubin, evaluate for cholestatic liver injury (ALP) or alternative causes of bilirubin elevation. Treat alternative causes according to local institutional guidelines	As shown below according to the degree of elevation
>1.5 – 3.0 ULN	● Maintain treatment<br>● Repeat LFTs within 48-72 hours	Monitor LFTs weekly until resolution to ≤ Grade 1 or to baseline
> 3 - 10 × ULN (in the absence of known Gilbert syndrome)	● Interrupt treatment<br>● Repeat LFT within 48-72 hours<br>● Hospitalize if clinically appropriate<br>● Establish causality<br>● Record the AE and contributing factors (e.g. concomitant medications, medical history, lab test) in the appropriate CRF	Monitor LFTs weekly until resolution to ≤ Grade 1 or to baseline (ALT, AST, total bilirubin, Alb, PT/INR, ALP and GGT)<br>Test for hemolysis (e.g. reticulocytes, haptoglobin, unconjugated [indirect] bilirubin)
> 10 x ULN	● Discontinue the study treatment immediately after all other alternative causes are ruled out<br>● Record the AE and contributing factors(e.g. concomitant medications, medical history, lab test)in the appropriate CRF	Monitor ALT, AST, total bilirubin, Alb, PT/INR, ALP and GGT until resolution (frequency at investigator discretion)
Any AE potentially indicative of a liver toxicity	● Consider study treatment interruption or discontinuation<br>● Hospitalization if clinically appropriate<br>● Establish causality<br>● Record the AE and contributing factors(e.g. concomitant medications, medical history, lab test)in the appropriate CRF	Investigator discretion

10.6 Appendix 6: Renal safety monitoring

10.6.1 Specific Renal Alert Criteria and Actions and Event Follow-up

Table 10-6 Renal event criteria and corresponding actions to be taken upon their occurrence

Renal event	Actions
Confirmed serum creatinine increase of 25 – 49% from baseline	• Consider causes and possible interventions<br>• Follow-up within 2-5 days
Confirmed serum creatinine increase ≥ 50% from baseline (corresponds to KDIGO criterion for acute kidney injury)	• Consider causes and possible interventions<br>• Repeat assessment within 24-48h if possible<br>• Consider drug interruption or discontinuation unless other causes are diagnosed and corrected<br>• Consider participant hospitalization and specialized treatment
New onset dipstick proteinuria ≥ 300 mg/dL<br>OR<br>Protein-creatinine ratio (PCR) ≥ 1g/g Cr (or mg/mmol equivalent as converted by the measuring laboratory)	• Consider causes and possible interventions<br>• Assess serum albumin and serum total protein<br>• Repeat assessment to confirm<br>• Consider drug interruption or discontinuation unless other causes are diagnosed and corrected
New onset of hematuria ≥ 1 mg/dL on urine dipstick	• Repeat assessment to confirm<br>• Distinguish hemoglobinuria from hematuria<br>• Urine sediment microscopy<br>• Assess serum creatinine<br>• Exclude infection, trauma, bleeding from the distal urinary tract or bladder, menstruation<br>• Consider bleeding disorder

FOLLOW-UP OF RENAL EVENTS

Assess, document and record in CRF:

- Urine dipstick and sediment microscopy evidence of drug-induced nephrotoxicity (DIN): crystals, red blood cells (dysmorphic/glomerular vs. non-dysmorphic/non-glomerular), white blood cells, tubular epithelial cells
- Blood pressure and body weight
- Serum creatinine, BUN, electrolytes (sodium, potassium, phosphate, calcium) and uric acid
- Urine output

Review and record possible contributing factors to the renal event (co-medications, other co-morbid conditions) and additional diagnostic procedures (MRI etc.) in the CRF

Monitor participant regularly (frequency at investigator's discretion) until:

- Event resolution: (sCr within 10% of baseline or PCR < 1 g/g Cr, or ACR <300 mg/g Cr) or
- Event stabilization: sCr level with ±10% variability over last 6 months or protein-creatinine ratio stabilization at a new level with ±50% variability over last 6 months.
- Analysis of urine markers in samples collected over the course of the DIN event

10.7 Appendix 7: Managing hypotension

Investigators should monitor BP closely to identify episodes of symptomatic hypotension at each clinic visit. The occurrence of symptomatic hypotension should be reported as adverse events. Participants should be instructed to report to the site if they experience any symptoms relating to hypotension (i.e. giddiness, lightheadedness, syncope, falls, etc.) at their earliest convenience. The investigator should evaluate the events and take appropriate action. Investigator should exercise best clinical judgment based on laboratory assessments, volume status and any other clinical considerations in individual participants.

When deciding on how to modify background medication, it should be taken into account that XXB750, a mAb with a relatively long half-life, is dosed by subcutaneous injection every 4 weeks and, consequently, it is not possible to modify its dose with the intention of producing immediate relief/resolution of symptomatic hypotension or other AEs. Therefore, the recommended way of managing symptomatic hypotension is as follows:

As a general guidance, **persistent and troublesome symptoms attributed to hypotension**, regardless of blood pressure readings, **should be addressed**. In contrast, **asymptomatic low blood pressure is common in patients with HF** receiving conventional therapy and **does not usually require intervention.**

Ensure the participant is not dehydrated and if appropriate, consider **adjusting diuretic dose.**

Consider reducing or stopping non-HF medications that may be contributing to hypotension (e.g., calcium channel blockers, alpha-blockers, nitrates, etc.).

If symptoms persist despite the above steps, consider reducing the dose of HF treatments (e.g., ACEI/ARB, sacubitril/valsartan, MRA, SGLT2 inhibitor and beta-blocker) or sacubitril/valsartan if it is the randomized study medication (i.e., participant randomized to arm 5).

Heart failure medication should be up-titrated or reintroduced at the earliest time point possible per investigator's best clinical judgment.

Figure 10-1 Flow chart for managing hypotension

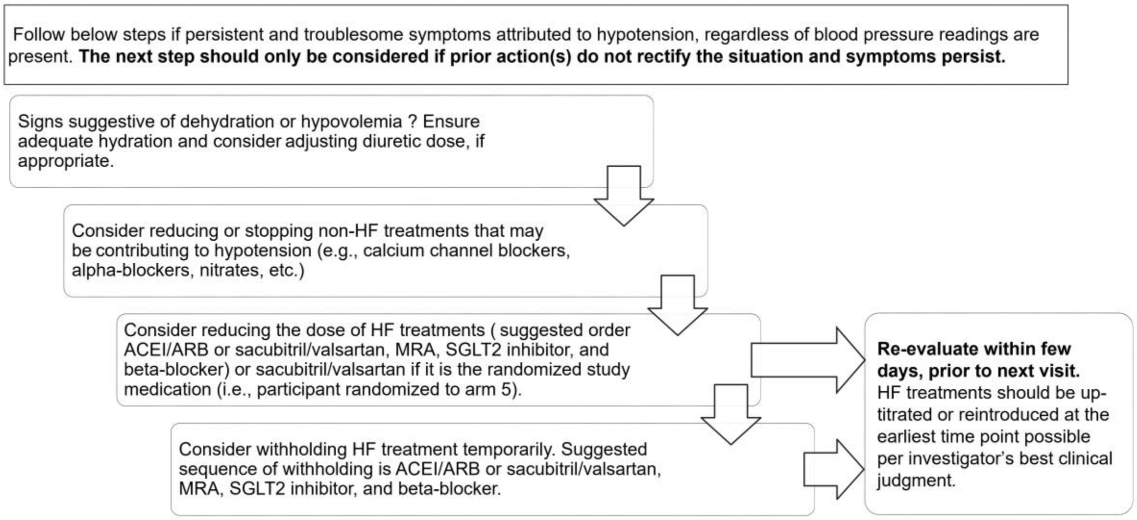

10.8 Appendix 8: Managing hyperkalemia

General principles

Elevation of potassium levels above the cut-off values defined below should be repeated and confirmed before any action is taken.

Any participant who presents with a potassium level > 5.4 mmol/L at screening, confirmed by repeat testing, should be considered a screen failure. Any participant with a serum potassium > 5.3 mmol/L after randomization requires regular, repeated checks of potassium concentration (beyond that required in the protocol) until it is clear that the potassium concentration is stable and not rising into the range of concern (≥ 5.5 and < 6.0 mmol/L) or of potential danger (≥ 6.0 mmol/L). Participants with an elevated potassium value should be managed according to the corrective actions outlined below. Hyperkalemia should be followed until resolution.

Corrective action for management of hyperkalemia

Serum potassium > 5.3 mmol/L and ≤ 5.5 mmol/L

- Confirm potassium concentration in a non-hemolyzed sample
- Reinforce low potassium diet and restriction of food/drinks with high potassium content (e.g. orange juice, melon, bananas, low-salt substitutes, etc.)
- Review medical regimen (including dietary supplements and over-the-counter medications) for agents known to cause hyperkalemia
- Consider reduction in dose or discontinuation of the following non-disease modifying agents:
  - Potassium-sparing diuretics (e.g., amiloride and triamterene) including when in combination products with thiazide or loop diuretics
  - Potassium supplements, e.g., potassium chloride
  - Salt substitutes
  - Trimethoprim and trimethoprim-containing combination products, such as Bactrim® and Septra® (trimethoprim/sulfamethoxazole fixed combination)
  - Non-steroidal anti-inflammatory drugs (NSAIDs)
  - Cyclo-oxygenase-2 (COX-2) inhibitors
  - Herbal Supplements: For example, Noni juice, alfalfa (*Medicagosativa*), dandelion (*Taraxacum officinale*), horsetail (*Equisetum arvense*), nettle (*Urtica dioica*), milkweed, lily of the valley, Siberian ginseng, hawthorn berries
- Consider down-titration of background HF agents:
  - Aldosterone antagonists (if they are believed to be the most likely cause of hyperkalemia)
  - Sacubitril/valsartan or ACEI/ARB, according to the investigator’s medical judgment
- Repeat serum potassium measurement within 3 to 5 days
- If serum potassium remains > 5.3 and ≤ 5.5 mmol/L, regularly monitor serum potassium levels to ensure stability (suggested once monthly)

Serum potassium > 5.5 and < 6.0 mmol/L

- Confirm potassium concentration in a non-hemolyzed sample
- Apply all measures outlined for serum potassium > 5.3 and ≤ 5.5 mmol/L
- Consider down-titration or discontinuation of background HF agents according to the investigator's medical judgment:
  - Aldosterone antagonists (if they are believed to be the most likely cause of hyperkalemia)
  - Sacubitril/valsartan or ACEI/ARB
- Repeat serum potassium measurement after 2-3 days
- If serum potassium < 5.5 mmol/L, consider resumption of background HF agent at a lower dose and repeat potassium measurement within 5 days

Serum potassium ≥ 6.0 mmol/L

- Immediately hold further administration of all non-disease modifying and disease-modifying medications known to cause hyperkalemia as listed above.
- Confirm potassium concentration in a non-hemolyzed sample
- Urgently evaluate patient and treat hyperkalemia as clinically indicated
- Apply all measures outlined for serum potassium > 5.3 and < 6.0 mmol/L

Discontinued background HF agents or study drug must not be resumed without individualized case discussion with, and permission from, Novartis medical monitor or his/her designee. In general, discontinued non-disease modifying agents known to cause hyperkalemia should not be resumed during the duration of the study.

11 References

References are available upon request.

Chen HH, Wan SH, Iyer SR, et al (2021) First-in-Human Study of MANP: A Novel ANP (Atrial Natriuretic Peptide) Analog in Human Hypertension. Hypertension; 78(6):1859-1867.

Crespo-Leiro MG, Anker SD, Maggioni AP, et al (2016) European Society of Cardiology Heart Failure Long-Term Registry (ESC-HF-LT): 1-year follow-up outcomes and differences across regions. Eur J Heart Fail; 18(6):613-25.

Desai AS, McMurray JJ, Packer M, et al (2015) Effect of the angiotensin-receptor-neprilysin inhibitor LCZ696 compared with enalapril on mode of death in heart failure patients. Eur Heart J; 36(30):1990-7.

Dong G, Qiu J, Wang D, et al (2018) The stratified win ratio. J Biopharm Stat; 28(4):778-96.

FDA (2021) Sponsor Responsibilities - Safety Reporting Requirements and Safety Assessment for IND and Bioavailability/Bioequivalence Studies. Guidance for Industry (draft guidance). US Department of Health and Human Services. FDA, CDER, CBER, Jun-2021. Drug Safety.

Fiuzat M, Hamo CE, Butler J, et al (2022) Optimal Background Pharmacological Therapy for Heart Failure Patients in Clinical Trials: JACC Review Topic of the Week. J Am Coll Cardiol; 79(5):504-10.

Gardner DG, Chen S, Glenn DJ, et al (2007) Molecular biology of the natriuretic peptide system: implications for physiology and hypertension. Hypertension; 49(3):419-26.

Green CP, Porter CB, Bresnahan DR, et al (2000) Development and evaluation of the Kansas City Cardiomyopathy Questionnaire: a new health status measure for heart failure. J Am Coll Cardiol; 35(5):1245-55.

Groenewegen A, Rutten FH, Mosterd A, et al (2020) Epidemiology of heart failure. Eur J Heart Fail; 22(8):1342-56.

Heidenreich PA, Bozkurt B, Aguilar D, et al (2022) 2022 AHA/ACC/HFSA Guideline for the Management of Heart Failure: A Report of the American College of Cardiology/American Heart Association Joint Committee on Clinical Practice Guidelines. J Am Coll Cardiol; 79(17):e263-e421.

Inker LA, Eneanya ND, Coresh J, et al (2021) New Creatinine- and Cystatin C-Based Equations to Estimate GFR without Race. N Engl J Med; 385(19):1737-49.

Januzzi JL, Zannad F, Anker SD, et al (2021) Prognostic Importance of NT-proBNP and Effect of Empagliflozin in the EMPEROR-Reduced Trial. J Am Coll Cardiol; 78(13):1321-32.

Lawson CA, Zaccardi F, Squire I, et al (2019) 20-year trends in cause-specific heart failure outcomes by sex, socioeconomic status, and place of diagnosis: a population-based study. Lancet Public Health; 4(8):e406-e420.

Levin ER, Gardner DG, Samson WK (1998) Natriuretic peptides. N Engl J Med; 339(5):321-8.

McDonagh TA, Metra M, Adamo M, et al (2021) 2021 ESC Guidelines for the diagnosis and treatment of acute and chronic heart failure. Eur Heart J; 42(36):3599-726.

McMurray JJ, Packer M, Desai AS, et al (2014) Angiotensin-neprilysin inhibition versus enalapril in heart failure. N Engl J Med; 371(11):993-1004.

Pandey KN (2008) Emerging Roles of Natriuretic Peptides and their Receptors in Pathophysiology of Hypertension and Cardiovascular Regulation. J Am Soc Hypertens; 2(4):210-26.

Pinheiro J, Bornkamp B, Bretz F (2006) Design and analysis of dose-finding studies combining multiple comparisons and modeling procedures. J Biopharm Stat; 16(5):639-56.

Pinheiro J, Bornkamp B, Glimm E, et al (2014) Model-based dose finding under model uncertainty using general parametric models. Stat Med; 33(10):1646-61.

Pocock SJ, Ariti CA, Collier TJ, et al (2012) The win ratio: a new approach to the analysis of composite endpoints in clinical trials based on clinical priorities. Eur Heart J; 33(2):176-82.

Roger VL (2021) Epidemiology of Heart Failure: A Contemporary Perspective. Circ Res; 128(10):1421-34.

Savarese G, Becher PM, Lund LH, et al (2022) Global burden of heart failure: A comprehensive and updated review of epidemiology. Cardiovasc Res; 118(17):3272-87.

Schmitt W, Rühs H, Burghaus R, et al (2021) NT-proBNP Qualifies as a Surrogate for Clinical End Points in Heart Failure. Clin Pharmacol Ther; 110(2):498-507.

Solomon SD, Vaduganathan M, L Claggett B, et al (2020) Sacubitril/Valsartan Across the Spectrum of Ejection Fraction in Heart Failure. Circulation; 141(5):352-61.

Tromp J, Ouwerkerk W, van Veldhuisen DJ, et al (2022) A Systematic Review and Network Meta-Analysis of Pharmacological Treatment of Heart Failure With Reduced Ejection Fraction. JACC Heart Fail; 10(2):73-84.

US Department of Health and Human Services, National Institutes of Health, National Cancer Institute. (2017) Common Terminology Criteria for Adverse Events, Version 5.0.

Vaduganathan M, Claggett B, Packer M, et al (2018) Natriuretic Peptides as Biomarkers of Treatment Response in Clinical Trials of Heart Failure. JACC Heart Fail; 6(7):564-69.

Vaduganathan M, Claggett BL, Jhund PS, et al (2020) Estimating lifetime benefits of comprehensive disease-modifying pharmacological therapies in patients with heart failure with reduced ejection fraction: a comparative analysis of three randomised controlled trials. Lancet; 396(10244):121-28.

Zile MR, Claggett BL, Prescott MF, et al (2016) Prognostic Implications of Changes in N-Terminal Pro-B-Type Natriuretic Peptide in Patients With Heart Failure. J Am Coll Cardiol; 68(22):2425-36.

Signature Page for: Protocol Amendment_-_V02_-_23 Feb 2024
VV-TMF-1272573 v 1 . 0

Reason for signing: I confirm this document is ready for submission/publication	Name: Thomas Nuehrenberg<br>Role: Approver<br>Date of signature: 23-Feb-2024 19:32:51 GMT+0000

Signature Page for: Protocol Amendment_-_V02_-_23 Feb 2024
VV-TMF-1272573 v 1 . 0
