# Supplementary material for: The NPR1 agonist antibody XXB750 in heart failure: a phase 2 randomized trial
Source: Nat Med. 2026 Mar 30;32(5):1694–700. doi: 10.1038/s41591-026-04313-w (PMC13190253; doi:10.1038/s41591-026-04313-w)
Supplement: Supplementary file 2 — Reporting summary [file 41591_2026_4313_MOESM2_ESM.pdf]

Corresponding author(s): Scott D. Solomon, MD

Last updated by author(s): Feb 15, 2026

Reporting Summary

Nature Portfolio wishes to improve the reproducibility of the work that we publish. This form provides structure for consistency and transparency in reporting. For further information on Nature Portfolio policies, see our Editorial Policies and the Editorial Policy Checklist.

Statistics

For all statistical analyses, confirm that the following items are present in the figure legend, table legend, main text, or Methods section.

n/a	Confirmed	
☐	☒	The exact sample size (*n*) for each experimental group/condition, given as a discrete number and unit of measurement
☒	☐	A statement on whether measurements were taken from distinct samples or whether the same sample was measured repeatedly
☐	☒	The statistical test(s) used AND whether they are one- or two-sided<br>*Only common tests should be described solely by name; describe more complex techniques in the Methods section.*
☐	☒	A description of all covariates tested
☐	☒	A description of any assumptions or corrections, such as tests of normality and adjustment for multiple comparisons
☐	☒	A full description of the statistical parameters including central tendency (e.g. means) or other basic estimates (e.g. regression coefficient) AND variation (e.g. standard deviation) or associated estimates of uncertainty (e.g. confidence intervals)
☐	☒	For null hypothesis testing, the test statistic (e.g. *F*, *t*, *r*) with confidence intervals, effect sizes, degrees of freedom and *P* value noted<br>*Give P values as exact values whenever suitable.*
☒	☐	For Bayesian analysis, information on the choice of priors and Markov chain Monte Carlo settings
☒	☐	For hierarchical and complex designs, identification of the appropriate level for tests and full reporting of outcomes
☐	☒	Estimates of effect sizes (e.g. Cohen's *d*, Pearson's *r*), indicating how they were calculated

Our web collection on statistics for biologists contains articles on many of the points above.

Software and code

Policy information about availability of computer code

Data collection	Data were collected at study sites as described and managed by the sponsor. The sponsor provided all data to the academic authors who performed all analyses in this manuscript.
Data analysis	Statistical analyses were conducted using STATA version 19.5.

For manuscripts utilizing custom algorithms or software that are central to the research but not yet described in published literature, software must be made available to editors and reviewers. We strongly encourage code deposition in a community repository (e.g. GitHub). See the Nature Portfolio guidelines for submitting code & software for further information.

Data

Policy information about availability of data

All manuscripts must include a data availability statement. This statement should provide the following information, where applicable:

- Accession codes, unique identifiers, or web links for publicly available datasets
- A description of any restrictions on data availability
- For clinical datasets or third party data, please ensure that the statement adheres to our policy

The methods and data sets generated and analyzed during the current study are not publicly available. The sponsor (Novartis) is committed to sharing access to patient-level data and supporting clinical documents from eligible studies with qualified external researchers. These requests are reviewed and approved based on

scientific merit. All data provided are anonymized to respect the privacy of patients who have participated in the trial, in line with applicable laws and regulations.The data can be requested from the corresponding author of the article.

Research involving human participants, their data, or biological material

Policy information about studies with human participants or human data. See also policy information about sex, gender (identity/presentation), and sexual orientation and race, ethnicity and racism.

Reporting on sex and gender	The n (%) of males/females was described overall, by randomized therapy. Treatment effects on the primary endpoint were not assessed separately in males and females.
Reporting on race, ethnicity, or other socially relevant groupings	The n (%) of each racial category (White, Black, Asian) was described by randomized therapy . Race was a prespecified subgroup and treatment effects on the primary endpoint were not assessed separately by racial category.
Population characteristics	Patients with heart failure and reduced or mildly reduced ejection fraction.
Recruitment	Recruitment occured at 62 sites in 12 countries worldwide.
Ethics oversight	The trial protocols were approved by ethics committees or institutional review boards at all participating sites and all patients provided explicit written informed consent.

Note that full information on the approval of the study protocol must also be provided in the manuscript.

Field-specific reporting

Please select the one below that is the best fit for your research. If you are not sure, read the appropriate sections before making your selection.

☒ Life sciences ☐ Behavioural & social sciences ☐ Ecological, evolutionary & environmental sciences

For a reference copy of the document with all sections, see nature.com/documents/nr-reporting-summary-flat.pdf

Life sciences study design

All studies must disclose on these points even when the disclosure is negative.

Sample size	Sample size consisted of 136 randomized patients of whom, one patient was mis-randomized and excluded from further analysis, leaving 135 patients in the final analysis set.
Data exclusions	One patient who was misrandomized was excluded from the analysis (see consort diagram)
Replication	Several measures of efficacy and safety were available and assessed.
Randomization	Individuals who met eligibility criteria who were on background ACEi or ARB were randomly allocated to receive blinded subcutaneous XXB750 60mg doses, XXB750 titrated to a maximum dose of 120mg, or matching subcutaneous placebo, or open-label oral sacubitril/valsartan. Those who were on background sacubitril/valsartan were randomly allocated to either blinded XXB750 60mg doses, XXB750 titrated to a maximum dose of 120mg, or matching placebo (Figure S1). Randomization was stratified by region and ACEi/ARB vs. sacubitril/valsartan background therapy to ensure that the XXB750 or placebo arms had approximately 2/3 patients on background sacubitril/valsartan therapy. Participants randomized to XXB750, or its matching placebo, received one subcutaneous injection every 4 weeks, with a total of 4 injections during the 16-week treatment period, while participants randomized to open-label sacubitril/valsartan were titrated, where possible, to a target dose of 97/103 mg twice daily for 16 weeks.
Blinding	Patients who were on background ACE inhibititors or ARBs were randomly allocated to receive BLINDED XXB or matching placebo OR open-label sacubitril/valsartan, while those on background sacubitril/valsartan were randomly allocated to blinded XXB750 in one of two doses or matching placebo.

Reporting for specific materials, systems and methods

We require information from authors about some types of materials, experimental systems and methods used in many studies. Here, indicate whether each material, system or method listed is relevant to your study. If you are not sure if a list item applies to your research, read the appropriate section before selecting a response.

Materials & experimental systems

n/a	Involved in the study
☒	☐ Antibodies
☒	☐ Eukaryotic cell lines
☒	☐ Palaeontology and archaeology
☒	☐ Animals and other organisms
☐	☒ Clinical data
☒	☐ Dual use research of concern
☒	☐ Plants

Methods

n/a	Involved in the study
☒	☐ ChIP-seq
☒	☐ Flow cytometry
☒	☐ MRI-based neuroimaging

Clinical data

Policy information about clinical studies

All manuscripts should comply with the ICMJE guidelines for publication of clinical research and a completed CONSORT checklist must be included with all submissions.

Clinical trial registration: Clinicaltrials.gov NCT06142383

Study protocol: The study protocol and statistical analysis plan were prespecified and available online.

Data collection: 136 Participants in this trial were randomized between December 27, 2023 and August 8, 2024 from 62 clinical sites in 12 countries.

Outcomes: The primary objective of the study was to evaluate the efficacy and dose-response relationship of the three XXB750 target dose levels compared to placebo in reducing NT-proBNP, reflecting a reduction in left ventricular wall stress from baseline to Week 16. Secondary objectives included evaluating the effect on NT-proBNP from adding the two highest doses of XXB750 to background ACEi/ARB treatment, compared to switching to sacubitril/valsartan. Safety assessments included adverse events, serious adverse events, and adverse events of special interest, including serious hypotension, presyncope/syncope, hypersensitivity reactions, injection site reactions, and severe tachycardia or bradycardia.

Plants

Seed stocks: *Report on the source of all seed stocks or other plant material used. If applicable, state the seed stock centre and catalogue number. If plant specimens were collected from the field, describe the collection location, date and sampling procedures.*

Novel plant genotypes: *Describe the methods by which all novel plant genotypes were produced. This includes those generated by transgenic approaches, gene editing, chemical/radiation-based mutagenesis and hybridization. For transgenic lines, describe the transformation method, the number of independent lines analyzed and the generation upon which experiments were performed. For gene-edited lines, describe the editor used, the endogenous sequence targeted for editing, the targeting guide RNA sequence (if applicable) and how the editor was applied.*

Authentication: *Describe any authentication procedures for each seed stock used or novel genotype generated. Describe any experiments used to assess the effect of a mutation and, where applicable, how potential secondary effects (e.g. second site T-DNA insertions, mosiacism, off-target gene editing) were examined.*
